# Innovative Strategy toward Mutant CFTR Rescue in Cystic Fibrosis: Design and Synthesis of Thiadiazole Inhibitors of the E3 Ligase RNF5

**DOI:** 10.1021/acs.jmedchem.3c00608

**Published:** 2023-07-13

**Authors:** Irene Brusa, Elvira Sondo, Emanuela Pesce, Valeria Tomati, Dario Gioia, Federico Falchi, Beatrice Balboni, Jose Antonio Ortega Martínez, Marina Veronesi, Elisa Romeo, Natasha Margaroli, Maurizio Recanatini, Stefania Girotto, Nicoletta Pedemonte, Marinella Roberti, Andrea Cavalli

**Affiliations:** † Department of Pharmacy and Biotechnology, University of Bologna, 40126 Bologna, Italy; ‡ Computational & Chemical Biology, Istituto Italiano di Tecnologia, 16163 Genova, Italy; § UOC Genetica Medica, IRCCS Istituto Giannina Gaslini, 16147 Genova, Italy; ∥ Structural Biophysics and Translational Pharmacology Facility, Istituto Italiano di Tecnologia, 16163 Genova, Italy; ⊥ Centre Européen de Calcul Atomique et Moléculaire, EPFL CECAM, 1015 Lousanne, Switzerland

## Abstract

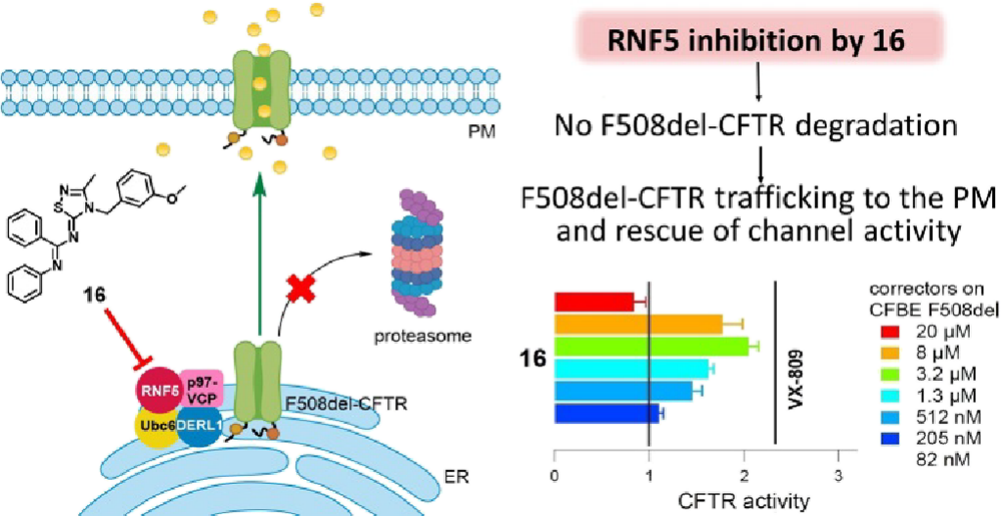

In cystic fibrosis (CF), deletion of phenylalanine 508 (F508del) in the CF transmembrane conductance regulator (CFTR) is associated to misfolding and defective gating of the mutant channel. One of the most promising CF drug targets is the ubiquitin ligase RNF5, which promotes F508del-CFTR degradation. Recently, the first ever reported inhibitor of RNF5 was discovered, i.e., the 1,2,4-thiadiazol-5-ylidene **inh-2**. Here, we designed and synthesized a series of new analogues to explore the structure–activity relationships (SAR) of this class of compounds. SAR efforts ultimately led to compound **16**, which showed a greater F508del-CFTR corrector activity than **inh-2**, good tolerability, and no toxic side effects. Analogue **16** increased the basal level of autophagy similar to what has been described with RNF5 silencing. Furthermore, co-treatment with **16** significantly improved the F508del-CFTR rescue induced by the triple combination elexacaftor/tezacaftor/ivacaftor in CFBE41o^–^ cells. These findings validate the 1,2,4-thiadiazolylidene scaffold for the discovery of novel RNF5 inhibitors and provide evidence to pursue this unprecedented strategy for the treatment of CF.

## 1 Introduction

Cystic fibrosis (CF) is the most common genetic disorder in Caucasian populations^1^ caused by loss of function mutations in the *CFTR* gene encoding for the cystic fibrosis transmembrane conductance regulator (CFTR) protein.^2^ CFTR is a cAMP-regulated anion channel of primary importance for transepithelial chloride and bicarbonate ion transport in various organs, where it contributes to regulate salt and fluid homeostasis.^3^ While CF is a multisystem disease, the main clinical features are exocrine pancreatic insufficiency and bronchiectasis with chronic airway infection leading to respiratory failure and premature death.^4^ Until 10 years ago, the conventional therapy in use for CF was primarily based on controlling disease symptoms. Nowadays, the improved understanding of CFTR protein structure and of the consequence of gene mutations has opened the way to personalized treatments targeting specific defects.^5^

Currently, over 2000 different mutations in the CFTR have been described, although a pathogenetic role has been demonstrated only for approx. 400 of them, as reported in the Clinical and Functional Translation of CFTR2 database (https://cftr2.org, accessed on 23/03/2023). However, the most prevalent mutation is the deletion of a phenylalanine at position 508 (F508del), which affects ∼80% of CF patients worldwide, although with marked differences in frequency based on the ethnic origin. F508del-CFTR is responsible for three distinct defects of the mutant protein: (i) a trafficking defect due to misfolding of the F508del-CFTR, resulting in a reduced amount of channel present at the plasma membrane (PM);^6−8^ (ii) a decreased stability when the mutated channel is expressed on the plasma membrane;^9^ and (iii) a channel gating defect due to the reduced open-channel probability.^10−12^ Noteworthily, both F508del defects can be rescued, at least partially, using two classes of small-molecule CFTR modulators: correctors can help the transport of the misfolded CFTR to the cell surface,^13^ and potentiators can ameliorate the gating defect, helping to keep this ion channel open at the cell surface.^14^ Hence, combination therapies involving small molecules that synergistically aim at distinct structural defects are likely required to promote a marked F508del rescue.^15^

**Figure 1:**
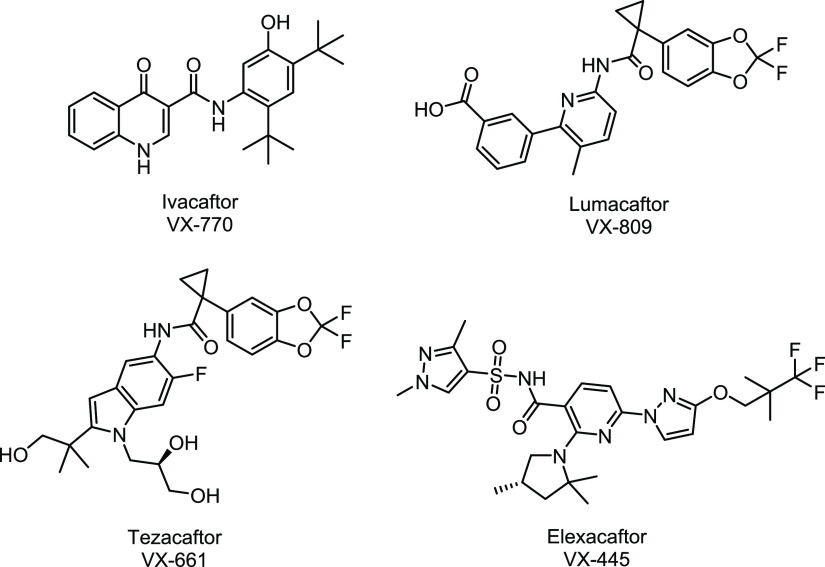
Structures of potentiators and correctors clinically approved.

Intense research efforts in the CFTR modulators field resulted in the registration in 2012 of the potentiator ivacaftor (VX-770, Figure 1)^16−18^ under the trade name Kalydeco for patients with at least one copy of the G551D mutation, subsequently expanded to a selection of class III and IV mutations. It followed the 2015 marketing approval of the fixed dose combination Orkambi composed of ivacaftor and the corrector lumacaftor (VX-809, Figure 1)^19^ for CF patients carrying F508del mutation.^19,20^ Tezacaftor, also known as VX-661 (Figure 1), is an analogue of lumacaftor with improved pharmacokinetics and less side effects. The tezacaftor/ivacaftor co-therapy (trade name Symdeko) received marketing authorization in 2018 for both F508del homozygous patients and heterozygous F508del with G551D or with residual function mutations.^21−24^ More recently, Vertex Pharmaceuticals developed the next generation corrector elaxacaftor (VX-445, Figure 1), which showed additive or synergistic effects in combination with a first generation corrector (lumacaftor and tezacaftor) and with the potentiator ivacaftor.^25,26^ Strikingly, ivacaftor, tezacaftor, and elexacaftor are now included in the triple drug combination Trikafta for the treatment of CF patients aged 12 years and older carrying at least one F508del mutation or another mutation included in the list of 178 variants considered to be eligible to drug treatment (for the complete list of mutations, see Trikafta.com).^27−29^

Although both Orkambi and Symdeko have limited effects in clinical use,^30^ they still represent the standard care for many CF patients. Trikafta, despite undoubtedly representing a breakthrough in CF treatment by significantly slowing down CF progress with substantiated clinical benefits,^31^ fails to fully restore mutant CFTR function.^26,32^ As an example, treatment with Trikafta reduces only partially the ubiquitylation status of F508del-CFTR.^32^ Therefore, both academies and pharmaceutical companies have been involved in searching for small-molecule correctors^33^ and potentiators^34^ with different mechanisms or with ameliorated characteristics. Encouragingly, a number of emerging CFTR modulators are currently in the pipeline for preclinical models and early phase clinical trials, strengthening the restoration of CFTR function as a new therapeutic solution for CF.^35^ Moreover, a great part of CF research is now focusing on the discovery of active compounds affecting different CFTR-related targets (namely, proteostasis regulators), which can modify the CFTR proteostasis environment leading to beneficial effects on CFTR maturation and trafficking to the PM.^36,37^ This innovative strategy holds great promise as it can specifically target the steps in CFTR processing that create the main bottlenecks in its rescue. Furthermore, proteostasis regulator effects were seen to be additive with those of other types of correctors and therefore they may be useful to optimize combination therapies, especially for patients with mutations that still lack effective treatments.^37^

Several proteins have been identified that could represent useful drug targets for a CF therapy based on proteostasis modulation.^36^ Among them, the ubiquitin ligase RNF5/RMA1 is particularly interesting as it acts at early stages of CFTR biosynthesis and its loss by gene silencing synergizes with pharmacological correctors to correct folding defects in F508del-CFTR.^38^ Our group previously demonstrated that genetic suppression of RNF5 *in vivo* leads to an attenuation of intestinal pathological phenotypes due to malabsorption in F508del-CFTR mice and concomitantly increases CFTR activity in intestinal epithelial cells. This work validates the relevance of RNF5 as a novel drug target for CF, providing a strong basis for developing small molecules to inhibit RNF5 activity.^39^

**Figure 2:**
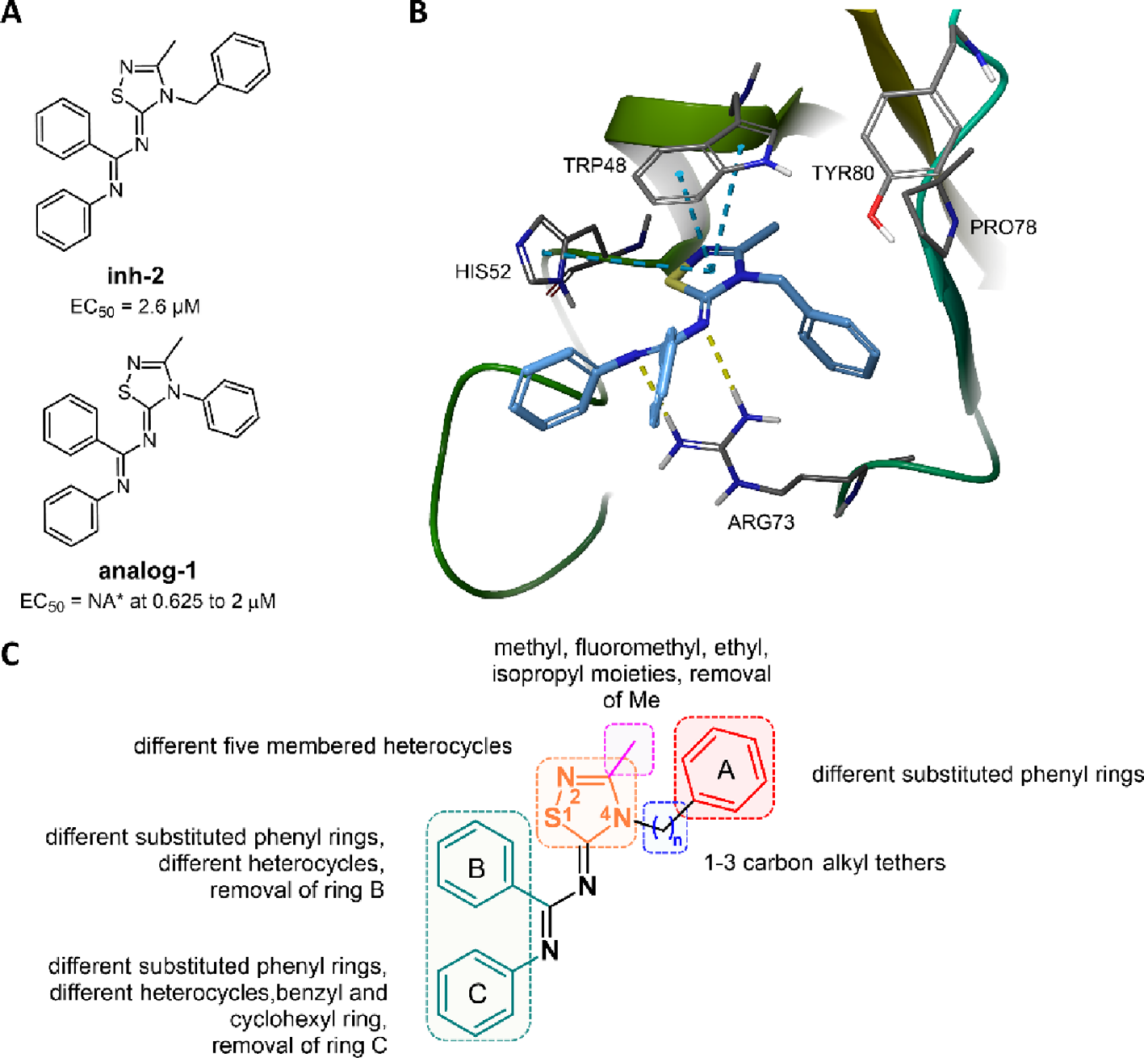
Discovery of the first RNF5 inhibitor, the 1,2,4-thiadiazolylidene **inh-2** by Sondo *et al.*(40) and chemical modification campaign around the 1,2,4-thiadiazol-5-ylidene scaffold. (A) Chemical structures and F508del-CFTR corrector activity of the RNF5 inhibitor **inh-2** and the RNF5 activator **analog-1**. NA = not active. (B) Proposed binding mode of **inh-2** into the RNF5 pocket. Blue dashed lines indicate the π–π stacking interactions, while yellow dashed lines indicate the hydrogen bonds. (C) Overview of the optimization strategy of **inh-2** for SAR exploration.

As a further development of this project, using a computational approach based on ligand docking and virtual screening (VS), we recently identified the 1,2,4-thiadiazole derivative **inh-2** (Figure 2A), a drug-like small molecule able to act as an RNF5 inhibitor. In *in vitro* experiments, **inh-2** rescued F508del-CFTR activity in both CFBE41o^–^ cells and human primary bronchial epithelia. Analysis of the **inh-2** mechanism of action confirmed that it decreases ubiquitination and increases half-life of F508del-CFTR, further validating RNF5 as a drug target for CF and providing evidence to support its druggability.^40^

Besides CF, given its important regulatory role in controlling cell differentiation, growth, and transformation, and its aberrant expression, RNF5 can be considered an interesting drug target also in pathological conditions, such as tumorigenesis.^41^ Previous studies identified an upregulation of RNF5 in breast cancer^41^ and tumor cell proliferation were inhibited after silencing of RNF5. Recently, RNF5 was correlated with glioma.^42^ In addition, modulation of RNF5 was demonstrated to be an effective treatment in neuroectodermal tumors.^43^ Taken together, these studies identify RNF5 as a valid candidate for the development of anti-cancer therapies.

Recently, a small-molecule inhibitor and degrader of RNF5 was discovered based on its ability to inhibit misfolded proteins from the ER lumen to the cytosol and to negatively regulate the RNF5 function.^44^ This finding further supports RNF5 druggability.

To discover more effective compounds, we here design and synthesize a library of new analogues (**1**–**46**, Table 1) of the 1,2,4-thiadiazole **inh-2**. In particular, we attempt to depict general structure–activity relationships (SAR) of **1**–**46** in inhibiting RNF5 and outline the biological profile of the most promising derivatives **6**, **9**–**11**, **14**, **16**, **17**, **19**, **21**–**25**, **27**–**29**, and **34**.

**Table 1:**
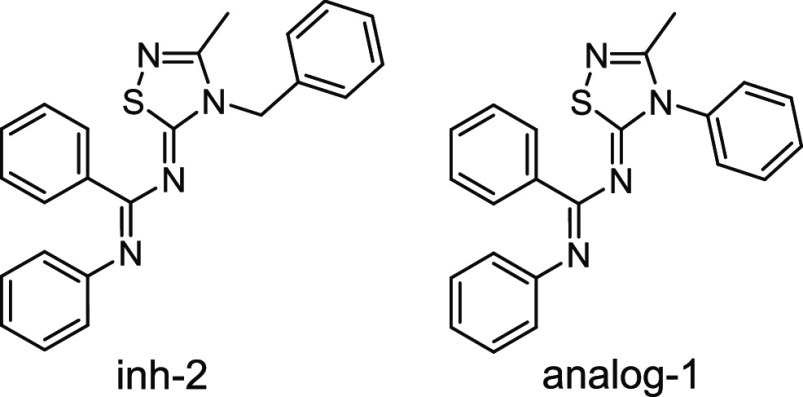

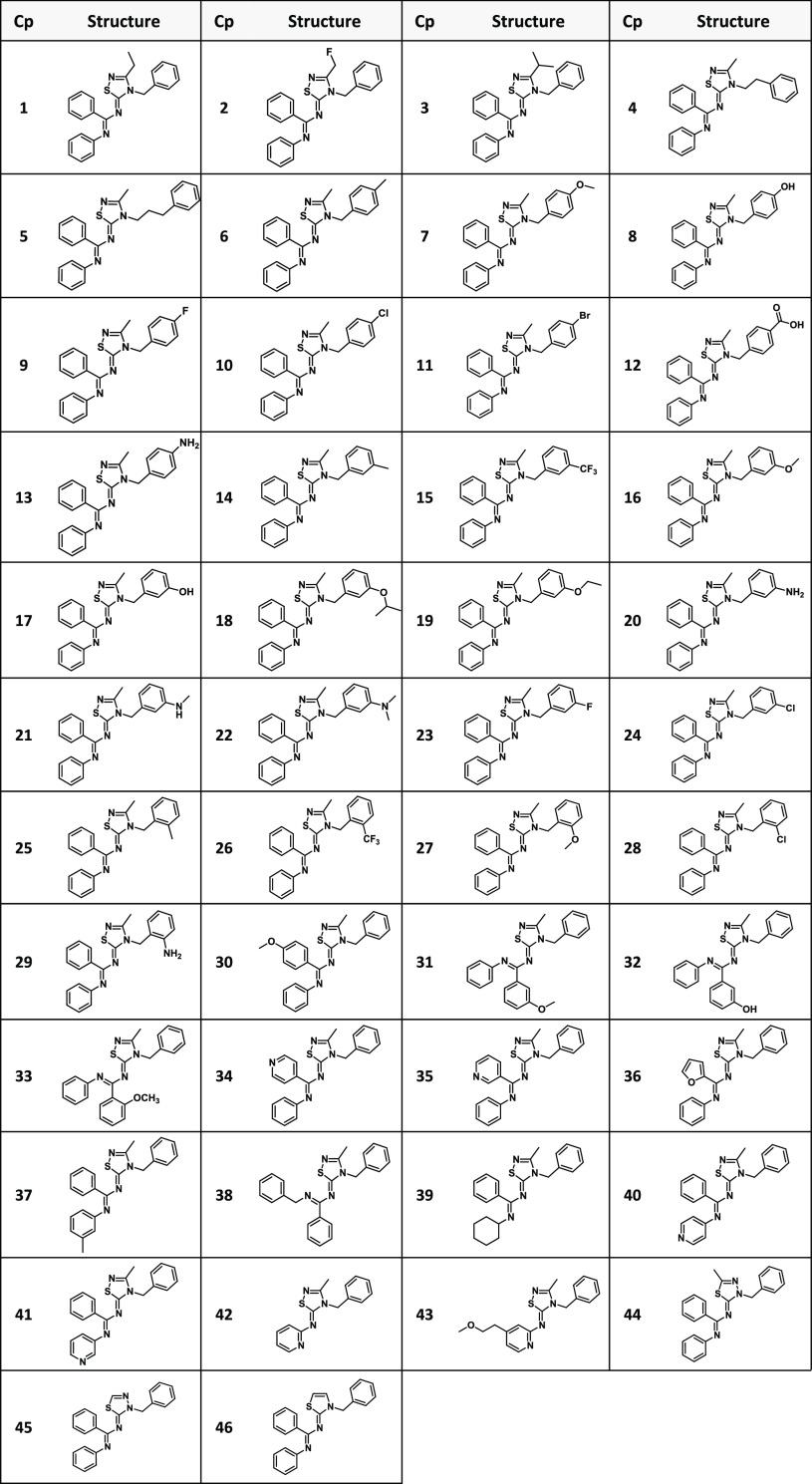
Structures of **inh-2**, **Analog-1**, and Compounds **1**–**46**

## 2 Results and Discussion

### 2.1 Design Approach

From a computational point of view, human RNF5 is a very challenging target; as to date, there are no structures available in the PDB of this E3 ligase. Moreover, there is very low identity with similar protein in the PDB for homology modeling endeavors. Therefore, to identify potential RNF5 inhibitors in a previous paper, we used two complementary approaches.^40^ First, we generated a homology model of RNF5 RING domain to perform VS based on ligand docking. In parallel, we used molecular fingerprinting to select a diversity set of compounds. With this strategy, we discovered the first ever reported RNF5 inhibitor **inh-2** based on a 1,2,4-thiadiazole moiety, which displayed an EC_50_ of 2.6 μM in CFBE41o^–^ cells from the HS-YFP assay.^40^ Notably, the same study showed that a close analog of **inh-2** (**analog-1**, Figure 2A) had no activity as a CFTR corrector, whereas it elicited the opposite effects on RNF5 downstream targets as compared with **inh-2**, suggesting that small differences in the chemical structure may shift the effect of **inh-2** analogues from RNF5 inhibition to activation. Furthermore, our group recently demonstrated that the RNF5 activator **analog-1** can reduce neuroblastoma and melanoma tumor growth, both *in vitro* and *in vivo* models, suggesting that the activation of RNF5 may represent a potential anti-tumor treatment strategy.^43^ On the other hand, the biological effects of **inh-2** are consistent with what has been described for RNF5 inhibition,^39,40^ although we cannot exclude that **inh-2** may also affect other cellular targets. Therefore, **inh-2** structural tuning is mandatory to gain a deeper knowledge on the handling of misfolded CFTR mutants by the quality control system of the cell.

The proposed binding mode based on docking simulations of **inh-2** to the homology model of RNF5 shows (i) a H-bond between the amidine portion of the compound and ARG73, (ii) two π–π interactions among the thiadiazolidine, phenyl ring B, TRP48, and HIS52, and (iii) some hydrophobic interactions between the benzyl ring A and the hydrophobic pocket outlined by LEU51, VAL38, and VAL76 (Figure 2B). For a comparison with a hypothetical binding mode of **analog-1**, see Figure S1. However, despite the substantial margins of uncertainty of the docking pose of **inh-2** due to the flexibility of RNF5, this binding mode offers the possibility to rationally modify it. Herein, to improve the inhibitory activity of **inh-2**, we conducted a chemical modification campaign around the 1,2,4-thiadiazol-5-ylidene scaffold. Figure 2C provides an overview of the structural variations introduced on the thiadiazole scaffold.

As the 3-methyl group of the thiadiazolidine central ring (pink region, Figure 2C) is shown to lie in a small hydrophobic pocket of the target, we first defined the optimal steric hindrance of this position by replacing the methyl group with ethyl, fluoromethyl, and isopropyl moieties (**1**–**3**, Table 1). Unfortunately, the removal of the methyl group was not possible due to poor chemical tractability of the 5-amino-1,2,4-thiadiazole and to the low reactivity of the functionalizable nitrogen atom of the ring. In the attempt to find the proper length of the alkyl chain connecting the thiadiazolidine and the phenyl ring A (blue region, Figure 2C), we replaced the methylene of **inh-2** with ethylene or propylene tethers (**4** and **5**, Table 1). Ring A (red region, Figure 2C) is shown to interact with a large hydrophobic pocket of the target from the docking simulation (Figure 2B). Therefore, we investigated the role of this portion in possible hydrophobic interactions by introducing different EDGs and EWGs, such as methyl, methoxyl, hydroxyl, carboxyl, ethoxyl, isopropoxyl, trifluoromethyl, amino, methylamino, dimethylamino groups, and fluorine, chlorine, and bromine atoms in *ortho*, *meta*, or *para* positions (**6**–**29**, Table 1). The phenyl ring B was modified by introducing methoxyl or hydroxyl groups at different positions (**30**–**33**, Table 1) or replacing by 4-pyridyl, 3-pyridyl, or 2-furanyl moieties (**34**–**36**, Table 1). Indeed, it was reasoned that proton acceptor or donor groups on ring B could engage favorable interactions with HIS52 of the site, while different heterocycles could stabilize the T-shape-type π–π stacking interaction with TRP48. To assess the importance of ring C, the phenyl was replaced by *m*-tolyl, benzyl, cyclohexyl, and pyridyl moieties (**37**–**41**). To further explore the role of the *N*-phenylbenzamidine portion in possible π–π stacking interactions (green region, Figure 2C), both rings B and C were removed and replaced by pyridin-2-yl and 4-(2-methoxyethyl)pyridin-2-yl moieties (**42** and **43**, Table 1). Indeed, as suggested by docking simulation, the pyridin-2-yl group should maintain the H-bond stacking interaction with ARG73, while the methoxyethyl moiety could engage favorable H-bonds with HIS52. Last, modifications of the central 3-methyl-1,2,4-thiadiazolidine core (orange region, Figure 2C) were envisioned to investigate if different five-membered heterocycles could affect the π–π stacking interaction with TRP48 and HIS52 and therefore the inhibitory activity. Herein, 5-methyl-1,3,4-thiadiazolidine, 1,3,4-thiadiazolidine, and 1,3-thiazolidine moieties were explored at this position (**44**–**46**, Table 1).

### 2.2 Chemistry

**Scheme 1:**
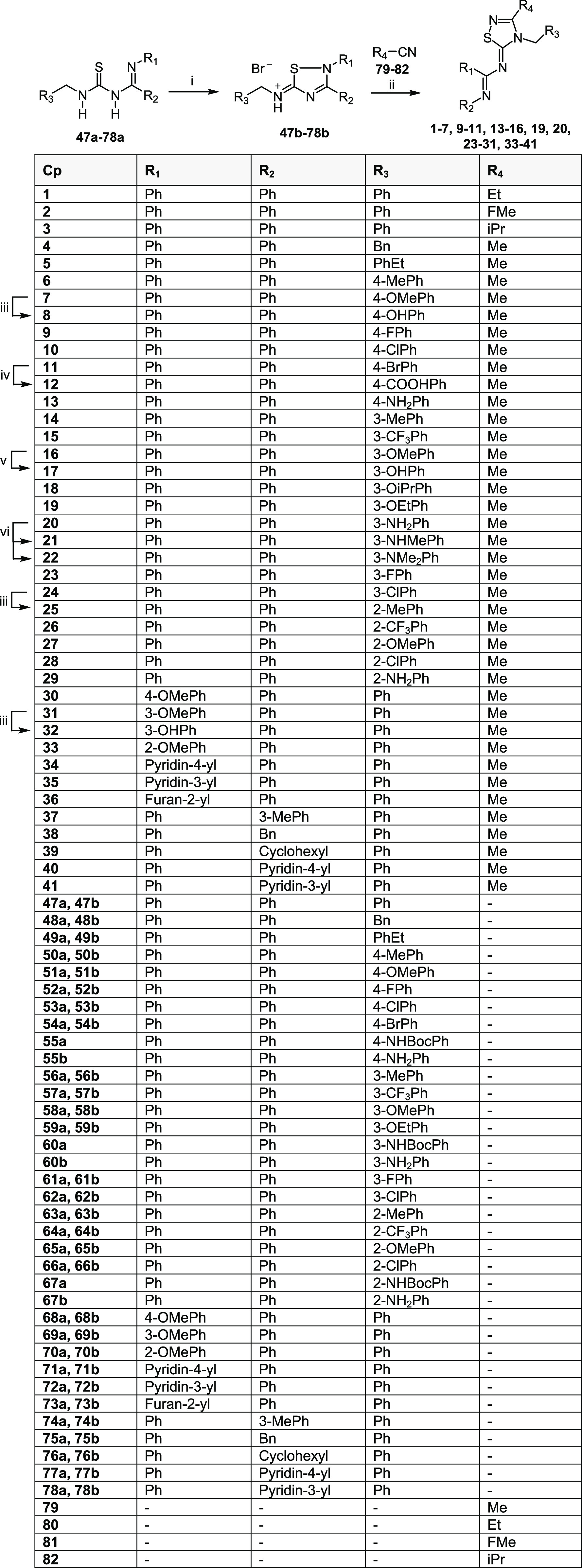
Synthesis of Final Compounds **1**–**41** Reagents and conditions: (i) Br_2_, DCM/EtOAc (1:2 v/v), 5 °C to RT; then RT, 12 h, yield 33% - quantitative; (ii) TEA, reflux 30 min, yield 13–95%; (iii) BBr_3_, DCM, 0 °C, 30 min; then RT, 12 h, yield 23–59%; (iv) *n*-BuLi, THF, −78 °C, 30 min; then CO_2_, −78 °C to RT, yield 26%; (v) K_2_CO_3_, DMF, RT 1 h; then isopropyl bromide, 70 C°, 3 h, yield 18%; (vi) MeI, K_2_CO_3_, DMF, 50 °C, 24 h, yield 22–59%.

Scheme 1 illustrates the common synthetic strategy for achieving the final desired compounds **1**–**41**. The key intermediate imidoylthioureas **47a**–**78a** underwent intramolecular cyclization by bromine oxidation, yielding thiadiazolium salts **47b**–**78b**. Following, the hydrobromide salts were treated with the appropriate nitriles **79**–**82** under basic conditions (trimethylamine) to afford the 1,2,4-thiadiazolylidene final compounds **1**–**7**, **9**–**11**, **13**–**16**, **19**, **20**, **23**–**31**, and **33**–**41**. Compounds **8**, **17**, and **32** were obtained by demethylation of the corresponding ether derivatives **7**, **16**, and **31** with BBr_3_. The carboxylic acid **12** was smoothly obtained by treatment of the bromo derivative **11** with *n*-buthyllithyum followed by the reaction with carbon dioxide. The isopropoxy derivative **18** was afforded by alkylation of the hydroxy derivative **17** with isopropyl bromide in the presence of K_2_CO_3_. Finally, alkylation of the primary amino group of derivative **20** with iodomethane under basic conditions gave the monomethylated and dimethylated derivatives **21** and **22**.

**Figure 3:**
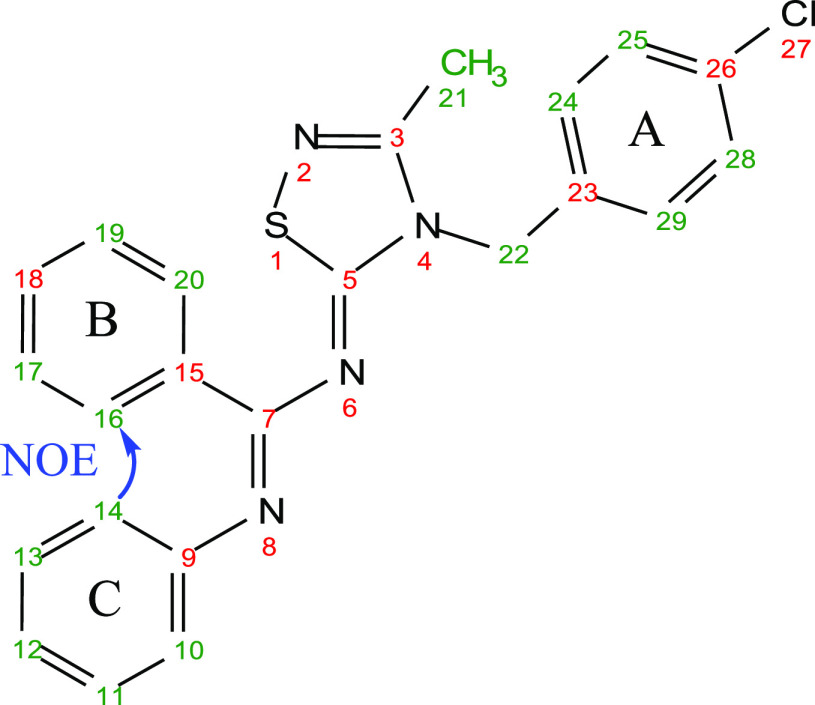
Key NOE effect of compound **10**.

The *E*- and *Z*-configurations of the two imines of the newly synthetized compounds **1**–**41** were established through selective 1D Nuclear Overhauser Effect (NOE) experiments with compound **10** as a representative example (Figure S2). Irradiation of H-22 (δ_H_ 5.54 ppm) resulted in obvious enhancement of H-24,29 (δ_H_ 7.35 ppm) of the ring A and H-21 (δ_H_ 2.41 ppm) of the methyl group. Instead, no enhancement of protons of rings B or C were detectable, ascertaining that the benzyl group A and the *N*-phenylbenzamidine portion are opposite oriented and indicating that the geometry of the double bond N_6_=C_5_ is *Z*. Meanwhile, irradiation of H-14,10 (δ_H_ 6.75 ppm) of phenyl C resulted in predictable enhancement of H-13,11 (δ_H_ 7.23 ppm) and H-12 (δ_H_ 7.02 ppm) of the same ring. Notably, selective irradiation of H-14,10 caused strong enhancement of H-16,20 (δ_H_ 7.41 ppm) of phenyl B (Figure 3), indicating that the two rings are *cis* oriented and confirming the *E* geometry of the double bond N_8_=C_7_.

**Scheme 2:**
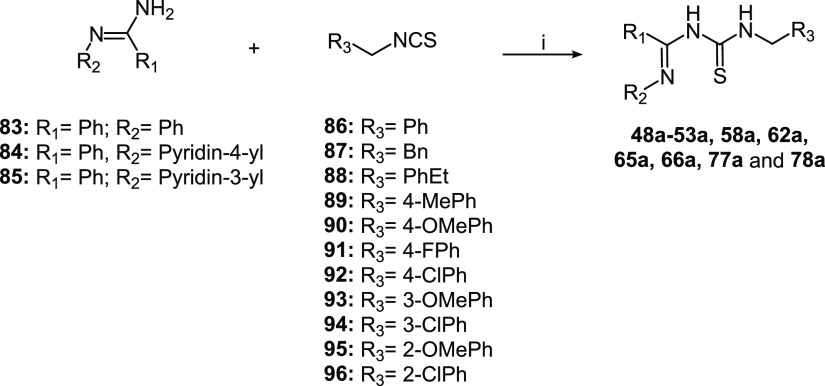
Synthesis of the Intermediate Imidoylthioureas **48a-53a**, **58a**, **62a**, **65a**, **66a**, **77a**, and **78a** Reagents and conditions: (i) dry DCE, 55 °C, 22 h, yield 14–51%.

The imidoylthioureas **47a**–**78a** necessary for final compound synthesis were prepared following two different strategies: **48a**–**53a**, **58a**, **62a**, **65a**, **66a**, **77a**, and **78a** were obtained starting from the *N*-arylbenzamidines **83**–**85** that were reacted with substituted isothiocyanates **86**–**96** to form the desired imidoylthioureas (Scheme 2). The not commercially available *N*-arylbenzamidines **84** and **85** and isocyanates **89**–**96** were obtained following standard procedures as reported in the Supporting Information (Schemes S1 and S2).

**Scheme 3:**
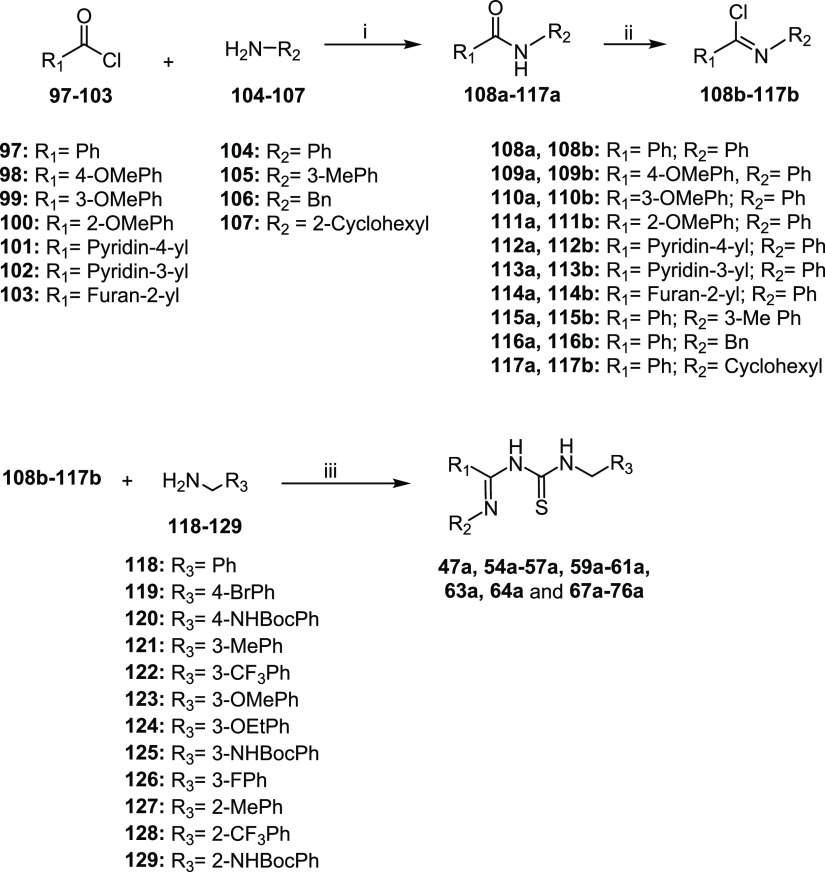
Synthesis of the Intermediate Imidoylthioureas **47a**, **54a**–**57a**, **59a**–**61a**, **63a**, **64a**, and **67a**–**76a** Reagents and conditions: (i) TEA, THF, 0 °C, 2-5 h, yield 85% - quantitative; (ii) treatment of compounds **108a**–**110a**, **112a**–**117a**: SOCl_2_, 70 °C, 2–2.5 h, yield 76–98%; treatment of compound **111a**: PCl_5_, dry toluene, reflux, 4 h, yield 81%; (iii) NaSCN, dry acetone, −15 °C to 0 °C; then benzylamines **118**–**129**, dry acetone, 0 °C to RT, yield 22–99%.

Unluckily in some cases, this strategy afforded inseparable byproducts that affected imidoylthioureas’ purification processes and reactions’ yields. Therefore, the imidoylthioureas **47a**, **54a**–**57a**, **59a**–**61a**, **63a**, **64a**, and **67a**–**76a** were synthesized by an alternative procedure as described in Scheme 3. The aromatic or heteroaromatic acyl chlorides **97**–**103** were reacted with the appropriate amines **104**–**107** to obtain the corresponding amides **108a**–**117a**. The latter were converted into imidoyl chlorides **108b**–**117b** through treatment with thionyl chloride or phosphorus pentachloride. Substitution of the chlorine atom by sodium thiocyanate followed by addition of the appropriate amines **118**–**129** afforded the desired thioureas.

**Scheme 4:**
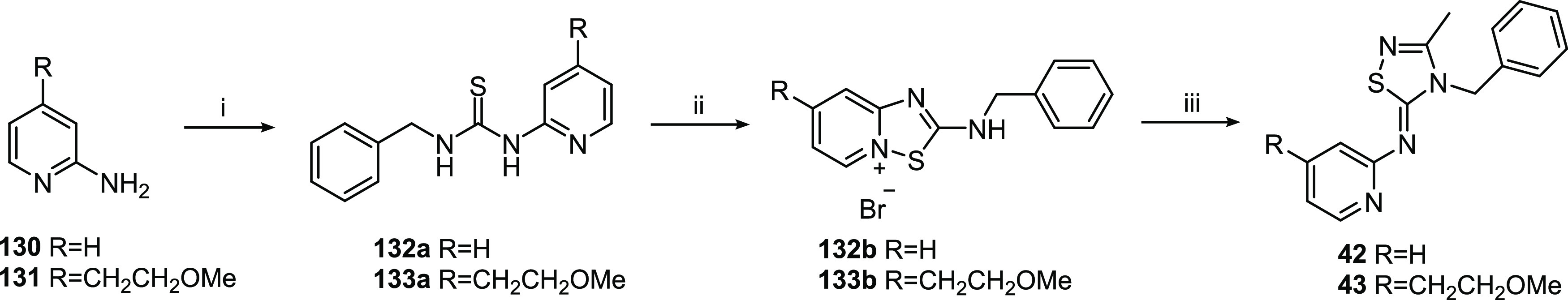
Synthesis of Final *N*-(Pyridin-2-yl)-1,2,4-thiadiazolic Compounds **42** and **43** Reagents and conditions: (i) benzyl isothiocyanate (**86**), dry DCE, 55 °C, 22 h, yield 68% - quantitative; (ii) Br_2_, DCM/EtOAc (1:2 v/v), 5 °C to RT; then RT, 12 h, yield 71–93%; (iii) ACN (**79**), DiPEA, reflux 2 h, yield 23–24%.

The 1,2,4-thiadiazolidines **42** and **43** bearing pyridylimino substituents were synthesized taking advantage of a synthetic strategy previously reported by Martinez *et al.* (Scheme 4).^45^ Reaction of appropriate pyridin-2-amines **130** and **131** with benzyl isothiocyanate **86** afforded the pyridinylthioureas **132a** and **133a**. Oxidation of **132a** and **133a** with bromine gave a regioselective ring-closure reaction, yielding the corresponding thiadiazolopyridinium bromides **132b** and **133b** in good yields. Finally, reaction of salts **132b** and **133b** in basic medium (diisopropylethylamine) with acetonitrile **79** at reflux temperature afforded the desired 5-pyridylimino 1,2,4-thiadiazolidines. The *Z*-configuration of compounds **42** and **43** was confirmed according to their ^1^H NMR spectroscopic data complemented with NOE experiments and in agreement with what was previously reported by Martinez *et al*.^45^

**Scheme 5:**
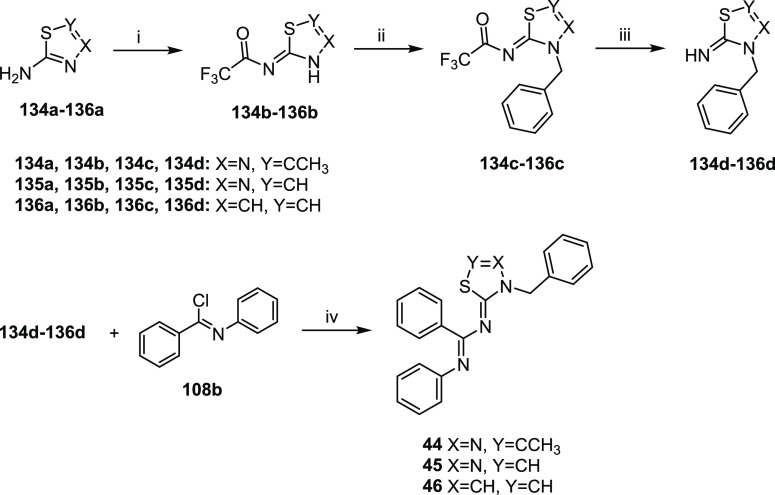
Synthesis of Final 1,3,4-Thiadiazolylidenes **44** and **45** and 1,3-Thiazolylidene **46** Reagents and conditions: (i) trifluoroacetic anhydride, toluene, 0 °C to RT; then 12 h, RT, yield 87% - quantitative; (ii) benzyl bromide (**137**), K_2_CO_3_, dry DMF, RT, 24 h, quantitative yield; (iii) NaOH 5%, THF, RT, yield 92% - quantitative; (vi) pyridine, DCM, 0 °C, 1 h; then RT, 12 h, yield 16–27%.

The 1,3,4-thiadiazolidines **44** and **45** and 1,3-thiazolidine **46** were readily synthesized through the synthetic procedure illustrated in Scheme 5, which was adapted from the synthesis reported by Nagao *et al*.^46^ Treatment of 2-amino-1,3,4-thiadiazoles **134a** and **135a** or 2-amino-1,3-thiazole **136a** with trifluoroacetic anhydride afforded the corresponding 2-trifluoroacetylamino derivatives **134b**–**136b**. Regioselective alkylation of heterocycles **134b**–**136b** with benzyl bromide **137** in the presence of K_2_CO_3_ gave the corresponding 3-benzylthiadiazoline derivatives **134c** and **135c** or 3-benzylthiazoline **136c**. After hydrolysis of the trifluoroacetyl**-**protecting group with 5% aqueous NaOH, the resulting 2-imino derivatives **134d**–**136d** were reacted with *N*-phenylbenzimidoyl chloride **108b** in the presence of pyridine to obtain the desired final compounds **44**–**46**.

**Figure 4:**
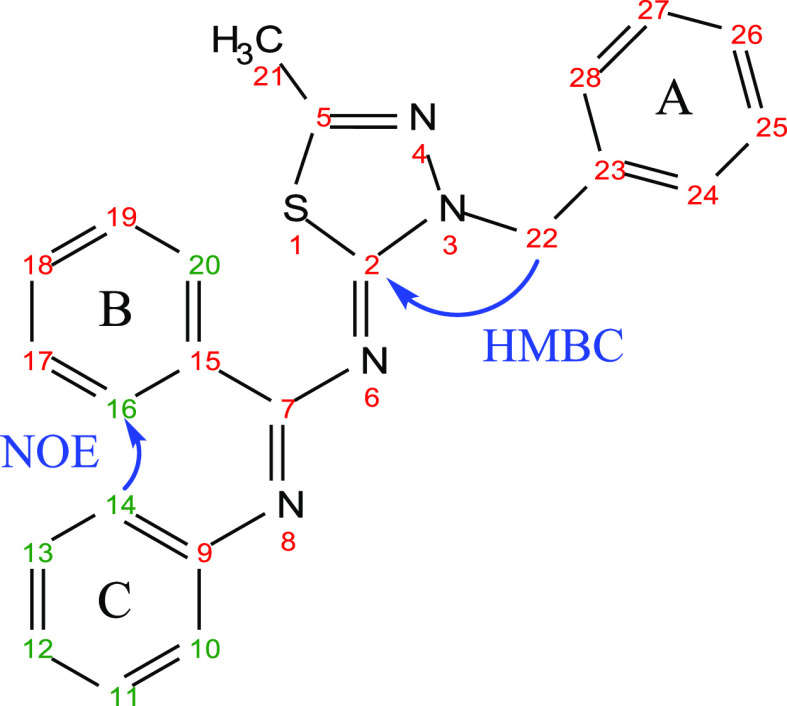
Key NOE and HMBC effects of compound **44**.

The proposed structures and isomerism of analogues **44**–**46** were confirmed by mono and two-dimensional NMR spectroscopy studies, including selective NOE (Figure S3) and Heteronuclear Multiple Bond Correlation (HMBC) experiments (Figure S4) with compound **44**, taken as a representative example. A strong correlation in the NOE spectrum was observed between the H-14,10 (δ_H_ 6.65 ppm) of phenyl C and H-16,20 (δ_H_ 7.37 ppm) of phenyl B, indicating that these protons were proximal in the *E*-configuration, as illustrated in Figure 4. Conversely, no enhancement of protons of rings B or C was detectable after irradiation of H-22 (δ_H_ 5.49 ppm), indicating a *Z* geometry of the double bond N_6_=C_2_. Furthermore, observation of the HMBC cross peak for benzylic protons H-22 with C-2 (δ_c_ 159.75 ppm), but not for H-22 with C-5 (δ_c_ 153.71 ppm), confirmed that substitution with the benzyl moiety occurred exclusively at the nitrogen N-3 of the thiadiazole (Figure 4).

### 2.3 Biological Evaluation

**Figure 5:**
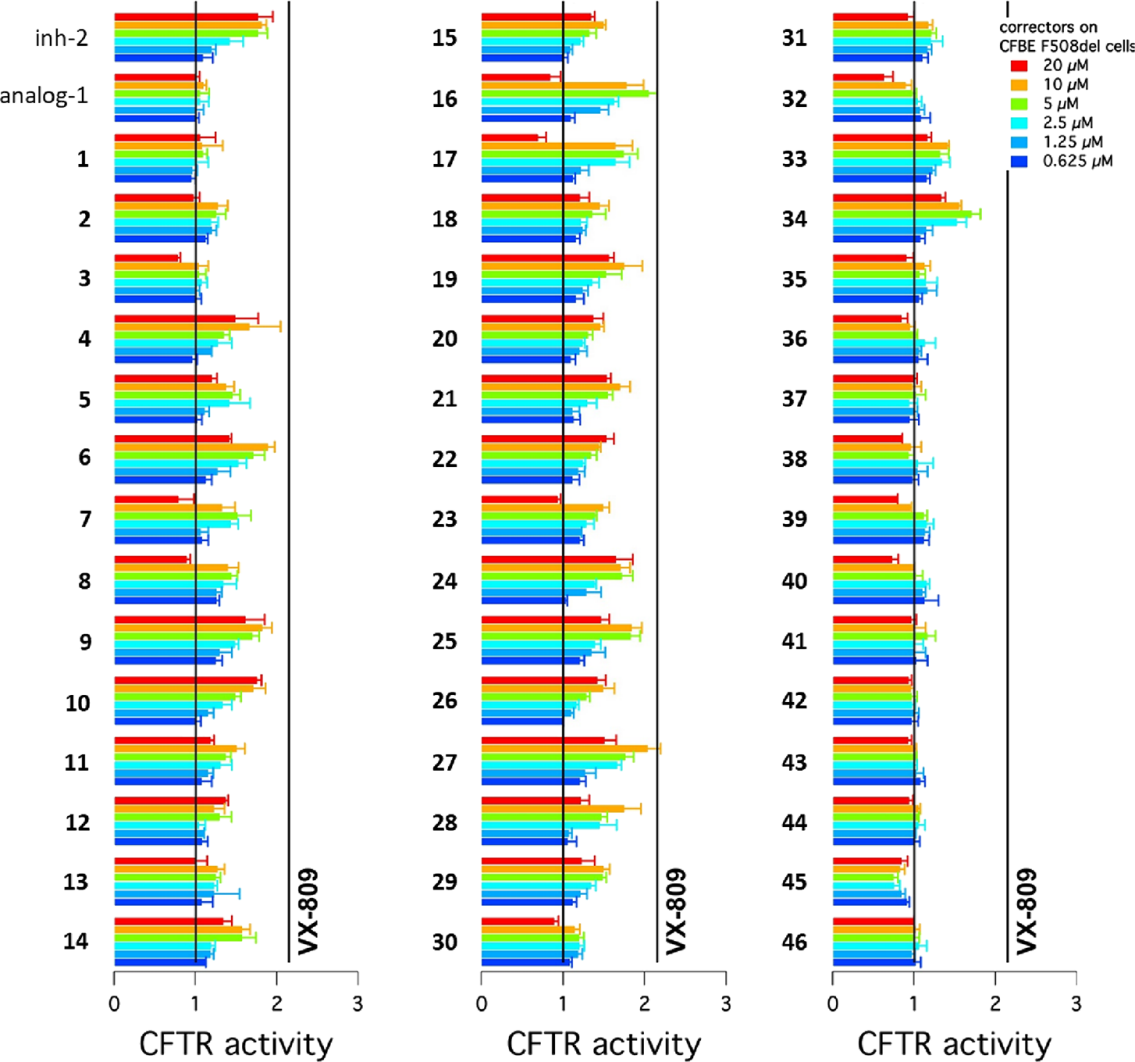
Bar graph showing CFTR activity in CFBE41o^–^ cells following 24 h treatment with vehicle alone or with analogs of **inh-2** at the indicated concentrations. The vehicle alone (DMSO) and corrector VX-809 (1 μM) were used as negative and positive controls, respectively.

As a primary screen, the new thiadiazole derivatives **1**–**46** (Table 1) were tested for their ability to rescue the F508del-CFTR trafficking defect in CFBE41o^–^ cells, stably co-expressing F508del-CFTR and the HS-YFP (Figure 5). This cell line has been extensively used by our group, in combination with the microfluorimetric assay based on the HS-YFP, to identify and characterize many CFTR correctors.^39,47^ This assay relies on the hypothesis that RNF5 inhibition results in increased mutant CFTR processing and activity. Therefore, it can easily identify putative RNF5 inhibitors, but not other types of RNF5-binding compounds that could have no effect or have a negative effect on CFTR function, such as RNF5 activators (see **analog-1**). Thus, we opted for this assay as our primary aim was the identification of RNF5 inhibitors as a possible therapeutic strategy in CF.

The substitution of the methyl group of the thiadiazoline core with larger lipophilic moieties as in compounds **1**–**3** (i.e., ethyl, fluoromethyl, and isopropyl, respectively) was not tolerated, suggesting that a methyl group best fits into the small lipophilic niche in the target pocket. Concerning the alkyl chain connecting phenyl ring A to the central core, the proper length of the tether emerged to be a methylene, as compounds **4** and **5**, bearing, respectively, ethylene and propylene linkers, were devoid of significant F508del-CFTR corrector activity. This speculation was further corroborated by the lack of CFTR corrector activity previously shown by **analog-1**, in which the phenyl ring A is directly connected to the thiadiazoline core.^40^

Next, we examined the effect of different substituents on the benzyl group A, which showed to be allocated in a large lipophilic pocket of RNF5 protein. In the *para*-position, different lipophilic substituents such as a methyl group (**6**), fluoride (**9**), chloride (**10**), and bromide (**11**) atoms maintained the activity unaltered. Meanwhile, polar, EWG, and EDG substituents such as hydroxyl (**8**), amino (**13**), carboxyl (**12**), and methoxyl (**7**) groups resulted in a reduced potency. Regarding the *meta*- substituted series, derivatives **16**, **17**, and **24** carrying a methoxyl group, hydroxyl group, and a chloride atom, respectively, proved to have increased activity, while **15**, **18**, and **20** bearing trifluoromethyl, isopropoxyl, and amino groups, respectively, presented a decreased potency. Furthermore, the *meta*-methyl (**14**), ethoxyl (**19**), methylamino (**21**), and dimethylamino (**22**) groups and *meta*-fluoride atom (**23**) did not affect the activity. Among the explored *ortho*-substituents, the methyl (**25**) and methoxyl (**27**) groups caused a slight increase in activity while the trifluoromethyl (**26**) had a detrimental effect. Finally, **28** and **29** bearing a chloride atom and an amino moiety, respectively, showed a comparable activity to the unsubstituted original hit **inh-2**. Overall, the SARs of the new series of 1,2,4-thiadiazolidines with variations on ring A were rather complex to rationalize, and no clear pattern could be identified. However, compounds **16** and **17** showed the best activities of the series, with EC_50_ equal to 1.2 and 1.6 μM, respectively (**inh-2**, EC_50_ = 2.6 μM).^40^

Moving to the *N*-phenylbenzamidine portion, addition of methoxyl and hydroxyl groups in the *ortho-*, *meta-*, and *para*-positions of phenyl ring B (**30**–**33**) or replacement of ring B with pyridine-3-yl and furan-2-yl moieties (**35** and **36**, respectively) resulted in a drop of activity. Instead, the substitution of phenyl B with a pyridine-4-yl moiety, as in compound **34**, was tolerated, maintaining the activity equal to **inh-2**. Hence, it appears that only a *para*-pyridine at this position could engage favorable hydrogen bond interactions with TRP48 of the RNF5 pocket, which may be responsible for the maintenance of the activity. The replacement of the phenyl ring C with *meta*-tolyl (**37**), benzyl (**38**), and cyclohexyl (**39**) rings or pyridines (**40** and **41**) led to inactive analogs. From the results obtained, it appears that both phenyl B and C of the *N*-phenylbenzamidine portion are necessary for π–π stacking interactions with the RNF5 pocket. This speculation was further corroborated by analogues **42** and **43**, in which the removal of both rings and their replacement with pyridin-2-yl moieties led to inactive compounds, contrary to what was expected from the docking model.

Finally, the replacement of the central 1,2,4-thiadiazolidine core of the scaffold with different five-membered heterocycles, such as 5-methyl-1,3,4-thiadiazolidine (**44**), 1,3,4-thiadiazolidine (**45**), and 1,3-thiazolidine (**46**), abolished any corrector activity of the compounds. Therefore, we concluded that the 1,2,4-thiadiazolidine ring is mandatory for optimal π–π stacking interactions with TRP48 and HIS52 of the target pocket and thus for the activity of compounds.

In parallel to the SAR campaign, binding assays were also attempted to assess the putative physical interaction of the novel inhibitors with the RNF5 target. First, the hit **inh-2**, **analog-1**, and compounds **16** and **17**, chosen as the most active of the series, were tested for their solubility and aggregation state by SPAM filter^48^ assays in PBS buffer (PBS pH 7.5, 1 μM ZnCl_2_, 1 mM DTT, 10% D_2_O, and 1% DMSO-*d*_6_) at different theoretical concentrations (20, 50, and 100 μM), using 4-(trifluoromethyl)benzenesulfonamide (200 μM) as an internal reference.

Unfortunately, the compounds showed a surprisingly poor solubility (Table 2) in spite of their four nitrogen atoms and hydroxyl group, which can be attributed to their expected near planarity.^49^ Under these circumstances, it was decided to explore the solubility of all the synthetized compounds, regardless of their biological activities, to find a suitable candidate for the validation of our working hypothesis, i.e., that the compounds exert their activities by directly binding to RNF5. For that, we first calculated the predicted octanol/water partition coefficient (log Po/w) and the predicted aqueous solubility (log *S*) of all the analogues by QikProp (Schrödinger Release 2022-1: QikProp, Schrödinger, LLC, New York, NY, 2021, Table S1). As expected, the obtained values of log Po/w > 1 suggest a hydrophobic nature of the studied compounds associated with a predicted poor aqueous solubility (Table S1).^50^ To shrink down the number of compounds for solubility experiments, just the ones that had −2.0 < log Po/w < 6 and −6.5 < log *S* <0.5 values were selected. Among them, the PBS solubility was determined for five representative analogues: (i) **12** and **29** bearing different substituents on the benzyl group A; (ii) **34** and **40** showing the replacement of rings B and C with pyridine-4-yl moieties; (iii) **42** in which the *N*-phenylbenzamidine portion was replaced with a pyridine-2-yl moiety (Table 1). The results reported in Table 2 highlighted that only compounds **12**, **40**, and **42** showed no aggregation and solubility suitable for in vitro/biophysical binding experiments.

**Table 2:** Computed Octanol/Water Log *P* Values, Log *S* Values (*S* in mol·dm^–3^), and Experimental Solubility (in μM) of Selected Compounds in PBS pH 7.5, 1 μM ZnCl_2_, 1 mM DTT, 10% D_2_O, and 1% DMSO-*d*_6_

			solubility and aggregation in PBS buffer by NMR			
compound	Plog Po/w	Plog *S*	20 μM	50 μM	100 μM	aggregation
**inh-2**	6.38	–6.56	<5	<5	<5	no
**analog-1**	6.096	–6.695	<5	<5	<5	no
**16**	6.349	–6.196	<5	<5	<5	no
**17**	5.688	-6.447	<5	<5	<5	no
**12**	5.677	-6.432	20	50	100	no
**29**	5.569	-6.371	<5	<5	<5	no
**34**	5.345	–5.741	10	10	10	no
**40**	5.342	–5.7	20	30	30	no
**42**	4.043	–4.279	20	40	5	no

**Figure 6:**
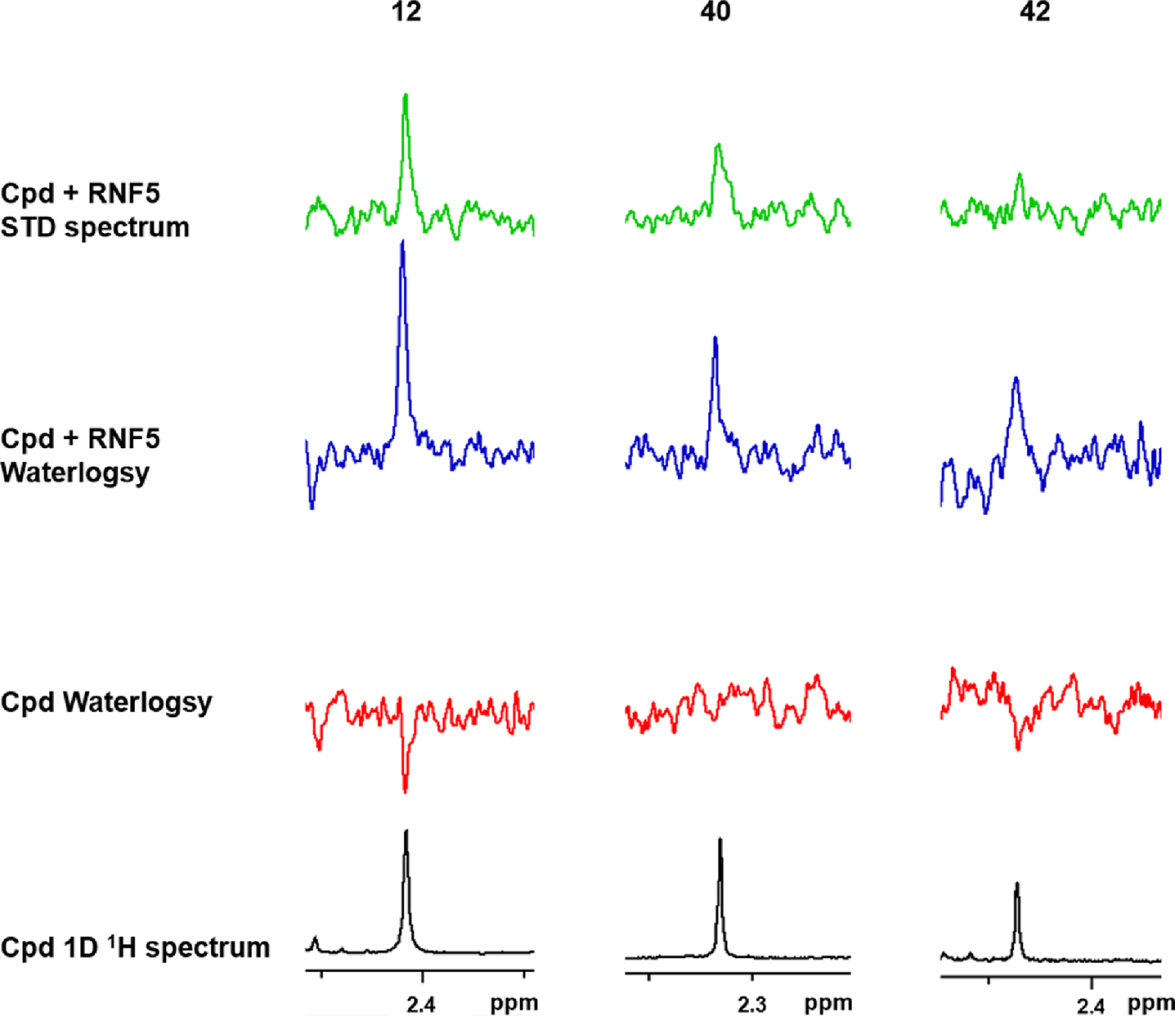
^1^H NMR methyl signal of compounds **12** (left), **40** (middle), and **42** (right):1D spectrum (black), WaterLOGSY spectrum (red); WaterLOGSY spectrum in the presence of RNF5 (blue) and STD spectrum (green). The change, upon protein addition, of compounds NMR signals from negative to positive in WaterLOGSY spectra and the presence of compounds NMR signals in STD spectra indicate compounds binding to RNF5.

The bindings of compounds **12**, **40**, and **42** were initially evaluated by MicroScale Thermophoresis (MST) using a purified recombinant truncated form of RNF5 (aa 1–117), lacking the C-terminal transmembrane domains. The protein was covalently labeled with a red dye (NHS) on the primary amines (lysine residues). Although a complete affinity curve could not be built, evidence of direct binding (binding check tests) to the protein was observed only for compound **12** (see Figure S5). ^1^H Water-Ligand Observed via Gradient SpectroscopY (WaterLOGSY)^51^ and Saturation-Transfer Difference (STD)^52^ NMR experiments on the recombinant RNF5 form were further performed to test the direct binding of the three compounds to the protein with a more sensitive, label-free, independent approach. Analogs **12**, **40**, and **42** were tested at 50 μM in the absence and in the presence of 3 μM RNF5 (1–117) protein. As reported in Figure 6, the three compounds bind the recombinant RNF5 protein. Indeed, for all three compounds, the NMR signals of their methyls are present in the STD spectra, and in the WaterLOGSY spectra, their signals change from negative to positive in the presence of the protein, indisputably highlighting the formation of compound–protein binding events. Despite the lower F508del-CFTR activity compared to **inh-2**, compound **12** clearly binds RNF5 protein pointing at this protein as the target responsible for the observed in cell activity. Also compounds **40** and **42**, although almost inactive as CFTR correctors, showed to interact with RNF5. It has to be noted that NMR is a very sensitive technique that allows detecting also compounds weakly binding to their target protein independent of the possible downstream biological effects. Indeed, we can speculate that compounds **40** and **42** may bind to RNF5 without inhibiting its ubiquitin ligase activity, thus not leading to mutant CFTR rescue. These compounds could even act as RNF5 activators, similar to **analog-1**. In conclusion, our data clearly confirm the ability of the synthesized compounds to directly bind RNF5, their putative target.

Even though the poor solubility demonstrated by most of the 1,2,4-thiadiazolylidene derivatives prevented us from performing NMR binding experiments on all the synthetized analogues, the SAR campaign allowed us to identify several compounds with promising corrector activity profiles, such as compounds **6**, **9**–**11**, **14**, **16**, **17**, **19**, **21**–**25**, **27**–**29**, and **34** that were selected for further cell-based studies.

**Figure 7:**
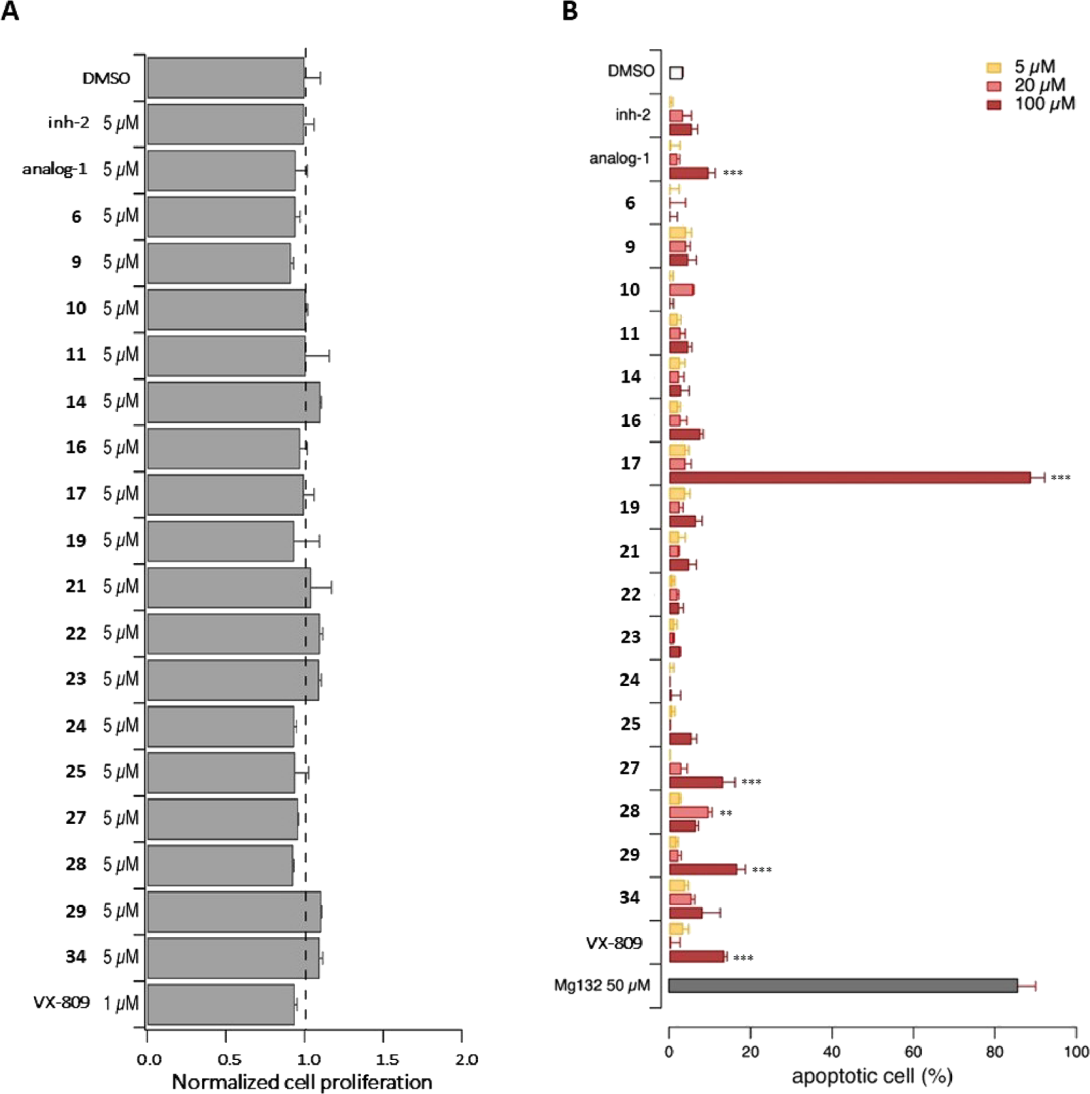
Evaluation of the effect of putative RNF5 inhibitors on cell proliferation and apoptosis. (A) The bar graph shows the number of viable CFBE41o^–^ cells following 48 h treatment with test compounds at 5 μM. (B) Bar graph shows the number of apoptotic CFBE41o^–^ cells following 24 h treatment with test compounds at the indicated concentrations. Data are expressed as means ± SEM, *n* = 4–6. Asterisks indicate statistical significance: ***p < 0.001, **p < 0.01.

We performed a preliminary evaluation of the effect of chronic treatment with selected analogues on the proliferation and apoptosis of CFBE41o^–^ cells (Figure 7). Indeed, RNF5 can modulate cell motility and proliferation by regulating paxillin ubiquitylation and thus its degradation.^53^ For the proliferation assay, CFBE41o^–^ cells stably co-expressing F508del-CFTR and the HS-YFP were treated with test compounds (5 μM), VX-809 (1 μM), or vehicle alone (DMSO) for 48 h. Cell proliferation was then monitored by measuring the area covered by the cells based on the YFP signal. For the apoptosis assay, 24 h after plating, CFBE41o^–^ cells stably expressing F508del-CFTR were treated with test compounds (5, 20, and 100 μM), VX-809 (1 μM), MG132 (50 μM), or vehicle alone (DMSO) for 24 h. Cell nuclei were then counterstained with Hoechst 33342 and propidium iodide to visualize the total and apoptotic cell count, respectively, and imaged by using an Opera Phenix high-content screening system. None of the test compounds significantly altered cell proliferation (Figure 7A). However, compounds **17** and **27**–**29** markedly increased cell apoptosis, although only at a very high concentration (100 μM; Figure 7B). Notably, similar effects were observed upon treatment with VX-809 and **analog-1** at the same concentration.

**Figure 8:**
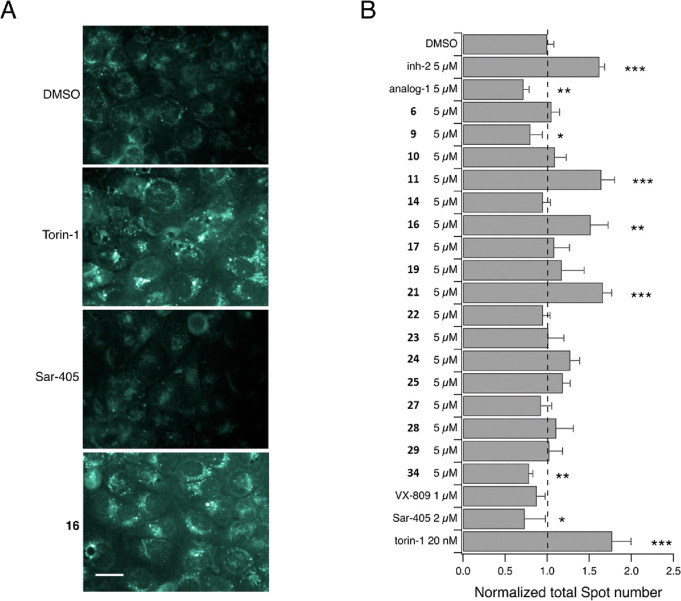
Evaluation of effects of putative RNF5 inhibitors on ATG4B-mediated basal autophagy. (A) Representative confocal microscopy images of F508del-CFTR expressing CFBE41o^–^ cells treated with the indicated compounds and loaded with MDC. Scale bar = 50 μm. (B) The bar graph shows the quantification of the number of spots (resembling autophagic vesicles) in cells treated with the indicated compounds, normalized for the control condition. Data are expressed as means ± SEM, *n* = 4–6. Asterisks indicate statistical significance: ***p < 0.001, **p < 0.01, *p < 0.05.

To further support our hypothesis that CFTR rescue following treatment with test compounds was indeed due to RNF5 inhibition, we considered additional biological processes known to be regulated by RNF5 ligase activity. To this aim, we focused our attention on ATG4B, a known regulator of basal autophagy, whose degradation is mediated by RNF5 activity.^54^*In vivo* ubiquitination experiments previously confirmed that **inh-2** inhibits ATG4B degradation, thus increasing the basal level of autophagy.^40^ Therefore, induction of autophagy can be considered as additional evidence demonstrating the ability of test compounds to inhibit RNF5. We thus evaluated the effect of **inh-2** analogues on induction of the autophagy pathway. To this aim, we monitored the formation of autophagic vacuoles in F508del-CFTR-expressing CFBE41o^–^ cells (Figure 8) by using the autolysosome marker monodansylcadaverine (MDC). MDC accumulates inside autophagosomes. After fusion of autophagosomes with lysosomes, MDC fluorescence increases due to the acidic environment.^55^ Therefore, F508del-CFTR-expressing CFBE41o^–^ cells were treated for 24 h with test compounds (5 μM), VX-809 (1 μM), or DMSO alone (vehicle). In the last 3 h of incubation, we added torin-1 (20 nM), a known autophagy inducer, and SAR-405 (2 μM), a potent inhibitor of the autophagic pathway. The cells were then loaded with MDC and evaluated by high-content confocal imaging to detect signal spots (corresponding to autophagic vacuoles) in each cell. Determination of signal spots clearly demonstrated that the number of autophagic vacuoles was significantly increased after treatment with torin-1, **inh-2**, or compounds **11**, **16**, and **21** (Figure 8). In contrast, incubation with SAR-405, **analog-1**, **9**, and **34** significantly decreased the number of autophagic vacuoles (Figure 8).

From the abovementioned investigations, we could discard compounds exhibiting (i) lower or no CFTR corrector activity compared to **inh-2** (**1**–**5**, **7**, **8**, **12**, **13**, **15**, **18**, **20**, **26**, **30**–**33**, and **35**–**46**), (ii) cytotoxic effects (**17** and **27**–**29**), and (iii) discrepancy between data obtained from MDC signal spot evaluation and the HS-YFP assay (**6**, **9**, **10**, **14**, **17**, **19**, **22**–**25**, **27**–**29**, and **34**). Therefore, **11**, **16**, and **21** resulted the most promising compounds of the library, and, among them, analogue **16** caused both a strong activation of basal autophagy and a greater F508del-CFTR rescue than **inh-2**.

**Figure 9:**
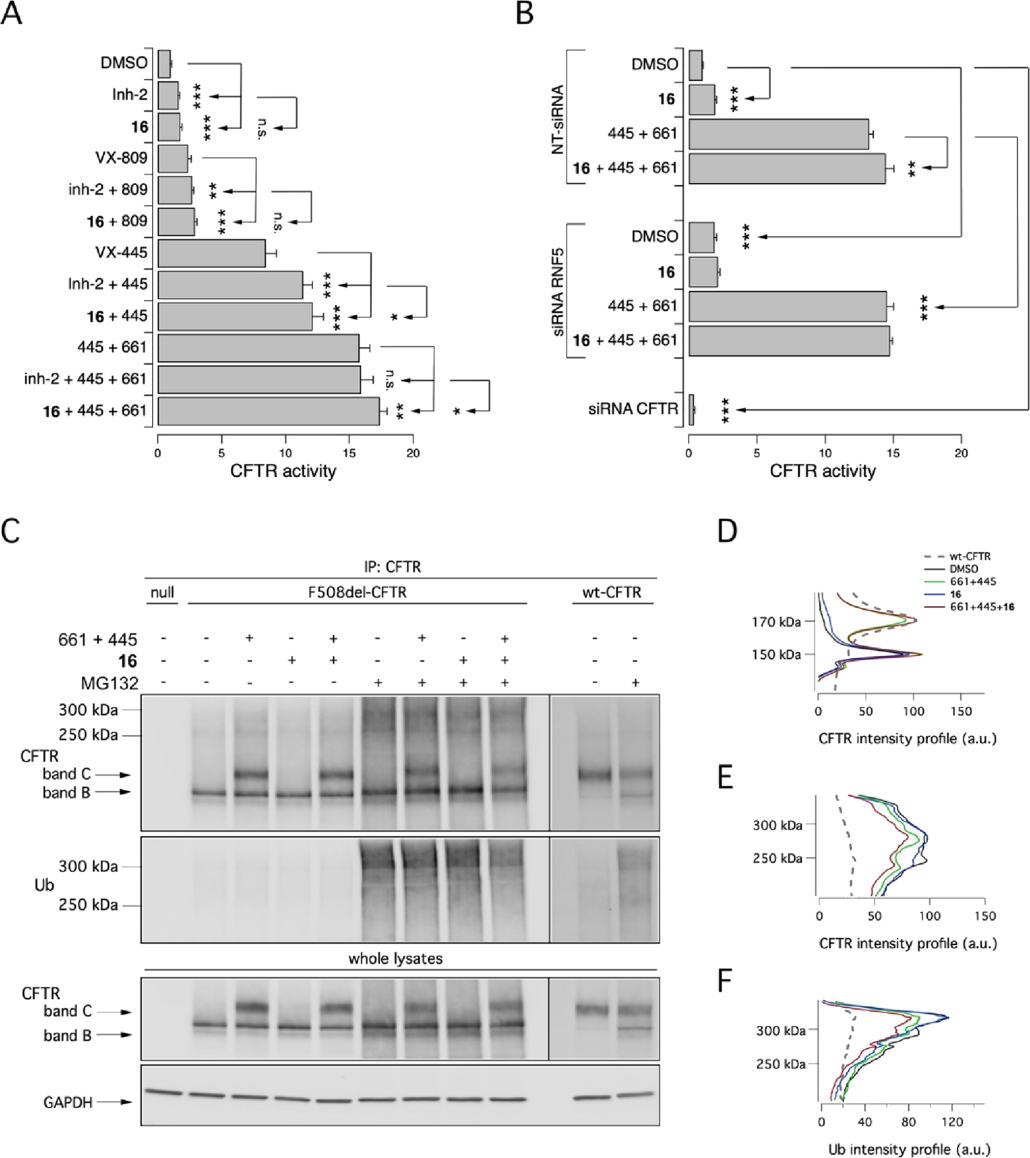
Putative RNF5 inhibitors improve mutant CFTR rescue by approved correctors by decreasing CFTR ubiquitylation. (A) Bar graph showing CFTR activity in CFBE41o^–^ cells following 24 h treatment with vehicle alone or with **inh-2** (5 μM) and its analogue **16** (5 μM) as single agents or combined with correctors VX-809 (3 μM), VX-445 (3 μM), or VX-661 + VX-445 (10 μM + 3 μM). (B) Bar graph showing CFTR activity in CFBE41o^–^ cells transfected with NT or RNF5 siRNA and treated for 24 h with DMSO, or analogue **16** (5 μM), or VX-661 + VX-445 (10 μM + 3 μM), or their combination. The effect of CFTR siRNA is shown as additional control of transfection efficiency. Asterisks indicate statistical significance: ***p < 0.001, **p < 0.01, *p < 0.05, while n.s. indicates “not significant”. (C) Biochemical analysis of CFTR ubiquitylation and expression pattern in CFTR immunoprecipitates from CFBE41o^–^ cells after 24 h treatment with analogue **16** (5 μM), VX-661 + VX-445 (10 μM + 3 μM), and their combination in the absence or in the presence of MG-132 (10 μM; last 4 h) to block proteasomal degradation. Images for CFTR and Ub blots of F508del- and wt-CFTR samples are different exposures of the same membranes. (D–F) Analysis of intensity profiles of CFTR and ubiquitin (D) in the absence or (E and F) in the presence of MG-132.

To further characterize the ability of RNF5 inhibitors to improve F508del rescue, we compared the efficacy of **inh-2** and analogue **16** in combination with approved correctors. Both RNF5 inhibitors significantly increased mutant CFTR activity upon co-treatment with VX-809 or VX-445 (Figure 9A). However, only analogue **16** was able to further improve the rescue elicited by the double combination VX-661 + VX-445 (Figure 9A).

We then aimed to indirectly confirm that the rescuing activity of analogue **16** was indeed due to RNF5 inhibition. We therefore tested the compound in F508del-CFTR-expressing CFBE41o^–^ cells transfected with a non-targeting (NT) siRNA or an siRNA molecule targeting RNF5. We reasoned that the presence of an additive effect between treatment with analogue **16** and RNF5 silencing would have disproved the mechanism of action of analogue **16** (i.e., RNF5 inhibition). Interestingly, the extent of F508del-CFTR rescue was similar in **16**-treated cells transfected with NT siRNA and in DMSO-treated cells transfected with RNF5 siRNA (Figure 9B). In addition, treatment with analogue **16** alone or combined with VX-661 + VX-445 increased the F508del-CFTR activity only in cells transfected with NT siRNA, but not in those transfected with RNF5 siRNA (Figure 9B).

Finally, we evaluated ubiquitylation of mutant CFTR in CFBE41o^–^ cells following 24 h treatment with DMSO (vehicle), analogue **16**, VX-661 + VX-445, and their combination. Subsequently, cells were treated for 4 h with DMSO alone or with MG-132 (10 μM; to block proteasomal degradation) and then lysed. Cell lysates were immunoprecipitated using an anti-CFTR antibody and then subjected to SDS-PAGE followed by Western blotting to evaluate CFTR expression and ubiquitylation status (Figure 9C–F). As previously reported, VX-661 + VX-445 caused a marked rescue of mutant CFTR, as shown by the appearance of the mature form of CFTR (band C; Figure 9C, CFTR blot) and evidenced also by the analysis of intensity profiles (Figure 9D), while the effect of analogue **16** was very modest (Figure 9C,D). Treatment with MG-132 caused the appearance of CFTR forms at high molecular weight (at 200–350 kDa, resembling ubiquitylated CFTR proteins) that decreased in the presence of test compounds, as evidenced by the analysis of intensity profiles (Figure 9E). MG-132 caused the accumulation of ubiquitylated CFTR, in particular under control (DMSO) conditions (Figure 9F, Ub blot), and markedly decreased upon treatment with VX-661 + VX-445, and, to a further extent, upon treatment with VX-661 + VX-445 plus the RNF5 inhibitor (Figure 9C,F). These data clearly demonstrate that the combination of approved drugs and an optimized RNF5 inhibitor can additively decrease ubiquitylation of mutant CFTR in immortalized bronchial cells.

**Figure 10:**
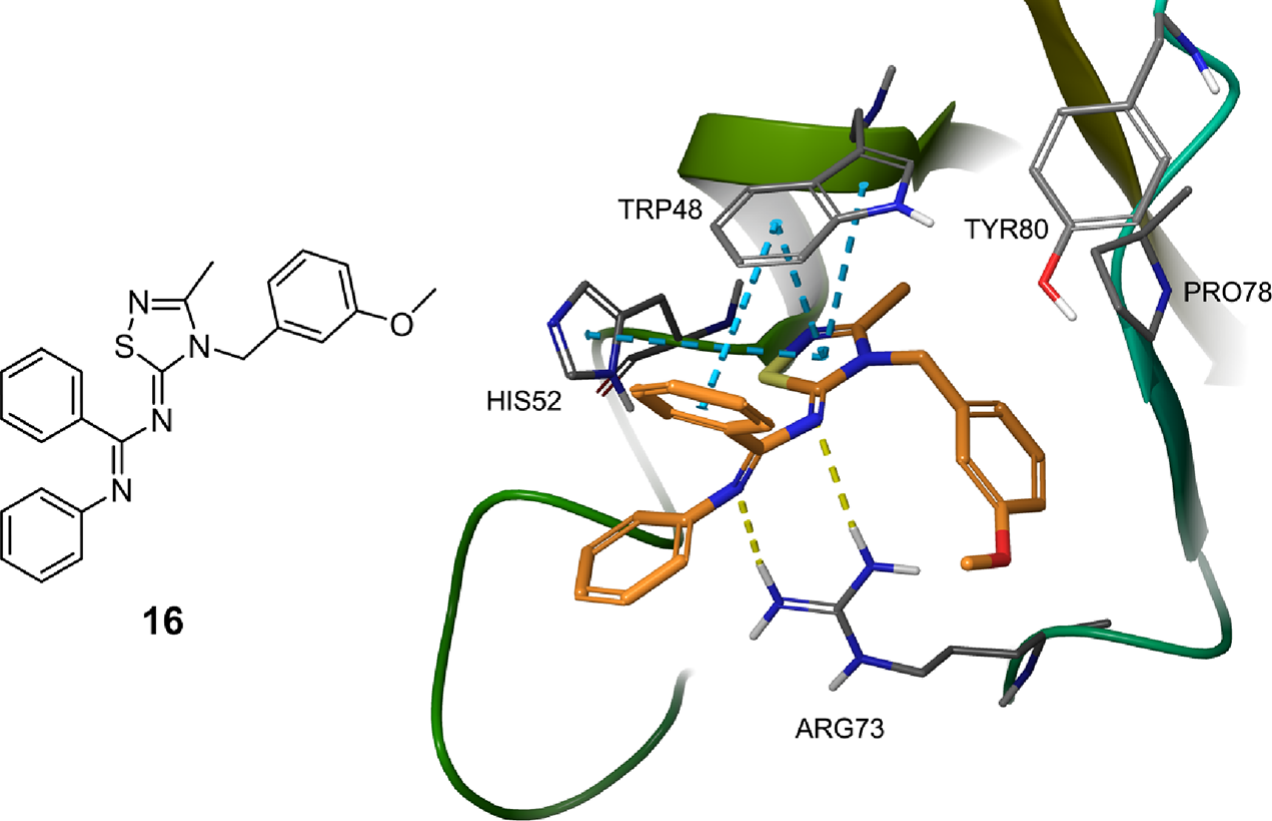
Proposed binding mode of analogue **16** into the RNF5 pocket. Yellow dashed lines indicate the hydrogen bonds, while blue dashed lines indicate the π–π stacking interactions.

Taken together, these findings suggest that **16** may represent the strongest RNF5 inhibitor of the series of tested compounds. The proposed binding mode based on docking simulations of compound **16** into the RNF5 homology model (Figure 10) suggests that **16** engages the same π–π and H-bond interactions as **inh-2**, but it might be hypothesized that the addition of a *meta-*methoxide group on the benzyl ring of the 1,2,4-thiadiazolylidene scaffold induces further favorable hydrophobic interactions with VAL38 and VAL76 (not shown in the figure) of the target pocket. Hence, these additional interactions may be responsible for the greater F508del-CFTR rescue activity elicited by compound **16** with respect to the parent compound **inh-2**. A comparison of the **16** and **inh-2** binding modes to RNF5 is shown in Figure S6.

## 3 Conclusions

In CF, the most frequent autosomal recessive disease, the deletion of F508 in the CFTR anion channel is associated to misfolding and defective gating of the mutant protein. Among the known proteins involved in CFTR processing, one of the most promising drug targets is the ubiquitin ligase RNF5, which normally promotes F508del-CFTR degradation. In this context, a small molecule RNF5 inhibitor is expected to chemically mimic a condition of RNF5 silencing, thus preventing mutant CFTR degradation and causing its stabilization and plasma membrane trafficking. Hence, by exploiting a virtual screening (VS) campaign, the hit compound **inh-2** was discovered as the first-in-class inhibitor of RNF5. Evaluation of **inh-2** efficacy on CFTR rescue showed that it efficiently decreases ubiquitination of mutant CFTR and increases chloride current in human primary bronchial epithelia. More recently, another study aimed to identify compounds able to inhibit dislocation of misfolded proteins from the endoplasmic reticulum (ER) lumen to the cytosol in ER-associated degradation. This study led to the discovery of FX12 as an RNF5 E3 inhibitor and degrader that binds directly to RNF5 and inhibit its ligase activity *in vitro*.^44^ Consistent with this activity, and as reported for **inh-2**, FX12 decreases mutant CFTR ubiquitylation therefore rescuing CFTR channel activity, also enhancing the effect of the FDA-approved drugs VX809 and VX661. Finally, similar to **inh-2**, FX12 also modulates paxillin expression. However, FX12 does not improve mutant CFTR channel activity in human primary bronchial epithelia.^44^ A possible reason for this is that, while **inh-2** is a RNF5 inhibitor, FX12 is not only an inhibitor but also a degrader of RNF5. This difference might impact mutant CFTR biogenesis, stability, and/or activity by affecting multiple pathways.

With the aim of gaining a better insight into the SAR of **inh-2**, a large library of analogs of the original hit compound was designed and synthetized. The optimization of general and versatile synthetic routes gave access to a series of novel 1,2,4-thiadiazolylidene-based compounds, which were subjected to biological activity evaluations as F508del-CFTR correctors. SAR efforts ultimately led to compound **16** that elicited a greater F508del-CFTR rescue than **inh-2** in the HS-YFP functional assay. Evaluation of the effect of **16** on cell proliferation and apoptosis showed good tolerability and no toxic side effects of the putative ubiquitin ligase inhibitor. Interestingly, analogue **16** showed also an additive effect upon co-treatment with the highly effective triple combination elexacaftor/tezacaftor/ivacaftor, resulting in a decreased mutant CFTR ubiquitylation paralleled by an increased CFTR function. These results are particularly encouraging as small molecule ligase inhibitors could also act on ligases other than RNF5, leading to cytotoxic effects. On the contrary, RNF5 genetic suppression has no apparent negative effects *in vitro* and *in vivo*,^39^ and therefore putative RNF5 inhibitors are expected to lead to few side effects*.* The mechanism of action of analogue **16** was further investigated by exploiting known cellular targets of RNF5, such as the regulator of basal autophagy ATG4B. Functional evidence demonstrated that **16** strongly increases the basal level of autophagy of F508del-CFTR-expressing CFBE41o^–^ cells, similar to the parent compound **inh-2**. Notably, there is an emerging interest for autophagy modulating compounds in controlling the pathogenesis of CF disease,^56^ and the restoration of autophagy has been proposed *per se* as a strategy to allow the rescue of F508del-CFTR trafficking.^57−61^ Furthermore, RNF5 knockout has shown to enhance autophagy-mediated clearance of bacterial infection,^54^ which is highly recommendable in CF patients having chronic lung infections. Although the induction of basal autophagy provided by **16** has to be considered as a secondary effect of RNF5 inhibition, it may have an additional positive effect on mutant CFTR rescue and innate host defense. These findings suggest that compound **16** may act as a stronger inhibitor of RNF5 ligase activity than **inh-2** by directly binding to the RNF5 RING domain, as suggested by *in silico* prediction studies. Although the poor solubility of compound **16** hampered the experimental evidence of its direct binding to RNF5, biophysical evidence of direct protein interaction has been obtained for compound **16** analogs that are more soluble. However, the extent of rescue remains lower than that obtained with VX-809. This may be due to cellular QC mechanisms, which are functionally redundant. Indeed, the modulation of one of the cellular QC elements (such as the inhibition of the ubiquitin ligase RNF5) may have a lower effect than classic correctors on the global biological outcome due to adaptive responses. Nevertheless, it has been widely described that proteostasis regulators’ effects are additive with other correctors,^37^ and therefore, their combination with current therapies is expected to have higher therapeutic ceiling and expand pharmacological treatment applicability to CF patients bearing mutations poorly responsive to already developed modulators.

Taken together, our findings suggest that the 1,2,4-thiadiazolylidene scaffold could be further exploited for the discovery of novel RNF5 inhibitors able to rescue mutant CFTRs. Therefore, structural tuning will be further implemented to increase the promising corrector activity of **16** and its pharmacokinetic profile, ultimately providing a more drug-like 1,2,4-thiadiazolylidene derivative.

## 4 Experimental Section

### 4.1 Chemistry

#### 4.1.1 General Chemical Methods

Solvents and reagents were purchased from commercial sources and used without further purification. If required, solvents were distilled prior to use. For simplicity, solvents and commonly used reagents are indicated as follows: acetonitrile (ACN), dichloromethane (DCM), 1,2-dichloroethane (DCE), diethyl ether (Et_2_O), petroleum ether (PE), dimethyl sulfoxide (DMSO), ethanol (EtOH), ethyl acetate (EtOAc), methanol (MeOH), *N*,*N*-dimethylformamide (DMF), tetrahydrofuran (THF), triethylamine (TEA), *N*,*N*-diisopropylethylamine (DIPEA), and 1,1′-thiocarbonyldiimidazole (TCDI). When stated, reactions were performed in an inert atmosphere. Reaction progress was monitored by thin layer chromatography (TLC) analyses on pre-coated silica gel plates (Kieselgel 60F_254_, Merck) and detected with UV light (254 nm) and/or KMnO_4_ stain. Flash column chromatography was carried out using a silica gel (particle size 40–63 μM, Merck) with the indicated solvent system as an eluent. NMR experiments were run on a Varian Gemini 401 MHz spectrometer (401.13 MHz for ^1^H and 100.62 MHz for ^13^C), equipped with a BBI probe and Z-gradients. Spectra were acquired at 300 K, using deuterated dimethyl sulfoxide (DMSO-*d*_6_) or deuterated chloroform (chloroform-*d*) as solvents. Chemical shifts (δ) for ^1^H and ^13^C spectra were recorded in parts per million (ppm) using the residual non-deuterated solvent as the internal standard (for chloroform-*d*, ^1^H 7.26 ppm; for DMSO-*d*_6_, ^1^H 2.50 ppm, ^13^C 39.52 ppm). Multiplicities are indicated using the following abbreviations: bs, broad signal; s, singlet; d, doublet; t, triplet; q, quartet; m, multiplet. The 2D experiments were acquired as follows: ^1^H-^1^H COSY (2 transients, 256 increments), ^1^H-^13^C HSQC (4 transients, 256 increments), ^1^H-^13^C HMBC (8 transients, 512 increments), and 2D NOESY (16 transients, 256 increments). The 1D NOESY experiment was performed with NOE DPFGSE pulse sequence at a mixing time of 2.5 s. UPLC/MS analyses of all the final compounds were run on a Waters ACQUITY UPLC-MS system consisting of an SQD (Single Quadrupole Detector) mass spectrometer equipped with an electrospray ionization (ESI) interface and a photodiode array detector (PDA). The PDA range was 210–401 nm. ESI in positive and negative mode was applied in the mass scan range 100–500 Da. Analyses were performed on an ACQUITY UPLC BEH C_18_ column (50 mm × 2.1 mm i.d., particle size 1.7 μm) with a VanGuard BEH C_18_ pre-column (5 mm × 2.1 mm i.d., particle size 1.7 μm) (log *D* > 1). The mobile phase was 10 mM NH_4_OAc in H_2_O at pH 5 adjusted with AcOH (A) and 10 mM NH_4_OAc in ACN/H_2_O (95:5) at pH 5 (B). Methods and gradients used were the following: Generic method. Linear gradient: 0–0.2 min, 5% B; 0.2–2.7 min, 5–95% B; 2.7–2.8 min, 95–100% B; 2.8–3.0 min, 100% B. Flow rate: 0.5 mL/min. Apolar method. Gradient: 0–0.2 min, 50% B; 0.2–2.7 min, 50–100% B; 2.7–3.0 min, 100% B. Flow rate: 0.5 mL/min. Compounds were named using the naming algorithm developed by CambridgeSoft Corporation and used in ChemBioDraw Ultra 19.0. All final compounds displayed ≥96% purity as determined by UPLC-MS analysis.

#### 4.1.2 General Procedure A for the Synthesis of Final 1,2,4-Thiadiazole Derivatives **1**–**7**, **9**–**11**, **13**–**16**, **19**, **20**, **23**–**31**, and **33**–**43**

To a suspension of thiadiazolium bromide salt **47b**–**78b**, **132b**, and **133b** (1.0 equiv) in the appropriate nitrile **79**, **80**, and **82** (100 equiv) solvent, TEA (for **47b**–**78b**, 1.5–2.5 equiv) or DiPEA (for **132b** and **133b**, 1 equiv) were added. For the 1,2,4-thiadiazolylidenes **2**, fluoroacetonitrile **81** (3 equiv) was added to the suspension of **47b** in THF (0.2 M) followed by TEA (1.5 equiv). The mixture became a clear solution, which was refluxed for 0.5–2 h and then quenched with ice. The crude was extracted with DCM (3 × 10 mL), washed with water (2 × 10 mL), and dried over Na_2_SO_4_, and the solvent was evaporated in vacuum. The crude material was purified via flash silica gel column chromatography or recrystallized from the appropriate solvent, unless otherwise noted.

#### 4.1.3 General Procedure B for the Synthesis of 1,2,4-Thiadiazolylidenes **8**, **17**, and **32**

To a stirring solution of the appropriate aryl methyl ether derivative **7**, **16**, and **31** (1 equiv) in dry DCM (0.2 M) and under an inert atmosphere, a solution 1 M BBr_3_ in DCM (2.0–2.5 equiv) was slowly added through the septum with a syringe at 0 °C. The reaction mixture was left to stir at the same temperature for 30 min and then overnight at room temperature. Afterward, the mixture was quenched with water, the crude was extracted with EtOAc (3 × 10 mL), washed with NaOH 1 M (2 × 10mL), and dried over Na_2_SO_4_, and the solvent was evaporated in vacuum. The crude material was purified via flash silica gel column chromatography, then washed with *n*-hexane, and filtered to give the desired compound, unless otherwise noted.

#### 4.1.4 General Procedure C for the Synthesis of Final 1,3,4-Thiadiazolylidenes **44** and **45** and 1,3-Thiazolylidene **46**

The appropriate 3-benzyl intermediate **134d-136b** (1 equiv) was dissolved in dry DCM (0.2 M), and pyridine (1.2 equiv) was added to the solution under inert conditions. The mixture was cooled to 0 °C, and a solution of *N*-phenylbenzimidoyl chloride **108b** (1.1 equiv) in dry DCM (0.3 M) was slowly added. When the addition was completed, the mixture was left stirring at 0 °C for 1 h and then at room temperature for additional 12 h. Afterward, the reaction mixture was quenched with water and extracted with DCM (3 × 20mL). The organic layers were combined, washed with water (2 × 20mL), dried over Na_2_SO_4_, and concentrated in vacuum. The remaining crude material was purified with flash silica gel column chromatography.

#### 4.1.5 General Procedure D_1_ for the Synthesis of Imidoylthioureas **48a**–**53a**, **58a**, **62a**, **65a**, **66a**, **77a**, and **78a**

A mixture of *N*-arylbenzamidines **83**–**85** (1.00 equiv) and the appropriate isothiocyanates **86**–**96** (1.00 equiv) in dry 1,2-dichloroethane (0.6 M) was heated at 55 °C for 22 h. Afterward, the reaction mixture was cooled to room temperature and the solvent was evaporated. The resulting crude material was purified via flash silica gel column chromatography to give the desired compound, unless otherwise noted.

#### 4.1.6 General Procedure D_2_ for the Synthesis of Imidoylthioureas **47a**, **54a**–**57a**, **59a**–**61a**, **63a**, **64a**, and **67a**–**76a**

Over a solution of the appropriate imidoyl chloride **108b**–**117b** (1.0 equiv) in anhydrous acetone (0.3 M) at −15 °C and under inert conditions, a solution of sodium thyocianate (1.1 equiv) in acetone (0.5 M) was slowly added. For the imidoylthioureas **71a** and **72a**, the corresponding imidoyl chloride was obtained as hydrochloride salt, and therefore, it was previously stirred with TEA (1 equiv) for 15 min at −15 °C. After the addition was completed, the mixture was allowed to reach 0 °C, and then, the appropriate primary amine **118**–**129** (1.1 equiv) was added. The resulting reaction mixture was stirred at room temperature for 12–24 h and then filtered through a plug of Celite. The solvent was removed in vacuum from the filtrate, and the remaining crude material was purified with flash silica gel column chromatography unless otherwise noted.

#### 4.1.7 General Procedure E for the Synthesis of Hydrobromide Iminothiadiazoles **47b**–**78b**, **132b**, and **133b**

To a solution of the appropriate thiourea **47a**–**78a**, **132a**, and **133a** (1.0 equiv) in a mixture of DCM/EtOAc (1/2 v/v, 0.2 M), a 0.5 M solution of bromine (1.5–2.0 equiv) in EtOAc was added dropwise at 0 °C. Upon completion of the addition, PE was added (∼1 mL) and the resulting mixture was left stirring at 5 °C for 1 h and then RT for 12 h. The precipitate formed was isolated, washed with a mixture of PE/EtOAc (2/1 v/v), and dried in vacuum to afford the desired compound.

#### 4.1.8 General Procedure F for the Synthesis of Amides **108a**–**117a**

A solution of aromatic or heteroaromatic acyl chloride **97**–**103** (1.0 equiv) in dry THF (5 M) was slowly added at 0 °C to a solution of the appropriate amine **104**–**107** (1.05 equiv) and TEA (1.1–2 equiv) in dry THF (2 M). The reaction mixture was stirred for 2–6 h at room temperature. The formed triethylammonium chloride was removed by filtration and washed with THF. The solvent was removed in vacuum from the filtrate to give a solid residue that was washed with pentane and filtered to afford the desired compound.

#### 4.1.9 General Procedure G for the Synthesis of Imidoyl Chlorides **108b**–**110b** and **112b**–**117b**

The appropriate amide **108a**–**110a** and **112a**–**117a** (1.0 equiv) was dissolved in thionyl chloride (4.3 equiv), and the resulting mixture was heated at 70 °C for 2–2.5 h. Then, the reaction mixture was cooled to room temperature, and the remaining thionyl chloride was removed in vacuum to afford the desired compound that was used in the next step without further purification.

Below, we report the characterization of compounds **108b** and **112b**. (See also the Supporting Information).

#### 4.1.10 General Procedure H for the Synthesis of 2-Trifluoroacetylamino-1,3,4-thiadiazoles **134b** and **135b** and 2-Trifluoroacetylaminothiazole **136b**

Commercially available 1,3,4-thiadiazoles **134a** and **135a** and thiazole **136a** (1 equiv) were dissolved in dry toluene (0.3 M), and the mixture was cooled to 0 °C. Then, trifluoroacetic anhydride (1.2 equiv) was added dropwise under nitrogen. When the addition was completed, the mixture was warmed to room temperature and left stirring for additional 12 h. Afterward, the reaction mixture was quenched with water and the aqueous layer extracted with EtOAc (3 × 20mL). The organic phases were combined, washed with water (2 × 20mL), dried over Na_2_SO_4_, and concentrated in vacuum. The afforded trifluoroacetyl protected derivatives were used in the next step without further purification.

#### 4.1.11 General Procedure I for the Synthesis of 2-Trifluoroacetylamino-3-benzyl-1,3,4-thiadiazoles **134c** and **135c** and 2-Trifluoroacetylamino-3-benzyl-thiazole **136c**

The appropriate 2-trifluoroacetylamino-1,3,4-thiadiazoles **134b** and **135b** or 2-trifluoroacetylamino thiazole **136b** (1 equiv) was dissolved in dry DMF (0.3 M), and K_2_CO_3_ (1.2 equiv) was added to the solution under an inert atmosphere. Then, (bromomethyl)benzene **137** (1.2 equiv) was added dropwise and the mixture was stirred at room temperature for 24 h. Afterward, the reaction mixture was quenched with water and extracted with EtOAc (3 × 20mL). The organic layers were combined, washed with water (2 × 20mL), dried over Na_2_SO_4_, and concentrated in vacuum. The afforded 2-trifluoroacetylamino-3-benzyl intermediates were used in the next step without further purification.

#### 4.1.12 General Procedure J for the Synthesis of 3-Benzyl-1,3,4-thiadiazoles **134d** and **135d** and 3-Benzylthiazole **136d**

To a stirring solution of the appropriate trifluoroacetyl-protected compounds **134c**–**136c** (1 equiv) in THF (0.25 M), a 5% aqueous solution of NaOH (2 equiv) was added and the reaction mixture was stirred at room temperature until TLC indicated the total consumption of the starting material. Afterward, acetic acid was added to adjust to pH = 7, and the product was subsequently extracted with EtOAc (3 × 10mL). The organic layers were combined, dried over Na_2_SO_4_, and concentrated in vacuum to afford the corresponding 3-benzyl intermediates that were used in the next step without further purification.

##### 4.1.12.1 (*E*)*-N-*((*Z*)-4-Benzyl-3-ethyl-1,2,4-thiadiazol-5(4*H*)-ylidene)-*N′*-phenylbenzimidamide (**1**)

*N*-Benzyl-2,3-diphenyl-1,2,4-thiadiazol-5(2*H*)-imine hydrobromide **47b** (403 mg, 1.05 mmol), propionitrile **80** (7.5 mL, 105 mmol), and TEA (220 μL, 1.6 mmol) were reacted according to general procedure A. The crude product was purified by flash silica gel chromatography (PE/EtOAc in 95/5 ratio) to achieve the final compound **1** as a light yellow solid (205 mg, yield 49%). ^1^H NMR (401 MHz, DMSO-*d*_6_) δ 7.43–7.36 (m, 4H), 7.35–7.27 (m, 6H), 7.26–7.20 (m, 2H), 7.05–6.98 (m, 1H), 6.78–6.74 (m, 2H), 5.57 (s, 2H), 2.72 (q, *J* = 7.3 Hz, 2H), 1.18 (t, *J* = 7.3 Hz, 3H) ppm. ^13^C NMR (101 MHz, DMSO-*d*_6_) δ 171.02, 158.54, 157.56, 146.85, 135.98, 134.72, 129.48, 129.24 (2C), 128.94 (2C), 128.90 (2C), 127.98 (2C), 127.75, 126.85 (2C), 123.00, 122.04 (2C), 48.18, 22.43, 9.93 ppm. Rt = 4.65 min (apolar method); ESI-MS for C_24_H_22_N_4_S: calculated 398.16, found *m*/*z* 399.18 [M + H]^+^; UPLC-MS purity (UV 215 nm) >99.5%.

##### 4.1.12.2 (*E*)-*N*-((*Z*)-4-Benzyl-3-(fluoromethyl)-1,2,4-thiadiazol-5(4*H*)-ylidene)-*N′*-phenylbenzimidamide (**2**)

*N*-Benzyl-2,3-diphenyl-1,2,4-thiadiazol-5(2*H*)-imine hydrobromide **47b** (395 mg, 0.93 mmol), fluoroacetonitrile **81** (155 μL, 2.79 mmol), and TEA (194 μL, 1.4 mmol) were reacted according to general procedure A. The crude product was purified by flash silica gel chromatography (PE/EtOAc in 97/3 ratio) and washed with *n*-hexane to achieve the final compound **2** as a yellow solid (248 mg, yield 66%). ^1^H NMR (401 MHz, DMSO-*d*_6_) δ 7.43–7.38 (m, 3H), 7.37–7.32 (m, 4H), 7.30 (d, *J* = 7.6 Hz, 2H), 7.28–7.23 (m, 3H), 7.08–7.01 (m, 1H), 6.81–6.76 (m, 2H), 5.59 (d, *J* = 46.7 Hz, 2H), 5.59 (s, 2H) ppm. ^13^C NMR (101 MHz, DMSO-*d*_6_) δ 170.04, 158.32, 151.19 (d, *J*_CF_ = 18.6 Hz), 146.18, 135.67, 134.20, 129.74, 129.27 (2C), 129.00 (2C), 128.66 (2C), 128.03 (2C), 127.77, 127.21 (2C), 123.37, 122.04 (2C), 77.38 (d, *J*_CF_ = 168.1 Hz), 48.87 ppm. Rt = 4.22 min (apolar method); ESI-MS for C_23_H_19_FN_4_S: calculated 402.13, found *m*/*z* 403.25 [M + H]^+^; UPLC-MS purity (UV 215 nm) >99.5%.

##### 4.1.12.3 (*E*)-*N*-((*Z*)-4-Benzyl-3-isopropyl-1,2,4-thiadiazol-5(4*H*)-ylidene)-*N′*-phenylbenzimidamide (**3**)

*N*-Benzyl-2,3-diphenyl-1,2,4-thiadiazol-5(2*H*)-imine hydrobromide **47b** (1.2 g, 2.8 mmol), isobutyronitrile **82** (25.46 mL, 280 mmol), and TEA (600 μL, 4.2 mmol) were reacted according to general procedure A. The crude product was purified by flash silica gel chromatography (PE/EtOAc in 95/5 ratio) to achieve the final compound **3** as a yellow solid (401 mg, yield 35%). ^1^H NMR (401 MHz, DMSO-*d*_6_) δ 7.43–7.36 (m, 4H), 7.34–7.21 (m, 8H), 7.01 (tt, *J* = 7.3, 1.1 Hz, 1H), 6.75 (dd, *J* = 7.3, 1.2 Hz, 2H), 5.63 (s, 2H), 3.18 (hept, *J* = 6.7 Hz, 1H), 1.13 (d, *J* = 6.7 Hz, 6H) ppm. ^13^C NMR (101 MHz, DMSO-*d*_6_) δ 170.93, 161.45, 158.57, 146.83, 136.28, 134.71, 129.45, 129.20 (2C), 128.92 (2C), 128.85 (2C), 127.95 (2C), 127.68, 126.60 (2C), 123.00, 122.01 (2C), 48.25, 27.87, 20.94 ppm. Rt = 5.04 min (apolar method); ESI-MS for C_25_H_24_N_4_S: calculated 412.17, found *m*/*z* 413.38 [M + H]^+^; UPLC-MS purity (UV 215 nm) >99.5%.

##### 4.1.12.4 (*E*)-*N*-((*Z*)-3-Methyl-4-phenethyl-1,2,4-thiadiazol-5(4*H*)-ylidene)-*N′*-phenylbenzimidamide (**4**)

*N*-Phenethyl-2,3-diphenyl-1,2,4-thiadiazol-5(2*H*)-imine hydrobromide **48b** (200 mg, 0.46 mmol), ACN **79** (2.402 mL, 46 mmol), and TEA (96 μL, 0.69 mmol) were reacted according to general procedure A. The crude product was purified by flash silica gel chromatography (PE/EtOAc in 9/1 ratio) and washed with *n*-hexane to achieve the final compound **4** as a shiny yellow solid (115 mg, yield 63%). ^1^H NMR (401 MHz, DMSO-*d*_6_) δ 7.51–7.46 (m, 2H), 7.40–7.29 (m, 5H), 7.28–7.20 (m, 5H), 7.01 (t, *J* = 7.0 Hz, 1H), 6.77–6.73 (m, 2H), 4.42 (t, *J* = 7.2 Hz, 2H), 3.19–3.12 (m, 2H), 2.21 (s, 3H) ppm. ^13^C NMR (101 MHz, DMSO-*d*_6_) δ 169.97, 158.53, 153.61, 146.98, 138.14, 134.93, 129.48, 129.33 (2C), 128.96 (2C), 128.93 (2C), 128.60 (2C), 128.02 (2C), 126.72, 122.92, 122.04 (2C), 47.56, 33.26, 15.72 ppm. Rt = 4.32 min (apolar method); ESI-MS for C_24_H_22_N_4_S: calculated 398.16, found *m*/*z* 399.25 [M + H]^+^; UPLC-MS purity (UV 215 nm) 99%.

##### 4.1.12.5 (*E*)-*N*-((*Z*)-3-Methyl-4-(3-phenylpropyl)-1,2,4-thiadiazol-5(4*H*)-ylidene)-*N′*-phenylbenzimidamide (**5**)

2,3-Diphenyl-N-(3-phenylpropyl)-1,2,4-thiadiazol-5(2*H*)-imine hydrobromide **49b** (300 mg, 0.66 mmol), ACN **79** (3.447 mL, 66 mmol), and TEA (139 μL, 0.99 mmol) were reacted according to general procedure A. The crude product was purified by flash silica gel chromatography (PE/EtOAc in 9/1 ratio) and washed with *n*-hexane to achieve the final compound **5** as a light yellow solid (204 mg, yield 75%). ^1^H NMR (401 MHz, DMSO-*d*_6_) δ 7.43–7.38 (m, 2H), 7.38–7.25 (m, 7H), 7.25–7.17 (m, 3H), 6.99 (ddt, *J* = 7.7, 7.0, 1.2 Hz, 1H), 6.76–6.70 (m, 2H), 4.23 (t, *J* = 7.7 Hz, 2H), 2.75 (t, *J* = 7.4 Hz, 2H), 2.46 (s, 3H), 2.14 (p, *J* = 7.5 Hz, 2H) ppm. ^13^C NMR (101 MHz, DMSO-*d*_6_) δ 170.14, 158.29, 153.53, 146.97, 140.75, 134.75, 129.41, 129.26 (2C), 128.88 (2C), 128.32 (2C), 128.26 (2C), 127.93 (2C), 125.94, 122.83, 121.93 (2C), 45.43, 32.11, 28.63, 15.94 ppm. Rt = 4.61 min (apolar method); ESI-MS for C_25_H_24_N_4_S: calculated 412.17, found *m*/*z* 413.21 [M + H]^+^; UPLC-MS purity (UV 215 nm) >99.5%.

##### 4.1.12.6 (*E*)-*N*-((*Z*)-3-Methyl-4-(4-methylbenzyl)-1,2,4-thiadiazol-5(4*H*)-ylidene)-*N′*-phenylbenzimidamide (**6**)

*N*-(4-Methylbenzyl)-2,3-diphenyl-1,2,4-thiadiazol-5(2*H*)-imine hydrobromide **50b** (216 mg, 0.49 mmol), ACN **79** (2.559 mL, 49 mmol), and TEA (103 μL, 0.74 mmol) were reacted according to general procedure A. The crude product was purified by flash silica gel chromatography (PE/EtOAc in 9/1 ratio) and washed with *n*-hexane to achieve the final compound **6** as a shiny yellow solid (161 mg, yield 82%). ^1^H NMR (401 MHz, DMSO-*d*_6_) δ 7.45–7.40 (m, 2H), 7.37–7.26 (m, 3H), 7.26–7.17 (m, 6H), 7.04–6.98 (m, 1H), 6.78–6.73 (m, 2H), 5.51 (s, 2H), 2.41 (s, 3H), 2.28 (s, 3H) ppm. ^13^C NMR (101 MHz, DMSO-*d*_6_) δ 170.66, 158.46, 153.65, 146.80, 137.01, 134.71, 132.83, 129.44, 129.38 (2C), 129.20 (2C), 128.87 (2C), 127.95 (2C), 126.97 (2C), 122.94, 121.98 (2C), 48.30, 20.64, 16.20 ppm. Rt = 4.57 min (apolar method); ESI-MS for C_24_H_22_N_4_S: calculated 398.16, found *m*/*z* 399.16 [M + H]^+^; UPLC-MS purity (UV 215 nm) >99.5%.

##### 4.1.12.7 (*E*)-*N*-((*Z*)-4-(4-Methoxybenzyl)-3-methyl-1,2,4-thiadiazol-5(4*H*)-ylidene)-*N′*-phenylbenzimidamide (**7**)

*N*-(4-Methoxybenzyl)-2,3-diphenyl-1,2,4-thiadiazol-5(2*H*)-imine hydrobromide **51b** (114 mg, 0.25 mmol), ACN **79** (1.306 mL, 25 mmol), and TEA (53 μL, 0.38 mmol) were reacted according to general procedure A. The crude product was purified by flash silica gel chromatography (PE/EtOAc in 9/1 ratio) and washed with *n*-hexane to achieve the final compound **7** as a shiny yellow solid (62 mg, yield 60%). ^1^H NMR (401 MHz, DMSO-*d*_6_) δ 7.47–7.42 (m, 2H), 7.38–7.26 (m, 5H), 7.23 (t, *J* = 7.8 Hz, 2H), 7.01 (t, *J* = 7.4 Hz, 1H), 6.95 (d, *J* = 8.6 Hz, 2H), 6.76 (d, *J* = 7.0 Hz, 2H), 5.48 (s, 2H), 3.73 (s, 3H), 2.43 (s, 3H) ppm. ^13^C NMR (101 MHz, DMSO-*d*_6_) δ 170.69, 158.85, 158.49, 153.68, 146.83, 134.75, 129.47, 129.23 (2C), 128.91 (2C), 128.65 (2C), 127.99 (2C), 127.80, 122.96, 122.00 (2C), 114.25 (2C), 55.09, 48.05, 16.29 ppm. Rt = 3.93 min (apolar method); ESI-MS for C_24_H_22_N_4_OS: calculated 414.15, found *m*/*z* 415.41 [M + H]^+^; UPLC-MS purity (UV 215 nm) 99%.

##### 4.1.12.8 (*E*)-*N*-((*Z*)-4-(4-Hydroxybenzyl)-3-methyl-1,2,4-thiadiazol-5(4*H*)-ylidene)-*N′*-phenylbenzimidamide (**8**)

(*E*)-*N*-((*Z*)-4-(4-Methoxybenzyl)-3-methyl-1,2,4-thiadiazol-5(4*H*)-ylidene)-*N′*-phenylbenzimidamide **7** (266 mg, 0.64 mmol) and 2.25 equiv of BBr_3_ (1.44 mmol) were reacted according to the general procedure B. The crude material was purified via flash silica gel column chromatography (PE/EtOAc in 8/2 ratio), then washed with *n*-hexane, and filtered to give the final compound **8** as a yellow solid (87 mg, yield 34%). ^1^H NMR (401 MHz, DMSO-*d*_6_) δ 9.50 (s, 1H), 7.49–7.40 (m, 2H), 7.32 (ddd, *J* = 14.1, 7.7, 5.9 Hz, 3H), 7.23 (t, *J* = 7.6 Hz, 2H), 7.18 (d, *J* = 8.4 Hz, 2H), 7.01 (t, *J* = 7.4 Hz, 1H), 6.76 (dd, *J* = 8.7, 2.3 Hz, 4H), 5.43 (s, 2H), 2.42 (s, 3H) ppm. ^13^C NMR (101 MHz, DMSO-*d*_6_) δ 170.72, 158.54, 157.06, 153.78, 146.83, 134.77, 129.49, 129.26 (2C), 128.92 (2C), 128.71 (2C), 128.01 (2C), 126.03, 122.98, 122.03 (2C), 115.56 (2C), 48.22, 18.90, 16.33 ppm. Rt = 5.26 min (generic method); ESI-MS for C_23_H_20_N_4_OS: calculated 400.14, found *m*/*z* 401.15 [M + H]^+^; UPLC-MS purity (UV 215 nm) 99%.

##### 4.1.12.9 (*E*)-*N*-((*Z*)-4-(4-Fluorobenzyl)-3-methyl-1,2,4-thiadiazol-5(4*H*)-ylidene)-*N′*-phenylbenzimidamide (**9**)

*N*-(4-Fluorobenzyl)-2,3-diphenyl-1,2,4-thiadiazol-5(2*H*)-imine hydrobromide **52b** (90 mg, 0.2 mmol), ACN **79** (1.045 mL, 20 mmol), and TEA (41 μL, 0.3 mmol) were reacted according to general procedure A. The crude product was purified by flash silica gel chromatography (PE/EtOAc in 9/1 ratio) and washed with *n*-hexane to achieve the final compound **9** as a yellow solid (48 mg, yield 60%). ^1^H NMR (401 MHz, DMSO-*d*_6_) δ 7.45–7.37 (m, 4H), 7.37–7.27 (m, 3H), 7.27–7.19 (m, 4H), 7.01 (tt, *J* = 7.3, 1.2 Hz, 1H), 6.79–6.73 (m, 2H), 5.54 (s, 2H), 2.42 (s, 3H) ppm. ^13^C NMR (101 MHz, DMSO-*d*_6_) δ 170.62, 161.62 (d, *J*_CF_ = 244.0 Hz), 158.46, 153.58, 146.76, 134.66, 132.11 (d, *J*_CF_ = 3.1 Hz), 129.49, 129.31 (d, *J*_CF_ = 8.4 Hz, 2C), 129.22 (2C), 128.91 (2C), 127.99 (2C), 123.00, 121.98 (2C), 115.70 (d, *J*_CF_ = 21.5 Hz, 2C), 47.85, 16.23 ppm. Rt = 4.12 min (apolar method); ESI-MS for C_23_H_19_FN_4_S: calculated 402.13, found *m*/*z* 403.09 [M + H]^+^; UPLC-MS purity (UV 215 nm) >99.5%.

##### 4.1.12.10 (*E*)-*N*-((*Z*)-4-(4-Chlorobenzyl)-3-methyl-1,2,4-thiadiazol-5(4*H*)-ylidene)-*N′*-phenylbenzimidamide (**10**)

*N*-(4-Chlorobenzyl)-2,3-diphenyl-1,2,4-thiadiazol-5(2*H*)-imine hydrobromide **53b** (70 mg, 0.15 mmol), ACN **79** (783 μL, 15 mmol), and TEA (32 μL, 0.23 mmol) were reacted according to general procedure A. The crude product was purified by flash silica gel chromatography (PE/EtOAc in 9/1 ratio) and washed with *n*-hexane to achieve the final compound **10** as a shiny yellow solid (50 mg, yield 80%). ^1^H NMR (401 MHz, DMSO-*d*_6_) δ 7.48–7.44 (m, 2H), 7.43–7.39 (m, 2H), 7.37–7.26 (m, 5H), 7.26–7.21 (m, 2H), 7.04–6.98 (m, 1H), 6.78–6.73 (m, 2H), 5.54 (s, 2H), 2.41 (s, 3H) ppm. ^13^C NMR (101 MHz, DMSO-*d*_6_) δ 170.58, 158.45, 153.55, 146.75, 134.89, 134.62, 132.40, 129.50, 129.22 (2C), 128.97 (2C), 128.92 (2C), 128.86 (2C), 127.98 (2C), 123.02, 121.98 (2C), 47.90, 16.19 ppm. Rt = 4.55 min (apolar method); ESI-MS for C_23_H_19_ClN_4_S: calculated 418.10, found *m*/*z* 419.13/421.16 [M + H]^+^; UPLC-MS purity (UV 215 nm) 99.5%.

##### 4.1.12.11 (*E*)-*N*-((*Z*)-4-(4-Bromobenzyl)-3-methyl-1,2,4-thiadiazol-5(4*H*)-ylidene)-*N′*-phenylbenzimidamide (**11**)

*N*-(4-Bromobenzyl)-2,3-diphenyl-1,2,4-thiadiazol-5(*2H*)-imine hydrobromide **54b** (558 mg, 1.11 mmol), ACN **79** (5.80 mL, 111 mmol), and TEA (232 μL, 1.67 mmol) were reacted according to general procedure A. The crude product was purified by flash silica gel chromatography (PE/EtOAc in 88/12 ratio) to achieve the final compound **11** as a yellow solid (380 mg, yield 74%). ^1^H NMR (401 MHz, DMSO-*d*_6_) δ 7.65–7.55 (m, 2H), 7.41 (dd, *J* = 6.8, 1.6 Hz, 2H), 7.38–7.24 (m, 5H), 7.28–7.19 (m, 2H), 7.01 (tt, *J* = 7.4, 1.2 Hz, 1H), 6.75 (dd, *J* = 8.3, 1.3 Hz, 2H), 5.53 (s, 2H), 2.41 (s, 3H) ppm. ^13^C NMR (101 MHz, DMSO-*d*_6_) δ 170.58, 158.45, 153.55, 146.75, 135.31, 134.61, 131.78 (2C), 129.51, 129.28 (2C), 129.23 (2C), 128.92 (2C), 127.99 (2C), 123.03, 121.98 (2C), 120.92, 47.95, 16.19 ppm. Rt = 4.63 min (apolar method); ESI-MS for C_23_H_19_BrN_4_S: calculated 462.05, found *m*/*z* 462.89/464.83 [M + H]^+^; UPLC-MS purity (UV 215 nm) >99.5%.

##### 4.1.12.12 4-(((*Z*)-3-Methyl-5-(((*E*)-phenyl(phenylimino)methyl)imino)-1,2,4-thiadiazol-4(5*H*)-yl)methyl)benzoic Acid (**12**)

To a solution of (*E*)-*N*-((*Z*)-4-(4-bromobenzyl)-3-methyl-1,2,4-thiadiazol-5(4*H*)-ylidene)-*N′*-phenylbenzimidamide **11** (148 mg, 0.32 mmol) in dry THF (5 mL) at −78 °C, 324 μL (0.81 mmol) of 2.5 M *n*-butyllithium in *n*-hexane was added dropwise. After being stirred at −78 °C for 30 min, the mixture was treated with an excess of dry ice and stirred for an additional 30 min at −78 °C. Then, the reaction mixture was warmed to room temperature, diluted with water, and adjusted to pH 3 with aqueous 2 N HCl. The crude was extracted with DCM (×3), the organic phase was washed with water (×2), and dried over Na_2_SO_4_, and the solvent was removed under reduced pressure. Purification was performed by direct phase flash chromatography (DCM/MeOH in 99.5/0.5 ratio, acetic acid 0.1%). Then, the solid was washed with a mixture of *n*-hexane/EtOAc (9/1) and filtered to achieve the final compound **12** as a light yellow solid (35 mg, yield 26%). ^1^H NMR (401 MHz, DMSO-*d*_6_) δ 12.25 (s, 1H), 7.96 (dd, *J* = 8.3, 5.3 Hz, 2H), 7.43–7.37 (m, 4H), 7.36–7.30 (m, 1H), 7.29 (dd, *J* = 6.9, 1.3 Hz, 2H), 7.26–7.21 (m, 2H), 7.05–6.98 (m, 1H), 6.78–6.73 (m, 2H), 5.63 (s, 2H), 2.40 (s, 3H) ppm. ^13^C NMR (101 MHz, DMSO-*d*_6_) δ 170.58, 167.00, 158.48, 153.62, 146.77, 140.61, 134.61, 130.53, 129.91 (2C), 129.52, 129.24 (2C), 128.94 (2C), 127.99 (2C), 126.98 (2C), 123.05, 122.01 (2C), 48.37, 16.19 ppm. Rt = 4.30 min (apolar method); ESI-MS for C_24_H_20_N_4_O_2_S: calculated 428.13, found *m*/*z* 429.27 [M + H]^+^, 427.43 [M – H]^−^; UPLC-MS purity (UV 215 nm) 98.5%.

##### 4.1.12.13 (*E*)-*N*-((*Z*)-4-(4-Aminobenzyl)-3-methyl-1,2,4-thiadiazol-5(4*H*)-ylidene)-*N′*-phenylbenzimidamide (**13**)

4-(((2,3-Diphenyl-1,2,4-thiadiazol-5(2*H*)-ylidene)amino)methyl) aniline dihydrobromide **55b** (137 mg, 0.26 mmol), ACN **79** (1.358 mL, 26 mmol), and TEA (65 μL, 0.47 mmol) were reacted according to general procedure A. The crude product was purified by flash silica gel chromatography (PE/EtOAc in 7/3 ratio) and washed with *n*-hexane to achieve the final compound **13** as a shiny yellow solid (42 mg, yield 40%). ^1^H NMR (401 MHz, DMSO-*d*_6_) δ 7.48–7.42 (m, 2H), 7.38–7.27 (m, 3H), 7.23 (t, *J* = 7.8 Hz, 2H), 7.05 (d, *J* = 8.4 Hz, 2H), 7.00 (tt, *J* = 7.3, 1.3 Hz, 1H), 6.75 (dd, *J* = 8.2, 1.3 Hz, 2H), 6.54 (d, *J* = 8.4 Hz, 2H), 5.35 (s, 2H), 5.13 (s, 2H), 2.43 (s, 3H) ppm. ^13^C NMR (101 MHz, DMSO-*d*_6_) δ 170.77, 158.54, 153.85, 148.48, 146.92, 134.87, 129.46, 129.27 (2C), 128.92 (2C), 128.46 (2C), 128.01 (2C), 122.93, 122.46, 122.04 (2C), 113.88 (2C), 48.50, 16.41 ppm. Rt = 3.02 min (apolar method); ESI-MS for C_23_H_21_N_5_S: calculated 399.15, found *m*/*z* 400.12 [M + H]^+^; UPLC-MS purity (UV 215 nm) 96.5%.

##### 4.1.12.14 (*E*)-*N*-((*Z*)-3-Methyl-4-(3-methylbenzyl)-1,2,4-thiadiazol-5(4*H*)-ylidene)-*N′*-phenylbenzimidamide (**14**)

*N*-(3-Methylbenzyl)-2,3-diphenyl-1,2,4-thiadiazol-5(2*H*)-imine hydrobromide **56b** (342 mg, 0.78 mmol), ACN **79** (4.07 mL, 78 mmol), and TEA (162 μL, 1.16 mmol) were reacted according to general procedure A. The crude product was purified by flash silica gel chromatography (PE/EtOAc in 93/7 ratio) to achieve the final compound **14** as a yellow solid (100 mg, yield 74%). ^1^H NMR (401 MHz, DMSO-*d*_6_) δ 7.46–7.38 (m, 2H), 7.37–7.19 (m, 6H), 7.17 (s, 1H), 7.13 (d, *J* = 7.6 Hz, 1H), 7.07 (d, *J* = 7.7 Hz, 1H), 7.01 (t, *J* = 7.4 Hz, 1H), 6.80–6.72 (m, 2H), 5.51 (s, 2H), 2.42 (s, 3H), 2.30 (s, 3H) ppm. ^13^C NMR (101 MHz, DMSO-*d*_6_) δ 170.64, 158.50, 153.72, 146.81, 138.04, 135.79, 134.73, 129.48, 129.22 (2C), 128.91 (2C), 128.81, 128.44, 127.98 (2C), 127.67, 123.99, 122.99, 122.01 (2C), 48.55, 21.01, 16.28 ppm. Rt = 4.39 min (apolar method); ESI-MS for C_24_H_22_N_4_S: calculated 398.16, found *m*/*z* 399.09 [M + H]^+^; UPLC-MS purity (UV 215 nm) >99.5%.

##### 4.1.12.15 (*E*)-*N*-((*Z*)-3-Methyl-4-(3-(trifluoromethyl)benzyl)-1,2,4-thiadiazol-5(4*H*)-ylidene)-*N′*-phenylbenzimidamide (**15**)

*N*-(3-(Trifluoromethyl)benzyl)-2,3-diphenyl-1,2,4-thiadiazol-5(2*H*)-imine hydrobromide **57b** (328 mg, 0.67 mmol), ACN **79** (3.48 mL, 67 mmol), and TEA (140 μL, 1 mmol) were reacted according to general procedure A. The crude product was purified by flash silica gel chromatography (PE/EtOAc in 96/4 ratio) to achieve the final compound **15** as a yellow solid (198 mg, yield 65%). ^1^H NMR (401 MHz, DMSO-*d*_6_) δ 7.87 (s, 1H), 7.71 (d, *J* = 7.7 Hz, 1H), 7.63 (t, *J* = 7.7 Hz, 1H), 7.57 (d, *J* = 7.8 Hz, 1H), 7.40–7.30 (m, 3H), 7.30–7.25 (m, 2H), 7.23 (t, *J* = 8.0 Hz, 2H), 7.06–6.97 (m, 1H), 6.79–6.71 (m, 2H), 5.62 (s, 2H), 2.47 (s, 3H) ppm. ^13^C NMR (101 MHz, DMSO-*d*_6_) δ 170.49, 158.40, 153.50, 146.72, 137.34, 134.62, 131.09 (q, *J*_CF_ = 1.6 Hz), 130.09, 129.49, 129.15 (2C), 128.92 (2C), 127.93 (2C), 124.57 (q, *J*_CF_ = 3.7, Hz), 122.88, 121.97 (2C), 48.26, 16.29 ppm. Rt = 4.40 min (apolar method); ESI-MS for C_24_H_19_F_3_N_4_S: calculated 452.13, found *m*/*z* 453.18 [M + H]^+^, 451.36 [M – H]^−^, 511.35 [M + OAc]^−^; UPLC-MS purity (UV 215 nm) >99.5%.

##### 4.1.12.16 (*E*)-*N*-((*Z*)-4-(3-Methoxybenzyl)-3-methyl-1,2,4-thiadiazol-5(4*H*)-ylidene)-*N′*-phenylbenzimidamide (**16**)

*N*-(3-Methoxybenzyl)-2,3-diphenyl-1,2,4-thiadiazol-5(2*H*)-imine hydrobromide **58b** (191 mg, 0.42 mmol), ACN **79** (2.194 mL, 42 mmol), and TEA (88 μL, 0.63 mmol) were reacted according to the general procedure. The crude product was purified by flash silica gel chromatography (PE/EtOAc in 9/1 ratio) and washed with *n*-hexane to achieve the final compound **16** as a shiny yellow solid (134 mg, yield 77%). ^1^H NMR (401 MHz, DMSO-*d*_6_) δ 7.45–7.40 (m, 2H), 7.36–7.21 (m, 6H), 7.04–6.98 (m, 1H), 6.95–6.87 (m, 2H), 6.85–6.80 (m, 1H), 6.78–6.74 (m, 2H), 5.52 (s, 2H), 3.72 (s, 3H), 2.42 (s, 3H) ppm. ^13^C NMR (101 MHz, DMSO-*d*_6_) δ 170.63, 159.47, 158.45, 153.70, 146.79, 137.41, 134.69, 130.09, 129.49, 129.22 (2C), 128.91 (2C), 127.98 (2C), 123.00, 122.00 (2C), 118.86, 113.15, 112.94, 55.01, 48.43, 16.22 ppm. Rt = 4.00 min (apolar method); ESI-MS for C_24_H_22_N_4_OS: calculated 414.15, found *m*/*z* 415.16 [M + H]^+^; UPLC-MS purity (UV 215 nm) >99.5%.

##### 4.1.12.17 (*E*)-*N*-((*Z*)-4-(3-Hydroxybenzyl)-3-methyl-1,2,4-thiadiazol-5(4*H*)-ylidene)-*N′*-phenylbenzimidamide (**17**)

(*E*)-*N*-((*Z*)-4-(3-Methoxybenzyl)-3-methyl-1,2,4-thiadiazol-5(4*H*)-ylidene)-*N′*-phenylbenzimidamide **16** (136 mg, 0.33 mmol) and 2.25 equiv of BBr_3_ (0.75 mmol) were reacted according to the general procedure B. The crude material was purified via flash silica gel column chromatography (PE/EtOAc in 8/2 ratio), then washed with *n*-hexane, and filtered to give the final compound **17** as a yellow solid (78 mg, yield 59%). ^1^H NMR (401 MHz, DMSO-*d*_6_) δ 9.52 (s, 1H), 7.46–7.40 (m, 2H), 7.34–7.15 (m, 6H), 7.01 (t, *J* = 7.4 Hz, 1H), 6.79–6.63 (m, 5H), 5.49 (s, 2H), 2.39 (s, 3H) ppm. ^13^C NMR (101 MHz, DMSO-*d*_6_) δ 170.71, 158.52, 157.74, 153.77, 146.81, 137.22, 134.68, 129.95, 129.49, 129.26 (2C), 128.92 (2C), 127.98 (2C), 123.00, 122.01 (2C), 117.42, 114.76, 113.32, 48.37, 16.16 ppm. Rt = 5.39 min (generic method); ESI-MS for C_23_H_20_N_4_OS: calculated 400.14, found *m*/*z* 401.12 [M + H]^+^, 399.24 [M – H]^−^; UPLC-MS purity (UV 215 nm) 98%.

##### 4.1.12.18 (*E*)-*N*-((*Z*)-4-(3-Isopropoxybenzyl)-3-methyl-1,2,4-thiadiazol-5(4*H*)-ylidene)-*N′*-phenylbenzimidamide (**18**)

(*E*)-*N*-((*Z*)-4-(3-Hydroxybenzyl)-3-methyl-1,2,4-thiadiazol-5(4*H*)-ylidene)-*N′*-phenylbenzimidamide **17** (270 mg, 0.47 mmol) was dissolved in dry DMF (0.2 M) and treated with K_2_CO_3_ (194 mg, 1.4 mmol) at room temperature for 1 h. Then, 2-bromopropane (129 μL, 1.4 mmol) was added and the reaction mixture was heated at 70 °C for 3 h. After completion of the reaction, the mixture was cooled to room temperature, diluted with H_2_O, and extracted with EtOAc (3 × 10mL). The organic phases were combined, washed with water (2 × 10mL) and brine (2 × 10mL), and dried over anhydrous Na_2_SO_4_, and the solvent was evaporated under reduced pressure. The crude product was purified by flash silica gel chromatography (PE/EtOAc in 92/8 ratio) to achieve the final compound **18** as a yellow solid (37 mg, yield 18%). ^1^H NMR (401 MHz, DMSO-*d*_6_) δ 7.44 (t, *J* = 1.5 Hz, 1H), 7.42 (d, *J* = 1.7 Hz, 1H), 7.37–7.32 (m, 1H), 7.31–7.26 (m, 3H), 7.26–7.20 (m, 2H), 7.01 (tdd, *J* = 7.4, 2.2, 1.1 Hz, 1H), 6.90 (t, *J* = 2.1 Hz, 1H), 6.86 (dd, *J* = 8.3, 2.4 Hz, 1H), 6.81 (dd, *J* = 7.6, 1.7 Hz, 1H), 6.79–6.74 (m, 2H), 5.51 (s, 2H), 4.50 (hept, *J* = 6.2 Hz, 1H), 2.43 (s, 3H), 1.21 (dq, *J* = 6.0, 0.5 Hz, 6H) ppm. ^13^C NMR (101 MHz, DMSO-*d*_6_) δ 170.63, 158.43, 157.64, 153.71, 146.81, 137.45, 134.70, 130.09, 129.51, 129.24 (2C), 128.93 (2C), 127.98 (2C), 123.01, 122.00 (2C), 118.71, 114.65, 114.52, 69.10, 48.48, 21.69, 16.25 ppm. Rt = 4.68 min (apolar method); ESI-MS for C_26_H_26_N_4_OS: calculated 442.18, found *m*/*z* 443.31 [M + H]^+^, 441.41 [M – H]^−^; UPLC-MS purity (UV 215 nm) 96.5%.

##### 4.1.12.19 (*E*)-*N*-((*Z*)-4-(3-Ethoxybenzyl)-3-methyl-1,2,4-thiadiazol-5(4*H*)-ylidene)-*N′*-phenylbenzimidamide (**19**)

*N*-(3-Ethoxybenzyl)-2,3-diphenyl-1,2,4-thiadiazol-5(2*H*)-imine hydrobromide **59b** (600 mg, 1.28 mmol), ACN **79** (6.67 mL, 128 mmol), and TEA (260 μL, 1.92 mmol) were reacted according to the general procedure. The crude product was purified by flash silica gel chromatography (PE/EtOAc in 90/10 ratio) to achieve the final compound **19** as a yellow solid (486 mg, yield 89%). ^1^H NMR (401 MHz, DMSO-*d*_6_) δ 7.45–7.40 (m, 2H), 7.37–7.26 (m, 4H), 7.26–7.21 (m, 2H), 7.01 (ddt, *J* = 8.6, 7.1, 1.2 Hz, 1H), 6.91–6.85 (m, 2H), 6.83 (ddd, *J* = 7.6, 1.7, 0.9 Hz, 1H), 6.79–6.74 (m, 2H), 5.51 (s, 2H), 3.96 (q, *J* = 7.0 Hz, 2H), 2.42 (s, 3H), 1.28 (t, *J* = 6.9 Hz, 3H) ppm. ^13^C NMR (101 MHz, DMSO-*d*_6_) δ 170.63, 158.72, 158.45, 153.72, 146.81, 137.39, 134.70, 130.09, 129.52, 129.24 (2C), 128.93 (2C), 127.99 (2C), 123.02, 122.01 (2C), 118.83, 113.53, 113.43, 62.96, 48.46, 16.25, 14.56 ppm. Rt = 4.37 min (apolar method); ESI-MS for C_25_H_24_N_4_OS: calculated 428.17, found *m*/*z* 429.31 [M + H]^+^; UPLC-MS purity (UV 215 nm) 99%.

##### 4.1.12.20 (*E*)-*N*-((*Z*)-4-(3-Aminobenzyl)-3-methyl-1,2,4-thiadiazol-5(4*H*)-ylidene)-*N′*-phenylbenzimidamide (**20**)

3-(((2,3-Diphenyl-1,2,4-thiadiazol-5(2*H*)-ylidene)amino)methyl) aniline dihydrobromide **60b** (1.757 g, 3.4 mmol), ACN **79** (17.65 mL, 338 mmol), and TEA (1.186 mL, 8.5 mmol) were reacted according to general procedure A. The crude product was purified by flash silica gel chromatography (PE/EtOAc in 70/30 ratio) to achieve the final compound **20** as a yellow solid (230 mg, yield 17%). ^1^H NMR (401 MHz, DMSO-*d*_6_) δ 7.46–7.40 (m, 2H), 7.36–7.26 (m, 3H), 7.26–7.20 (m, 2H), 7.01 (tt, *J* = 7.6, 1.1 Hz, 2H), 6.79–6.73 (m, 2H), 6.48 (ddd, *J* = 8.0, 2.2, 1.0 Hz, 1H), 6.42 (ddd, *J* = 7.4, 1.7, 1.0 Hz, 1H), 6.39 (t, *J* = 1.9 Hz, 1H), 5.42 (s, 2H), 5.17 (s, 2H), 2.39 (s, 3H) ppm. ^13^C NMR (101 MHz, DMSO-*d*_6_) δ 170.74, 158.56, 153.89, 149.20, 146.87, 136.40, 134.73, 129.46, 129.35, 129.28 (2C), 128.93 (2C), 127.98 (2C), 122.96, 122.02 (2C), 113.99, 113.23, 111.36, 48.65, 16.21 ppm. Rt = 5.41 min (generic method); ESI-MS for C_23_H_21_N_5_S: calculated 399.15, found *m*/*z* 400.3 [M + H]^+^, 398.47 [M – H]^−^; UPLC-MS purity (UV 215 nm) >99.5%.

##### 4.1.12.21 (*E*)-*N*-((*Z*)-3-Methyl-4-(3-(methylamino)benzyl)-1,2,4-thiadiazol-5(4*H*)-ylidene)-*N′*-phenylbenzimidamide (**21**) and (*E*)-*N*-((*Z*)-4-(3-(Dimethylamino)benzyl)-3-methyl-1,2,4-thiadiazol-5(4*H*)-ylidene)-*N′*-phenylbenzimidamide (**22**)

In a screw-capped pressure tube, (*E*)-*N*-((*Z*)-4-(3-aminobenzyl)-3-methyl-1,2,4-thiadiazol-5(4*H*)-ylidene)-*N′*-phenylbenzimidamide **20** (250 mg, 0.63 mmol) was dissolved in dry DMF (4 mL), and then K_2_CO_3_ (113 mg, 0.81 mmol) and methyl iodide (47 μL, 0.75 mmol) were added. The vessel was then sealed and heated to 55 °C for 24 h. After reaction cooling, 5 mL of water was added into the mixture. The organic phase was separated, and the aqueous layer was extracted with EtOAc (3 × 10mL). The combined organic phase was washed with brine (2 × 10mL) and dried over Na_2_SO_4_. The solvent was removed under reduced pressure, and the crude product was purified by flash silica gel chromatography (A = PE, B = EtOAc, gradient 0–20% B). Yields: (**21**) yellow solid 90 mg, 35%; (**22**) yellow solid, 60 mg, 22%.

**21**) ^1^H NMR (401 MHz, DMSO-*d*_6_) δ 7.46–7.41 (m, 2H), 7.36–7.26 (m, 3H), 7.26–7.20 (m, 2H), 7.08 (t, *J* = 7.7 Hz, 1H), 7.01 (tt, *J* = 7.4, 1.1 Hz, 1H), 6.78–6.73 (m, 2H), 6.49–6.39 (m, 3H), 5.75 (q, *J* = 5.0 Hz, 1H), 5.44 (s, 2H), 2.61 (d, *J* = 5.1 Hz, 3H), 2.41 (s, 3H) ppm. ^13^C NMR (101 MHz, DMSO-*d*_6_) δ 170.72, 158.51, 153.86, 150.23, 146.87, 136.43, 134.75, 129.44, 129.25 (2C), 128.92 (2C), 127.96 (2C), 122.95, 122.00 (2C), 113.69, 110.65, 110.03, 48.77, 29.53, 16.22 ppm. Rt = 5.92 min (generic method); ESI-MS for C_24_H_23_N_5_S: calculated 413.17, found *m*/*z* 414.13 [M + H]^+^; UPLC-MS purity (UV 215 nm) 99%.

**22**) ^1^H NMR (401 MHz, DMSO-*d*_6_) δ 7.46–7.42 (m, 2H), 7.37–7.26 (m, 3H), 7.26–7.20 (m, 2H), 7.15 (dd, *J* = 8.3, 7.5 Hz, 1H), 7.03–6.98 (m, 1H), 6.80 (t, *J* = 2.1 Hz, 1H), 6.78–6.72 (m, 2H), 6.65 (dd, *J* = 8.1, 2.6 Hz, 1H), 6.48 (d, *J* = 7.8 Hz, 1H), 5.48 (s, 2H), 2.84 (s, 6H), 2.44 (s, 3H) ppm. ^13^C NMR (101 MHz, DMSO-*d*_6_) δ 170.69, 158.44, 153.82, 150.56, 146.87, 136.51, 134.77, 129.46, 129.43, 129.22 (2C), 128.91 (2C), 127.96 (2C), 122.96, 121.99 (2C), 114.16, 111.75, 111.26, 49.00, 16.30, 16.29 (2C) ppm. Rt = 4.37 min (apolar method); ESI-MS for C_25_H_25_N_5_S: calculated 427.18, found *m*/*z* 428.13 [M + H]^+^; UPLC-MS purity (UV 215 nm) 98%.

##### 4.1.12.22 (*E*)-*N*-((*Z*)-4-(3-Fluorobenzyl)-3-methyl-1,2,4-thiadiazol-5(4*H*)-ylidene)-*N′*-phenylbenzimidamide (**23**)

(*Z*)-*N*-(3-Fluorobenzyl)-2,3-diphenyl-1,2,4-thiadiazol-5(2*H*)-imine hydrobromide **61b** (416 mg, 0.94 mmol), ACN **79** (4.89 mL, 94 mmol), and TEA (197 μL, 1.41 mmol) were reacted according to general procedure A. The crude product was purified by flash silica gel chromatography (PE/EtOAc in 9/1 ratio) to achieve the final compound **23** as a yellow solid (308 mg, yield 81%). ^1^H NMR (401 MHz, DMSO-*d*_6_) δ 7.48–7.38 (m, 3H), 7.37–7.09 (m, 8H), 7.01 (t, *J* = 7.4 Hz, 1H), 6.79–6.73 (m, 2H), 5.56 (s, 2H), 2.42 (s, 3H) ppm. ^13^C NMR (101 MHz, DMSO-*d*_6_) δ 170.59, 162.24 (d, *J*_CF_ = 244.2 Hz), 158.44, 153.58, 146.76, 138.70 (d, *J*_CF_ = 7.5 Hz) 134.64, 131.00 (d, *J*_CF_ = 8.3 Hz), 129.52, 129.22 (2C), 128.92 (2C), 127.99 (2C), 123.04, 122.95 (d, *J*_CF_ = 2.8 Hz), 122.00 (2C), 114.68 (d, *J*_CF_ = 20.8 Hz), 114.10 (d, *J*_CF_ = 22.1 Hz), 48.06, 16.21 ppm. Rt = 3.99 min (apolar method); ESI-MS for C_23_H_19_FN_4_S: calculated 402.13, found *m*/*z* 403.09 [M + H]^+^, 401.21 [M – H]^−^; UPLC-MS purity (UV 215 nm) >99.5%.

##### 4.1.12.23 (*E*)-*N*-((*Z*)-4-(3-Chlorobenzyl)-3-methyl-1,2,4-thiadiazol-5(4*H*)-ylidene)-*N′*-phenylbenzimidamide (**24**)

*N*-(3-Chlorobenzyl)-2,3-diphenyl-1,2,4-thiadiazol-5(2*H*)-imine hydrobromide **62b** (133 mg, 0.29 mmol), ACN **79** (1.515 mL, 29 mmol), and TEA (61 μL, 0.43 mmol) were reacted according to general procedure A. The crude product was purified by flash silica gel chromatography (PE/EtOAc in 9/1 ratio) and washed with *n*-hexane to achieve the final compound **24** as a shiny yellow solid (115 mg, yield 95%). ^1^H NMR (401 MHz, DMSO-*d*_6_) δ 7.48 (d, *J* = 2.2 Hz, 1H), 7.46–7.37 (m, 4H), 7.37–7.27 (m, 3H), 7.26–7.20 (m, 3H), 7.05–6.98 (m, 1H), 6.78–6.73 (m, 2H), 5.54 (s, 2H), 2.44 (s, 3H) ppm. ^13^C NMR (101 MHz, DMSO-*d*_6_) δ 170.52, 158.43, 153.53, 146.73, 138.33, 134.64, 133.33, 130.83, 129.52, 129.20 (2C), 128.91 (2C), 127.99 (2C), 127.82, 127.32, 125.65, 123.04, 121.99 (2C), 48.04, 16.24 ppm. Rt = 4.53 min (apolar method); ESI-MS for C_23_H_19_ClN_4_S: calculated 418.10, found *m*/*z* 419.14/421.13 [M + H]^+^; UPLC-MS purity (UV 215 nm) 99%.

##### 4.1.12.24 (*E*)-*N*-((*Z*)-3-Methyl-4-(2-methylbenzyl)-1,2,4-thiadiazol-5(4*H*)-ylidene)-*N′*-phenylbenzimidamide (**25**)

*N*-(2-Methylbenzyl)-2,3-diphenyl-1,2,4-thiadiazol-5(2*H*)-imine hydrobromide **63b** (239 mg, 0.55 mmol), ACN **79** (2.872 mL, 55 mmol), and TEA (114 μL, 0.82 mmol) were reacted according to general procedure A. The crude product was purified by flash silica gel chromatography (PE/EtOAc in 92/8 ratio) and washed with *n*-hexane to achieve the final compound **25** as a yellow solid (111 mg, yield 51%). ^1^H NMR (401 MHz, DMSO-*d*_6_) δ 7.37–7.32 (m, 2H), 7.32–7.21 (m, 6H), 7.21–7.12 (m, 2H), 7.05–6.98 (m, 1H), 6.78–6.72 (m, 2H), 6.60 (dd, *J* = 7.5, 1.5 Hz, 1H), 5.53 (s, 2H), 2.42 (s, 3H), 2.35 (s, 3H) ppm. ^13^C NMR (101 MHz, DMSO-*d*_6_) δ 170.55, 158.58, 153.88, 146.77, 135.17, 134.64, 133.71, 130.45, 129.48, 129.21 (2C), 128.93 (2C), 127.96 (2C), 127.33, 126.46, 124.29, 123.04, 122.06 (2C), 46.45, 18.85, 15.95 ppm. Rt = 4.27 min (apolar method); ESI-MS for C_24_H_22_N_4_S: calculated 398.16, found *m*/*z* 399.33 [M + H]^+^; UPLC-MS purity (UV 215 nm) 99.5%.

##### 4.1.12.25 (*E*)-*N*-((*Z*)-3-Methyl-4-(2-(trifluoromethyl)benzyl)-1,2,4-thiadiazol-5(4*H*)-ylidene)-*N′*-phenylbenzimidamide (**26**)

*N*-(2-(Trifluoromethyl)benzyl)-2,3-diphenyl-1,2,4-thiadiazol-5(2*H*)-imine hydrobromide **64b** (375 mg, 0.76 mmol), ACN **79** (3.98 mL, 76 mmol), and TEA (159 μL, 1.14 mmol) were reacted according to general procedure A. The crude product was purified by flash silica gel chromatography (PE/EtOAc in 98/2 ratio) and washed with *n*-hexane to achieve the final compound **26** as a yellow solid (251 mg, yield 73%). ^1^H NMR (401 MHz, DMSO-*d*_6_) δ 7.85 (d, *J* = 7.8 Hz, 1H), 7.66 (t, *J* = 7.7 Hz, 1H), 7.54 (t, *J* = 7.7 Hz, 1H), 7.34–7.27 (m, 3H), 7.27–7.20 (m, 4H), 7.02 (t, *J* = 7.4 Hz, 1H), 6.88 (d, *J* = 7.8 Hz, 1H), 6.76 (dt, *J* = 7.4, 1.1 Hz, 2H), 5.70 (s, 2H), 2.37 (s, 3H) ppm. ^13^C NMR (101 MHz, DMSO-*d*_6_) δ 170.35, 158.28, 153.57, 146.69, 134.34, 133.89 (q, *J*_CF_ = 1.5 Hz), 133.52, 129.57, 129.16 (2C), 128.95 (2C), 128.12, 127.92 (2C), 126.43 (q, *J*_CF_ = 5.9 Hz), 126.22, 126.13, 125.76, 123.04, 121.93 (2C), 45.54, 15.82 ppm. Rt = 4.54 min (apolar method); ESI-MS for C_24_H_19_F_3_N_4_S: calculated 452.13, found *m*/*z* 453.17 [M + H]^+^; UPLC-MS purity (UV 215 nm) >99.5%.

##### 4.1.12.26 (*E*)-*N*-((*Z*)-4-(2-Methoxybenzyl)-3-methyl-1,2,4-thiadiazol-5(4*H*)-ylidene)-*N′*-phenylbenzimidamide (**27**)

*N*-(2-Methoxybenzyl)-2,3-diphenyl-1,2,4-thiadiazol-5(2*H*)-imine hydrobromide **65b** (235 mg, 0.52 mmol), ACN **79** (2.716 mL, 52 mmol), and TEA (108 μL, 0.78 mmol) were reacted according to general procedure A. The crude product was purified by flash silica gel chromatography (PE/EtOAc in 9/1 ratio) and washed with *n*-hexane to achieve the final compound **27** as a shiny yellow solid (97 mg, yield 45%). ^1^H NMR (401 MHz, DMSO-*d*_6_) δ 7.38–7.26 (m, 6H), 7.25–7.20 (m, 2H), 7.08 (dd, *J* = 8.4, 1.0 Hz, 1H), 7.00 (tt, *J* = 7.3, 1.3 Hz, 1H), 6.93 (td, *J* = 7.4, 1.0 Hz, 1H), 6.87 (dd, *J* = 7.6, 1.8 Hz, 1H), 6.76–6.71 (m, 2H), 5.45 (s, 2H), 3.87 (s, 3H), 2.41 (s, 3H) ppm. ^13^C NMR (101 MHz, DMSO-*d*_6_) δ 170.47, 158.46, 156.48, 153.84, 146.79, 134.70, 129.38, 129.15 (2C), 128.97, 128.85 (2C), 127.90 (2C), 127.15, 123.10, 122.91, 121.96 (2C), 120.48, 111.05, 55.46, 44.43, 15.87 ppm. Rt = 4.26 min (apolar method); ESI-MS for C_24_H_22_N_4_OS: calculated 414.15, found *m*/*z* 415.11 [M + H]^+^; UPLC-MS purity (UV 215 nm) 99%.

##### 4.1.12.27 (*E*)-*N*-((*Z*)-4-(2-Chlorobenzyl)-3-methyl-1,2,4-thiadiazol-5(4*H*)-ylidene)-*N′*-phenylbenzimidamide (**28**)

*N*-(2-Chlorobenzyl)-2,3-diphenyl-1,2,4-thiadiazol-5(2*H*)-imine hydrobromide **66b** (323 mg, 0.7 mmol), ACN **79** (3.656 mL, 70 mmol), and TEA (147 μL, 1.1 mmol) were reacted according to general procedure A. The crude product was purified by flash silica gel chromatography (PE/EtOAc in 9/1 ratio) and washed with *n*-hexane to achieve the final compound **28** as a shiny yellow solid (102 mg, yield 35%). ^1^H NMR (401 MHz, DMSO-*d*_6_) δ 7.56 (dd, *J* = 7.2, 2.0 Hz, 1H), 7.39–7.28 (m, 5H), 7.24 (q, *J* = 7.4 Hz, 4H), 7.01 (t, *J* = 7.4 Hz, 1H), 6.86 (dd, *J* = 7.0, 2.3 Hz, 1H), 6.75 (d, *J* = 7.8 Hz, 2H), 5.60 (s, 2H), 2.39 (s, 3H) ppm. ^13^C NMR (101 MHz, DMSO-*d*_6_) δ 170.31, 158.37, 153.57, 146.69, 134.46, 132.93, 131.54, 129.70, 129.51, 129.33, 129.17 (2C), 128.91 (2C), 127.93 (2C), 127.90, 126.98, 123.06, 121.96 (2C), 46.47, 15.92 ppm. Rt = 4.74 min (apolar method); ESI-MS for C_23_H_19_ClN_4_S: calculated 418.10, found *m*/*z* 419.11/421.11 [M + H]^+^; UPLC-MS purity (UV 215 nm) 96%.

##### 4.1.12.28 (*E*)-*N*-((*Z*)-4-(2-Aminobenzyl)-3-methyl-1,2,4-thiadiazol-5(4*H*)-ylidene)-*N′*-phenylbenzimidamide (**29**)

*N*-(2-Aminobenzyl)-2,3-diphenyl-1,2,4-thiadiazol-5(2*H*)-imine dihydrobromide **67b** (1.222 g, 2.35 mmol), ACN **79** (12.273 mL, 235 mmol), and TEA (820 μL, 5.88 mmol) were reacted according to general procedure A. The crude product was purified by flash silica gel chromatography (PE/EtOAc in 83/17 ratio) and washed with *n*-hexane to achieve the final compound **29** as a shiny yellow solid (195 mg, yield 21%). ^1^H NMR (401 MHz, DMSO-*d*_6_) δ 7.44–7.39 (m, 2H), 7.37–7.26 (m, 3H), 7.24 (t, *J* = 7.6 Hz, 3H), 7.01 (t, *J* = 7.5 Hz, 2H), 6.76 (d, *J* = 7.6 Hz, 3H), 6.70 (d, *J* = 7.8 Hz, 2H), 6.54 (t, *J* = 7.4 Hz, 1H), 5.42 (s, 2H), 5.40 (s, 2H), 2.38 (s, 3H) ppm. ^13^C NMR (101 MHz, DMSO-*d*_6_) δ 170.97, 158.44, 154.11, 146.70, 146.25, 134.65, 129.50, 129.11 (2C), 128.92 (2C), 128.51, 128.04 (2C), 127.32, 123.04, 122.05 (2C9, 118.07, 116.23, 115.33, 45.91, 16.28 ppm. Rt = 5.70 min (generic method); ESI-MS for C_23_H_21_N_5_S: calculated 399.15, found *m*/*z* 400.14 [M + H]^+^; UPLC-MS purity (UV 215 nm) 98%.

##### 4.1.12.29 (*E*)-*N*-((*Z*)-4-Benzyl-3-methyl-1,2,4-thiadiazol-5(4*H*)-ylidene)-4-methoxy-*N′*-phenylbenzimidamide (**30**)

*N*-Benzyl-3-(4-methoxyphenyl)-2-phenyl-1,2,4-thiadiazol-5(2*H*)-imine hydrobromide **68b** (300 mg, 0.66 mmol), ACN **79** (3.4 mL, 66 mmol), and TEA (138 μL, 0.99 mmol) were reacted according to general procedure A. The crude product was purified by recrystallization from EtOAc to achieve the final compound **30** as a yellow solid (100 mg, yield 37%). ^1^H NMR (401 MHz, DMSO-*d*_6_) δ 7.46–7.40 (m, 2H), 7.40–7.36 (m, 2H), 7.32 (d, *J* = 7.2 Hz, 3H), 7.26 (t, *J* = 7.8 Hz, 2H), 7.02 (td, *J* = 7.4, 1.3 Hz, 1H), 6.86–6.80 (m, 2H), 6.80–6.75 (m, 2H), 5.56 (s, 2H), 3.72 (s, 3H), 2.40 (s, 3H) ppm. ^13^C NMR (101 MHz, DMSO-*d*_6_) δ 170.37, 160.17, 157.56, 153.49, 147.09, 135.98, 131.13 (2C), 129.01 (2C), 128.87 (2C), 127.75, 126.98 (2C), 126.56, 122.86, 121.83 (2C), 113.33 (2C), 55.16, 48.51, 16.22 ppm. Rt = 3.86 min (apolar method); ESI-MS for C_24_H_22_N_4_OS: calculated 414.15, found *m*/*z* 415.10 [M + H]^+^; UPLC-MS purity (UV 215 nm) >99.5%.

##### 4.1.12.30 (*E*)-*N*-((*Z*)-4-Benzyl-3-methyl-1,2,4-thiadiazol-5(4*H*)-ylidene)-3-methoxy-*N′*-phenylbenzimidamide (**31**)

*N*-Benzyl-3-(3-methoxyphenyl)-2-phenyl-1,2,4-thiadiazol-5(2*H*)-imine hydrobromide **69b** (417 mg, 0.84 mmol), ACN **79** (4.387 mL, 84 mmol), and TEA (176 μL, 1.26 mmol) were reacted according to general procedure A. The crude product was purified by flash silica gel chromatography (PE/EtOAc in 85/15 ratio) and further recrystallized from EtOAc to achieve the final compound **31** as a yellow solid (105 mg, yield 30%). ^1^H NMR (401 MHz, DMSO-*d*_6_) δ 7.42–7.36 (m, 2H), 7.35–7.29 (m, 3H), 7.28–7.21 (m, 2H), 7.19 (t, *J* = 7.9 Hz, 1H), 7.05–6.98 (m, 2H), 6.95 (dd, *J* = 2.6, 1.5 Hz, 1H), 6.90 (ddd, *J* = 8.2, 2.7, 1.0 Hz, 1H), 6.79–6.74 (m, 2H), 5.55 (s, 2H), 3.57 (s, 3H), 2.42 (s, 3H) ppm. ^13^C NMR (101 MHz, DMSO-*d*_6_) δ 170.63, 158.58, 158.14, 153.68, 146.89, 135.90, 135.88, 129.05, 128.88 (2C), 128.84 (2C), 127.75, 126.98 (2C), 122.97, 121.90 (2C), 121.53, 115.22, 114.71, 54.81, 48.56, 16.22 ppm. Rt = 3.91 min (apolar method); ESI-MS for C_24_H_22_N_4_OS: calculated 414.15, found *m*/*z* 415.25 [M + H]^+^; UPLC-MS purity (UV 215 nm) 99.5%.

##### 4.1.12.31 (*E*)-*N*-((*Z*)-4-Benzyl-3-methyl-1,2,4-thiadiazol-5(4*H*)-ylidene)-3-hydroxy-*N′*-phenylbenzimidamide (**32**)

(*E*)-*N*-((*Z*)-4-Benzyl-3-methyl-1,2,4-thiadiazol-5(4*H*)-ylidene)-3-methoxy-*N′*-phenylbenzimidamide **31** (258 mg, 0.63 mmol) and 3 equiv of BBr_3_ (1.9 mmol) were reacted according to the general procedure B. The crude material was purified via flash silica gel column chromatography (PE/EtOAc in 8/2 ratio). The product was further recrystallized from a mixture of EtOAc/*n*-hexane (9/1), then washed with *n*-hexane, and filtered to give the final compound **32** as a light yellow solid (58 mg, yield 23%). ^1^H NMR (401 MHz, DMSO-*d*_6_) δ 9.46 (s, 1H), 7.40 (ddt, *J* = 7.8, 6.3, 1.0 Hz, 2H), 7.32 (td, *J* = 6.6, 1.5 Hz, 3H), 7.27–7.20 (m, 2H), 7.08–6.98 (m, 2H), 6.91 (dd, *J* = 2.6, 1.5 Hz, 1H), 6.80–6.69 (m, 4H), 5.55 (s, 2H), 2.39 (s, 3H) ppm. ^13^C NMR (101 MHz, DMSO-*d*_6_) δ 170.58, 158.48, 156.91, 153.59, 146.70, 135.98, 135.87, 128.90 (2C), 128.88, 128.86 (2C), 127.78, 126.94 (2C), 122.93, 121.92 (2C), 119.96, 116.46, 116.13, 48.45, 16.20 ppm. Rt = 5.17 min (generic method); ESI-MS for C_23_H_20_N_4_OS: calculated 400.14, found *m*/*z* 401.08 [M + H]^+^, 399.18 [M – H]^−^; UPLC-MS purity (UV 215 nm) >99.5%.

##### 4.1.12.32 (*E*)-*N*-((*Z*)-4-Benzyl-3-methyl-1,2,4-thiadiazol-5(4*H*)-ylidene)-2-methoxy-*N′*-phenylbenzimidamide (**33**)

*N*-Benzyl-3-(2-methoxyphenyl)-2-phenyl-1,2,4-thiadiazol-5(2*H*)-imine hydrobromide **70b** (290 mg, 0.59 mmol), ACN **79** (3 mL, 59 mmol), and TEA (123 μL, 0.88 mmol) were reacted according to general procedure A. The crude product was purified by flash silica gel chromatography (PE/EtOAc in 84/16 ratio) and further recrystallized from EtOAc to achieve the final compound **33** as a yellow solid (92 mg, yield 38%). ^1^H NMR (401 MHz, DMSO-*d*_6_) δ 7.42–7.36 (m, 2H), 7.35–7.25 (m, 4H), 7.23 (dd, *J* = 7.5, 1.8 Hz, 1H), 7.17–7.10 (m, 2H), 6.95–6.86 (m, 3H), 6.71–6.66 (m, 2H), 5.48 (s, 2H), 3.40 (s, 3H), 2.39 (s, 3H) ppm. ^13^C NMR (101 MHz, DMSO-*d*_6_) δ 170.07, 158.61, 155.63, 153.51, 146.95, 135.87, 130.30, 129.58, 128.84 (2C), 128.21 (2C), 127.78, 127.03 (2C), 125.68, 122.94, 121.66 (2C), 120.22, 111.50, 54.95, 48.31, 16.20 ppm. Rt = 5.84 min (generic method); ESI-MS for C_24_H_22_N_4_OS: calculated 414.15, found *m*/*z* 415.27 [M + H]^+^; UPLC-MS purity (UV 215 nm) >99.5%.

##### 4.1.12.33 (*E*)-*N*-((*Z*)-4-Benzyl-3-methyl-1,2,4-thiadiazol-5(4*H*)-ylidene)-*N′*-phenylisonicotinimidamide (**34**)

*N*-Benzyl-2-phenyl-3-(pyridin-4-yl)-1,2,4-thiadiazol-5(2*H*)-imine hydrobromide **71b** (362 mg, 0.85 mmol), ACN **79** (4.439 mL, 85 mmol), and TEA (297 μL, 2.13 mmol) were reacted according to general procedure A. The crude product was purified by flash silica gel chromatography (DCM/MeOH in 99/1 ratio) to achieve the final compound **34** as a yellow solid (230 mg, yield 60%). ^1^H NMR (401 MHz, DMSO-*d*_6_) δ 8.53 (dd, *J* = 6.0, 1.6 Hz, 2H), 7.43–7.36 (m, 2H), 7.35–7.28 (m, 5H), 7.25 (td, *J* = 8.1, 7.6, 2.0 Hz, 2H), 7.04 (tt, *J* = 7.4, 1.2 Hz, 1H), 6.77 (dd, *J* = 7.3, 1.1 Hz, 2H), 5.56 (s, 2H), 2.43 (s, 3H) ppm. ^13^C NMR (101 MHz, DMSO-*d*_6_) δ 171.24, 156.91, 154.09, 149.71 (2C), 146.33, 142.14, 135.68, 129.01 (2C), 128.90 (2C), 127.83, 127.04 (2C), 123.48, 123.28 (2C), 121.95 (2C), 48.62, 16.25 ppm. Rt = 5.14 min (generic method); ESI-MS for C_22_H_19_N_5_S: calculated 385.14, found *m*/*z* 386.16 [M + H]^+^; UPLC-MS purity (UV 215 nm) >99.5%.

##### 4.1.12.34 (*E*)-*N*-((*Z*)-4-Benzyl-3-methyl-1,2,4-thiadiazol-5(4*H*)-ylidene)-*N′*-phenylnicotinimidamide (**35**)

*N*-Benzyl-2-phenyl-3-(pyridin-3-yl)-1,2,4-thiadiazol-5(2*H*)-imine hydrobromide **72b** (238 mg, 0.56 mmol), ACN **79** (2.925 mL, 56 mmol), and TEA (195 μL, 1.4 mmol) were reacted according to general procedure A. The crude product was purified by flash silica gel chromatography (DCM/MeOH in 99/1 ratio) to achieve the final compound **35** as a yellow solid (114 mg, yield 53%). ^1^H NMR (401 MHz, DMSO-*d*_6_) δ 8.58 (d, *J* = 2.0 Hz, 1H), 8.50 (dd, *J* = 4.8, 1.7 Hz, 1H), 7.75 (dt, *J* = 8.0, 2.0 Hz, 1H), 7.43–7.36 (m, 2H), 7.36–7.29 (m, 4H), 7.29–7.22 (m, 2H), 7.04 (tt, *J* = 7.3, 1.1, 0.6 Hz, 1H), 6.82–6.77 (m, 2H), 5.57 (s, 2H), 2.43 (s, 3H) ppm. ^13^C NMR (101 MHz, DMSO-*d*_6_) δ 171.02, 156.63, 153.97, 150.00, 149.84, 146.62, 136.64, 135.78, 130.56, 129.07 (2C), 128.89 (2C), 127.81, 127.01 (2C), 123.32, 123.11, 122.07 (2C), 48.62, 16.24 ppm. Rt = 5.19 min (generic method); ESI-MS for C_22_H_19_N_5_S: calculated 385.14, found *m*/*z* 386.34 [M + H]^+^; UPLC-MS purity (UV 215 nm) >99.5%.

##### 4.1.12.35 (*E*)-*N*-((*Z*)-4-Benzyl-3-methyl-1,2,4-thiadiazol-5(4*H*)-ylidene)-*N′*-phenylfuran-2-carboximidamide (**36**)

*N*-Benzyl-3-(furan-2-yl)-2-phenyl-1,2,4-thiadiazol-5(2*H*)-imine hydrobromide **73b** (270 mg, 0.65 mmol), ACN **79** (3.4 mL, 65 mmol), and TEA (136 μL, 0.98 mmol) were reacted according to general procedure A. The crude product was purified by flash silica gel chromatography (PE/EtOAc in 92/8 ratio) to achieve the final compound **36** as a light brown solid (213 mg, yield 88%). ^1^H NMR (401 MHz, DMSO-*d*_6_) δ 7.68–7.63 (m, 1H), 7.44–7.28 (m, 7H), 7.10 (t, *J* = 7.4 Hz, 1H), 6.89–6.82 (m, 2H), 6.64–6.58 (m, 1H), 6.50 (dq, *J* = 3.4, 1.6 Hz, 1H), 5.56 (s, 2H), 2.41 (s, 3H) ppm. ^13^C NMR (101 MHz, DMSO-*d*_6_) δ 170.51, 153.75, 148.40, 148.11, 147.20, 144.98, 135.95, 128.88 (2C), 128.78 (2C), 127.80, 127.13 (2C), 123.19, 121.26 (2C), 115.66, 111.60, 48.47, 16.25 ppm. Rt = 3.42 min (apolar method); ESI-MS for C_21_H_18_N_4_OS: calculated 374.12, found *m*/*z* 375.04 [M + H]^+^, 373.01 [M – H]^−^; UPLC-MS purity (UV 215 nm) >99.5%.

##### 4.1.12.36 (*E*)-*N*-((*Z*)-4-Benzyl-3-methyl-1,2,4-thiadiazol-5(4*H*)-ylidene)-*N′*-(*m*-tolyl)benzimidamide (**37**)

*N*-Benzyl-3-phenyl-2-(*m*-tolyl)-1,2,4-thiadiazol-5(2*H*)-imine hydrobromide **74b** (118 mg, 0.3 mmol), ACN **79** (1.6 mL, 30 mmol), and TEA (63 μL, 0.45 mmol) were reacted according to general procedure A. The crude product was purified by flash silica gel chromatography (PE/EtOAc in 92/8 ratio) to achieve the final compound **37** as a shiny yellow solid (134 mg, yield 77%). ^1^H NMR (401 MHz, DMSO-*d*_6_) δ 7.47–7.36 (m, 4H), 7.36–7.25 (m, 6H), 7.09 (t, *J* = 7.7 Hz, 1H), 6.83 (d, *J* = 7.5 Hz, 1H), 6.65 (s, 1H), 6.49 (d, *J* = 7.8 Hz, 1H), 5.56 (s, 2H), 2.41 (s, 3H), 2.22 (s, 3H) ppm. ^13^C NMR (101 MHz, DMSO-*d*_6_) δ 170.56, 158.17, 153.62, 146.68, 138.15, 135.91, 134.69, 129.49, 129.21 (2C), 128.88, (2C) 128.69, 127.96 (2C), 127.77, 126.97 (2C), 123.75, 122.59, 118.96, 48.52, 20.98, 16.22 ppm. Rt = 4.48 min (apolar method); ESI-MS for C_24_H_22_N_4_S: calculated 398.16, found *m*/*z* 399.18 [M + H]^+^; UPLC-MS purity (UV 215 nm) >99.5%.

##### 4.1.12.37 (*E*)*-N′*-Benzyl-*N*-((*Z*)-4-benzyl-3-methyl-1,2,4-thiadiazol-5(4*H*)-ylidene)benzimidamide (**38**)

*N*,2-Dibenzyl-3-phenyl-1,2,4-thiadiazol-5(2*H*)-imine hydrobromide **75b** (580 mg, 1.3 mmol), ACN **79** (6.5 mL, 130 mmol), and TEA (272 μL, 1.95 mmol) were reacted according to general procedure A. The crude product was purified by flash silica gel chromatography (PE/EtOAc in 91/9 ratio) and further recrystallized from EtOAc to achieve the final compound **38** as a white solid (199 mg, yield 38%). ^1^H NMR (401 MHz, DMSO-*d*_6_) δ 7.50–7.42 (m, 5H), 7.40–7.28 (m, 7H), 7.29–7.21 (m, 3H), 5.44 (s, 2H), 4.80 (s, 2H), 2.33 (s, 3H) ppm. ^13^C NMR (101 MHz, DMSO-d_6_) δ 168.55, 161.48, 152.98, 140.87, 136.02, 135.52, 129.11, 128.81 (2C), 128.27 (4C), 127.95 (2C), 127.65, 127.45 (2C), 126.84 (2C), 126.48, 53.53, 48.09, 16.14 ppm. Rt = 3.47 min (apolar method); ESI-MS for C_24_H_22_N_4_S: calculated 398.16, found *m*/*z* 399.33 [M + H]^+^; UPLC-MS purity (UV 215 nm) >99.5%.

##### 4.1.12.38 (*E*)-*N*-((*Z*)-4-Benzyl-3-methyl-1,2,4-thiadiazol-5(4*H*)-ylidene)-*N′*-cyclohexylbenzimidamide (**39**)

*N*-Benzyl-2-cyclohexyl-3-phenyl-1,2,4-thiadiazol-5(2*H*)-imine hydrobromide **76b** (192 mg, 0.45 mmol), ACN **79** (2.35 mL, 45 mmol), and TEA (94 μL, 0.67 mmol) were reacted according to general procedure A. The crude product was purified by flash silica gel chromatography (PE/EtOAc in 92/8 ratio) and further recrystallized from EtOAc and washed with *n*-hexane to achieve the final compound **39** as a white crystalline solid (65 mg, yield 37%). ^1^H NMR (401 MHz, DMSO-*d*_6_) δ 7.49–7.39 (m, 3H), 7.38–7.32 (m, 4H), 7.32–7.20 (m, 3H), 5.40 (s, 2H), 3.52 (tt, *J* = 13.0, 9.0, 3.5 Hz, 1H), 2.32 (s, 3H), 1.80–1.67 (m, 4H), 1.62–1.50 (m, 3H), 1.37–1.13 (m, 3H) ppm. ^13^C NMR (101 MHz, DMSO-d_6_) δ 167.77, 158.98, 152.62, 136.22, 136.10, 128.78 (2C), 128.66, 128.26 (2C), 127.61, 127.55 (2C), 126.84 (2C), 57.70, 48.00, 35.43 (2C), 25.32, 24.22 (2C), 16.11 ppm. Impossible to assign a Rt due broadening of the main peak; ESI-MS for C_23_H_26_N_4_S: calculated 390.19, found *m*/*z* 391.33 [M + H]^+^.

##### 4.1.12.39 (*E*)-*N*-((*Z*)-4-Benzyl-3-methyl-1,2,4-thiadiazol-5(4*H*)-ylidene)-*N′*-(pyridin-4-yl)benzimidamide (**40**)

*N*-Benzyl-3-phenyl-2-(pyridin-4-yl)-1,2,4-thiadiazol-5(*2H*)-imine hydrobromide **77b** (336 mg, 0.79 mmol), ACN **79** (4.126 mL, 79 mmol), and TEA (276 μL, 1.98 mmol) were reacted according to general procedure A. The crude product was purified by flash silica gel chromatography (DCM/MeOH in 99.5/0.5 ratio) to achieve the final compound **40** as a yellow solid (53 mg, yield 17%). ^1^H NMR (401 MHz, DMSO-*d*_6_) δ 8.33 (dd, *J* = 6.1, 3.1 Hz, 2H), 7.46–7.41 (m, 2H), 7.41–7.36 (m, 3H), 7.36–7.29 (m, 5H), 6.75 (dd, *J* = 6.2, 3.0 Hz, 2H), 5.58 (s, 2H), 2.44 (s, 3H) ppm. ^13^C NMR (101 MHz, DMSO-*d*_6_) δ 172.03, 159.92, 154.35, 154.04, 150.15 (2C), 135.64, 134.10, 130.00, 129.26 (2C), 128.91 (2C), 128.18 (2C), 127.85, 127.01 (2C), 117.41, 48.71, 16.27 ppm. Rt = 4.74 min (generic method); ESI-MS for C_22_H_19_N_5_S: calculated 385.14, found *m*/*z* 386.18 [M + H]^+^; UPLC-MS purity (UV 215 nm) 97%.

##### 4.1.12.40 (*E*)-*N*-((*Z*)-4-Benzyl-3-methyl-1,2,4-thiadiazol-5(4*H*)-ylidene)-*N′*-(pyridin-3-yl)benzimidamide (**41**)

*N*-Benzyl-3-phenyl-2-(pyridin-3-yl)-1,2,4-thiadiazol-5(2*H*)-imine hydrobromide **78b** (698 mg, 1.64 mmol), ACN **79** (8.565 mL, 164 mmol), and TEA (572 μL, 4.1 mmol) were reacted according to the general procedure. The crude product was purified by flash silica gel chromatography (DCM/MeOH in 99/1 ratio) to achieve the final compound **41** as a yellow solid (85 mg, yield 13%). ^1^H NMR (401 MHz, DMSO-*d*_6_) δ 8.20 (dd, *J* = 4.7, 1.6 Hz, 1H), 7.99 (dd, *J* = 2.6, 0.8 Hz, 1H), 7.43–7.37 (m, 4H), 7.37–7.29 (m, 6H), 7.26 (ddd, *J* = 8.1, 4.7, 0.8 Hz, 1H), 7.18 (ddd, *J* = 8.1, 2.5, 1.6 Hz, 1H), 5.57 (s, 2H), 2.43 (s, 3H) ppm. ^13^C NMR (101 MHz, DMSO-*d*_6_) δ 171.48, 160.38, 154.04, 143.87, 143.24, 143.07, 135.69, 134.45, 129.64, 129.23 (2C), 128.97, 128.87 (2C), 128.13 (2C), 127.79, 126.96 (2C), 123.64, 48.61, 16.22 ppm. Rt = 5.01 min (generic method); ESI-MS for C_22_H_19_N_5_S: 386.18 [M + H]^+^; UPLC-MS purity (UV 215 nm) 98%.

##### 4.1.12.41 (*Z*)-4-Benzyl-3-methyl-*N*-(pyridin-2-yl)-1,2,4-thiadiazol-5(4*H*)-imine (**42**)

2-(Benzylamino)-[1,2,4]thiadiazolo[2,3-*a*]pyridin-4-ium bromide **132b** (380 mg, 1.2 mmol), ACN **79** (7.5 mL, 120 mmol), and DiPEA (209 μL, 1.2 mmol) were reacted according to general procedure A. The crude product was purified by flash silica gel chromatography (PE/EtOAc in 9/1 ratio) to achieve the final compound **42** as a white solid (79 mg, yield 23%). ^1^H NMR (401 MHz, DMSO-*d*_6_) δ 8.47 (d, *J* = 4.9 Hz, 1H), 7.75 (td, *J* = 7.6, 1.9 Hz, 1H), 7.40–7.33 (m, 2H), 7.32–7.23 (m, 3H), 7.19 (d, *J* = 8.1 Hz, 1H), 7.04–6.96 (m, 1H), 5.45 (s, 2H), 2.33 (s, 3H) ppm. ^13^C NMR (101 MHz, DMSO-*d*_6_) δ 166.42, 157.30, 153.79, 145.26, 138.01, 136.17, 128.82 (2C), 127.59, 126.67 (2C), 119.30, 116.83, 47.71, 16.36 ppm. In agreement with that previously reported by Martinez *et al*.^45^ Rt = 4.95 min (generic method); ESI-MS for C_15_H_14_N_4_S: calculated 282.09, found *m*/*z* 283.1 [M + H]^+^; UPLC-MS purity (UV 215 nm) >99.5%.

##### 4.1.12.42 (*Z*)-4-Benzyl-*N*-(4-(2-methoxyethyl)pyridin-2-yl)-3-methyl-1,2,4-thiadiazol-5(4*H*)-imine (**43**)

2-(Benzylamino)-7-(2-methoxyethyl)-[1,2,4]thiadiazolo[2,3-*a*]pyridin-4-ium bromide **133b** (655 mg, 1.7 mmol), ACN **79** (8.878 mL, 170 mmol), and DiPEA (300 μL, 1.7 mmol) were reacted according to general procedure A. The crude product was purified by flash silica gel chromatography (PE/EtOAc in 8/2 ratio) to achieve the final compound **43** as a white solid (140 mg, yield 24%). ^1^H NMR (401 MHz, DMSO-*d*_6_) δ 8.34 (d, *J* = 5.3 Hz, 1H), 7.40–7.33 (m, 2H), 7.33–7.28 (m, 1H), 7.28–7.22 (m, 2H), 7.07 (s, 1H), 6.90 (dd, *J* = 5.4, 1.5 Hz, 1H), 5.44 (s, 2H), 3.57 (t, *J* = 6.5 Hz, 2H), 3.22 (s, 3H), 2.82 (t, *J* = 6.6 Hz, 2H), 2.32 (s, 3H) ppm. ^13^C NMR (101 MHz, DMSO-*d*6) δ 166.32, 157.36, 153.68, 150.31, 144.83, 136.18, 128.79 (2C), 127.54, 126.58 (2C), 119.27, 117.82, 71.47, 57.78, 47.64, 34.51, 16.31 ppm. Rt = 4.94 min (generic method); ESI-MS for C_18_H_20_N_4_OS: calculated 340.14, found *m*/*z* 341.05 [M + H]^+^; UPLC-MS purity (UV 215 nm) 99%.

##### 4.1.12.43 (*E*)-*N*-((*Z*)-3-Benzyl-5-methyl-1,3,4-thiadiazol-2(3*H*)-ylidene)-*N′*-phenylbenzimidamide (**44**)

3-Benzyl-5-methyl-1,3,4-thiadiazol-2(3*H*)-imine **134d** (500 mg, 2.43 mmol), *N*-phenylbenzimidoyl chloride **108b** (625 mg, 2.9 mmol), and pyridine (243 μL, 2.9 mmol) were reacted according to general procedure C. The crude product was purified by flash silica gel chromatography (PE/EtOAc in 98/2 ratio) to achieve the final compound **44** as a yellow solid (222 mg, yield 23%). ^1^H NMR (401 MHz, DMSO-*d*_6_) δ 7.43–7.34 (m, 6H), 7.29 (dq, *J* = 14.5, 7.7, 7.1 Hz, 4H), 7.18 (t, *J* = 7.7 Hz, 2H), 6.93 (t, *J* = 7.4 Hz, 1H), 6.68 (d, *J* = 7.7 Hz, 2H), 5.53 (s, 2H), 2.47 (s, 3H) ppm. ^13^C NMR (101 MHz, DMSO-*d*_6_) δ 159.75, 157.61, 153.72, 148.77, 136.38, 135.85, 129.25 (2C), 129.15, 128.76 (2C), 128.63 (2C), 128.00 (2C), 127.83 (2C), 127.75, 122.17, 121.65 (2C), 52.51, 15.75 ppm. Rt = 4.77 min (apolar method); ESI-MS for C_23_H_20_N_4_S: calculated 384.14, found *m*/*z* 385.21 [M + H]^+^; UPLC-MS purity (UV 215 nm) 96%.

##### 4.1.12.44 (*E*)-*N*-((*Z*)-3-Benzyl-1,3,4-thiadiazol-2(3*H*)-ylidene)-*N′*-phenylbenzimidamide (**45**)

3-Benzyl-1,3,4-thiadiazol-2(3*H*)-imine **135d** (363 mg, 1.9 mmol), *N*-phenylbenzimidoyl chloride **108b** (500 mg, 2.3 mmol), and pyridine (185 μL, 2.3 mmol) were reacted according to general procedure C. The crude product was purified by flash silica gel chromatography (PE/EtOAc in 97/3 ratio) to achieve the final compound **45** as a yellow solid (190 mg, yield 27%). ^1^H NMR (401 MHz, DMSO-*d*_6_) δ 8.78 (s, 1H), 7.43–7.34 (m, 6H), 7.34–7.24 (m, 4H), 7.18 (t, *J* = 7.8 Hz, 2H), 6.93 (t, *J* = 7.4 Hz, 1H), 6.69 (d, *J* = 7.7 Hz, 2H), 5.59 (s, 2H) ppm. ^13^C NMR (101 MHz, DMSO-*d*_6_) δ 158.58, 157.69, 148.75, 144.24, 136.22, 135.87, 129.23 (2C), 129.14, 128.77 (2C), 128.63 (2C), 128.05 (2C), 127.84 (2C), 127.81, 122.24, 121.63 (2C), 52.84, 16.07 ppm. Rt = 4.35 min (apolar method); ESI-MS for C_22_H_18_N_4_S: calculated 370.13, found *m*/*z* 371.15 [M + H]^+^, 369.43 [M – H]^−^; UPLC-MS purity (UV 215 nm) 97%.

##### 4.1.12.45 (*E*)-*N*-((*Z*)-3-Benzylthiazol-2(3*H*)-ylidene)-*N′*-phenylbenzimidamide (**46**)

3-Benzylthiazol-2(*3H*)-imine **136d** (790 mg, 4.1 mmol), *N*-phenyl benzimidoyl chloride **108b** (1.06 g, 4.9 mmol), and pyridine (395 μL, 4.9 mmol) were reacted according to general procedure C. The crude product was purified by flash silica gel chromatography (DCM/MeOH in 99.5/0.5 ratio) to achieve the final compound **46** as a light yellow solid (240 mg, yield 16%). ^1^H NMR (401 MHz, DMSO-*d*_6_) δ 7.60 (d, *J* = 3.3 Hz, 1H), 7.42–7.29 (m, 7H), 7.29–7.21 (m, 3H), 7.14 (t, *J* = 7.7 Hz, 2H), 6.87 (t, *J* = 7.5 Hz, 1H), 6.84 (d, *J* = 3.3 Hz, 1H), 6.64 (d, *J* = 7.7 Hz, 2H), 5.41 (s, 2H) ppm. ^13^C NMR (101 MHz, DMSO-*d*_6_) δ 149.51, 136.93 (2C), 136.65, 129.16 (2C), 128.60 (4C), 127.95 (2C), 127.68 (4C), 127.30, 121.72 (2C), 121.56, 107.91, 50.47 ppm. Rt = 5.08 min (generic method); ESI-MS for C_23_H_19_N_3_S: calculated 369.13, found *m*/*z* 370.11 [M + H]^+^; UPLC-MS purity (UV 215 nm) >99.5%.

### 4.2 Biology

#### 4.2.1 Fluorescence Assay for CFTR

Twenty-four hours after seeding on 96-well plates, CFBE41o^–^ cells stably expressing F508del-CFTR and the HS-YFP were treated with test compounds at the desired concentration, one compound per well. Cells treated with vehicle alone (DMSO) and with corrector VX-809 (1 μM), respectively, served as negative and positive controls. At the time of the assay, cells were washed with PBS containing (in μM) 137 NaCl, 2.7 KCl, 8.1 Na_2_HPO_4_, 1.5 KH_2_PO_4_, 1 CaCl_2_, and 0.5 MgCl_2_. Cells were then incubated for 25 min with 60 mL of PBS plus forskolin (20 μM) and VX-770 (1 μM) to maximally stimulate F508del-CFTR. Cells were then transferred to a microplate reader (FluoStar Galaxy; BMG Labtech, Offenburg, Germany) for CFTR activity determination. The plate reader was equipped with high-quality excitation (HQ500/20X: 500 ± 10 nm) and emission (HQ535/30M: 535 ± 15 nm) filters for YFP (Chroma Technology). Each assay comprised a continuous 14 s fluorescence reading and 2 s before and 12 s after injection of 165 mL of an iodide-containing solution (PBS with Cl^–^ replaced by I^–^; final I^–^ concentration 100 μM). Data were normalized to the initial background-subtracted fluorescence. To determine the I^–^ influx rate, the final 11 s of the data for each well was fitted with an exponential function to extrapolate initial slope (d*F*/d*t*). Reproducibility of results was confirmed by performing three independent experiments.

#### 4.2.2 Gene Silencing by siRNA Transfection

CFBE41o^–^ cells co-expressing F508del-CFTR and the HS-YFP were reverse-transfected with 50 nM (final concentration) siRNAs (Stealth, Life Technologies) in the presence of lipofectamine 2000 as a transfection agent. The following day, the medium was changed, and the cells were incubated at 37 °C for additional 24 h treated with test compounds at the desired concentration, prior to processing the cells for the YFP-based functional assay.

#### 4.2.3 CFTR Immunoprecipitation (IP) Assay

CFBE41o^–^ cells stably expressing wt- or F508del-CFTR were grown to confluence on 60 mm diameter dishes and treated for 24 h with the indicated compounds in the absence or in the presence of MG-132 (10 μM) in the last 4 h. CFTR immunoprecipitation was performed as previously reported.^40^ In brief, cells were lysed and, after centrifugation, the protein concentration in the supernatant was calculated using the BCA assay (Euroclone, Pero (MI), Italy) following supplier’s instructions An aliquot of supernatant corresponding to 600 μg of protein was incubated for 1 h with 2 μg/sample of rabbit polyclonal anti-CFTR antibody (Alomone Labs, Jerusalem, Israel), with rocking at room temperature (RT). Subsequently, the antibody–antigen mixture was precipitated with 25 μL/sample of Pierce Protein A/G Magnetic Beads (Thermo Fisher Scientific, Waltham, MA, USA) for 1 h rocking at RT, following manufacturer’s instructions. Immunoprecipitated proteins were eluted from the resin under reducing conditions with 100 μL of Laemli Sample Buffer 1× at RT. Equal amounts of IP products were analyzed by Western blotting (20 μL).

#### 4.2.4 Western Blot

CFBE41o^–^ cells were grown to confluence on 60 mm diameter dishes, treated for 24 h with the indicated compounds, lysed, and processed as previously reported.^40^ In brief, equal amounts of lysates (25 μg) or IP products (20 μL) were separated onto gradient 4–15% Criterion TGX Precast gels (Bio-rad laboratories Inc.), transferred to a nitrocellulose membrane with a Trans-Blot Turbo system (Bio-rad Laboratories Inc.), and analyzed by Western blotting. Proteins were detected using the following antibodies: mouse monoclonal anti-CFTR (cl.596; Cystic Fibrosis Therapeutics); mouse monoclonal anti-GAPDH (cl.6C5; Santa Cruz Biotechnology); mouse monoclonal anti-Ub (P4D1, Santa Cruz Biotechnology); or horseradish peroxidase (HRP)-conjugated anti-mouse IgG (Abcam). The proteins were then visualized by chemiluminescence using a SuperSignal West Femto Substrate (Thermo Scientific) and a Molecular Imager ChemiDoc XRS System. The molecular weight of the proteins (based on the Precision Plus Protein WesternC Standards, Bio-rad Laboratories Inc.) and the lane density profiles of ubiquitylated CFTR were calculated using the software Quantity one 4.6 of the Molecular Imager ChemiDoc XRS System.

#### 4.2.5 Proliferation Study

CFBE41o^–^ cells stably expressing F508del-CFTR and the HS-YFP were plated at a low density (5000 cell/well) on 96-well plates suitable for high-content imaging. After 6 h, cells were treated with different concentrations of test compounds or vehicle alone (DMSO). Cell proliferation was monitored (by exploiting the YFP signal to determine the area covered by cells) for 48 h using the Opera Phenix (PerkinElmer) high-content screening system. Alternatively, to monitor the cytotoxic effect of high concentrations of test compounds, CFBE41o^–^ cells were plated (10,000 cell/well) and treated (after 6 h) with test compounds or vehicle alone (DMSO). After 24 h, cells were counterstained with Hoechst 33342 and propidium iodide to visualize total cells and apoptotic cells, respectively, and imaged by using the Opera Phenix (PerkinElmer) high-content screening system. Data are expressed as means ± SEM, *n* = 6. Reproducibility of results was confirmed by performing three independent experiments. Statistical significance was tested by parametric ANOVA followed by the Dunnet multiple comparisons test.

#### 4.2.6 Labeling of Autophagic Vacuoles with Monodansylcadaverine (MDC)

CFBE41o^–^ cells stably expressing F508del-CFTR and the HS-YFP were plated (50,000 cells/well) on good-quality clear-bottom 96-well black microplates suitable for high-content imaging. After 24 h, cells were treated with test compounds or DMSO (as the negative control). In the last 6 h of incubation, SAR405 2 μM (autophagy inhibitor) and torin1 20 nM (autophagy activator) were added to the cells. After 24 h, cells were incubated with 50 μM MDC (Sigma-Aldrich) in PBS at 37 °C for 10 min.^62^ After incubation, cells were washed three times with PBS and immediately analyzed. High-content imaging and data analysis were performed using the Opera Phenix (PerkinElmer) high-content screening system. Wells were imaged in confocal mode using a 40× water-immersion objective. The MDC signal was laser-excited at 405 nm, and the emission wavelengths were collected between 435 and 550 nm. Data analysis of MDC spot number was performed using the Harmony software (ver 4.5) of the Opera Phenix high-content system. Data are expressed as means ± SEM, *n* = 3 independent experiments. Statistical significance was tested by parametric ANOVA followed by the Dunnet multiple comparisons test (all groups against the control group).

#### 4.2.7 RNF5 Expression and Purification

A construct coding for human RNF5 protein (aa 1–117) truncated to exclude the transmembrane domains, with an N-terminal GST-tag followed by the recognition site for TEV protease (pET vector backbone), was purchased from CliniSciences. A similar construct was recently described in the work of Ruan *et al*.^44^ The plasmid was first verified by sequencing and then transformed in BL21(DE3) Competent Cells (EC0114, Thermo Fischer Scientific). Overexpression of the protein was achieved in *Escherichia coli* by growing cells in LB medium at 37 °C to an OD_600_ ∼ 0.6 followed by induction with 0.2 mM isopropyl β-d-thiogalactopyranoside (IPTG) for 5 h at 37 °C. Bacterial pellets, obtained by centrifugation, were then stored at −80 °C until further processing. Once thawed, pellets were resuspended in lysis buffer [50 mM Tris–HCl pH 8, 1 mM EDTA, 1% Triton X-100, 5 mM DTT, 20 μM ZnCl_2_] supplemented with 200 μg/mL lysozyme, 10 μg/mL DNAse, 10 mM MgCl_2_, and protease inhibitor cocktail 1× (S8830, Sigma-Aldrich) and incubated for 45 min at 37 °C. Cell lysis was completed by sonication. The lysate was centrifuged, and the clear supernatant was incubated with Glutathione Sepharose 4 Fast Flow (FF) resin (17513202, Cytiva) for 1 h at RT. Resin was washed sequentially with lysis buffer and 50 mM Tris–HCl pH 8 and then eluted with GST elution buffer [50 mM Tris–HCl pH 8, 10 mM reduced glutathione]. Buffer was exchanged with PBS using a PD-10 desalting column (17085101, Cytiva), and TEV protease (T4455, Sigma-Aldrich) was added O/N at 4 °C to cleave the GST-tag. Undigested protein and soluble GST were removed by incubation with Glutathione Sepharose 4 FF resin for 1 h at RT. The unbound flow-through was further incubated with Ni-NTA agarose resin (30210, Qiagen) to remove the TEV protease, which contains a 6xHis-Tag. Finally, the unbound fraction was collected and concentrated using Amicon Ultra centrifugal filter devices with a cutoff of 3 kDa (Merck). Protein purity was verified by SDS-PAGE and blue Coomassie staining. Protein was either immediately used or stored at −80 °C in aliquots containing 5% glycerol.

#### 4.2.8 MicroScale Thermophoresis (MST) Experiments

Purified RNF5 was labeled using a Monolith protein labeling kit RED-NHS 2nd generation (NanoTemper Technologies, München, Germany) following manufacturer’s instructions. Labeled protein (5 nM) was first tested in a pre-test assay for optimal fluorescence, absence of aggregation, and sticking to capillaries using MST buffer [PBS, 0.05% Tween-20, 0.1% PEG-8000, 1 μM ZnCl_2_] with or without 1 mM DTT. Compounds were tested in binding check experiments at the maximum concentration allowed based on their solubility. 5% DMSO was tested as the maximum final concentration. MST experiments were performed at medium MST-power and 20% laser excitation on standard capillaries. Experiments were performed on a Monolith Pico-Red/Nano-Blue Instrument (NanoTemper Technologies, München, Germany).

#### 4.2.9 NMR Experiments

All the NMR experiments were recorded at 298 K using a 5 mm CryoProbe QCI ^1^H/^19^F–^13^C/^15^N–D quadruple resonance, a shielded *z*-gradient coil, and an automatic sample changer SampleJet NMR system with temperature control. The solubility of the compounds were evaluated by ^1^H 1D experiments and aggregation by WaterLOGSY, testing the compounds in buffer PBS pH 7,5, 1 μM ZnCl_2_, 1 mM DTT, 10% D_2_O (for the lock signal), and 1% DMSO-*d*_6_ at the theoretical concentrations of 20, 50, and 100 μM in the presence of 200 μM 4-trifluoromethyl benzoic acid (internal reference). For all samples, a 1D ^1^H NMR experiment was recorded, the water suppression was obtained using the standard NOESY (nuclear Overhauser effect spectroscopy) preset Bruker pulse sequence, with 64k data points, a spectral width (sw) of 30 ppm, 64 scans, acquisition time (aq) of 1.835 s, a relaxation delay (d1) of 4 s, and a mixing time of 10 ms. The WaterLOGSY experiments were achieved with a 7.5 ms long 180° Gaussian-shaped pulse, aq 0.852 s, mixing time of 1.7 s, relaxation delay of 2 s, and 256 scans. The data were multiplied by an exponential window function with 1 Hz line broadening prior to Fourier transformation. For NMR binding experiments, the compounds were tested at 50 μM in buffer PBS pH 7.5, 1 μM ZnCl_2_, 1 mM DTT, 10% D_2_O (for the lock signal), and 1% DMSO-*d*_6_ in the absence and in the presence of 3 μM RNF5. WaterLOGSY experiments were recorded with the same parameters used in solubility assays, but with a higher number of scans (512); STD experiments were recorded with 128 scans, with two on (0.7 and 1 ppm) and one off (−50 ppm) resonance spectra, a Gaussian-shaped train pulse of 50 ms each was employed, with a total saturation time of the protein envelope of 5, 3, 2, 1.5, 1, or 0.5 s. A *T*_1ρ_ filter of 2 ms was employed in STD experiments to eliminate the background signals from the protein. The STD spectrum was obtained by subtraction of the on-resonance spectrum from the off-resonance spectrum. Subtraction was performed by phase cycling to minimize artifacts arising from magnet and temperature instabilities.

## Supporting Information Available

Computed octanol/water log *P* values and log *S* values; computational methods; experimental procedures of chemical intermediates **47a**–**78a**, **47b**–**78b**, **108a**–**117a**, **108b**–**117b**, **132a**, **132b**, **133a**, **133b**, **134b**–**136b**, **134c**–**136c**, and **134d**–**136d**; experimental procedures of *N′*-(pyridinyl)benzimidamides **84** and **85**; experimental procedures of isothiocyanates **89**–**96**; ^1^H and ^13^C NMR spectra of final compounds **1**–**46**; HPLC–MS analysis of selected final compounds **6**, **9**–**11**, **14**, **16**, **17**, **19**, **21**–**25**, **27**–**29**, and **34**; 1D NOE spectrum of compounds **10** and **44**; and HMBC spectrum of compound **44** (PDF)
Molecular formula strings (CSV)
**16** complex (PDB)
**inh-2** complex (PDB)
**Analog-1** complex (PDB)

## Supplementary Material

jm3c00608_si_002.csv

jm3c00608_si_003.pdb

jm3c00608_si_004.pdb

jm3c00608_si_005.pdb

## Abbreviations Used

CF: cystic fibrosis
CFBE41o^–^: F508del-CFTR human CF bronchial epithelial cell line
CFTR: cystic fibrosis transmembrane conductance regulator
F508del: deletion of a phenylalanine 508
HMBC: Heteronuclear Multiple Bond Correlation
HS-YFP: halide-sensitive yellow fluorescent protein
MDC: monodansylcadaverine
MST: MicroScale Thermophoresis
NOE: Nuclear Overhauser Effect
PM: plasma membrane
RNF5: RING finger protein 5
STD: Saturation-Transfer Difference
WaterLOGSY: ^1^H Water-Ligand Observed via Gradient Spectros
