# Supplementary material for: Innovative Strategy toward Mutant CFTR Rescue in Cystic Fibrosis: Design and Synthesis of Thiadiazole Inhibitors of the E3 Ligase RNF5
Source: J Med Chem. 2023 Jul 13;66(14):9797–822. doi: 10.1021/acs.jmedchem.3c00608 (PMC10388311; doi:10.1021/acs.jmedchem.3c00608)
Supplement: Supplementary file 1 — jm3c00608_si_001.pdf [file jm3c00608_si_001.pdf]

SUPPORTING INFORMATION

An Innovative Strategy Toward Mutant CFTR rescue in Cystic Fibrosis: Design and Synthesis of Thiadiazole Inhibitors of the E3 ligase RNF5

Irene Brusa,[a, b] Elvira Sondo,[c] Emanuela Pesce, [c] Valeria Tomati, [c] Dario Gioia,[b] Federico Falchi,[a, b] Beatrice Balboni,[a, b] Jose Antonio Ortega Martínez,[b] Marina Veronesi,[b] Elisa Romeo,[b] Natasha Margaroli,[b] Maurizio Recanatini,[a] Stefania Girotto,[b] Nicoletta Pedemonte,*[c] Marinella Roberti,*[a] and Andrea Cavalli [a, b, d]

[a]*Department of Pharmacy and Biotechnology, University of Bologna, 40126, Bologna, Italy.*

[b]*Computational & Chemical Biology, Istituto Italiano di Tecnologia, 16163, Genova, Italy.*

[c]*UOC Genetica Medica, IRCCS Istituto Giannina Gaslini, 16147, Genova, Italy.*

[d]*Centre Européen de Calcul Atomique et Moléculaire, EPFL CECAM, 1015 Lousanne, Switzerland.*

* To whom correspondence should be addressed (NP) e-mail: nicolettapedemonte@gaslini.org; (MR) e-mail: marinella.roberti@unibo.it

TABLE OF CONTENT

Table S1. Computed Octanol/Water log P's, logS's of **inh-2**, **analog-1** and compounds **1-46**. S3

Computational methods S4

Figure S1. Docking pose of **analog1** into RNF5 pocket S5

Figure S2. 1D NOE (DMSO-*d6*) spectrum of compound **10**. S6

Figure S3. 1D NOE (DMSO-*d6*) spectrum of compound **44**. S7

Figure S4. HMBC (DMSO-*d*6) spectrum of compound **44**. S8

Figure S5. MST analysis of compound **12** S9

Figure S6. Comparison of the binding modes of **inh-2** and **16** into RNF5 pocket S10

Experimental Procedures of Chemical Intermediates S11

Scheme S1: Synthesis of N'-(pyridinyl)benzimidamides **84** and **85** S41

Scheme S2: Synthesis of Isothiocyanates **89-96**. S42

^{1}H-NMR spectrum, ^{13}C-NMR spectrum of final compounds **1-46** S46

HPLC-MS analysis of selected analogues **6, 9-11, 14, 16, 17, 19, 21-25, 27-29** and **34** S92

References S125

Table S1. Computed Octanol/Water log P's, logS's (S in mol·dm^{-3}) of inh-2, analog-1 and compounds 1-46.

Molecule	MW	QPlogPo/w	QPlogS
**42**	282,362	4,043	-4,279
**43**	340,442	4,41	-4,777
**36**	374,459	5,471	-5,287
**41**	385,486	5,344	-5,65
**35**	385,486	5,344	-5,681
**40**	385,486	5,342	-5,7
**34**	385,486	5,345	-5,741
**32**	400,497	5,398	-5,827
**33**	414,524	6,131	-5,972
**16**	414,524	6,349	-6,196
**22**	427,566	6,421	-6,197
**2**	402,488	6,261	-6,216
**39**	390,545	6,198	-6,217
**20**	399,512	5,5	-6,293
**45**	370,471	6,29	-6,344
**21**	413,539	6,114	-6,368
**27**	414,524	6,412	-6,371
**29**	399,512	5,569	-6,371
**18**	442,578	6,612	-6,408
**13**	399,512	5,508	-6,429
**12**	428,508	5,677	-6,432
**17**	400,497	5,688	-6,447
**46**	369,483	6,762	-6,498
**8**	400,497	5,731	-6,608
**38**	398,525	6,529	-6,628
**inh-2**	384,498	6,386	-6,632
**19**	428,551	6,789	-6,645
**analog-1**	370,471	6,096	-6,695
**30**	414,524	6,454	-6,783
**31**	414,524	6,465	-6,83
**7**	414,524	6,49	-6,893
**23**	402,488	6,611	-6,918
**44**	384,498	6,664	-6,996
**9**	402,488	6,629	-7,027
**14**	398,525	6,709	-7,031
**5**	412,551	7,005	-7,08
**37**	398,525	6,689	-7,082
**1**	398,525	6,839	-7,093
**25**	398,525	6,694	-7,145
**6**	398,525	6,709	-7,216
**4**	398,525	6,85	-7,248
**22**	427,566	6,879	-7,265
**24**	418,943	6,892	-7,306
**28**	418,943	6,872	-7,362
**10**	418,943	6,894	-7,403
**11**	463,394	6,973	-7,522
**3**	412,551	7,189	-7,531
**26**	452,496	7,285	-7,994
**15**	452,496	7,369	-8,02

Computational methods

Docking studies have been performed on a RNF5 protein model obtained as described in a previous work.[1] The **inh-2** proposed binding mode has been considered as the reference for the subsequent optimization strategy and therefore, prior to proceed with the docking of the designed compounds, the ability of Glide[2] (Schrödinger Release 2018-2: Glide, Schrödinger, LLC, New York, NY, 2018) docking software in reproducing it has been evaluated.

The ligands were manually designed and then prepared for the subsequent studies using the LigPrep utility of the Schrödinger suite, selecting the specific stereoisomers and evaluating all the possible tautomers and ionization states at pH 7.0 ± 2.0 by means of the Epik software.[3]

Docking studies were run using the Glide SP scoring function, an enhanced sampling for the generation of ligand conformations, leaving the rest of parameters to their default values. Then, at least 5 binding modes for each ligand were visually inspected to evaluate their adherence to the proposed **inh-2** binding mode.

Figure S1. Docking pose of analog1 into RNF5 pocket

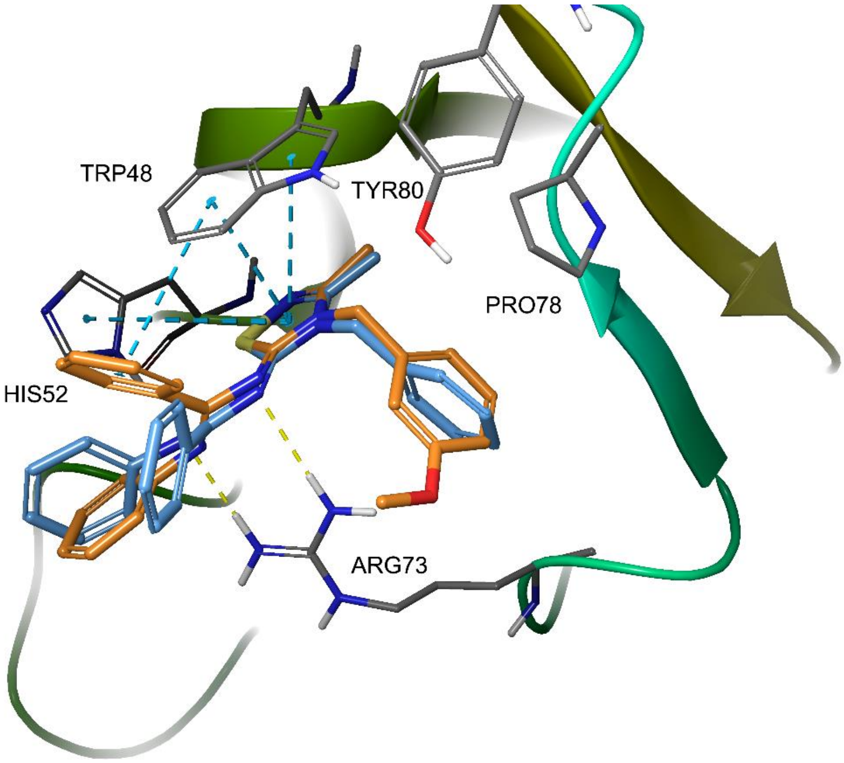

Figure S1. Docking pose of **analog-1** into the RNF5 pocket. With respect to the proposed binding mode of **inh-2** (see Figure 2), **analog-1** seems to lose at least two H-bond interactions, which might explain the loss of activity.

Figure S2. 1D NOE (DMSO-*d6*) spectrum of compound 10.[a]

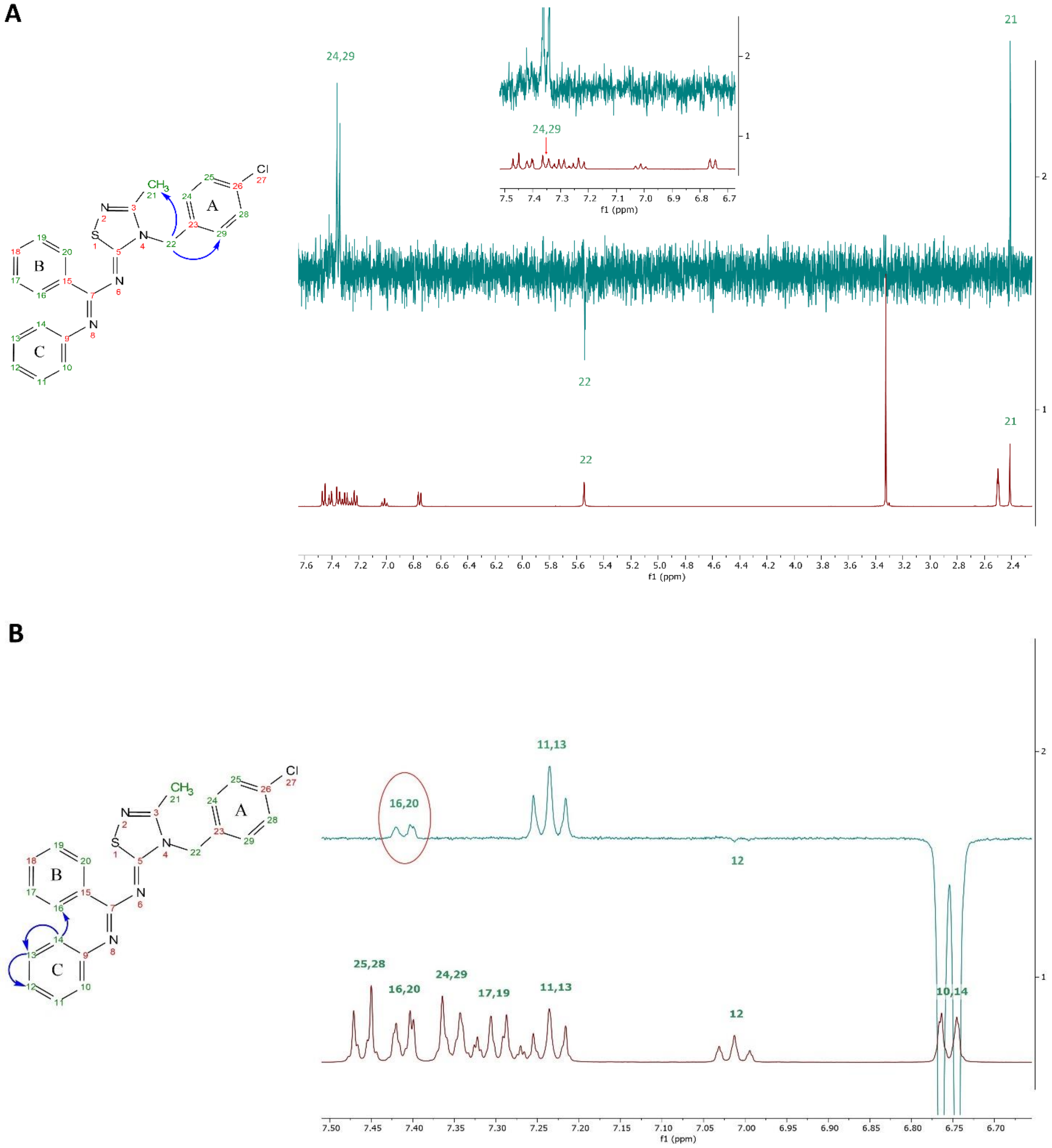

[a]A) selective irradiation of H-22 (δ_H 5.54 ppm); B) selective irradiation of of H-14,10 (δ_H 6.75 ppm).

Figure S3. 1D NOE (DMSO-*d6*) spectrum of compound 44.[a]

A

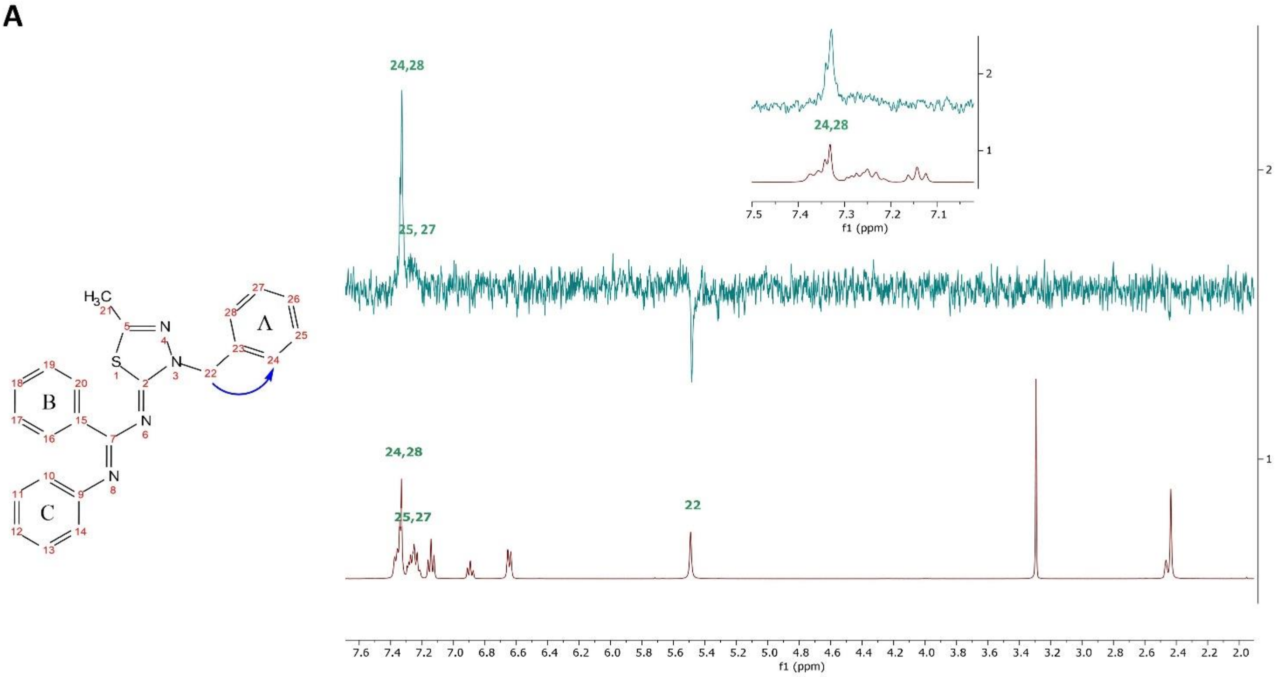

B

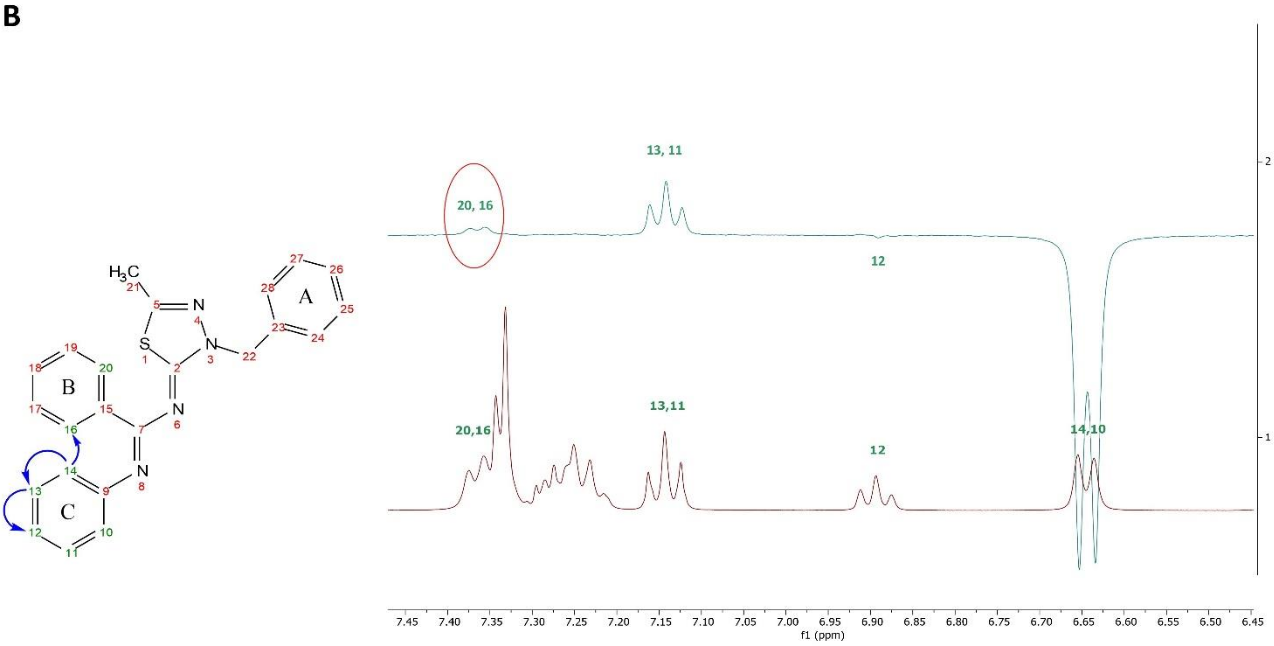

[a]A) selective irradiation of H-22 (δ_H 5.49 ppm); B) selective irradiation of H-14,10 (δ_H 6.65 ppm).

Figure S4. HMBC (DMSO-*d*6) spectrum of compound 44.[a]

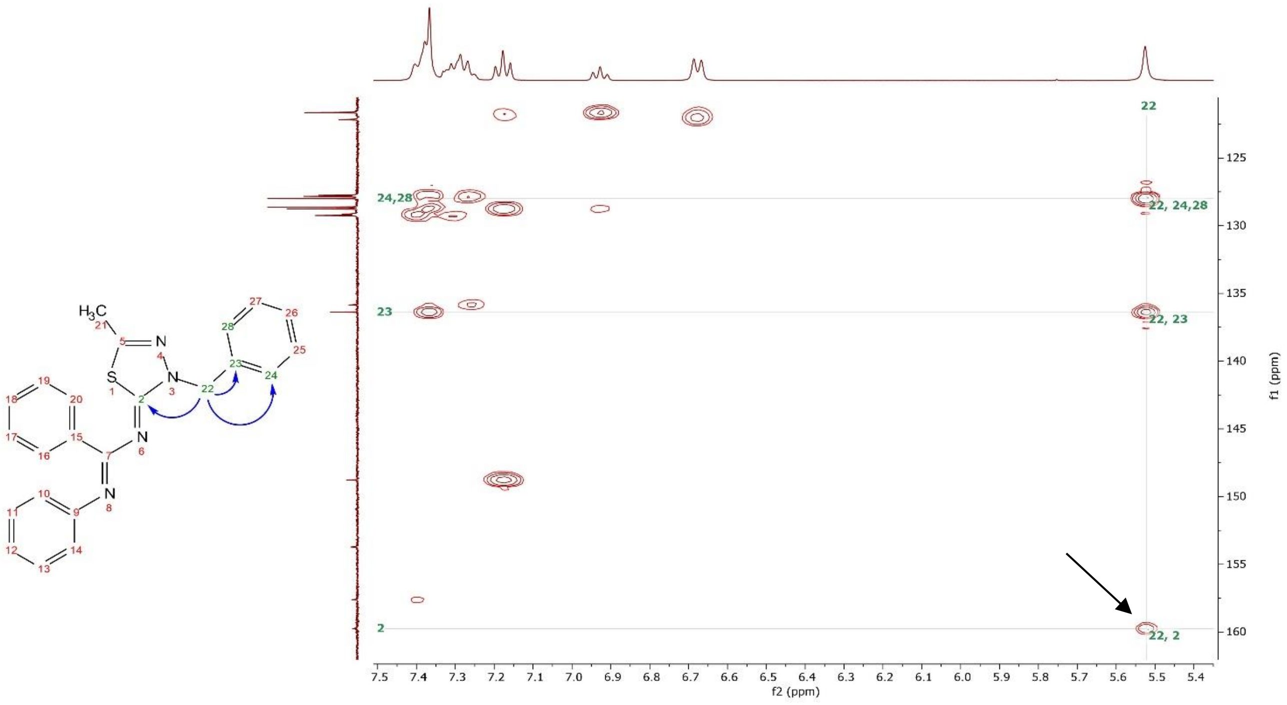

[a]Only the Interactions among aromatic and benzylic regions are shown.

Figure S5. MST analysis of compound 12

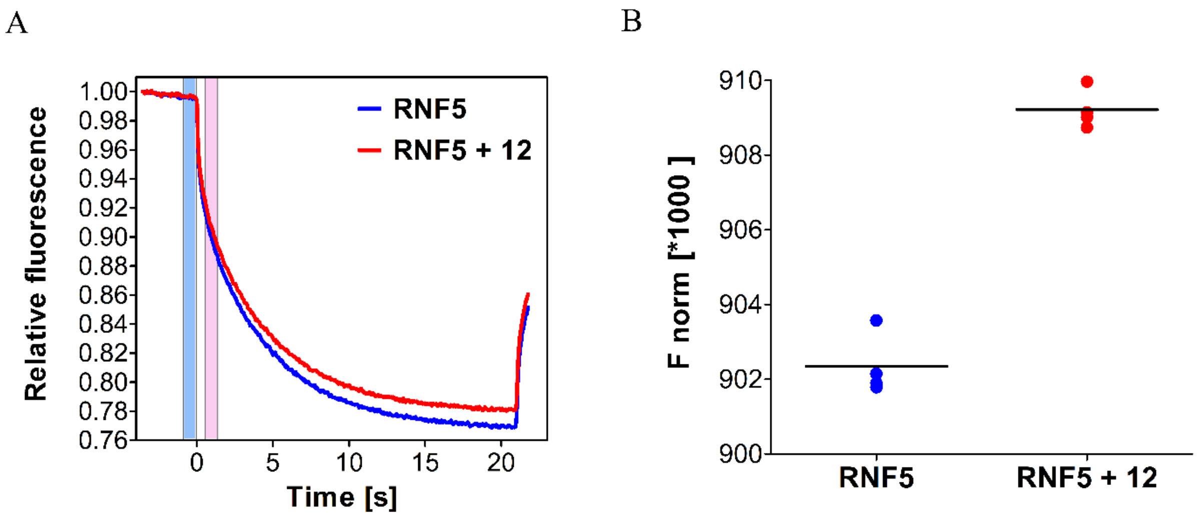

Figure S5. An example of binding check analysis by MST is reported: purified RNF5 (2.5 nM) is tested in the absence (blue) or presence (red) of compound **12** (100 μM). Data are reported as **A)** relative fluorescence of the labeled protein *vs* time (MST traces, where cold and hot regions are highlighted in light blue and pink, respectively) or **B**) normalized fluorescence, i.e. the relative fluorescence in the hot region (0.5 – 1.5 sec) normalized to the fluorescence in the cold region (-1 – 0 sec), multiplied by a 1000 factor (F norm *1000). The amplitude of the response and the signal-to-noise ratio indicate that compound **12** is binding to RNF5. A representative experiment of n=4 replicates is shown.

Figure S6. Comparison of the binding modes of inh-2 (cyan) and 16 (orange) into RNF5 pocket

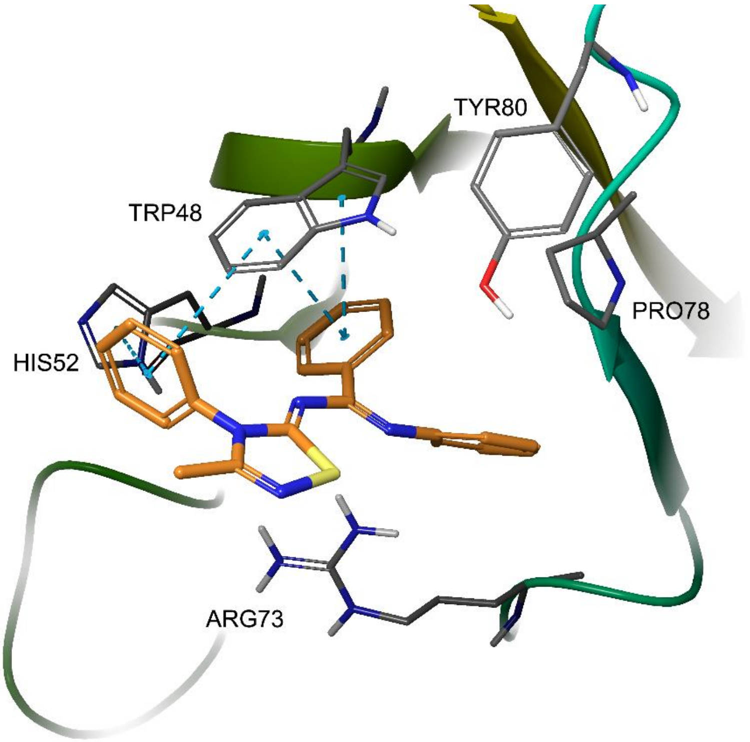

Figure S6. Comparison of the binding modes of **inh-2** (cyan) and **16** (orange) into RNF5 pocket. The dashed lines indicate the interactions of **16** with RNF5 residues (as in Figure 9). It appears that **16** assumes a more favorable pose with respect to **inh-2** (see interactions in Figure 2) probably due to the presence of the methoxy group.

Experimental Procedures of Chemical Intermediates

***N*-(benzylcarbamothioyl)-*N'*-phenylbenzimidamide (47a).**

N-phenylbenzimidoyl chloride **108a** (1 g, 4.6 mmol), NaNCS (413 mg, 5.1 mmol) and phenyl-methanamine **118** (556 µL, 5.1 mmol) were reacted according to general procedure D_2. The crude product was purified by flash silica gel chromatography (PE/EtOAc in 95/5 ratio) to achieve the intermediate imidoylthiourea **47a** as a white solid (926 mg, yield 62%). ^{1}H NMR (401 MHz, Chloroform-*d*) δ 12.39 (bs, 1H), 8.06 (bs, 1H), 7.42 (d, *J* = 7.6 Hz, 2H), 7.39 – 7.32 (m, 3H), 7.32 – 7.27 (m, 3H), 7.23 (d, *J* = 7.7 Hz, 2H), 7.11 (t, *J* = 7.6 Hz, 2H), 6.95 (t, *J* = 7.4 Hz, 1H), 6.62 (d, *J* = 7.7 Hz, 2H), 4.98 (d, *J* = 5.3 Hz, 2H) ppm.

***N*-benzyl-2,3-diphenyl-1,2,4-thiadiazol-5(2*H*)-imine hydrobromide (47b).**

N-(benzylcarbamothioyl)-*N'*-phenylbenzimidamide **47a** (1 g, 2.9 mmol) and bromine (224 µL, 4.3 mmol) were reacted according to general procedure E. The precipitate was washed with a mixture of PE/EtOAc (2/1 v/v) to achieve the intermediate hydrobromide iminothiadiazole **47b** as a light yellow solid (1.2 g, yield 97%). ^{1}H NMR (401 MHz, DMSO-d_6) δ 10.27 (t, *J* = 6.1 Hz, 1H), 7.62 (dd, *J* = 7.7, 2.0 Hz, 2H), 7.58 – 7.51 (m, 6H), 7.50 – 7.46 (m, 2H), 7.45 – 7.40 (m, 4H), 7.35 (tt, *J* = 7.2, 1.4 Hz, 1H), 4.97 (d, *J* = 6.0 Hz, 2H) ppm.

***N*-(phenethylcarbamothioyl)-*N'*-phenylbenzimidamide (48a)**.

(2-isothiocyanatoethyl)benzene **87** (300 µL, 2.0 mmol) and *N'*-phenylbenzimidamide **83** (393 mg, 2.0 mmol) were reacted according to general procedure D_1. The crude product was purified by flash silica gel chromatography (PE/EtOAc in 96/4 ratio) to achieve the intermediate imidoylthiourea **48a** as a light-yellow solid (270 mg, yield 38%). ^{1}H NMR (401 MHz, Chloroform-*d*) δ 11.95 (bs, 1H), 7.98 (bs, 1H), 7.36 – 7.31 (m, 1H), 7.30 – 7.16 (m, 9H), 7.15 – 7.09 (m, 2H), 6.99 – 6.94 (m, 1H), 6.59 – 6.52 (m, 2H), 4.00 (td, *J* = 7.0, 5.2 Hz, 2H), 3.02 (t, *J* = 7.0 Hz, 2H) ppm.

***N*-phenethyl-2,3-diphenyl-1,2,4-thiadiazol-5(2*H*)-imine hydrobromide (48b).**

N-(phenethylcarbamothioyl)-*N'*-phenylbenzimidamide **48a** (270 mg, 0.75 mmol) and bromine (76.9 µL, 1.5 mmol) were reacted according to general procedure E. The precipitate was washed with a mixture of PE/EtOAc (2/1 v/v) to achieve the intermediate hydrobromide iminothiadiazole **48b** as a white solid (222 mg, yield 68%). ^{1}H NMR (401 MHz, DMSO-d_6) δ 9.74 (t, *J* = 5.8 Hz, 1H), 7.61 – 7.49 (m, 8H), 7.46 – 7.40 (m, 2H), 7.36 (d, *J* = 4.4 Hz, 4H), 7.26 (ddd, *J* = 8.7, 4.9, 3.9 Hz, 1H), 3.99 (q, *J* = 6.9 Hz, 2H), 3.04 (t, *J* = 7.1 Hz, 2H) ppm.

***N'*-phenyl-*N*-((3-phenylpropyl)carbamothioyl)benzimidamide (49a)**.

(3-isothiocyanatopropyl)benzene **88** (414 µL, 2.5 mmol) and *N'*-phenylbenzimidamide **83** (491 mg, 2.5 mmol) were reacted according to general procedure D_1. The crude product was purified by flash silica gel chromatography (PE/EtOAc in 97/3 ratio) to achieve the intermediate imidoylthiourea **49a** as a yellow solid (473 mg, yield 51%). ^{1}H NMR (401 MHz, Chloroform-*d*) δ 12.06 (bs, 1H), 7.99 (bs, 1H), 7.37 – 7.32 (m, 1H), 7.28 (d, *J* = 12.1 Hz, 4H), 7.25 – 7.11 (m, 7H), 6.97 (t, *J* = 7.5 Hz, 1H), 6.69 (d, *J* = 7.7 Hz, 2H), 3.75 (q, *J* = 6.6 Hz, 2H), 2.75 (t, *J* = 7.7 Hz, 2H), 2.06 (p, *J* = 7.2 Hz, 2H) ppm.

2,3-diphenyl-*N*-(3-phenylpropyl)-1,2,4-thiadiazol-5(2*H*)-imine hydrobromide (49b).

N'-phenyl-*N*-((3-phenylpropyl)carbamothioyl)benzimidamide **49a** (466 mg, 1.25 mmol) and bromine (128 µL, 2.5 mmol) were reacted according to general procedure E. The precipitate was washed with a mixture of PE/EtOAc (2/1 v/v) to achieve the intermediate hydrobromide iminothiadiazole **49b** as a white solid (316 mg, yield 56%). ^{1}H NMR (401 MHz, DMSO-d_6) δ 10.04 (t, *J* = 5.7 Hz, 1H), 7.63 – 7.46 (m, 8H), 7.45 – 7.39 (m, 2H), 7.34 – 7.26 (m, 4H), 7.24 – 7.18 (m, 1H), 3.73 (q, *J* = 6.6 Hz, 2H), 2.75 (t, *J* = 7.6 Hz, 2H), 2.07 – 1.97 (m, 2H) ppm.

***N*-((4-methylbenzyl)carbamothioyl)-*N'*-phenylbenzimidamide (50a)**.

1-(isothiocyanatomethyl)-4-methylbenzene **89** (739 mg, 4.5 mmol) and *N'*-phenylbenzimidamide **83** (889 mg, 4.5 mmol) were reacted according to general procedure D_1. The crude product was purified by flash silica gel chromatography (PE/EtOAc in 96/4 ratio), which was recrystallized from a mixture of PE/EtOAc (9/1 v/v) to achieve the intermediate imidoylthiourea **50a** as a white crystalline solid (202 mg, yield 13%). ^{1}H NMR (401 MHz, Chloroform-*d*) δ 12.31 (bs, 1H), 8.04 (bs, 1H), 7.38 – 7.25 (m, 5H), 7.24 – 7.18 (m, 2H), 7.15 (d, *J* = 7.8 Hz, 2H), 7.10 (t, *J* = 7.8 Hz, 2H), 6.94 (td, *J* = 7.3, 1.2 Hz, 1H), 6.64 – 6.58 (m, 2H), 4.92 (d, *J* = 5.3 Hz, 2H), 2.33 (s, 3H) ppm.

***N*-(4-methylbenzyl)-2,3-diphenyl-1,2,4-thiadiazol-5(2*H*)-imine hydrobromide (50b).**

N-((4-methylbenzyl)carbamothioyl)-*N'*-phenylbenzimidamide **50a** (200 mg, 0.6 mmol) and bromine (57 µL, 1.1 mmol) were reacted according to general procedure E. The precipitate was washed with a mixture of PE/EtOAc (2/1 v/v) to achieve the intermediate hydrobromide iminothiadiazole **50b** as a white solid (223 mg, yield 85%). ^{1}H NMR (401 MHz, DMSO-d_6) δ 10.26 (t, *J* = 5.7 Hz, 1H), 7.61 (dd, *J* = 7.7, 2.0 Hz, 2H), 7.58 – 7.50 (m, 6H), 7.43 (dd, *J* = 8.4, 7.2 Hz, 2H), 7.36 (d, *J* = 8.0 Hz, 2H), 7.23 (d, *J* = 7.8 Hz, 2H), 4.92 (d, *J* = 5.4 Hz, 2H), 2.31 (s, 3H) ppm.

***N*-((4-methoxybenzyl)carbamothioyl)-*N'*-phenylbenzimidamide (51a)**.

1-(isothiocyanatomethyl)-4-methoxybenzene **90** (728 mg, 4.0 mmol) and N'-phenylbenzimidamide **83** (798 mg, 4.0 mmol) were reacted according to general procedure D_1. The crude product was purified by flash silica gel chromatography (PE/EtOAc in 96/4 ratio) to achieve the intermediate imidoylthiourea **51a** as a light-yellow solid (406 mg, yield 27%). ^{1}H NMR (401 MHz, Chloroform-*d*) δ 12.30 (bs, 1H), 8.05 (bs, 1H), 7.38 – 7.32 (m, 3H), 7.31 – 7.27 (m, 2H), 7.22 (d, *J* = 7.6 Hz, 2H), 7.11 (t, *J* = 7.7 Hz, 2H), 6.94 (t, *J* = 7.4 Hz, 1H), 6.90 – 6.86 (m, 2H), 6.61 (d, *J* = 7.7 Hz, 2H), 4.89 (d, *J* = 5.3 Hz, 2H), 3.80 (s, 3H) ppm.

***N*-(4-methoxybenzyl)-2,3-diphenyl-1,2,4-thiadiazol-5(2*H*)-imine hydrobromide (51b).**

N-((4-methoxybenzyl)carbamothioyl)-*N'*-phenylbenzimidamide **51a** (333 mg, 0.9 mmol) and bromine (69 µL, 1.35 mmol) were reacted according to general procedure E. The precipitate was washed with a mixture of PE/EtOAc (2/1 v/v) to achieve the intermediate hydrobromide iminothiadiazole **51b** as a white solid (311 mg, yield 76%). ^{1}H NMR (401 MHz, DMSO-d_6) δ 10.36 (t, *J* = 5.9 Hz, 1H), 7.63 – 7.58 (m, 2H), 7.58 – 7.51 (m, 6H), 7.45 – 7.39 (m, 4H), 6.98 (dt, *J* = 8.7, 2.9, 2.0 Hz, 2H), 4.89 (d, *J* = 5.9 Hz, 2H), 3.76 (s, 3H) ppm.

***N*-((4-fluorobenzyl)carbamothioyl)-*N'*-phenylbenzimidamide (52a)**.

1-fluoro-4-(isothiocyanatomethyl)benzene **91** (494 mg, 3.0 mmol) and N'-phenylbenzimidamide **83** (580 mg, 3.0 mmol) were reacted according to general procedure D_1. The crude product was purified by flash silica gel chromatography (PE/EtOAc in 96/4 ratio) to achieve the intermediate imidoylthiourea **52a** as a yellow solid (202 mg, yield 19%). ^{1}H NMR (401 MHz, Chloroform-*d*) δ 12.36 (bs, 1H), 8.07 (bs, 1H), 7.41 – 7.33 (m, 3H), 7.29 (d, *J* = 7.7 Hz, 2H), 7.24 – 7.18 (m, 2H), 7.10 (t, *J* = 7.8 Hz, 2H), 7.03 (t, *J* = 8.7 Hz, 2H), 6.94 (t, *J* = 7.5 Hz, 1H), 6.63 – 6.57 (m, 2H), 4.92 (d, *J* = 5.5 Hz, 2H) ppm.

***N*-(4-fluorobenzyl)-2,3-diphenyl-1,2,4-thiadiazol-5(2*H*)-imine hydrobromide (52b).**

N-((4-fluorobenzyl)carbamothioyl)-*N'*-phenylbenzimidamide **52a** (186 mg, 0.51 mmol) and bromine (52.6 µL, 1.02 mmol) were reacted according to general procedure E. The precipitate was washed with a mixture of PE/EtOAc (2/1 v/v) to achieve the intermediate hydrobromide iminothiadiazole **52b** as a light yellow solid (105 mg, yield 47%). ^{1}H NMR (401 MHz, DMSO-d_6) δ 10.23 (t, *J* = 6.1 Hz, 1H), 7.63 – 7.59 (m, 2H), 7.54 (ddt, *J* = 9.0, 5.7, 3.2 Hz, 8H), 7.43 (dd, *J* = 8.5, 7.2 Hz, 2H), 7.30 – 7.22 (m, 2H), 4.95 (d, *J* = 5.7 Hz, 2H) ppm.

***N*-((4-chlorobenzyl)carbamothioyl)-*N'*-phenylbenzimidamide (53a)**.

1-chloro-4-(isothiocyanatomethyl)benzene **92** (367 mg, 2.0 mmol) and *N'*-phenylbenzimidamide **83** (393 mg, 2.0 mmol) were reacted according to general procedure D_1. The crude product was purified by flash silica gel chromatography (PE/EtOAc in 97/3 ratio) to achieve the intermediate imidoylthiourea **53a** as a light-yellow solid (160 mg, yield 21%). ^{1}H NMR (401 MHz, Chloroform-*d*) δ 12.39 (bs, 1H), 8.08 (bs, 1H), 7.38 – 7.29 (m, 7H), 7.25 – 7.21 (m, 2H), 7.15 – 7.10 (m, 2H), 6.96 (t, *J* = 7.5 Hz, 1H), 6.63 (d, *J* = 7.8 Hz, 2H), 4.94 (d, *J* = 5.5 Hz, 2H) ppm.

***N*-(4-chlorobenzyl)-2,3-diphenyl-1,2,4-thiadiazol-5(2*H*)-imine hydrobromide (53b).**

N-((4-chlorobenzyl)carbamothioyl)-*N'*-phenylbenzimidamide **53a** (160 mg, 0.42 mmol) and bromine (43.2 µL, 0.84 mmol) were reacted according to general procedure E. The precipitate was washed with a mixture of PE/EtOAc (2/1 v/v) to achieve the intermediate hydrobromide iminothiadiazole **53b** as a yellow solid (87 mg, yield 45%). ^{1}H NMR (401 MHz, DMSO-d_6) δ 10.20 (t, *J* = 6.1 Hz, 1H), 7.61 (dt, *J* = 7.3, 1.6 Hz, 2H), 7.58 – 7.47 (m, 10H), 7.42 (dd, *J* = 8.4, 7.2 Hz, 2H), 4.96 (d, *J* = 5.8 Hz, 2H) ppm.

***N*-((4-bromobenzyl)carbamothioyl)-*N'*-phenylbenzimidamide (54a).**

N-phenylbenzimidoyl chloride **108b** (1.05 g, 4.9 mmol), NaNCS (440 mg, 5.4 mmol) and 4-bromo-benzylamine **119** (679 µL, 5.4 mmol) were reacted according to general procedure D_2. The crude product was purified by recrystallization from a mixture of EtOAc/DCM (95/5 v/v) to achieve the intermediate imidoylthiourea **54a** as a white crystalline solid (525 mg, yield 25%). ^{1}H NMR (401 MHz, Chloroform-*d*) δ 12.38 (bs, 1H), 8.09 (bs, 1H), 7.45 – 7.25 (m, 5H), 7.21 (t, *J* = 8.2 Hz, 1H), 7.12 (t, *J* = 7.7 Hz, 2H), 6.95 (t, *J* = 7.5 Hz, 1H), 6.85 (dd, *J* = 22.3, 7.8 Hz, 2H), 6.70 (s, 1H), 6.64 (d, *J* = 7.7 Hz, 2H), 4.97 (d, *J* = 5.0 Hz, 2H), 3.65 (s, 3H) ppm.

***N*-(4-bromobenzyl)-2,3-diphenyl-1,2,4-thiadiazol-5(*2H*)-imine hydrobromide (54b).**

N-((4-bromobenzyl)carbamothioyl)-*N'*-phenylbenzimidamide **54a** (525 mg, 1.23 mmol) and bromine (94 µL, 1.84 mmol) were reacted according to general procedure E. The precipitate was washed with a mixture of PE/EtOAc (2/1 v/v) to achieve the intermediate hydrobromide iminothiadiazole **54b** as a white solid (580 mg, yield 94%). ^{1}H NMR (401 MHz, DMSO-d_6) δ 10.17 (t, *J* = 6.1 Hz, 1H), 7.66 – 7.58 (m, 4H), 7.58 – 7.48 (m, 6H), 7.47 – 7.39 (m, 4H), 4.94 (d, *J* = 6.0 Hz, 2H) ppm.

***tert*-butyl (4-((3-(phenyl(phenylimino)methyl)thioureido)methyl)phenyl)carbamate (55a)**.

N-phenylbenzimidoyl chloride **108b** (882 mg, 4.09 mmol), NaNCS (365 mg, 4.5 mmol) and *tert*-butyl (4-(aminomethyl) phenyl)carbamate **120** (1 g, 4.5 mmol) were reacted according to general procedure D_2. The crude product was purified by flash silica gel chromatography (DCM/MeOH in 98/2 ratio) to achieve the intermediate imidoylthiourea **55a** as a yellow solid (714 mg, yield 38%). ^{1}H NMR (401 MHz, Chloroform-*d*) δ 12.31 (bs, 1H), 8.05 (bs, 1H), 7.38 – 7.31 (m, 7H), 7.23 – 7.18 (m, 2H), 7.10 (t, *J* = 7.6 Hz, 2H), 6.95 (t, *J* = 8.0 Hz, 1H), 6.61 (d, *J* = 7.7 Hz, 2H), 6.48 (bs, 1H), 4.89 (dd, *J* = 10.7, 5.6 Hz, 2H), 1.51 (s, 9H) ppm.

4-(((2,3-diphenyl-1,2,4-thiadiazol-5(2*H*)-ylidene)amino)methyl)aniline dihydrobromide (55b).

Tert-butyl (4-((3-(phenyl(phenylimino)methyl)thioureido)methyl)phenyl)carbamate **55a** (282 mg, 0.61 mmol) and bromine (63 µL, 1.22 mmol) were reacted according to general procedure E. The precipitate was washed with a mixture of PE/EtOAc (2/1 v/v) to achieve the intermediate dihydro-bromide iminothiadiazole **55b** as a white solid (235 mg, yield 74%). ^{1}H NMR (401 MHz, DMSO-d_6) δ 10.39 (t, *J* = 6.1 Hz, 1H), 7.63 – 7.58 (m, 2H), 7.58 – 7.50 (m, 9H), 7.45 – 7.40 (m, 3H), 7.32 – 7.27 (m, 3H), 4.97 (d, *J* = 5.6 Hz, 2H) ppm.

***N*-((3-methylbenzyl)carbamothioyl)-*N'*-phenylbenzimidamide (56a)**.

N-phenylbenzimidoyl chloride **108b** (537 mg, 2.5 mmol), NaNCS (243 mg, 3 mmol) and *m*-tolylmethanamine **121** (347 µL, 2.75 mmol) were reacted according to general procedure D_2. The crude product was purified by flash silica gel chromatography (PE/EtOAc in 95/5 ratio) to achieve the intermediate imidoylthiourea **56a** as a yellow solid (359 mg, yield 40%). ^{1}H NMR (401 MHz, Chloroform-*d*) δ 12.40 (bs, 1H), 8.06 (bs, 1H), 7.40 – 7.33 (m, 1H), 7.33 – 7.27 (m, 2H), 7.27 – 7.17 (m, 5H), 7.15 – 7.06 (m, 3H), 6.95 (t, *J* = 7.4 Hz, 1H), 6.70 – 6.57 (m, 2H), 4.94 (d, *J* = 5.3 Hz, 2H), 2.34 (s, 3H) ppm.

***N*-(3-methylbenzyl)-2,3-diphenyl-1,2,4-thiadiazol-5(2*H*)-imine hydrobromide (56b).**

N-((3-methylbenzyl)carbamothioyl)-*N'*-phenylbenzimidamide **56a** (349 mg, 0.96 mmol) and bromine (75 µL, 1.46 mmol) were reacted according to general procedure E. The precipitate was washed with a mixture of PE/EtOAc (2/1 v/v) to achieve the intermediate hydrobromide iminothiadiazole **56b** as a white solid (339 mg, yield 80%). ^{1}H NMR (401 MHz, DMSO-d_6) δ 10.22 (t, *J* = 6.0 Hz, 1H), 7.61 (dt, *J* = 7.1, 1.6 Hz, 2H), 7.59 – 7.50 (m, 6H), 7.43 (dd, *J* = 8.4, 7.2 Hz, 2H), 7.35 – 7.24 (m, 3H), 7.16 (d, *J* = 7.3 Hz, 1H), 4.93 (d, *J* = 5.9 Hz, 2H), 2.33 (s, 3H) ppm.

***N'*-phenyl-*N*-((3-(trifluoromethyl)benzyl)carbamothioyl)benzimidamide (57a)**.

N-phenylbenzimidoyl chloride **108b** (700 mg, 3.25 mmol), NaNCS (290 mg, 3.6 mmol) and (3-(trifluoromethyl)phenyl)methanamine **122** (513 µL, 3.6 mmol) were reacted according to general procedure D_2. The crude product was purified by flash silica gel chromatography (PE/EtOAc in 98/2 ratio) to achieve the intermediate imidoylthiourea **57a** as a yellow solid (1.305 g, yield 97%). ^{1}H NMR (401 MHz, Chloroform-*d*) δ 12.51 (bs, 1H), 8.13 (bs, 1H), 7.68 (s, 1H), 7.62 (d, *J* = 7.9 Hz, 1H), 7.56 (d, *J* = 7.9 Hz, 1H), 7.48 (t, *J* = 7.9 Hz, 1H), 7.41 – 7.34 (m, 1H), 7.33 – 7.21 (m, 4H), 7.12 (t, *J* = 7.8 Hz, 2H), 6.96 (t, *J* = 7.5 Hz, 1H), 6.64 (d, *J* = 7.8 Hz, 2H), 5.04 (d, *J* = 5.5 Hz, 2H) ppm.

2,3-diphenyl-*N*-(3-(trifluoromethyl)benzyl)-1,2,4-thiadiazol-5(2*H*)-imine hydrobromide (57b).

N-((3-(trifluoromethyl)benzyl)carbamothioyl)-*N'*-phenyl benzimidamide **57a** (740 mg, 1.79 mmol) and bromine (138 µL, 2.68 mmol) were reacted according to general procedure E. The precipitate was washed with a mixture of PE/EtOAc (2/1 v/v) to achieve the intermediate hydrobromide imino-thiadiazole **57b** as a white solid (352 mg, yield 40%). ^{1}H NMR (401 MHz, DMSO-d_6) δ 10.35 (bs, 1H), 7.90 (s, 1H), 7.84 – 7.75 (m, 1H), 7.69 (dt, *J* = 15.2, 7.7 Hz, 2H), 7.65 – 7.59 (m, 2H), 7.58 – 7.49 (m, 6H), 7.45 – 7.38 (m, 2H), 5.07 (bs, 2H) ppm.

***N*-((3-methoxybenzyl)carbamothioyl)-*N'*-phenylbenzimidamide (58a)**.
1-(isothiocyanatomethyl)-3-methoxybenzene **93** (1.382 g, 7.7 mmol) and *N'*-phenylbenzimidamide **83** (1.513 g, 7.7 mmol) were reacted according to general procedure D_1. The crude product was purified by flash silica gel chromatography (PE/EtOAc in 96/4 ratio) to achieve the intermediate imidoylthiourea **58a** as a light-yellow solid (733 mg, yield 25%). ^{1}H NMR (401 MHz, Chloroform-*d*) δ 12.39 (bs, 1H), 8.08 (bs, 1H), 7.39 – 7.27 (m, 4H), 7.25 – 7.20 (m, 2H), 7.11 (t, *J* = 7.7 Hz, 2H), 7.02 – 6.91 (m, 3H), 6.83 (dd, *J* = 8.2, 2.6 Hz, 1H), 6.63 (d, *J* = 7.7 Hz, 2H), 4.95 (d, *J* = 5.4 Hz, 2H), 3.76 (s, 3H) ppm.

***N*-(3-methoxybenzyl)-2,3-diphenyl-1,2,4-thiadiazol-5(2*H*)-imine hydrobromide (58b).**
N-((3-methoxybenzyl)carbamothioyl)-*N'*-phenylbenzimidamide **58a** (724 mg, 1.93 mmol) and bromine (197 µL, 3.85 mmol) were reacted according to general procedure E. The precipitate was washed with a mixture of PE/EtOAc (2/1 v/v) to achieve the intermediate hydrobromide iminothiadiazole **58b** as a light yellow solid (465 mg, yield 53%). ^{1}H NMR (401 MHz, DMSO-d_6) δ 10.32 (t, *J* = 6.0 Hz, 1H), 7.65 – 7.59 (m, 2H), 7.58 – 7.50 (m, 6H), 7.42 (dd, *J* = 8.4, 7.1 Hz, 2H), 7.34 (t, *J* = 7.8 Hz, 1H), 7.09 – 7.02 (m, 2H), 6.91 (dd, *J* = 8.3, 2.6 Hz, 1H), 4.95 (d, *J* = 6.0 Hz, 2H), 3.77 (s, 3H) ppm.

***N*-((3-ethoxybenzyl)carbamothioyl)-*N'*-phenylbenzimidamide (59a).**

N-phenylbenzimidoyl chloride **108b** (900 mg, 4.1 mmol), NaNCS (365 mg, 4.51 mmol) and (3-ethoxyphenyl)methanamine **124** (670 µL, 4.51 mmol) were reacted according to general procedure D_2. The crude product was purified by flash silica gel chromatography (PE/EtOAc in 95/5 ratio) to achieve the intermediate imidoylthiourea **59a** as a white crystalline solid (850 mg, yield 53%). ^{1}H NMR (401 MHz, Chloroform-*d*) δ 12.41 (bs, 1H), 8.09 (bs, 1H), 7.36 (d, *J* = 7.2 Hz, 1H), 7.34 – 7.21 (m, 5H), 7.12 (t, *J* = 7.8 Hz, 2H), 7.01 – 6.93 (m, 3H), 6.83 (d, *J* = 8.4 Hz, 1H), 6.65 (d, *J* = 7.7 Hz, 2H), 4.96 (d, *J* = 6.8 Hz, 2H), 3.98 (q, *J* = 7.2 Hz, 2H), 1.38 (t, *J* = 6.9 Hz, 3H) ppm.

***N*-(3-ethoxybenzyl)-2,3-diphenyl-1,2,4-thiadiazol-5(2*H*)-imine hydrobromide (59b).**
N-((3-ethoxybenzyl)carbamothioyl)-*N'*-phenylbenzimidamide **59a** (850 mg, 2.2 mmol) and bromine (170 µL, 3.3 mmol) were reacted according to general procedure E. The precipitate was washed with a mixture of PE/EtOAc (2/1 v/v) to achieve the intermediate hydrobromide iminothiadiazole **59b** as a white solid (640 mg, yield 62%). ^{1}H NMR (401 MHz, DMSO-d_6) δ 10.24 (t, *J* = 5.9 Hz, 1H), 7.64 – 7.60 (m, 2H), 7.59 – 7.50 (m, 6H), 7.42 (dd, *J* = 8.4, 7.1 Hz, 2H), 7.32 (t, *J* = 7.8 Hz, 1H), 7.06 – 6.99 (m, 2H), 6.90 (dd, *J* = 7.9, 2.3 Hz, 1H), 4.93 (d, *J* = 5.5 Hz, 2H), 4.03 (q, *J* = 6.9 Hz, 2H), 1.32 (t, *J* = 7.0 Hz, 3H) ppm.

***tert*-butyl (3-((3-(phenyl(phenylimino)methyl)thioureido)methyl)phenyl)carbamate (60a)**.
N-phenylbenzimidoyl chloride **108b** (882 mg, 4.09 mmol), NaNCS (365 mg, 4.5 mmol) and *tert*-butyl (3-(aminomethyl) phenyl)carbamate **125** (1 g, 4.5 mmol) were reacted according to general procedure D_2. The crude product was purified by flash silica gel chromatography (DCM/MeOH in 98/2 ratio) to achieve the intermediate imidoylthiourea **60a** as a yellow solid (917 mg, yield 49%). ^{1}H NMR (401 MHz, Chloroform-*d*) δ 12.40 (bs, 1H), 8.05 (bs, 1H), 7.55 – 7.41 (m, 3H), 7.37 – 7.27 (m, 6H), 7.15 – 7.03 (m, 3H), 7.03 – 6.85 (m, 2H), 6.66 (bs, 1H), 4.93 (d, *J* = 5.8 Hz, 2H), 1.51 (s, 9H) ppm.

3-(((2,3-diphenyl-1,2,4-thiadiazol-5(2*H*)-ylidene)amino)methyl)aniline dihydrobromide (60b).

Tert-butyl (3-((3-(phenyl(phenylimino)methyl)thioureido)methyl)phenyl)carbamate **60a** (463 mg, 1.01 mmol) and bromine (104 µL, 2.02 mmol) were reacted according to general procedure E. The precipitate was washed with a mixture of PE/EtOAc (2/1 v/v) to achieve the intermediate dihydrobromide iminothiadiazole **60b** as a yellow solid (388 mg, yield 74%). ^{1}H NMR (401 MHz, DMSO-d_6) δ 10.47 (t, *J* = 6.2 Hz, 1H), 7.62 – 7.59 (m, 2H), 7.58 – 7.47 (m, 9H), 7.45 – 7.41 (m, 3H), 7.36 (bs, 1H), 7.24 (d, *J* = 7.5 Hz, 2H), 5.01 (d, *J* = 5.8 Hz, 2H) ppm.

***N*-((3-fluorobenzyl)carbamothioyl)-*N'*-phenylbenzimidamide (61a).**

N-phenylbenzimidoyl chloride **108b** (700 mg, 3.24 mmol), NaNCS (316 mg, 3.89 mmol) and (3-fluorophenyl)methanamine **126** (406 µL, 3.56 mmol) were reacted according to general procedure D_2. The crude product was purified by flash silica gel chromatography (PE/EtOAc in 96/4 ratio) to achieve the intermediate imidoylthiourea **61a** as a yellow solid (458 mg, yield 39%).^{1}H NMR (401 MHz, Chloroform-*d*) δ 12.43 (bs, 1H), 8.09 (bs, 1H), 7.39 – 7.27 (m, 4H), 7.25 – 7.21 (m, 2H), 7.19 (d, *J* = 7.7 Hz, 1H), 7.17 – 7.06 (m, 3H), 7.03 – 6.92 (m, 2H), 6.64 (d, *J* = 7.7 Hz, 2H), 4.98 (d, *J* = 5.4 Hz, 2H) ppm.

(*Z*)-*N*-(3-fluorobenzyl)-2,3-diphenyl-1,2,4-thiadiazol-5(2*H*)-imine hydrobromide (61b).

N-((3-fluorobenzyl)carbamothioyl)-*N'*-phenylbenzimidamide **61a** (450 mg, 1.24 mmol) and bromine (95 µL, 1.86 mmol) were reacted according to general procedure E. The precipitate was washed with a mixture of PE/EtOAc (2/1 v/v) to achieve the intermediate hydrobromide iminothiadiazole **61b** as a white solid (426 mg, yield 78%). ^{1}H NMR (401 MHz, DMSO-d_6) δ 10.30 (t, *J* = 6.0 Hz, 1H), 7.65 – 7.58 (m, 2H), 7.58 – 7.50 (m, 6H), 7.50 – 7.39 (m, 3H), 7.36 – 7.29 (m, 2H), 7.23 – 7.15 (m, 1H), 5.00 (d, *J* = 5.7 Hz, 2H) ppm.

***N*-((3-chlorobenzyl)carbamothioyl)-*N'*-phenylbenzimidamide (62a)**.

1-chloro-3-(isothiocyanatomethyl)benzene **94** (646 mg, 3.5 mmol) and *N′*-phenylbenzimidamide **83** (690 mg, 3.5 mmol) were reacted according to general procedure D_1. The crude product was purified by flash silica gel chromatography (PE/EtOAc in 97/3 ratio) to achieve the intermediate imidoylthiourea **62a** as a light-yellow solid (181 mg, yield 14%). ^{1}H NMR (401 MHz, Chloroform-*d*) δ 12.44 (bs, 1H), 8.11 (bs, 1H), 7.44 – 7.34 (m, 3H), 7.34 – 7.22 (m, 7H), 7.15 (dt, *J* = 16.2, 7.5 Hz, 2H), 6.97 (q, *J* = 7.6 Hz, 1H), 6.65 (d, *J* = 7.8 Hz, 1H), 4.96 (d, *J* = 5.5 Hz, 2H) ppm.

***N*-(3-chlorobenzyl)-2,3-diphenyl-1,2,4-thiadiazol-5(2*H*)-imine hydrobromide (62b).**

N-((3-chlorobenzyl)carbamothioyl)-*N′*-phenylbenzimidamide **62a** (166 mg, 0.44 mmol) and bromine (45 µL, 0.87 mmol) were reacted according to general procedure E. The precipitate was washed with a mixture of PE/EtOAc (2/1 v/v) to achieve the intermediate hydrobromide iminothiadiazole **62b** as a light yellow solid (156 mg, yield 77%). ^{1}H NMR (401 MHz, DMSO-d_6) δ 10.28 (t, *J* = 6.0 Hz, 1H), 7.62 (dd, *J* = 7.7, 2.0 Hz, 2H), 7.58 – 7.49 (m, 7H), 7.48 – 7.39 (m, 5H), 4.99 (d, *J* = 5.8 Hz, 2H) ppm.

***N*-((2-methylbenzyl)carbamothioyl)-*N′*-phenylbenzimidamide (63a)**.

N-phenylbenzimidoyl chloride **108b** (515 mg, 2.4 mmol), NaNCS (235 mg, 2.9 mmol) and *o*-tolylmethanamine **127** (327 µL, 2.64 mmol) were reacted according to general procedure D_2. The crude product was purified by flash silica gel chromatography (PE/EtOAc in 95/5 ratio) to achieve the intermediate imidoylthiourea **63a** as a yellow solid (302 mg, yield 35%). ^{1}H NMR (401 MHz, Chloroform-*d*) δ 12.32 (bs, 1H), 8.05 (bs, 1H), 7.43 – 7.32 (m, 2H), 7.29 (t, *J* = 7.5 Hz, 2H), 7.25 – 7.15 (m, 5H), 7.10 (t, *J* = 7.7 Hz, 2H), 6.94 (t, *J* = 7.4 Hz, 1H), 6.60 (d, *J* = 7.8 Hz, 2H), 4.94 (d, *J* = 5.2 Hz, 2H), 2.40 (s, 3H) ppm.

***N*-(2-methylbenzyl)-2,3-diphenyl-1,2,4-thiadiazol-5(2*H*)-imine hydrobromide (63b).**

N-((2-methylbenzyl)carbamothioyl)-*N'*-phenylbenzimidamide **63a** (255 mg, 0.71 mmol) and bromine (73 µL, 1.42 mmol) were reacted according to general procedure E. The precipitate was washed with a mixture of PE/EtOAc (2/1 v/v) to achieve the intermediate hydrobromide iminothiadiazole **63b** as a white solid (244 mg, yield 78%). ^{1}H NMR (401 MHz, DMSO-*d₆*) δ 10.22 (t, *J* = 5.9 Hz, 1H), 7.61 (dt, *J* = 7.5, 1.5 Hz, 2H), 7.59 – 7.50 (m, 6H), 7.46 – 7.39 (m, 3H), 7.28 – 7.21 (m, 3H), 4.94 (d, *J* = 5.9 Hz, 2H), 2.42 (s, 3H) ppm.

***N'*-phenyl-*N*-((2-(trifluoromethyl)benzyl)carbamothioyl)benzimidamide (64a)**.

N-phenylbenzimidoyl chloride **108b** (700 mg, 3.25 mmol), NaNCS (290 mg, 3.6 mmol) and (2-(trifluoromethyl)phenyl)methanamine **128** (627 µL, 3.6 mmol) were reacted according to general procedure D_2. The crude product was purified by flash silica gel chromatography (PE/EtOAc in 98/2 ratio) to achieve the intermediate imidoylthiourea **64a** as a yellow solid (1.337 g, yield 99%). ^{1}H NMR (401 MHz, Chloroform-*d*) δ 12.48 (bs, 1H), 8.08 (bs, 1H), 7.82 (d, *J* = 7.8 Hz, 1H), 7.68 (d, *J* = 7.8 Hz, 1H), 7.57 (t, *J* = 7.5 Hz, 1H), 7.41 (t, *J* = 7.7 Hz, 1H), 7.38 – 7.33 (m, 1H), 7.32 – 7.26 (m, 2H), 7.25 – 7.19 (m, 2H), 7.11 (t, *J* = 7.8 Hz, 2H), 6.95 (t, *J* = 7.4 Hz, 1H), 6.67 – 6.60 (m, 2H), 5.19 (d, *J* = 5.8 Hz, 2H) ppm.

2,3-diphenyl-*N*-(2-(trifluoromethyl)benzyl)-1,2,4-thiadiazol-5(2*H*)-imine hydrobromide (64b).

N-((2-(trifluoromethyl)benzyl)carbamothioyl)-*N'*-phenylbenzimidamide **64a** (740 mg, 1.79 mmol) and bromine (138 µL, 2.68 mmol) were reacted according to general procedure E. The precipitate was washed with a mixture of PE/EtOAc (2/1 v/v) to achieve the intermediate hydrobromide iminothiadiazole **64b** as a white solid (411 mg, yield 47%). ^{1}H NMR (401 MHz, DMSO-*d₆*) δ 10.34 (bs, 1H), 7.82 (d, *J* = 8.0 Hz, 1H), 7.80 – 7.70 (m, 2H), 7.65 – 7.58 (m, 3H), 7.58 – 7.52 (m, 4H), 7.52 – 7.48 (m, 2H), 7.45 – 7.38 (m, 2H), 5.14 (bs, 2H) ppm.

***N*-((2-methoxybenzyl)carbamothioyl)-*N'*-phenylbenzimidamide (65a)**.

1-(isothiocyanatomethyl)-2-methoxybenzene **95** (393 mg, 2.2 mmol) and *N'*-phenylbenzimidamide **83** (430 mg, 2.2 mmol) were reacted according to general procedure D_1. The crude product was purified by flash silica gel chromatography (PE/EtOAc in 96/4 ratio) to achieve the intermediate imidoylthiourea **65a** as a light-yellow solid (243 mg, yield 30%). ^{1}H NMR (401 MHz, Chloroform-*d*) δ 12.37 (bs, 1H), 7.97 (bs, 1H), 7.44 (dd, *J* = 7.4, 1.7 Hz, 1H), 7.36 – 7.29 (m, 1H), 7.26 (ddt, *J* = 7.6, 5.9, 1.4 Hz, 3H), 7.23 – 7.18 (m, 2H), 7.11 (ddd, *J* = 8.3, 5.6, 1.7 Hz, 2H), 6.94 (tt, *J* = 7.2, 1.2 Hz, 2H), 6.84 (dd, *J* = 8.3, 1.1 Hz, 1H), 6.66 – 6.58 (m, 2H), 4.96 (d, *J* = 5.7 Hz, 2H), 3.66 (s, 3H) ppm.

***N*-(2-methoxybenzyl)-2,3-diphenyl-1,2,4-thiadiazol-5(2*H*)-imine hydrobromide (65b).**

N-((2-methoxybenzyl)carbamothioyl)-*N'*-phenylbenzimidamide **65a** (243 mg, 0.65 mmol) and bromine (66 µL, 1.29 mmol) were reacted according to general procedure E. The precipitate was washed with a mixture of PE/EtOAc (2/1 v/v) to achieve the intermediate hydrobromide iminothiadiazole **65b** as a white solid (241 mg, yield 82%). ^{1}H NMR (401 MHz, DMSO-d_6) δ 10.21 (t, *J* = 5.8 Hz, 1H), 7.64 – 7.58 (m, 2H), 7.57 – 7.50 (m, 6H), 7.47 – 7.39 (m, 3H), 7.36 (td, *J* = 7.8, 1.8 Hz, 1H), 7.12 – 7.05 (m, 1H), 6.98 (td, *J* = 7.5, 1.2 Hz, 1H), 4.88 (d, *J* = 6.0 Hz, 2H), 3.88 (s, 3H) ppm.

***N*-((2-chlorobenzyl)carbamothioyl)-*N'*-phenylbenzimidamide (66a)**.

1-chloro-2-(isothiocyanatomethyl)benzene **96** (314 mg, 1.7 mmol) and *N'*-phenylbenzimidamide **83** (336 mg, 1.7 mmol) were reacted according to general procedure D_1. The crude product was purified by flash silica gel chromatography (PE/EtOAc in 97/3 ratio) to achieve the intermediate imidoylthiourea **66a** as a light-yellow solid (330 mg, yield 51%). ^{1}H NMR (401 MHz, Chloroform-*d*) δ 12.57 (bs, 1H), 8.03 (bs, 1H), 7.63 – 7.57 (m, 1H), 7.41 – 7.27 (m, 6H), 7.24 – 7.19 (m, 2H), 7.15 – 7.08 (m, 2H), 6.99 – 6.92 (m, 1H), 6.66 (d, *J* = 7.7 Hz, 2H), 5.05 (d, *J* = 5.7 Hz, 2H) ppm.

***N*-(2-chlorobenzyl)-2,3-diphenyl-1,2,4-thiadiazol-5(2*H*)-imine hydrobromide (66b).**

N-((2-chlorobenzyl)carbamothioyl)-*N'*-phenylbenzimidamide **66a** (322 mg, 0.85 mmol) and bromine (87 µL, 1.7 mmol) were reacted according to general procedure E. The precipitate was washed with

a mixture of PE/EtOAc (2/1 v/v) to achieve the intermediate hydrobromide iminothiadiazole **66b** as a light yellow solid (340 mg, yield 87%). ^{1}H NMR (401 MHz, DMSO-d_6) δ 10.26 (t, *J* = 6.0 Hz, 1H), 7.64 – 7.59 (m, 3H), 7.58 – 7.50 (m, 7H), 7.43 (ddd, *J* = 9.7, 5.7, 2.3 Hz, 4H), 5.03 (d, *J* = 5.9 Hz, 2H) ppm.

***tert*-butyl (2-((3-(phenyl(phenylimino)methyl)thioureido)methyl)phenyl)carbamate (67a)**.
N-phenylbenzimidoyl chloride **108b** (882 mg, 4.09 mmol), NaNCS (365 mg, 4.5 mmol) and *tert*-butyl (2-(aminomethyl) phenyl)carbamate **129** (1 g, 4.5 mmol) were reacted according to general procedure D_2. The crude product was purified by flash silica gel chromatography (DCM/MeOH in 98/2 ratio) to achieve the intermediate imidoylthiourea **67a** as a yellow solid (1.377 g, yield 73%). *E/Z* ^{1}H NMR (401 MHz, Chloroform-*d*) δ 12.02 (bs, 1H), 11.20 (bs, 1H), 7.83 (d, *J* = 7.5 Hz, 1H), 7.63 (d, *J* = 8.0 Hz, 1H), 7.58 (d, *J* = 7.6 Hz, 4H), 7.51 (d, *J* = 7.7 Hz, 2H), 7.44 (dt, *J* = 15.8, 7.5 Hz, 8H), 7.38 (d, *J* = 7.4 Hz, 1H), 7.35 – 7.28 (m, 7H), 7.22 (d, *J* = 7.6 Hz, 2H), 7.19 – 7.14 (m, 3H), 7.02 – 6.95 (m, 3H), 4.88 (d, *J* = 6.1 Hz, 2H), 4.81 (d, *J* = 5.6 Hz, 2H), 1.52 (s, 9H), 1.43 (s, 9H) ppm.

2-(((2,3-diphenyl-1,2,4-thiadiazol-5(2*H*)-ylidene)amino)methyl)aniline dihydrobromide (67b).
Tert-butyl (2-((3-(phenyl(phenylimino)methyl)thioureido)methyl) phenyl)carbamate **67a** (1.377 g, 2.99 mmol) and bromine (306 µL, 5.98 mmol) were reacted according to general procedure E. The precipitate was washed with a mixture of PE/EtOAc (2/1 v/v) to achieve the intermediate dihydro-bromide Iminothiadiazole **67b** as a light yellow solid (1.222 g, yield 79%). ^{1}H NMR (401 MHz, DMSO-d_6) δ 7.63 – 7.59 (m, 2H), 7.57 – 7.50 (m, 7H), 7.46 – 7.39 (m, 4H), 7.32 – 7.19 (m, 2H), 7.15 – 7.03 (m, 2H), 4.98 (s, 2H), 4.83 (bs, 1H) ppm.

***N*-(benzylcarbamothioyl)-4-methoxy-*N'*-phenylbenzimidamide (68a).**
4-methoxy-*N*-phenylbenzimidoyl chloride **109b** (1.049 g, 4.26 mmol), NaNCS (414 mg, 5.11 mmol) and benzylamine **118** (513 µL, 4.7 mmol) were reacted according to general procedure D_2. The crude

product was purified by flash silica gel chromatography (PE/EtOAc in 9/1 ratio) to achieve the intermediate imidoylthiourea **68a** as a yellow solid (350 mg, yield 22%). ^{1}H NMR (401 MHz, Chloroform-*d*) δ 12.45 (bs, 1H), 8.05 (bs, 1H), 7.41 – 7.28 (m, 6H), 7.19 – 7.10 (m, 3H), 7.04 – 6.91 (m, 2H), 6.83 – 6.72 (m, 2H), 6.67 – 6.60 (m, 1H), 4.94 (d, *J* = 5.1 Hz, 2H), 3.78 (s, 3H) ppm.

***N*-benzyl-3-(4-methoxyphenyl)-2-phenyl-1,2,4-thiadiazol-5(2*H*)-imine hydrobromide (68b).**
N-(benzylcarbamothioyl)-4-methoxy-*N'*-phenylbenzimidamide **68a** (350 mg, 0.93 mmol) and bromine (57 µL, 1.1 mmol) were reacted according to general procedure E. The precipitate was washed with a mixture of PE/EtOAc (2/1 v/v) to achieve the intermediate hydrobromide iminothiadiazole **68b** as a yellow solid (303 mg, yield 72%). ^{1}H NMR (401 MHz, DMSO-d_6) δ 10.23 (t, *J* = 6.0 Hz, 1H), 7.64 (dd, *J* = 7.5, 2.2 Hz, 2H), 7.61 – 7.54 (m, 3H), 7.52 – 7.46 (m, 4H), 7.42 (dd, *J* = 8.5, 6.5 Hz, 2H), 7.35 (t, *J* = 7.2 Hz, 1H), 7.00 – 6.95 (m, 2H), 4.97 (d, *J* = 5.7 Hz, 2H), 3.78 (s, 3H) ppm.

***N*-(benzylcarbamothioyl)-3-methoxy-*N'*-phenylbenzimidamide (69a).**
3-methoxy-*N*-phenylbenzimidoyl chloride **110b** (846 mg, 3.4 mmol), NaNCS (307 mg, 3.8 mmol) and benzylamine **118** (414 µL, 3.8 mmol) were reacted according to general procedure D_2. The crude product was purified by recrystallization from a mixture of EtOAc/MeOH (95/5, v/v) to achieve the intermediate imidoylthiourea **69a** as a white crystalline solid (458 mg, yield 36%). ^{1}H NMR (401 MHz, Chloroform-*d*) δ 12.38 (bs, 1H), 8.09 (bs, 1H), 7.45 – 7.25 (m, 5H), 7.21 (t, *J* = 8.2 Hz, 1H), 7.12 (t, *J* = 7.7 Hz, 2H), 6.95 (t, *J* = 7.5 Hz, 1H), 6.85 (dd, *J* = 22.3, 7.8 Hz, 2H), 6.70 (s, 1H), 6.64 (d, *J* = 7.7 Hz, 2H), 4.97 (d, *J* = 5.0 Hz, 2H), 3.65 (s, 3H) ppm.

***N*-benzyl-3-(3-methoxyphenyl)-2-phenyl-1,2,4-thiadiazol-5(2*H*)-imine hydrobromide (69b).**
N-(benzylcarbamothioyl)-3-methoxy-*N'*-phenylbenzimidamide **69a** (423 mg, 1.1 mmol) and bromine (87 µL, 1.7 mmol) were reacted according to general procedure E. The precipitate was washed with a mixture of PE/EtOAc (2/1 v/v) to achieve the intermediate hydrobromide iminothiadiazole **69b** as

a light yellow solid (440 mg, yield 81%). ^{1}H NMR (401 MHz, DMSO-d_6) δ 10.34 (s, 1H), 7.62 (dd, *J* = 7.6, 2.1 Hz, 2H), 7.58 – 7.53 (m, 3H), 7.50 – 7.46 (m, 2H), 7.45 – 7.39 (m, 2H), 7.38 – 7.32 (m, 2H), 7.12 (dd, *J* = 8.0, 2.1 Hz, 2H), 7.01 (t, *J* = 2.0 Hz, 1H), 4.97 (s, 2H), 3.62 (s, 3H) ppm.

***N*-(benzylcarbamothioyl)-2-methoxy-*N'*-phenylbenzimidamide (70a)**.

2-methoxy-*N*-phenylbenzimidoyl chloride **111b** (1.1 g, 4.8 mmol), NaNCS (462 mg, 5.7 mmol) and bezylamine **118** (580 µL, 5.3 mmol) were reacted according to general procedure D_2. The crude product was purified by flash silica gel chromatography (PE/EtOAc in 91/9 ratio) to achieve the intermediate imidoylthiourea **70a** as a yellow solid (686 mg, yield 41%). ^{1}H NMR (401 MHz, Chloroform-*d*) δ 12.41 (bs, 1H), 8.03 (bs, 1H), 7.45 – 7.40 (m, 2H), 7.40 – 7.33 (m, 2H), 7.33 – 7.25 (m, 2H), 7.08 (td, *J* = 8.0, 3.4 Hz, 3H), 6.92 (t, *J* = 7.4 Hz, 1H), 6.85 (t, *J* = 7.5 Hz, 1H), 6.80 (dd, *J* = 8.4, 0.9 Hz, 1H), 6.64 (d, *J* = 7.7 Hz, 2H), 4.97 (d, *J* = 5.3 Hz, 2H), 3.68 (s, 3H) ppm.

***N*-benzyl-3-(2-methoxyphenyl)-2-phenyl-1,2,4-thiadiazol-5(2*H*)-imine hydrobromide (70b).**

N-(benzylcarbamothioyl)-2-methoxy-*N'*-phenylbenzimidamide **70a** (336 mg, 0.9 mmol) and bromine (92 µL, 1.8 mmol) were reacted according to general procedure E. The precipitate was washed with a mixture of PE/EtOAc (2/1 v/v) to achieve the intermediate hydrobromide iminothiadiazole **70b** as a white solid (324 mg, yield 73%). ^{1}H NMR (401 MHz, DMSO-d_6) δ 10.27 (t, *J* = 6.0 Hz, 1H), 7.65 (dd, *J* = 7.6, 1.7 Hz, 1H), 7.57 – 7.48 (m, 1H), 7.48 – 7.40 (m, 8H), 7.40 – 7.32 (m, 2H), 7.09 (td, *J* = 7.5, 0.9 Hz, 1H), 6.97 (d, *J* = 8.5 Hz, 1H), 4.92 (d, *J* = 5.9 Hz, 2H), 3.41 (s, 3H) ppm.

***N*-(benzylcarbamothioyl)-*N'*-phenylisonicotinimidamide (71a).**

N-phenylisonicotinimidoyl chloride hydrochloride **112b** (460 mg, 1.82 mmol), TEA (254 µL, 1.82 mmol), NaNCS (162 mg, 2 mmol) and benzylamine **118** (218 µL, 2 mmol) were reacted according to general procedure D_2. The crude product was purified by flash silica gel chromatography (DCM/MeOH in 99.5/0.5 ratio) to achieve the intermediate imidoylthiourea **71a** as a white crystalline

solid (354 mg, yield 56%). ^{1}H NMR (401 MHz, Chloroform-*d*) δ 12.11 (bs, 1H), 8.62 – 8.57 (m, 2H), 8.04 (s, 1H), 7.44 – 7.32 (m, 4H), 7.32 – 7.27 (m, 1H), 7.16 – 7.07 (m, 4H), 6.98 (t, *J* = 7.5 Hz, 1H), 6.60 (dd, *J* = 7.6, 1.2 Hz, 2H), 4.96 (d, *J* = 5.4 Hz, 2H).

***N*-benzyl-2-phenyl-3-(pyridin-4-yl)-1,2,4-thiadiazol-5(2*H*)-imine hydrobromide (71b).**

N-(benzylcarbamothioyl)-*N'*-phenylisonicotinimidamide **71a** (445 mg, 1.28 mmol) and bromine (130 µL, 2.57 mmol) were reacted according to general procedure E. The precipitate was washed with a mixture of PE/EtOAc (2/1 v/v) to achieve the intermediate hydrobromide iminothiadiazole **71b** as a yellow solid (431 mg, yield 79%). ^{1}H NMR (401 MHz, DMSO-d_6) δ 10.60 (t, *J* = 6.1 Hz, 1H), 8.74 (d, *J* = 5.2 Hz, 2H), 7.66 – 7.60 (m, 2H), 7.59 – 7.51 (m, 5H), 7.50 – 7.45 (m, 2H), 7.43 (t, *J* = 7.3 Hz, 2H), 7.38 – 7.31 (m, 1H), 4.99 (d, *J* = 5.8 Hz, 2H) ppm.

***N*-(benzylcarbamothioyl)-*N'*-phenylnicotinimidamide (72a)**.

N-phenylnicotinimidoyl chloride hydrochloride **113b** (460 mg, 1.82 mmol), TEA (254 µL, 1.82 mmol), NaNCS (162 mg, 2 mmol) and benzylamine **118** (218 µL, 2 mmol) were reacted according to general procedure D_2. The crude product was purified by flash silica gel chromatography (DCM/MeOH in 99.5/0.5 ratio) to achieve the intermediate imidoylthiourea **72a** as a white crystalline solid (360 mg, yield 57%). ^{1}H NMR (401 MHz, Chloroform-*d*) δ 12.32 (bs, 1H), 8.61 – 8.56 (m, 2H), 8.03 (s, 1H), 7.44 – 7.32 (m, 4H), 7.32 – 7.27 (m, 1H), 7.16 – 7.07 (m, 4H), 6.98 (t, *J* = 7.5 Hz, 1H), 6.63 (dd, *J* = 7.6, 1.2 Hz, 2H), 4.98 (d, *J* = 5.4 Hz, 2H).

***N*-benzyl-2-phenyl-3-(pyridin-3-yl)-1,2,4-thiadiazol-5(2*H*)-imine hydrobromide 72b.**

N-(benzylcarbamothioyl)-*N'*-phenylnicotinimidamide **72a** (282 mg, 0.81 mmol) and bromine (62 µL, 1.21 mmol) were reacted according to general procedure E. The precipitate was washed with a mixture of PE/EtOAc (2/1 v/v) to achieve the intermediate hydrobromide iminothiadiazole **72b** as a yellow solid (285 mg, yield 83%). ^{1}H NMR (401 MHz, DMSO-d_6) δ 10.37 (t, *J* = 6.0 Hz, 1H), 8.70 (d,

J = 2.7 Hz, 2H), 7.87 (dt, *J* = 8.1, 2.0 Hz, 1H), 7.68 – 7.63 (m, 2H), 7.60 – 7.54 (m, 3H), 7.51 – 7.46 (m, 3H), 7.43 (t, *J* = 7.5 Hz, 2H), 7.38 – 7.32 (m, 1H), 4.99 (d, *J* = 5.9 Hz, 2H) ppm.

***N*-(benzylcarbamothioyl)-*N′*-phenylfuran-2-carboximidamide (73a).**

N-phenylfuran-2-carbimidoyl chloride **114b** (720 mg, 3.5 mmol), NaNCS (312 mg, 3.85 mmol) and benzylamine **118** (421 µL, 3.8 mmol) were reacted according to general procedure D_2. The crude product was purified by flash silica gel chromatography (PE/EtOAc in 95/5 ratio) to achieve the intermediate imidoylthiourea **73a** as a brown solid (689 mg, yield 59%). ^{1}H NMR (401 MHz, Chloroform-*d*) δ 12.05 (bs, 1H), 8.85 (bs, 1H), 7.42 (d, *J* = 1.7 Hz, 1H), 7.39 – 7.27 (m, 7H), 7.15 (td, *J* = 7.4, 1.3 Hz, 1H), 6.83 (d, *J* = 7.8 Hz, 2H), 6.28 (dd, *J* = 3.7, 1.8 Hz, 1H), 5.68 (d, *J* = 3.7 Hz, 1H), 4.98 (d, *J* = 5.5 Hz, 2H) ppm.

***N*-benzyl-3-(furan-2-yl)-2-phenyl-1,2,4-thiadiazol-5(2*H*)-imine hydrobromide (73b).**

N-(benzylcarbamothioyl)-*N′*-phenylfuran-2-carboximidamide **73a** (660 mg, 1.97 mmol) and bromine (151 µL, 2.95 mmol) were reacted according to general procedure E. The precipitate was washed with a mixture of PE/EtOAc (2/1 v/v) to achieve the intermediate hydrobromide iminothiadiazole **73b** as a pink solid (270 mg, yield 33%). ^{1}H NMR (401 MHz, DMSO-d_6) δ 10.16 (t, *J* = 6.0 Hz, 1H), 8.00 (d, *J* = 1.8 Hz, 1H), 7.81 – 7.74 (m, 2H), 7.72 – 7.61 (m, 3H), 7.48 (d, *J* = 6.9 Hz, 2H), 7.42 (t, *J* = 7.6 Hz, 2H), 7.39 – 7.31 (m, 1H), 6.82 (d, *J* = 3.6 Hz, 1H), 6.72 (dd, *J* = 3.7, 1.7 Hz, 1H), 4.96 (d, *J* = 5.9 Hz, 2H) ppm.

***N*-(benzylcarbamothioyl)-*N′*-(*m*-tolyl)benzimidamide (74a).**

N-(*m*-tolyl)benzimidoyl chloride **115b** (950 mg, 4.1 mmol), NaNCS (369 mg, 4.5 mmol) and benzylamine **118** (497 µL, 4.5 mmol) were reacted according to general procedure D_2. The crude product was purified by flash silica gel chromatography (PE/EtOAc in 96/4 ratio) to achieve the intermediate imidoylthiourea **74a** as a brown solid (340 mg, yield 24%). ^{1}H NMR (401 MHz, Chloroform-*d*) δ

12.42 (bs, 1H), 8.05 (bs, 1H), 7.42 (d, *J* = 7.5 Hz, 2H), 7.36 (dd, *J* = 8.4, 6.3 Hz, 3H), 7.32 – 7.26 (m, 3H), 7.26 – 7.19 (m, 2H), 6.97 (t, *J* = 7.7 Hz, 1H), 6.76 (d, *J* = 7.6 Hz, 1H), 6.48 (s, 1H), 6.38 (d, *J* = 7.8 Hz, 1H), 4.98 (d, *J* = 5.4 Hz, 2H), 2.18 (s, 3H) ppm.

***N*-benzyl-3-phenyl-2-(*m*-tolyl)-1,2,4-thiadiazol-5(2*H*)-imine hydrobromide (74b).**

N-(benzylcarbamothioyl)-*N'*-(*m*-tolyl)benzimidamide **74a** (163 mg, 0.45 mmol) and bromine (46 µL, 0.9 mmol) were reacted according to general procedure E. The precipitate was washed with a mixture of PE/EtOAc (2/1 v/v) to achieve the intermediate hydrobromide iminothiadiazole **74b** as a white solid (130 mg, yield 66%). ^{1}H NMR (401 MHz, DMSO-d_6) δ 10.26 (t, *J* = 5.6, 5.1 Hz, 1H), 7.60 – 7.52 (m, 3H), 7.51 – 7.31 (m, 11H), 4.97 (d, *J* = 4.4 Hz, 2H), 2.32 (s, 3H) ppm.

***N'*-benzyl-*N*-(benzylcarbamothioyl)benzimidamide (75a)**.

N-benzylbenzimidoyl chloride **116b** (1.105 g, 4.8 mmol), NaNCS (462 mg, 5.7 mmol) and bezylamine **118** (580 µL, 5.3 mmol) were reacted according to general procedure D_2. The crude product was purified by flash silica gel chromatography (PE/EtOAc in 91/9 ratio) to achieve the intermediate imidoylthiourea **75a** as a white solid (1.154 g, yield 67%). *E/Z* ^{1}H NMR (401 MHz, Chloroform-*d*) δ 12.79 (s, 2H), 7.85 – 7.76 (m, 2H), 7.55 – 7.41 (m, 8H), 7.41 – 7.27 (m, 16H), 7.20 (q, *J* = 4.5, 3.5 Hz, 4H), 6.98 (dd, *J* = 6.6, 2.9 Hz, 2H), 4.88 (d, *J* = 4.9 Hz, 2H), 4.66 (d, *J* = 5.7 Hz, 2H), 4.55 (s, 2H), 4.36 (s, 2H) ppm.

***N*,2-dibenzyl-3-phenyl-1,2,4-thiadiazol-5(2*H*)-imine hydrobromide (75b).**

N'-benzyl-*N*-(benzylcarbamothioyl)benzimidamide **75a** (600 mg, 1.67 mmol) and bromine (171 µL, 3.34 mmol) were reacted according to general procedure E. The precipitate was washed with a mixture of PE/EtOAc (2/1 v/v) to achieve the intermediate hydrobromide iminothiadiazole **75b** as a yellow-orange solid (588 mg, yield 80%). ^{1}H NMR (401 MHz, DMSO-d_6) δ 10.10 (t, *J* = 6.0 Hz, 1H), 7.87 – 7.81 (m, 2H), 7.75 – 7.69 (m, 1H), 7.64 (dd, *J* = 8.3, 6.8 Hz, 2H), 7.41 (td, *J* = 6.4, 5.7, 3.9 Hz, 7H), 7.37 – 7.29 (m, 3H), 5.40 (s, 2H), 4.85 (d, *J* = 5.9 Hz, 2H) ppm.

***N*-(benzylcarbamothioyl)-*N'*-cyclohexylbenzimidamide (76a)**.

N-cyclohexylbenzimidoyl chloride **117b** (1.051 g, 4.7 mmol), NaNCS (422 mg, 5.2 mmol) and bezyl-amine **118** (568 µL, 5.2 mmol) were reacted according to general procedure D_2. The crude product was purified by flash silica gel chromatography (PE/EtOAc in 93/7 ratio) to achieve the intermediate imidoylthiourea **76a** as a light-yellow solid (375 mg, yield 23%). *E/Z* ^{1}H NMR (401 MHz, Chloro-form-*d*) δ 12.94 (d, *J* = 43.7 Hz, 2H), 7.64 (tt, *J* = 4.7, 2.5 Hz, 1H), 7.62 – 7.55 (m, 1H), 7.54 – 7.38 (m, 10H), 7.38 – 7.30 (m, 6H), 7.29 – 7.22 (m, 4H), 4.86 (d, 5.4 Hz, 2H), 4.77 (d, *J* = 5.5 Hz, 2H), 3.48 (m, 1H), 3.07 (tt, *J* = 8.9, 3.6 Hz, 1H), 1.84 (s, 2H), 1.75 (dd, *J* = 11.2, 5.5 Hz, 2H), 1.57 – 1.38 (m, 8H), 1.25 – 1.15 (m, 4H), 1.09 (d, *J* = 6.5 Hz, 4H) ppm.

***N*-benzyl-2-cyclohexyl-3-phenyl-1,2,4-thiadiazol-5(2*H*)-imine hydrobromide (76b).**

N-(benzylcarbamothioyl)-*N'*-cyclohexylbenzimidamide **76a** (190 mg, 0.54 mmol) and bromine (42 µL, 0.81 mmol) were reacted according to general procedure E. The precipitate was washed with a mixture of PE/EtOAc (2/1 v/v) to achieve the intermediate hydrobromide iminothiadiazole **76b** as a white solid (202 mg, yield 87%). ^{1}H NMR (401 MHz, DMSO-d_6) δ 10.28 (t, 1H), 7.77 – 7.73 (m, 2H), 7.73 – 7.69 (m, 1H), 7.68 – 7.63 (m, 2H), 7.47 – 7.36 (m, 4H), 7.35 – 7.30 (m, 1H), 4.83 (d, *J* = 5.2 Hz, 2H), 4.23 (tt, *J* = 11.5, 3.8 Hz, 1H), 2.20 – 2.11 (m, 2H), 1.80 – 1.52 (m, 5H), 1.29 – 1.14 (m, 3H) ppm.

***N*-(benzylcarbamothioyl)-*N'*-(pyridin-4-yl)benzimidamide (77a)**.

(isothiocyanatomethyl)benzene **86** (350 µL, 2.64 mmol) and *N'*-(pyridin-4-yl)benzimidamide **84** (521 mg, 2.64 mmol) were reacted according to general procedure D_1. The crude product was purified by flash silica gel chromatography (DCM/MeOH in 99/1 ratio) to achieve the intermediate imidoylthio-urea **77a** as a white solid (222 mg, yield 24%). ^{1}H NMR (401 MHz, Chloroform-*d*) δ 11.88 (bs, 1H),

8.29 (dd, *J* = 6.0, 3.0 Hz, 2H), 8.21 (bs, 1H), 7.44 – 7.35 (m, 4H), 7.35 – 7.27 (m, 4H), 7.25 – 7.22 (m, 1H), 7.21 (d, *J* = 1.5 Hz, 1H), 6.54 (dd, *J* = 6.2, 3.0 Hz, 2H), 4.96 (d, *J* = 5.3 Hz, 2H) ppm.

***N*-benzyl-3-phenyl-2-(pyridin-4-yl)-1,2,4-thiadiazol-5(2*H*)-imine hydrobromide (77b).**

N-(benzylcarbamothioyl)-*N'*-(pyridin-4-yl)benzimidamide **77a** (277 mg, 0.8 mmol) and bromine (82 µL, 1.6 mmol) were reacted according to general procedure E. The precipitate was washed with a mixture of PE/EtOAc (2/1 v/v) to achieve the intermediate hydrobromide iminothiadiazole **77b** as a yellow solid (340 mg, yield 100%). ^{1}H NMR (401 MHz, DMSO-d_6) δ 10.55 (t, *J* = 6.1 Hz, 1H), 8.80 (dd, *J* = 6.4, 3.2 Hz, 2H), 7.68 (dd, *J* = 6.3, 3.3 Hz, 2H), 7.64 – 7.58 (m, 1H), 7.58 – 7.53 (m, 2H), 7.51 – 7.39 (m, 7H), 7.38 – 7.32 (m, 1H), 5.01 (d, *J* = 6.0 Hz, 2H) ppm.

***N*-(benzylcarbamothioyl)-*N'*-(pyridin-3-yl)benzimidamide (78a)**.

(isothiocyanatomethyl)benzene **86** (1.173 mL, 8.84 mmol) and *N'*-(pyridin-3-yl)benzimidamide **85** (1.744 g, 8.84 mmol) were reacted according to general procedure D_1. The crude product was purified by flash silica gel chromatography (DCM/MeOH in 99.5/0.5 ratio) to achieve the intermediate im-idoylthiourea **78a** as a white solid (582 mg, yield 19%). ^{1}H NMR (401 MHz, Chloroform-*d*) δ 12.12 (bs, 1H), 8.23 – 8.13 (m, 2H), 7.96 (dd, *J* = 2.5, 0.8 Hz, 1H), 7.44 – 7.27 (m, 8H), 7.24 – 7.19 (m, 2H), 7.03 (*pseudo* dd, *J* = 8.1, 4.7, 1H), 6.91 (*pseudo* ddd, *J* = 8.1, 2.6, 1.5 Hz, 1H), 4.97 (d, *J* = 5.3 Hz, 2H).

***N*-benzyl-3-phenyl-2-(pyridin-3-yl)-1,2,4-thiadiazol-5(2*H*)-imine hydrobromide (78b).**

N-(benzylcarbamothioyl)-*N'*-(pyridin-3-yl)benzimidamide **78a** (570 mg, 1.65 mmol) and bromine (169 µL, 3.29 mmol) were reacted according to general procedure E. The precipitate was washed with a mixture of PE/EtOAc (2/1 v/v) to achieve the intermediate hydrobromide iminothiadiazole **78b** as a yellow solid (701 mg, yield 100%). ^{1}H NMR (401 MHz, DMSO-d_6) δ 10.46 (t, *J* = 6.0 Hz, 1H), 8.79 (d, *J* = 2.5 Hz, 1H), 8.73 (dd, *J* = 4.8, 1.5 Hz, 1H), 8.13 (dt, *J* = 8.3, 1.9 Hz, 1H), 7.62 (dd,

J = 8.2, 4.8 Hz, 1H), 7.59 – 7.51 (m, 3H), 7.51 – 7.39 (m, 6H), 7.38 – 7.32 (m, 1H), 5.00 (d, *J* = 5.9 Hz, 2H) ppm.

***N*-phenylbenzamide (108a).**

Aniline **104** (489 µL, 5.25 mmol), benzoyl chloride **97** (580 µL, 5 mmol) and TEA (768 µL, 5.5 mmol) were reacted according to general procedure F. The precipitate was removed from the reaction mixture and the solvent evaporated from the filtrate. The solid residue was washed with pentane and filtered to afford the desired amide **108a** as a white solid (986 mg, yield 100%). ^{1}H NMR (401 MHz, Chloroform-*d*) δ 7.91 – 7.85 (m, 2H), 7.81 (bs, 1H), 7.68 – 7.62 (m, 2H), 7.59 – 7.52 (m, 1H), 7.52 – 7.44 (m, 2H), 7.42 – 7.33 (m, 2H), 7.16 (ddt, *J* = 8.6, 7.2, 1.2 Hz, 1H) ppm.

***N*-phenylbenzimidoyl chloride (108b).**

N-phenylbenzamide **108a** (980 mg, 4.97 mmol) and thionylchloride (1.558 mL, 21.36 mmol) were reacted according to general procedure G. The remaining thionyl chloride was removed in *vacuum* to afford the desired imidoyl chloride **108b** as a white solid (986 mg, yield 92%). ^{1}H NMR (401 MHz, Chloroform-*d*) δ 8.17 (dt, *J* = 7.2, 1.4 Hz, 2H), 7.59 – 7.51 (m, 1H), 7.48 (dd, *J* = 8.3, 6.9 Hz, 2H), 7.45 – 7.34 (m, 2H), 7.21 (td, *J* = 7.5, 1.2 Hz, 1H), 7.02 (dt, *J* = 8.2, 1.2 Hz, 2H) ppm.

4-methoxy-*N*-phenylbenzamide (109a).

Aniline **104** (489 µL, 5.25 mmol), 4-methoxybenzoyl chloride **98** (677 µL, 5 mmol) and TEA (768 µL, 5.5 mmol) were reacted according to general procedure F. The precipitate was removed from the reaction mixture and the solvent evaporated from the filtrate. The solid residue was washed with pentane and filtered to afford the desired amide **109a** as a light yellow solid (1.067 g, yield 94%). ^{1}H NMR (401 MHz, Chloroform-*d*) δ 7.87 – 7.82 (m, 2H), 7.80 (bs, 1H), 7.66 – 7.60 (m, 2H), 7.40 – 7.33 (m, 2H), 7.14 (tt, *J* = 7.2, 1.1 Hz, 1H), 7.00 – 6.93 (m, 2H), 3.87 (s, 3H) ppm.

4-methoxy-*N*-phenylbenzimidoyl chloride (109b).

4-methoxy-*N*-phenylbenzamide **109a** (1.03 g, 4.53 mmol) and thionylchloride (1.42 mL, 19.48 mmol) were reacted according to general procedure G. The remaining thionyl chloride was removed in *vacuum* to afford the desired imidoyl chloride **109b** as a light-brown solid (1.054 g, yield 95%). ^{1}H NMR (401 MHz, Chloroform-*d*) δ 8.16 – 8.09 (m, 2H), 7.43 – 7.37 (m, 2H), 7.20 (t, *J* = 8.2 Hz, 1H), 7.02 – 6.97 (m, 4H), 3.89 (s, 3H) ppm.

3-methoxy-*N*-phenylbenzamide (110a).

Aniline **104** (489 µL, 5.25 mmol), 3-methoxybenzoyl chloride **99** (704 µL, 5 mmol) and TEA (768 µL, 5.5 mmol) were reacted according to general procedure F. The precipitate was removed from the reaction mixture and the solvent evaporated from the filtrate. The solid residue was washed with pentane and filtered to afford the desired amide **110a** as a light yellow solid (970 mg, yield 85%). ^{1}H NMR (401 MHz, Chloroform-*d*) δ 7.83 (bs, 1H), 7.67 – 7.61 (m, 2H), 7.46 – 7.42 (m, 1H), 7.41 – 7.34 (m, 4H), 7.16 (td, *J* = 7.4, 1.2 Hz, 1H), 7.12 – 7.05 (m, 1H), 3.87 (s, 3H) ppm.

3-methoxy-*N*-phenylbenzimidoyl chloride (110b).

3-methoxy-*N*-phenylbenzamide **110a** (943 mg, 4.15 mmol) and thionylchloride (1.211 mL, 16.6 mmol) were reacted according to general procedure G. The remaining thionyl chloride was removed in *vacuum* to afford the desired imidoyl chloride **110b** as a yellow solid (846 mg, yield 83%). ^{1}H NMR (401 MHz, Chloroform-*d*) δ 7.77 (ddd, *J* = 7.8, 1.8, 0.9 Hz, 1H), 7.71 (dd, *J* = 2.6, 1.7 Hz, 1H), 7.45 – 7.36 (m, 3H), 7.24 – 7.18 (m, 1H), 7.10 (ddd, *J* = 8.3, 2.7, 1.0 Hz, 1H), 7.04 – 6.99 (m, 2H), 3.89 (s, 3H) ppm.

2-methoxy-*N*-phenylbenzamide (111a).

Aniline **104** (489 µL, 5.25 mmol), 2-methoxybenzoyl chloride **100** (704 µL, 5 mmol) and TEA (768 µL, 5.5 mmol) were reacted according to general procedure F. The precipitate was removed from the reaction mixture and the solvent evaporated from the filtrate. The solid residue was washed with pentane and filtered to afford the desired amide **111a** as a light yellow solid (1.135 mg, yield 100%). ^{1}H NMR (401 MHz, Chloroform-*d*) δ 9.77 (bs, 1H), 8.30 (dd, *J* = 7.8, 1.9 Hz, 1H), 7.72 – 7.64 (m, 2H), 7.49 (ddd, *J* = 8.3, 7.3, 1.9 Hz, 1H), 7.40 – 7.32 (m, 2H), 7.18 – 7.09 (m, 2H), 7.03 (d, *J* = 8.3 Hz, 1H), 4.06 (s, 3H) ppm.

2-methoxy-N-phenylbenzimidoyl chloride (111b).

An equimolar mixture of 2-methoxy-*N*-phenylbenzamide **111a** (1.269 g, 5.6 mmol) and phosphorus pentachloride (1.163g, 5.6 mmol) was heated in toluene (5 mL) at reflux temperature for 4 h. Then, the reaction mixture was cooled to room temperature and the solids formed were filtered off. The solvent was removed under reduced pressure from the filtrate to afford the desired imidoyl chloride **111b** as a brown oil (1.112 g, yield 81%), which was used in the next step without further purification. ^{1}H NMR (401 MHz, Chloroform-*d*) δ 7.61 (dd, *J* = 7.7, 1.8 Hz, 1H), 7.44 – 7.33 (m, 4H), 7.22 – 7.17 (m, 1H), 7.07 – 7.02 (m, 3H), 3.92 (s, 3H) ppm.

***N*-phenylisonicotinamide (112a).**

Aniline **104** (489 µL, 5.25 mmol), Isonicotinoyl chloride hydrochloride **101** (890 mg, 5 mmol) and TEA (1.396 mL, 10 mmol) were reacted according to general procedure F. The precipitate was removed from the reaction mixture and the solvent evaporated from the filtrate. The solid residue was washed with pentane and filtered to afford the desired amide **112a** as a white solid (956 mg, yield 96%). ^{1}H NMR (401 MHz, Chloroform-*d*) δ 8.80 (d, *J* = 6.1 Hz, 2H), 7.94 (bs, 1H), 7.72 (d, *J* = 6.1 Hz, 2H), 7.64 (d, *J* = 8.0 Hz, 2H), 7.40 (t, *J* = 7.8 Hz, 2H), 7.20 (t, *J* = 7.6 Hz, 1H) ppm.

N-phenylisonicotinimidoyl chloride hydrochloride (112b).

N-phenylisonicotinamide **112a** (946 mg, 4.8 mmol) and thionylchloride (1.497 mL, 20.5 mmol) were reacted according to general procedure G. The remaining thionyl chloride was removed in *vacuum* to afford the desired imidoyl chloride **112b** as a yellow solid (927 mg, yield 76%). ^{1}H NMR (401 MHz, Chloroform-*d*) δ 8.96 (d, *J* = 6.4 Hz, 2H), 8.66 (d, *J* = 6.2 Hz, 2H), 7.54 – 7.45 (m, 2H), 7.36 (tt, *J* = 7.5, 1.1 Hz, 1H), 7.24 – 7.15 (m, 2H) ppm.

N-phenylnicotinamide (113a).

Aniline **104** (489 µL, 5.25 mmol), nicotinoyl chloride hydrochloride **102** (890 mg, 5 mmol) and TEA (1.396 mL, 10 mmol) were reacted according to general procedure F. The precipitate was removed from the reaction mixture and the solvent evaporated from the filtrate. The solid residue was washed with pentane and filtered to afford the desired amide **113a** as a white solid (854 mg, yield 86%). ^{1}H NMR (401 MHz, DMSO-d_6) δ 10.45 (bs, 1H), 9.11 (dd, *J* = 2.3, 0.9 Hz, 1H), 8.76 (dd, *J* = 4.8, 1.7 Hz, 1H), 8.30 (ddd, *J* = 7.9, 2.4, 1.7 Hz, 1H), 7.83 – 7.73 (m, 2H), 7.57 (ddd, *J* = 8.0, 4.8, 0.9 Hz, 1H), 7.41 – 7.32 (m, 2H), 7.13 (ddt, *J* = 8.6, 7.4, 1.2 Hz, 1H) ppm.

N-phenylnicotinimidoyl chloride hydrochloride (113b).

N-phenylnicotinamide **113a** (825 mg, 4.16 mmol) and thionylchloride (1.306 mL, 17.9 mmol) were reacted according to general procedure G. The remaining thionyl chloride was removed in *vacuum* to afford the desired imidoyl chloride **113b** as a yellow solid (810 mg, yield 77%). ^{1}H NMR (401 MHz, DMSO-d_6) δ 9.05 (s, 1H), 8.47 – 8.40 (m, 2H), 7.87 – 7.79 (m, 2H), 7.43 – 7.36 (m, 3H), 7.21 – 7.15 (m, 1H) ppm.

N-phenylfuran-2-carboxamide (114a).

Aniline **104** (580 µL, 6.3 mmol), 2-furoyl chloride **103** (590 µL, 6 mmol) and TEA (920 µL, 6.6 mmol) were reacted according to general procedure F. The precipitate was removed from the reaction

mixture and the solvent evaporated from the filtrate. The solid residue was washed with pentane and filtered to afford the desired amide **114a** as a white solid (951 mg, yield 85%). ^{1}H NMR (401 MHz, Chloroform-*d*) δ 8.17 – 7.98 (bs, 1H), 7.65 (dt, *J* = 8.6, 1.0 Hz, 2H), 7.51 (dd, *J* = 1.8, 1.0 Hz, 1H), 7.36 (td, *J* = 7.4, 1.5 Hz, 2H), 7.24 (d, *J* = 3.4 Hz, 1H), 7.15 (td, *J* = 7.4, 1.2 Hz, 1H), 6.60 – 6.52 (m, 1H) ppm.

***N*-phenylfuran-2-carbimidoyl chloride (114b).**

N-phenylfuran-2-carboxamide **114a** (739 mg, 3.95 mmol) and thionylchloride (1.24 mL, 17 mmol) were reacted according to general procedure G. The remaining thionyl chloride was removed in vacuum to afford the desired imidoyl chloride **114b** as a brown solid (720 mg, yield 89%). ^{1}H NMR (401 MHz, Chloroform-*d*) δ 7.64 (dt, *J* = 1.8, 0.9 Hz, 1H), 7.44 – 7.35 (m, 2H), 7.24 – 7.17 (m, 2H), 7.06 (dq, *J* = 7.2, 1.1 Hz, 2H), 6.57 (ddd, *J* = 3.5, 1.8, 0.9 Hz, 1H) ppm.

***N*-(*m*-tolyl)benzamide (115a).**

m-toluidine **105** (563 µL, 5.25 mmol), benzoyl chloride **97** (580 µL, 5 mmol) and TEA (768 µL, 5.5 mmol) were reacted according to general procedure F. The precipitate was removed from the reaction mixture and the solvent evaporated from the filtrate. The solid residue was washed with pentane and filtered to afford the desired amide **115a** as a grey solid (960 mg, yield 91%). ^{1}H NMR (401 MHz, Chloroform-*d*) δ 7.86 (d, *J* = 8.2 Hz, 2H), 7.83 (bs, 1H), 7.57 – 7.45 (m, 4H), 7.42 (d, *J* = 8.0 Hz, 1H), 7.25 (t, *J* = 7.8 Hz, 1H), 6.97 (d, *J* = 7.6 Hz, 1H), 2.37 (s, 3H) ppm.

***N*-(*m*-tolyl)benzimidoyl chloride (115b).**

N-(*m*-tolyl)benzamide **115a** (920 mg, 4.4 mmol) and thionylchloride (1.364 mL, 18.7 mmol) were reacted according to general procedure G. The remaining thionyl chloride was removed in vacuum to afford the desired imidoyl chloride **115b** as a white solid (994 mg, yield 98%). ^{1}H NMR (401 MHz,

Chloroform-*d*) δ 8.22 – 8.16 (m, 2H), 7.59 – 7.52 (m, 1H), 7.52 – 7.46 (m, 2H), 7.31 (t, *J* = 7.6 Hz, 1H), 7.04 (d, *J* = 7.6 Hz, 1H), 6.88 – 6.81 (m, 2H), 2.41 (s, 3H) ppm.

***N*-benzylbenzamide (116a).**

Benzylamine **106** (573 µL, 5.25 mmol), benzoyl chloride **97** (580 µL, 5 mmol) and TEA (768 µL, 5.5 mmol) were reacted according to general procedure F. The precipitate was removed from the reaction mixture and the solvent evaporated from the filtrate. The solid residue was washed with pentane and filtered to afford the desired amide **116a** as a white solid (1.062 g, yield 100%). ^{1}H NMR (401 MHz, Chloroform-*d*) δ 7.84 – 7.74 (m, 2H), 7.53 – 7.46 (m, 1H), 7.46 – 7.39 (m, 2H), 7.38 – 7.34 (m, 4H), 7.34 – 7.28 (m, 1H), 6.43 (bs, 1H), 4.65 (d, *J* = 5.6 Hz, 2H) ppm.

***N*-benzylbenzimidoyl chloride (116b).**

N-benzylbenzamide **116a** (1.052 g, 5 mmol) and thionylchloride (1.460 mL, 20 mmol) were reacted according to general procedure G. The remaining thionyl chloride was removed in vacuum to afford the desired imidoyl chloride **116b** as a yellow liquid (1.111 g, yield 97%). ^{1}H NMR (401 MHz, Chloroform-*d*) δ 8.11 – 8.05 (m, 2H), 7.52 – 7.40 (m, 5H), 7.40 – 7.34 (m, 2H), 7.32 – 7.28 (m, 1H), 4.95 (s, 2H) ppm.

***N*-cyclohexylbenzamide (117a).**

Cyclohexylamine **107** (570 µL, 5 mmol), benzoyl chloride **97** (580 µL, 5 mmol) and TEA (768 µL, 5.5 mmol) were reacted according to general procedure F. The precipitate was removed from the reaction mixture and the solvent evaporated from the filtrate. The solid residue was washed with pentane and filtered to afford the desired amide **117a** as a pink solid (1.037 g, yield 100%). ^{1}H NMR (401 MHz, Chloroform-*d*) δ 7.77 – 7.72 (m, 2H), 7.51 – 7.45 (m, 1H), 7.44 – 7.38 (m, 2H), 5.96 (bs, 1H), 3.99 (dddd, *J* = 14.6, 10.6, 8.0, 3.9 Hz, 1H), 2.03 (dt, *J* = 12.3, 4.0 Hz, 2H), 1.82 – 1.71 (m, 2H), 1.70 – 1.60 (m, 1H), 1.50 – 1.37 (m, 2H), 1.31 – 1.15 (m, 3H) ppm.

***N*-cyclohexylbenzimidoyl chloride (117b).**

N-cyclohexylbenzamide **117a** (1.029 g, 5 mmol) and thionylchloride (1.460 mL, 20 mmol) were reacted according to general procedure G. The remaining thionyl chloride was removed in vacuum to afford the desired imidoyl chloride **117b** as a brown oil (1.054 g, yield 95%). ^{1}H NMR (401 MHz, Chloroform-*d*) δ 8.02 (d, *J* = 7.6 Hz, 2H), 7.51 – 7.43 (m, 1H), 7.43 – 7.36 (m, 2H), 3.91 (tt, *J* = 10.1, 3.6 Hz, 1H), 1.94 – 1.79 (m, 4H), 1.72 – 1.56 (m, 3H), 1.49 – 1.30 (m, 3H) ppm.

1-benzyl-3-(pyridin-2-yl)thiourea (132a).

(isothiocyanatomethyl)benzene **86** (530 µL, 4 mmol) and pyridin-2-amine **130** (376 mg, 4 mmol) were reacted according to general procedure D_1. The crude product was purified by flash silica gel chromatography (PE/EtOAc in 90/10 ratio) to achieve the intermediate thiourea **132a** as a white solid (659 mg, yield 68%). ^{1}H NMR (401 MHz, Chloroform-*d*) δ 12.06 (s, 1H), 8.82 (s, 1H), 8.09 (d, *J* = 4.8 Hz, 1H), 7.65 (t, *J* = 7.2 Hz, 1H), 7.44 – 7.39 (m, 2H), 7.36 (t, *J* = 7.4 Hz, 2H), 7.32 – 7.27 (m, 1H), 6.95 (t, *J* = 6.3 Hz, 1H), 6.84 (s, 1H), 5.00 (d, *J* = 5.4 Hz, 2H) ppm.

2-(benzylamino)-[1,2,4]thiadiazolo[2,3-*a*]pyridin-4-ium bromide (132b).

1-benzyl-3-(pyridin-2-yl)thiourea **132a** (407 mg, 1.67 mmol) and bromine (102 µL, 2 mmol) were reacted according to general procedure E. The precipitate was washed with a mixture of PE/EtOAc (2/1 v/v) to achieve the intermediate pyridinium bromide **132b** as a white solid (381 mg, yield 71%). ^{1}H NMR (401 MHz, DMSO-d_6) δ 10.04 (t, *J* = 5.9 Hz, 1H), 9.11 (d, *J* = 6.6 Hz, 1H), 8.10 (t, *J* = 7.9 Hz, 1H), 7.78 (d, *J* = 8.7 Hz, 1H), 7.44 – 7.34 (m, 5H), 7.31 (t, *J* = 6.8 Hz, 1H), 4.85 (d, *J* = 5.8 Hz, 2H) ppm.

1-benzyl-3-(4-(2-methoxyethyl)pyridin-2-yl)thiourea (133a).

(isothiocyanatomethyl)benzene **86** (262 µL, 2 mmol) and 4-(2-methoxyethyl)pyridin-2-amine **131** (300 mg, 2 mmol) were reacted according to general procedure D_1. The crude product was purified by flash silica gel chromatography (DCM/MeOH in 98/2 ratio) to achieve the intermediate thiourea **133a** as a white solid (600 mg, yield 100%). ^{1}H NMR (401 MHz, DMSO-d_6) δ 12.13 (t, J = 5.7 Hz, 1H), 10.62 (s, 1H), 8.06 (d, J = 5.3 Hz, 1H), 7.39 – 7.23 (m, 5H), 7.07 (dd, J = 1.6, 0.8 Hz, 1H), 6.93 (dd, J = 5.4, 1.4 Hz, 1H), 4.91 (d, J = 5.7 Hz, 2H), 3.55 (t, J = 6.3 Hz, 2H), 3.23 (s, 3H), 2.77 (t, J = 6.3 Hz, 2H) ppm.

2-(benzylamino)-7-(2-methoxyethyl)-[1,2,4]thiadiazolo[2,3-*a*]pyridin-4-ium bromide (133b).

1-benzyl-3-(4-(2-methoxyethyl)pyridin-2-yl)thiourea **133a** (608 mg, 2 mmol) and bromine (155 µL, 3 mmol) were reacted according to general procedure E. The precipitate was washed with a mixture of PE/EtOAc (2/1 v/v) to achieve the intermediate pyridinium bromide **133b** as a white solid (710 mg, yield 93%). ^{1}H NMR (401 MHz, DMSO-d_6) δ 9.96 (t, J = 5.9 Hz, 1H), 8.99 (d, J = 6.8 Hz, 1H), 7.68 (d, J = 1.4 Hz, 1H), 7.45 – 7.34 (m, 4H), 7.36 – 7.27 (m, 2H), 4.84 (d, J = 5.9 Hz, 2H), 3.64 (t, J = 6.2 Hz, 2H), 3.24 (s, 3H), 2.97 (t, J = 6.2 Hz, 2H) ppm.

2,2,2-trifluoro-*N*-(5-methyl-1,3,4-thiadiazol-2-yl)acetamide (134b).

5-methyl-1,3,4-thiadiazol-2-amine **134a** (1 g, 8.7 mmol) and trifluoroacetic anhydride (1.45 mL, 10.45 mmol) were reacted according to general procedure H to afford the desired intermediate **134b** as an orange solid (1.647 g, yield 90%). ^{1}H NMR (401 MHz, DMSO-d_6) δ 2.61 (s, 3H) ppm.

***N*-(3-benzyl-5-methyl-1,3,4-thiadiazol-2(3*H*)-ylidene)-2,2,2-trifluoroacetamide (134c).**

2,2,2-trifluoro-*N*-(5-methyl-1,3,4-thiadiazol-2-yl)acetamide **134b** (1.714 g, 8.1 mmol), K_2CO_3 (1.340 g, 9.7 mmol) and (bromomethyl)benzene **137** (1.15 mL, 9.7 mmol) were reacted according to general procedure I. The desired intermediate **134c** was obtained as a yellow liquid (2.44 mg, yield 100%). ^{1}H NMR (401 MHz, DMSO-d_6) δ 7.41 – 7.30 (m, 5H), 5.55 (s, 2H), 2.61 (s, 3H) ppm.

3-benzyl-5-methyl-1,3,4-thiadiazol-2(3*H*)-imine (134d).

N-(3-benzyl-5-methyl-1,3,4-thiadiazol-2(*3H*)-ylidene)-2,2,2-trifluoroacetamide **134c** (2.4 g, 8 mmol) was treated with aqueous 5% NaOH (13 mL, 16 mmol) according to general procedure J. The desired intermediate **134d** was obtained as a yellow liquid (1.642 g, yield 100%). ^{1}H NMR (401 MHz, DMSO-d_6) δ 7.94 (s, 1H), 7.37 – 7.30 (m, 2H), 7.29 – 7.23 (m, 3H), 4.95 (s, 2H), 2.22 (s, 3H) ppm.

2,2,2-trifluoro-*N*-(1,3,4-thiadiazol-2-yl)acetamide (135b).

1,3,4-thiadiazol-2-amine **135a** (500 mg, 5 mmol) and trifluoroacetic anhydride (835 µL, 6 mmol) were reacted according to general procedure H to afford the desired intermediate **135b** as an orange solid (853 mg, yield 87%). ^{1}H NMR (401 MHz, DMSO-d_6) δ 9.19 (s, 1H) ppm.

***N*-(3-benzyl-1,3,4-thiadiazol-2(3*H*)-ylidene)-2,2,2-trifluoroacetamide (135c).**

2,2,2-trifluoro-*N*-(1,3,4-thiadiazol-2-yl)acetamide **135b** (800 mg, 4 mmol), K_2CO_3 (664 mg, 4.8 mmol) and (bromomethyl)benzene **137** (570 µL, 4.8 mmol) were reacted according to general procedure I. The desired intermediate **135c** was obtained as a yellow liquid (1.149 g, yield 100%). ^{1}H NMR (401 MHz, DMSO-d_6) δ 9.24 (s, 1H), 7.39 – 7.30 (m, 5H), 5.62 (s, 2H) ppm.

3-benzyl-1,3,4-thiadiazol-2(3*H*)-imine (135d).

N-(3-benzyl-1,3,4-thiadiazol-2(*3H*)-ylidene)-2,2,2-trifluoroacetamide **135c** (1.15 g, 4 mmol) was treated with aqueous 5% NaOH (6.5 mL, 8 mmol) according to general procedure J. The desired intermediate **135d** was obtained as a yellow liquid (707 mg, yield 92%). ^{1}H NMR (401 MHz, DMSO-d_6) δ 8.06 (s, 1H), 8.04 (s, 1H), 7.35 – 7.30 (m, 2H), 7.29 – 7.24 (m, 3H), 5.01 (s, 2H) ppm.

2,2,2-trifluoro-*N*-(thiazol-2-yl)acetamide (136b).

Thiazol-2-amine **136a** (500 mg, 5 mmol) and trifluoroacetic anhydride (835 µL, 6 mmol) were reacted according to general procedure H to afford the desired intermediate **136b** as a yellow solid (980 mg, yield 100%). ^{1}H NMR (401 MHz, DMSO-d_6) δ 7.64 (d, J = 4.4 Hz, 1H), 7.32 (d, J = 4.4 Hz, 1H) ppm.

***N*-(3-benzylthiazol-2(3*H*)-ylidene)-2,2,2-trifluoroacetamide (136c).**

2,2,2-trifluoro-*N*-(thiazol-2-yl)acetamide **136b** (860 mg, 4.4 mmol), K_2CO_3 (732 mg, 5.3 mmol) and (bromomethyl)benzene **137** (630 µL, 5.3 mmol) were reacted according to general procedure I. The desired intermediate **136c** was obtained as a yellow liquid (1.26 g, yield 100%). ^{1}H NMR (401 MHz, DMSO-d_6) δ 7.90 (d, J = 4.6 Hz, 1H), 7.40 (d, J = 4.6 Hz, 1H), 7.39 – 7.30 (m, 5H), 5.46 (s, 2H) ppm.

3-benzylthiazol-2(3*H*)-imine (136d).

N-(3-benzylthiazol-2(*3H*)-ylidene)-2,2,2-trifluoroacetamide **136c** (1.26 g, 4.4 mmol) was treated with aqueous 5% NaOH (7.15 mL, 8.8 mmol) according to general procedure J. The desired intermediate **136d** was obtained as a yellow liquid (800 mg, yield 95%). ^{1}H NMR (401 MHz, DMSO-d_6) δ 7.56 (s, 1H), 7.36 – 7.25 (m, 5H), 6.80 (d, J = 5.0 Hz, 1H), 5.97 (d, J = 5.0 Hz, 1H), 4.82 (s, 2H) ppm.

Scheme S1: Synthesis of N'-(pyridinyl)benzimidamides 84 and 85

The commercially unavailable *N'*-(pyridinyl)benzimidamides **84** and **85** were synthetized through activation of the appropriate aryl amine **139** or **140** with NaH, followed by coupling with benzonitrile **138** (Scheme S1).

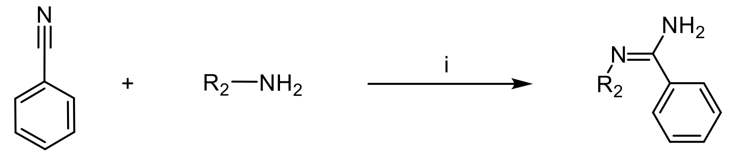

138 **139:** R_2= Pyridin-4-yl **140:** R_2= Pyridin-3-yl **84:** R_2= Pyridin-4-yl **85:** R_2= Pyridin-3-yl

Reagents and conditions: (i) NaH, dry DMF, 0°C, 30 min; then rt, 8-12h, yield 69-91%.

***N'*-(pyridin-4-yl)benzimidamide (84)**.

A solution of pyridin-4-amine **139** (376 mg, 4 mmol), in dry DMF (2 M) was treated with NaH (60% dispersion in mineral oil, 240 mg, 6 mmol) at 0 °C and stirred at the same temperature for 30 min. Then, benzonitrile **138** (619 µL, 6 mmol) was added to the reaction mixture, which was stirred at room temperature until TLC indicated the total consumption of the amine. The reaction was quenched with 5% aqueous $NaHCO_3$ and extracted with EtOAc (3x20mL). The combined organic layers were washed with brine (2x20mL), dried over Na_2SO_4, and then concentrated in *vacuum*. The crude product was triturated with EtOAc, filtered and washed to afford the intermediate benzimidamide **84** as a white crystalline solid (543 mg, yield 69%) ^{1}H NMR (401 MHz, Chloroform-*d*) δ 8.51 (d, *J* = 5.2 Hz, 2H), 7.87 (d, *J* = 7.4 Hz, 2H), 7.59 – 7.35 (m, 3H), 6.92 (d, *J* = 5.2 Hz, 2H), 4.92 (bs, 2H) ppm.

***N'*-(pyridin-3-yl)benzimidamide (85)**.

Pyridin-3-amine **140** (941 mg, 10 mmol), benzonitrile **138** (1.547 mL, 15 mmol) and NaH (600 mg, 15 mmol) were reacted following the procedure described for **84**. The intermediate benzimidamide **85** was obtained as a white crystalline solid (1.79 g, yield 91%). ^{1}H NMR (401 MHz, DMSO-*d*$_6$) δ 8.18 (d, *J* = 4.9 Hz, 1H), 8.11 (s, 1H), 7.98 (d, *J* = 7.4 Hz, 2H), 7.54 – 7.37 (m, 3H), 7.31 (dd, *J* = 8.0, 4.6 Hz, 1H), 7.24 (d, *J* = 8.1 Hz, 1H), 6.57 (bs, 2H) ppm.

Scheme S2: Synthesis of Isothiocyanates 89-96.

Isothiocyanates **89-96**, not commercially available, were prepared by treating the appropriate primary amines **141-148** with the thiocarbonyl transfer reagent 1,1′-Thiocarbonyldiimidazole (TCDI) **149** in the presence of TEA (Scheme S2).

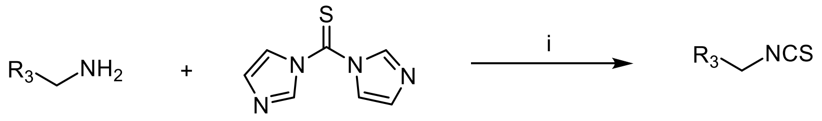

141: R_3= 4-MePh
142: R_3= 4-OMePh
143: R_3= 4-FPh
144: R_3= 4-ClPh
145: R_3= 3-OMePh
146: R_3= 3-ClPh
147: R_3= 2-OMePh
148: R_3= 2-ClPh

149
TDCI

89: R_3= 4-MePh
90: R_3= 4-OMePh
91: R_3= 4-FPh
92: R_3= 4-ClPh
93: R_3= 3-OMePh
94: R_3= 3-ClPh
95: R_3= 2-OMePh
96: R_3= 2-ClPh

Reagents and conditions: (i) TEA, dry DMF, 50°C, 6-7h, yield 36-79%.

1-(isothiocyanatomethyl)-4-methylbenzene (89).

TCDI **149** (1.604 g, 9.0 mmol) was dissolved in dry DMF (2 M) at 50°C. To this solution, a 1 M solution of *p*-tolylmethanamine **141** (764 µL, 6.0 mmol) and TEA (1.250 mL, 9.0 mmol) in DMF was added dropwise. The mixture was stirred at 50°C for 5-6h, then diluted with water and extracted with EtOAc (3x10mL). The combined organic phases were washed with water (2x20mL), dried over Na_2SO_4 and concentrated. The residue was purified via flash silica gel chromatography (PE/EtOAc in 99/1 ratio) to achieve the intermediate isothiocyanate **89** as a colorless liquid (774 mg, yield 79%). ^{1}H NMR (401 MHz, Chloroform-*d*) δ 7.20 (s, 4H), 4.67 (s, 2H), 2.36 (s, 3H) ppm.

1-(isothiocyanatomethyl)-4-methoxybenzene (90).

(4-methoxyphenyl)methanamine **142** (428 µL, 3.3 mmol), TCDI **149** (882 mg, 4.95 mmol) and TEA (688 µL, 4.95 mmol) were reacted following the procedure described for **89**. The crude product was purified by flash silica gel chromatography (PE/EtOAc in 99/1 ratio) to achieve the intermediate isothiocyanate **90** as a light-yellow liquid (458 mg, yield 77%). ^{1}H NMR (401 MHz, Chloroform-*d*) δ 7.24 (d, *J* = 8.9 Hz, 2H), 6.91 (d, *J* = 8.7 Hz, 2H), 4.64 (s, 2H), 3.82 (s, 3H) ppm.

1-fluoro-4-(isothiocyanatomethyl)benzene (91).

(4-fluorophenyl)methanamine **143** (572 µL, 5.0 mmol), TCDI **149** (1.337 g, 7.5 mmol) and TEA (1.042 mL, 7.5 mmol) were reacted following the procedure described for **89**. The crude product was purified by flash silica gel chromatography (PE/EtOAc in 99/1 ratio) to achieve the intermediate isothiocyanate **91** as a colorless liquid (494 mg, yield 59%). ^{1}H NMR (401 MHz, Chloroform-*d*) δ 7.33 – 7.27 (m, 2H), 7.08 (t, *J* = 8.6 Hz, 2H), 4.69 (s, 2H) ppm.

1-chloro-4-(isothiocyanatomethyl)benzene (92).

(4-chlorophenyl)methanamine **144** (403 µL, 3.3 mmol), TCDI **149** (882 mg, 4.95 mmol) and TEA (688 µL, 4.95 mmol) were reacted following the procedure described for **89**. The crude product was purified by flash silica gel chromatography (PE/EtOAc in 99/1 ratio) to achieve the intermediate isothiocyanate **92** as a light-yellow liquid (244 mg, yield 50%). ^{1}H NMR (401 MHz, Chloroform-*d*) δ 7.37 (d, *J* = 8.5 Hz, 2H), 7.26 (d, *J* = 8.1 Hz, 2H), 4.69 (s, 2H) ppm.

1-(isothiocyanatomethyl)-3-methoxybenzene (93).

(3-methoxyphenyl)methanamine **145** (1.932 mL, 15.0 mmol), TCDI **149** (4.011 g, 22.5 mmol) and TEA (3.126 mL, 22.5 mmol) were reacted following the procedure described for **89**. The crude product was purified by flash silica gel chromatography (PE/EtOAc in 99/1 ratio) to achieve the intermediate isothiocyanate **93** as a light-yellow liquid (1.517 g, yield 56%). ^{1}H NMR (401 MHz, Chloroform-*d*) δ 7.30 (t, *J* = 7.8 Hz, 1H), 6.91 – 6.84 (m, 3H), 4.69 (s, 2H), 3.83 (s, 3H) ppm.

1-chloro-3-(isothiocyanatomethyl)benzene (94).

(3-chlorophenyl)methanamine **146** (604 µL, 5.0 mmol), TCDI **149** (1.337 g, 7.5 mmol) and TEA (1.042 mL, 7.5 mmol) were reacted following the procedure described for **89**. The crude product was purified by flash silica gel chromatography (PE/EtOAc in 99/1 ratio) to achieve the

intermediate isothiocyanate **94** as a light-yellow liquid (659 mg, yield 72%). ^{1}H NMR (401 MHz, Chloroform-*d*) δ 7.32 (dd, *J* = 5.1, 4.0 Hz, 3H), 7.21 (dq, *J* = 5.7, 3.5, 2.3 Hz, 1H), 4.71 (s, 2H) ppm.

1-(isothiocyanatomethyl)-2-methoxybenzene (95).

(2-methoxyphenyl)methanamine **147** (649 µL, 5.0 mmol), TCDI **149** (1.337 g, 7.5 mmol) and TEA (1.042 mL, 7.5 mmol) were reacted following the procedure described for **89**. The crude product was purified by flash silica gel chromatography (PE/EtOAc in 99/1 ratio) to achieve the intermediate isothiocyanate **95** as a white crystalline solid (419 mg, yield 47%). ^{1}H NMR (401 MHz, Chloroform-*d*) δ 7.33 (t, *J* = 8.4 Hz, 2H), 6.98 (t, *J* = 7.5 Hz, 1H), 6.90 (d, *J* = 8.8 Hz, 1H), 4.70 (s, 2H), 3.86 (s, 3H) ppm.

1-chloro-2-(isothiocyanatomethyl)benzene (96).

(2-chlorophenyl)methanamine **148** (604 µL, 5.0 mmol), TCDI **149** (1.337 g, 7.5 mmol) and TEA (1.042 mL, 7.5 mmol) were reacted following the procedure described for **89**. The crude product was purified by flash silica gel chromatography (PE/EtOAc in 99/1 ratio) to achieve the intermediate isothiocyanate **96** as a colorless liquid (327 mg, yield 36%). ^{1}H NMR (401 MHz, Chloroform-*d*) δ 7.48 – 7.44 (m, 1H), 7.43 – 7.38 (m, 1H), 7.36 – 7.28 (m, 2H), 4.83 (s, 2H) ppm.

^{1}H-NMR spectrum, ^{13}C-NMR spectrum of final compounds 1-46

^{1}H-NMR spectrum (400 MHz, DMSO-d_6) of **1**

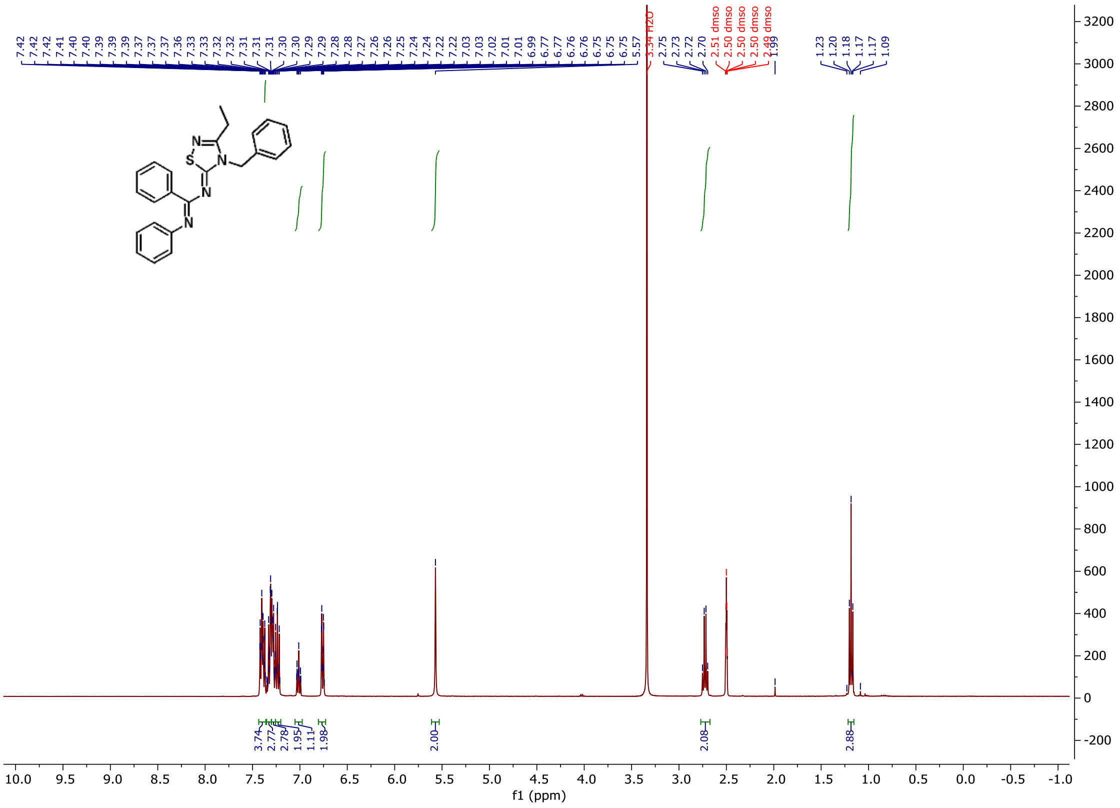

^{13}C-NMR spectrum (101 MHz, DMSO-d_6) of **1**

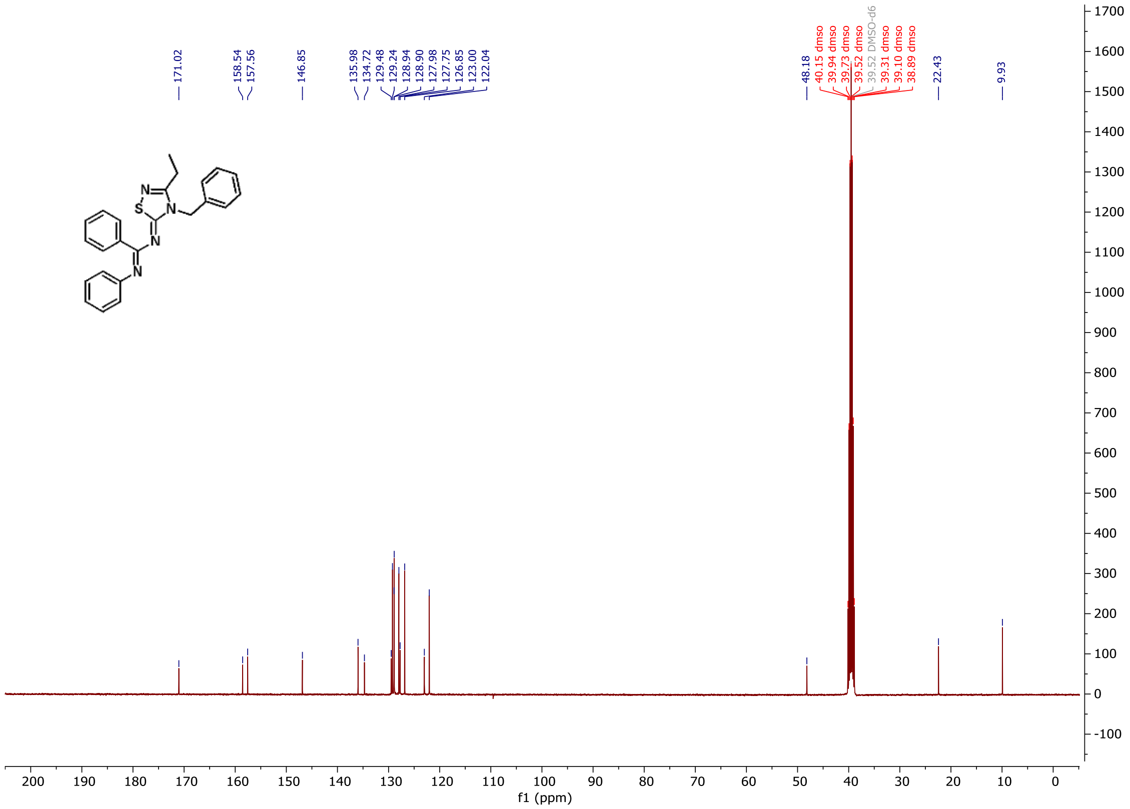

^{1}H-NMR spectrum (400 MHz, DMSO-d_6) of **2**

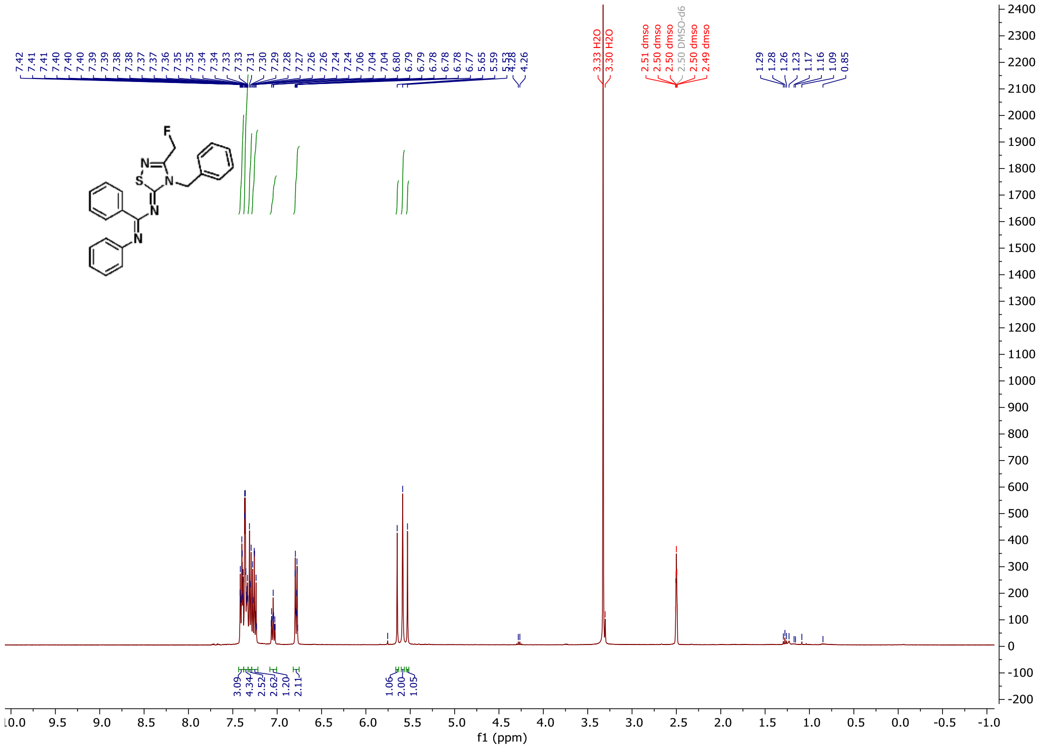

^{13}C-NMR spectrum (101 MHz, DMSO-d_6) of **2**

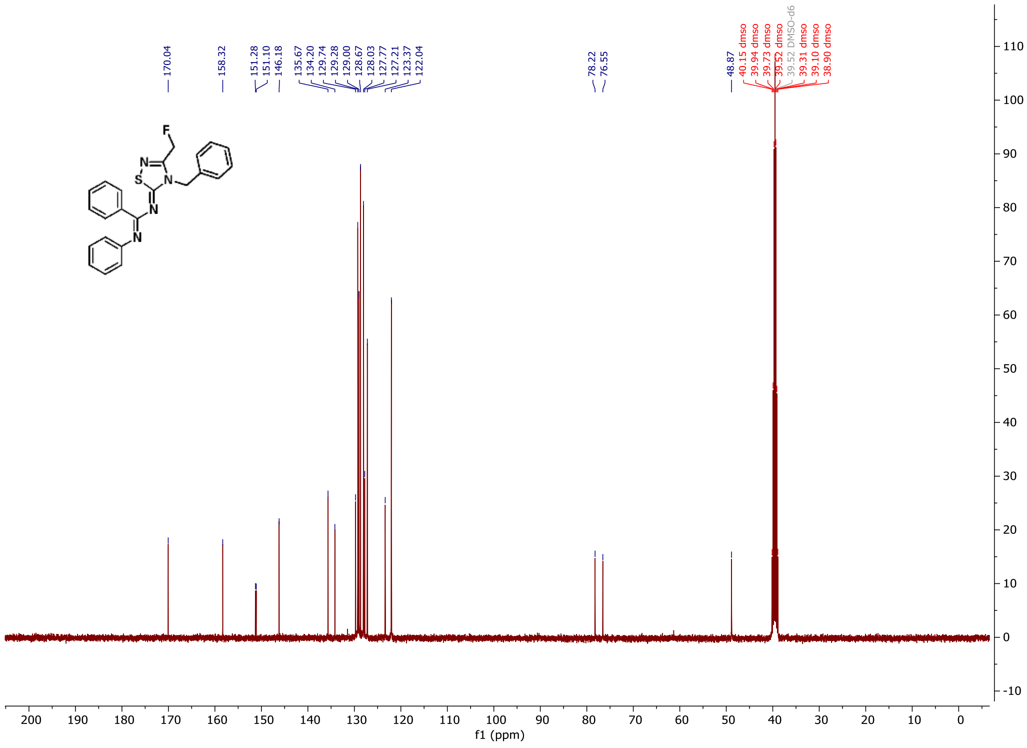

^{1}H-NMR spectrum (400 MHz, DMSO-d_6) of **3**

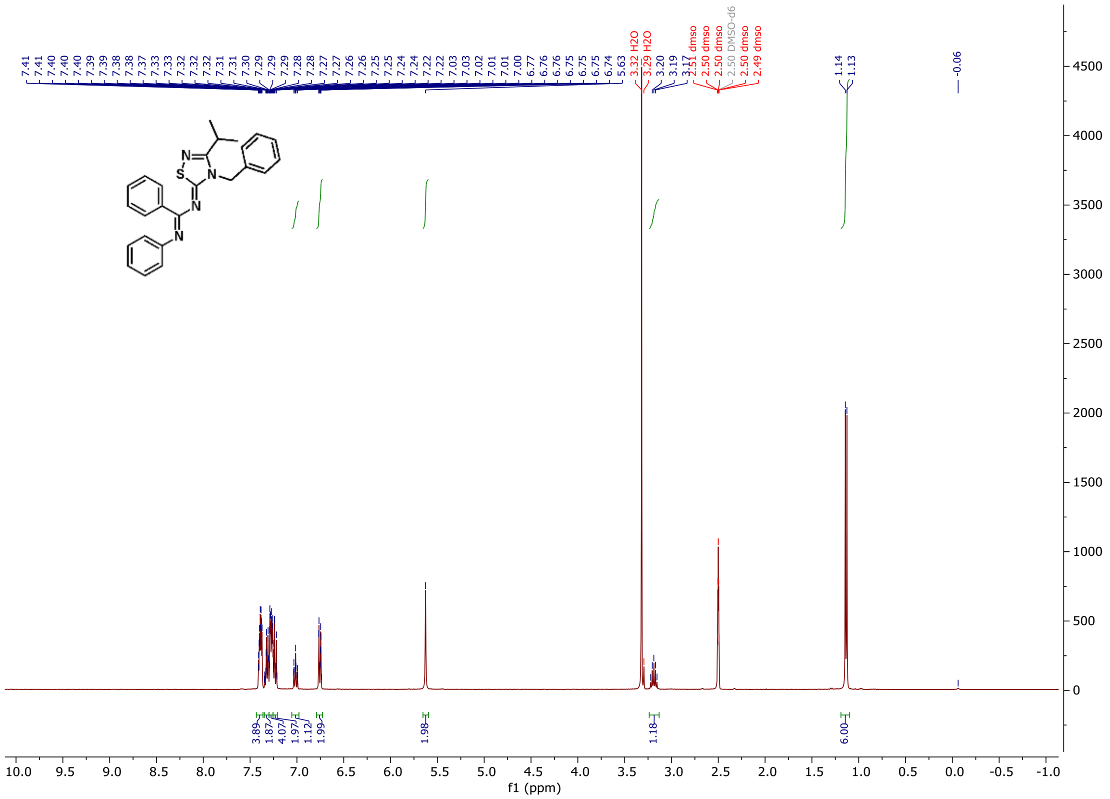

^{13}C-NMR spectrum (101 MHz, DMSO-d_6) of **3**

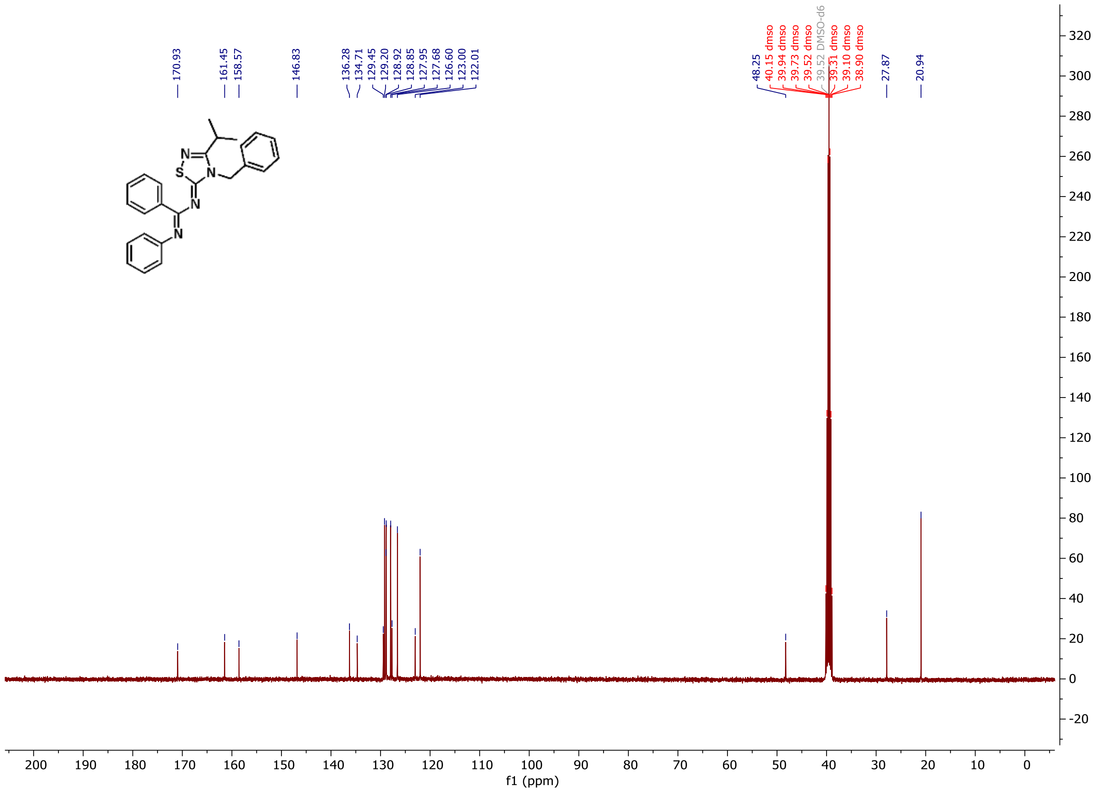

^{1}H-NMR spectrum (400 MHz, DMSO-d_6) of **4**

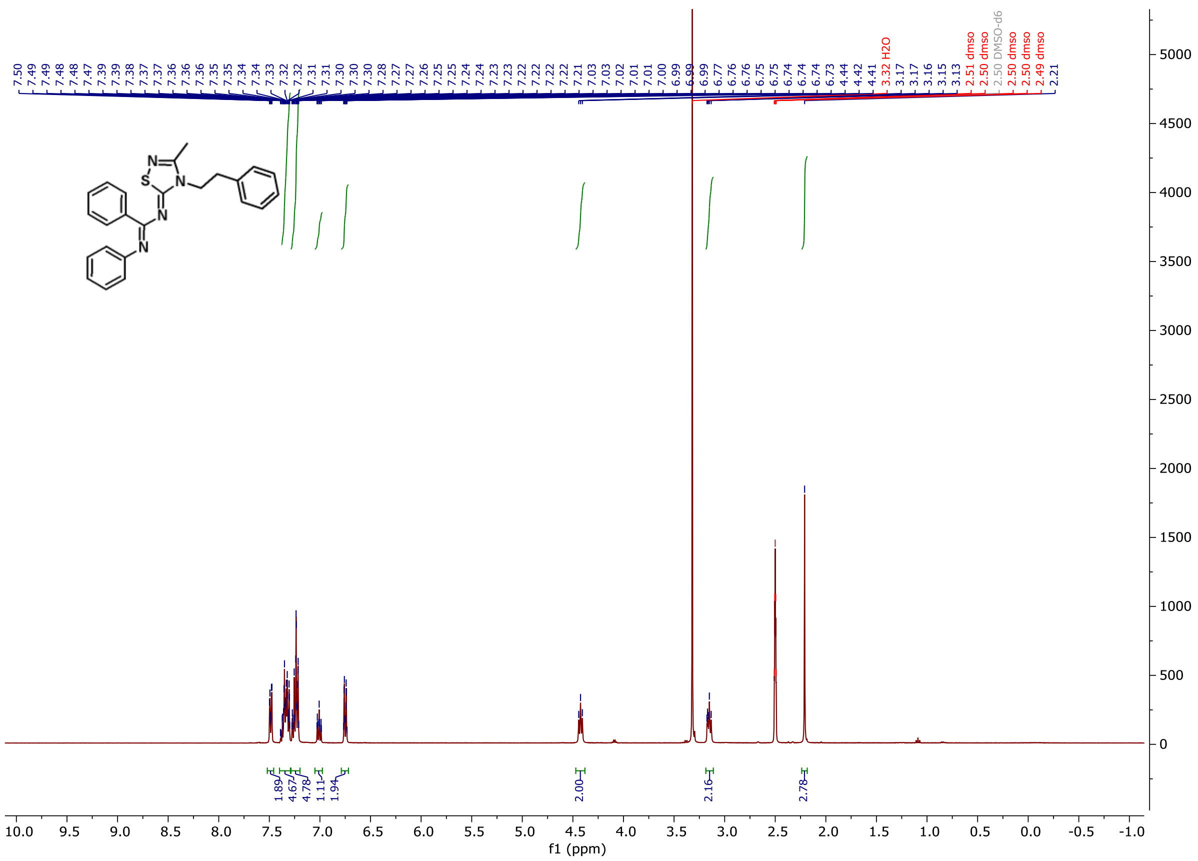

^{13}C-NMR spectrum (101 MHz, DMSO-d_6) of **4**

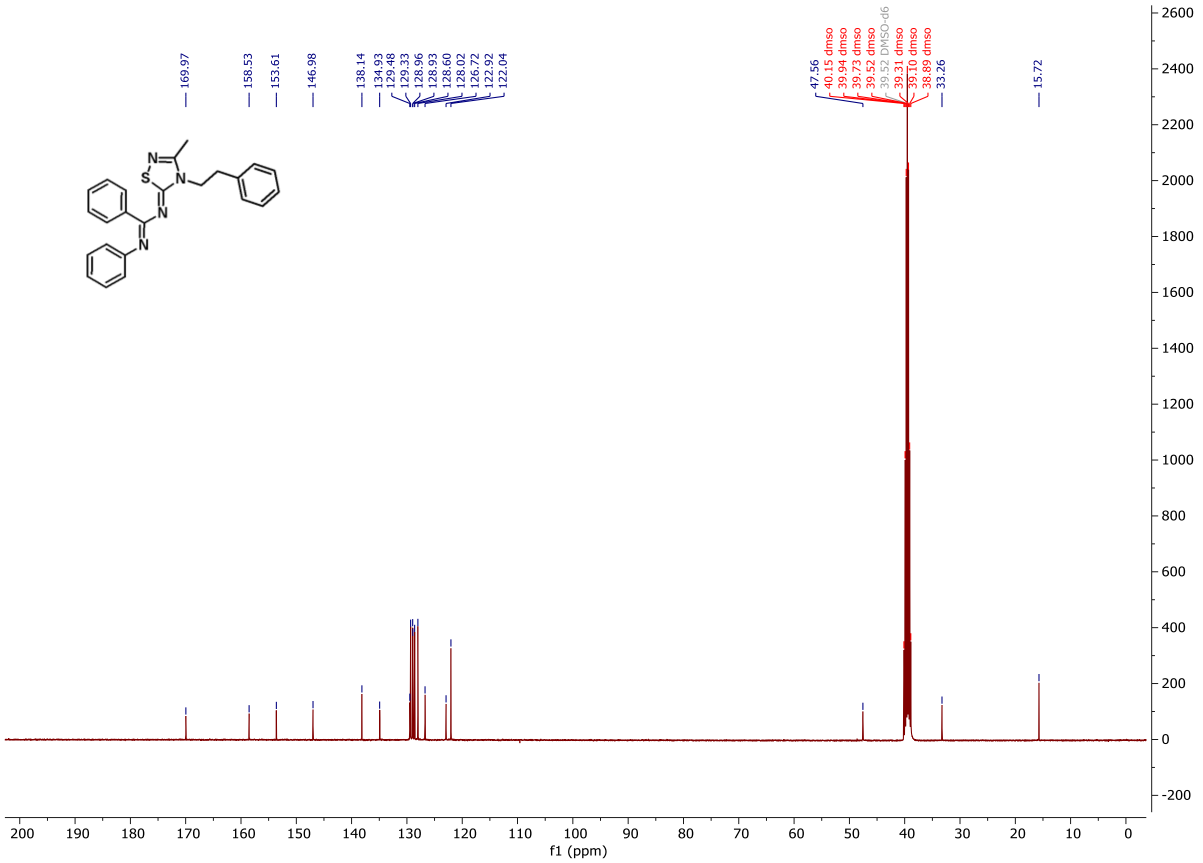

^{1}H-NMR spectrum (400 MHz, DMSO-d_6) of **5**

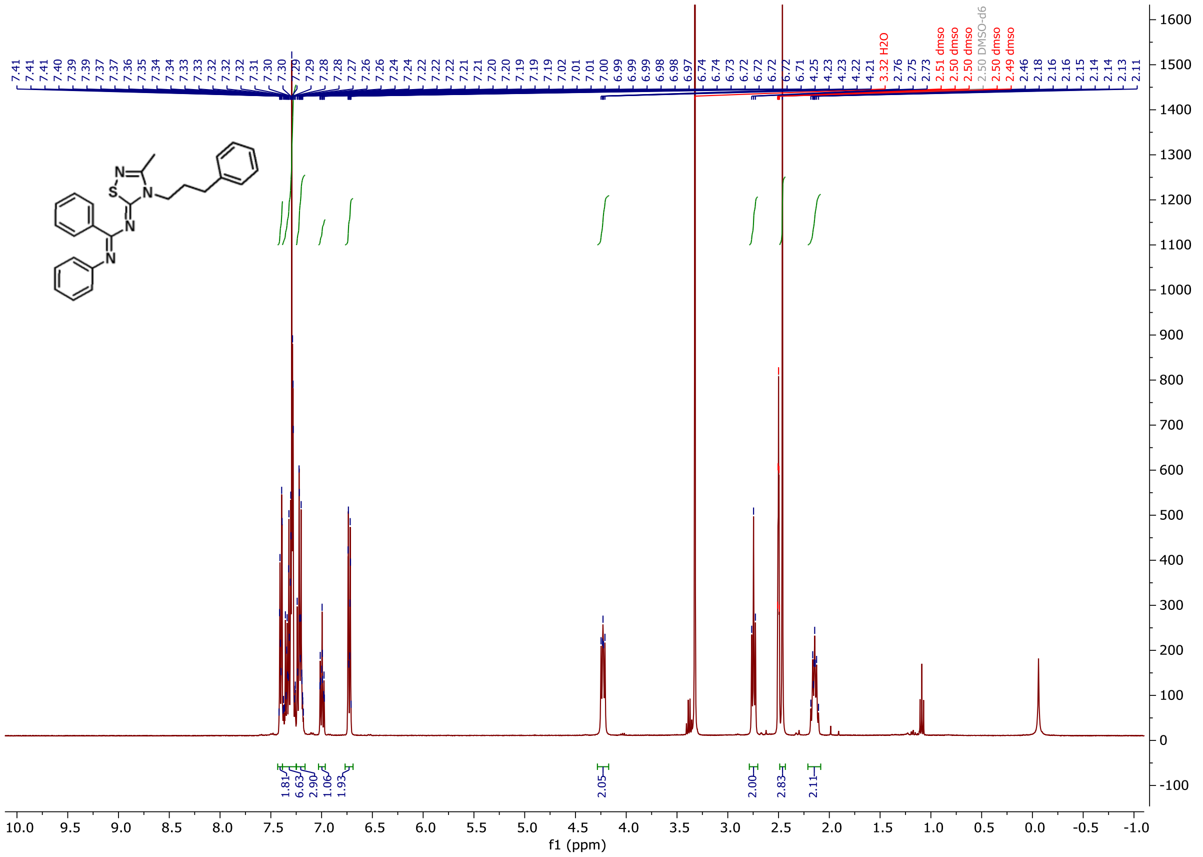

^{13}C-NMR spectrum (101 MHz, DMSO-d_6) of **5**

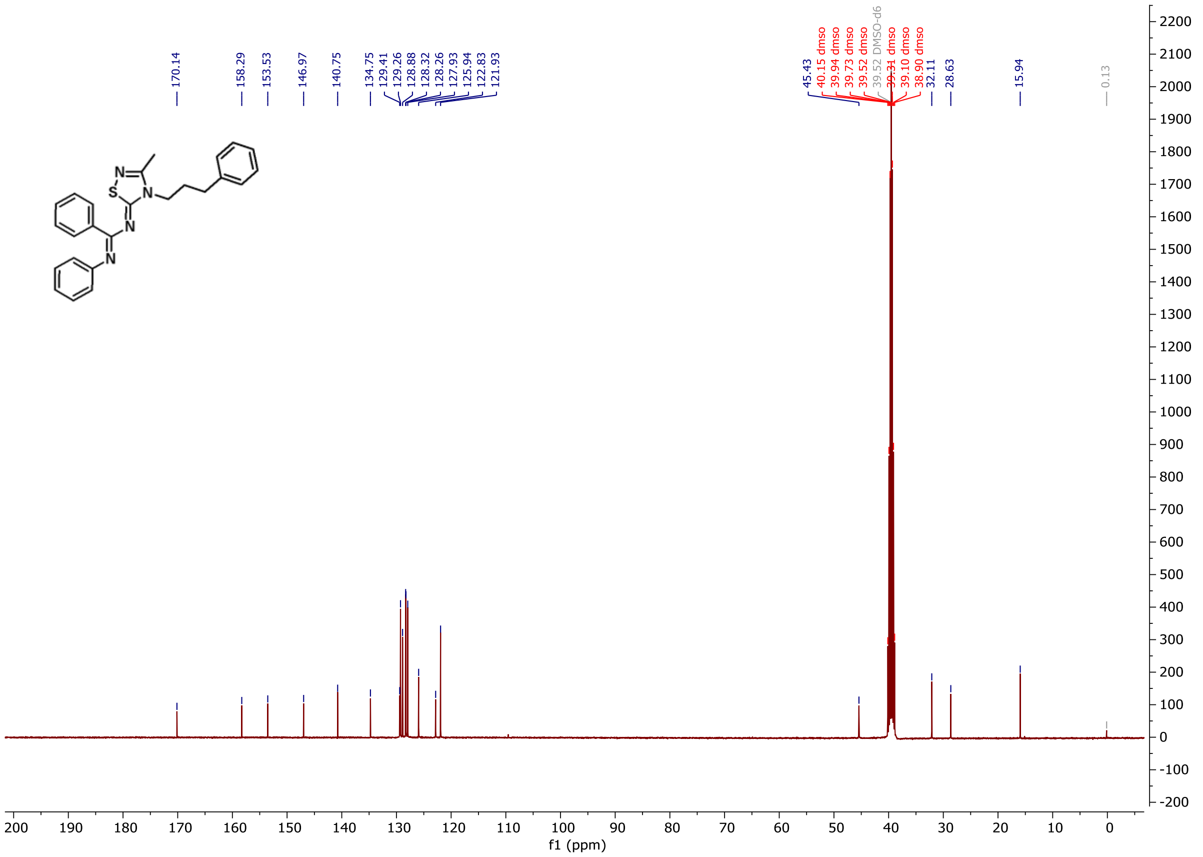

^{1}H-NMR spectrum (400 MHz, DMSO-d_6) of **6**

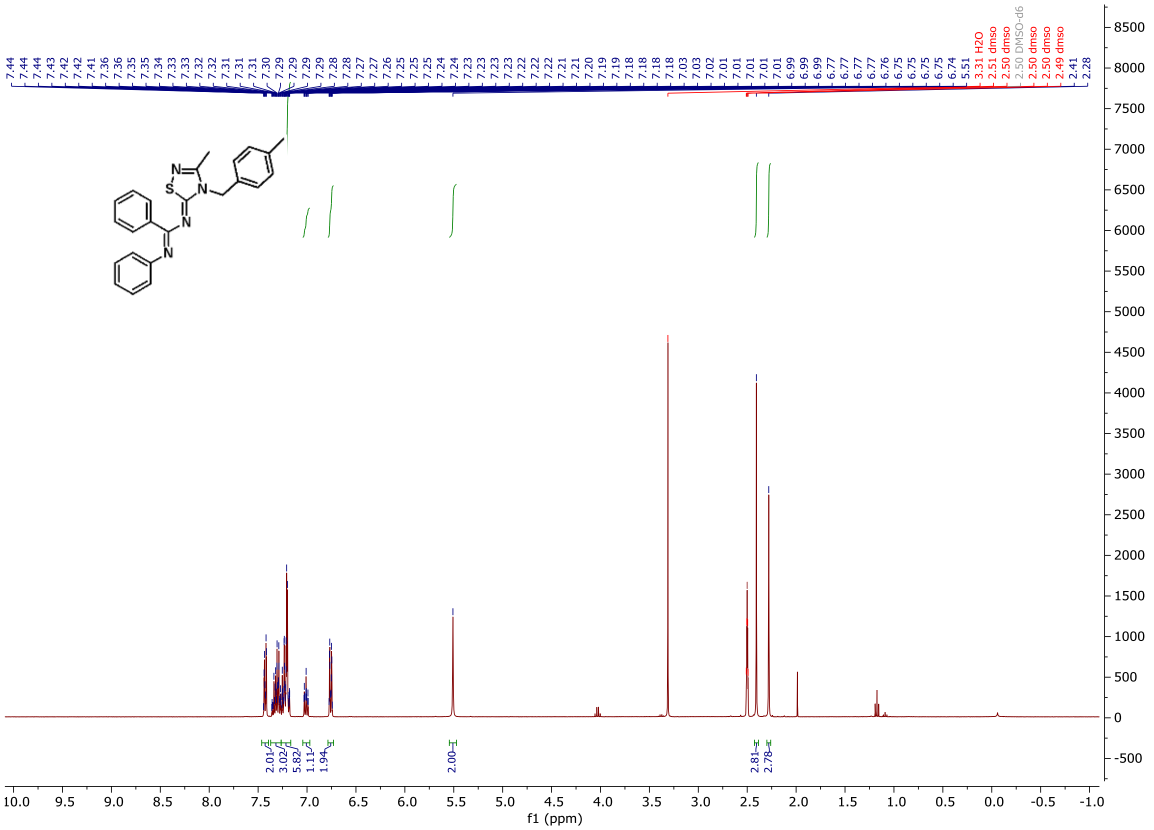

^{13}C-NMR spectrum (101 MHz, DMSO-d_6) of **6**

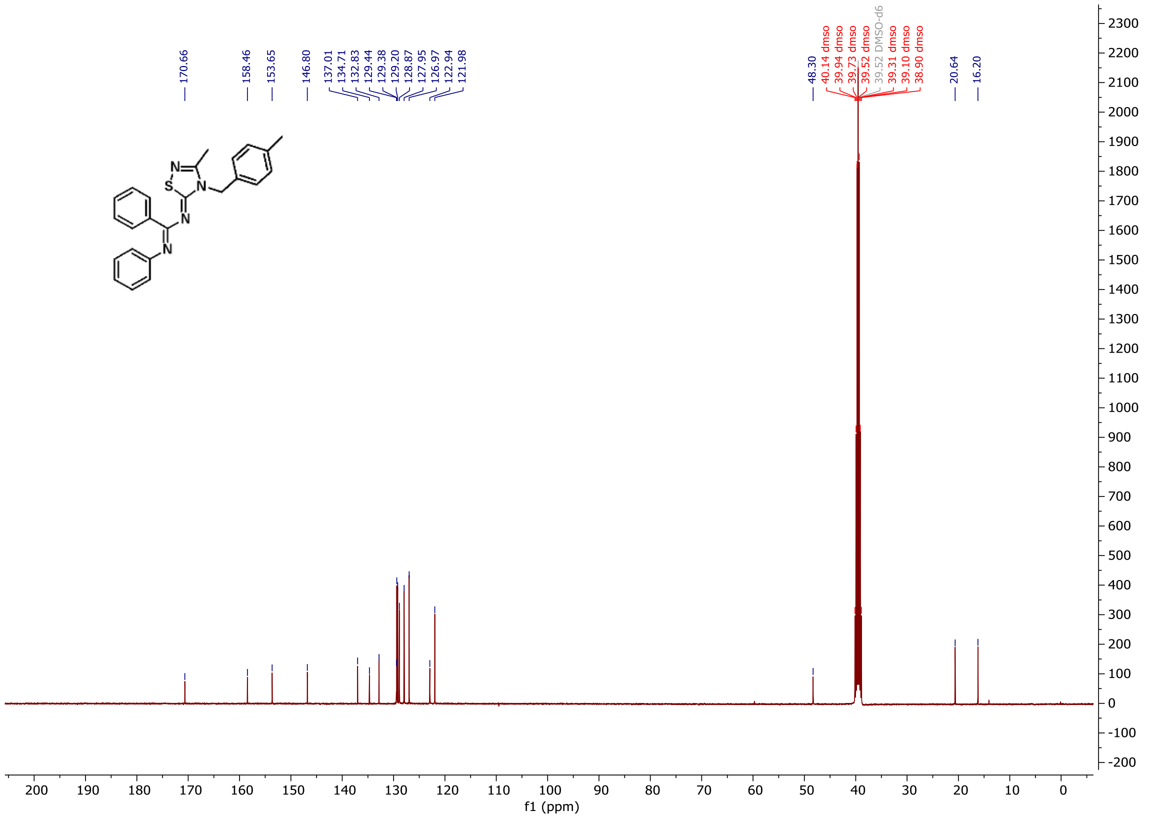

^{1}H-NMR spectrum (400 MHz, DMSO-d_6) of **7**

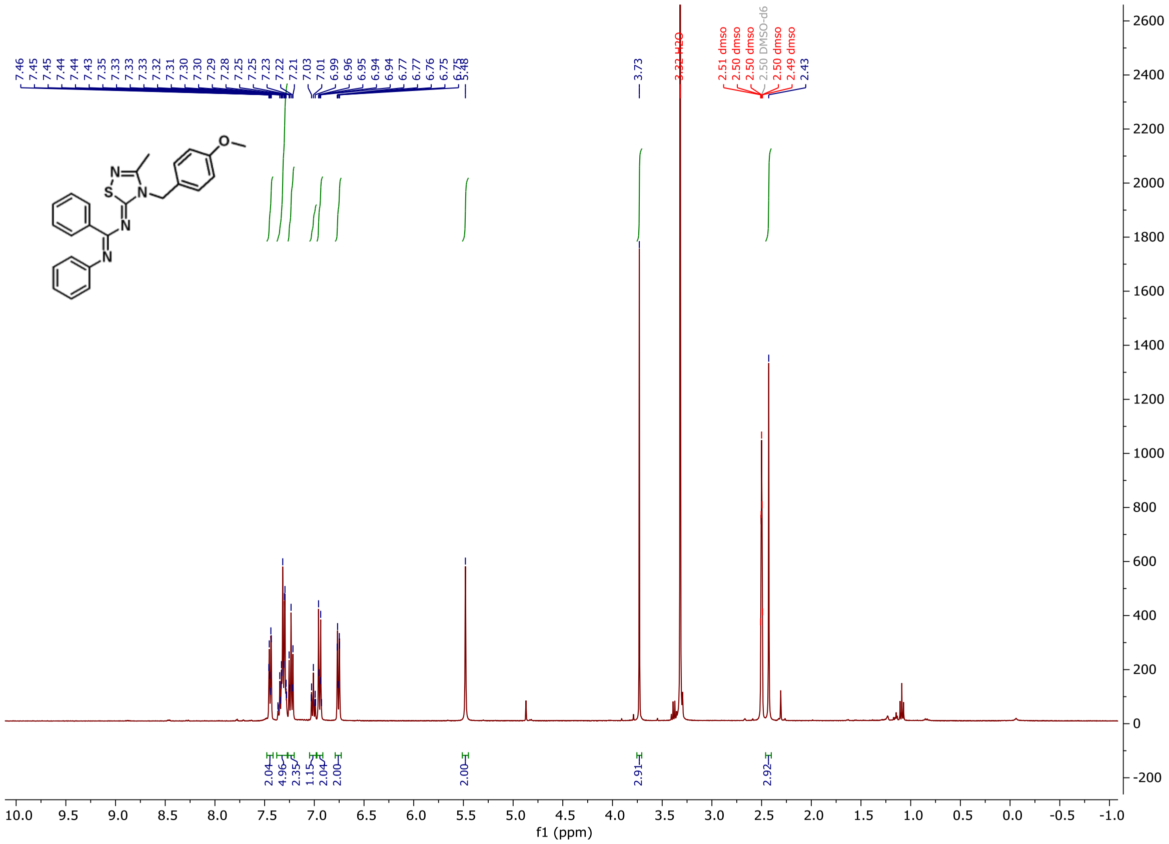

^{13}C-NMR spectrum (101 MHz, DMSO-d_6) of **7**

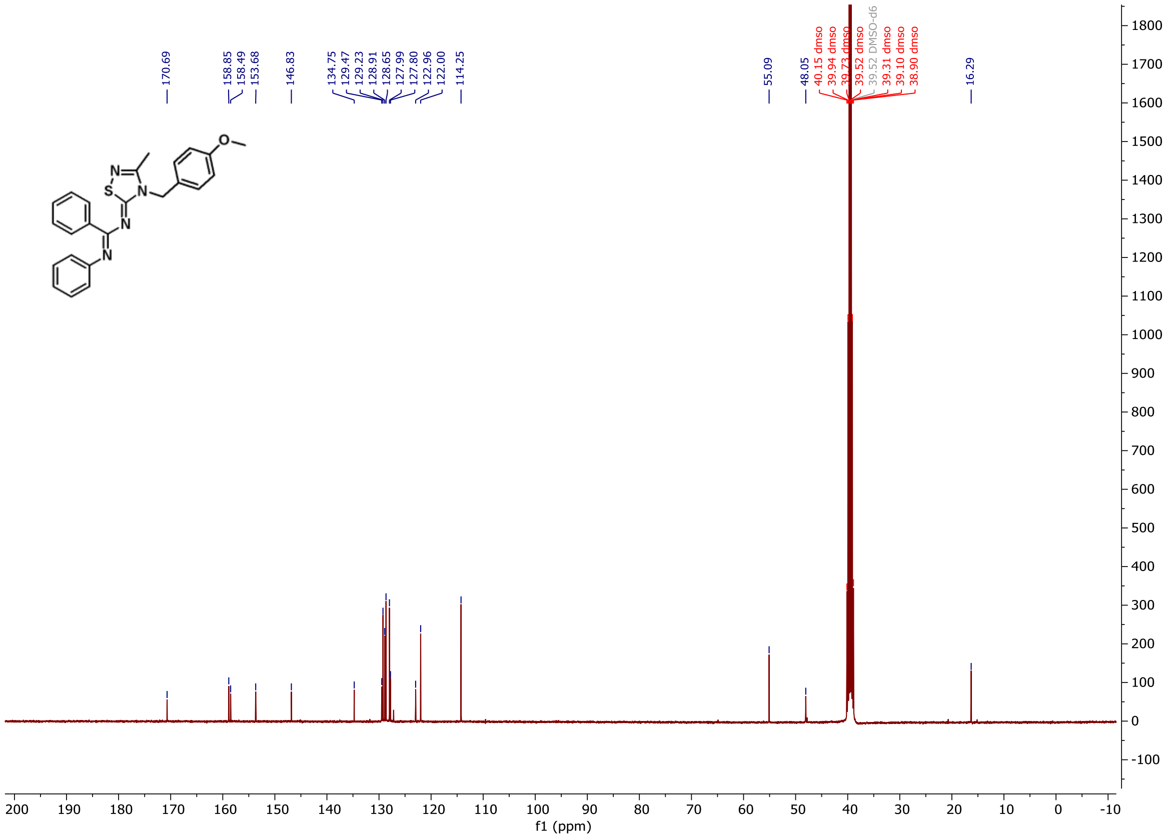

^{1}H-NMR spectrum (400 MHz, DMSO-d_6) of **8**

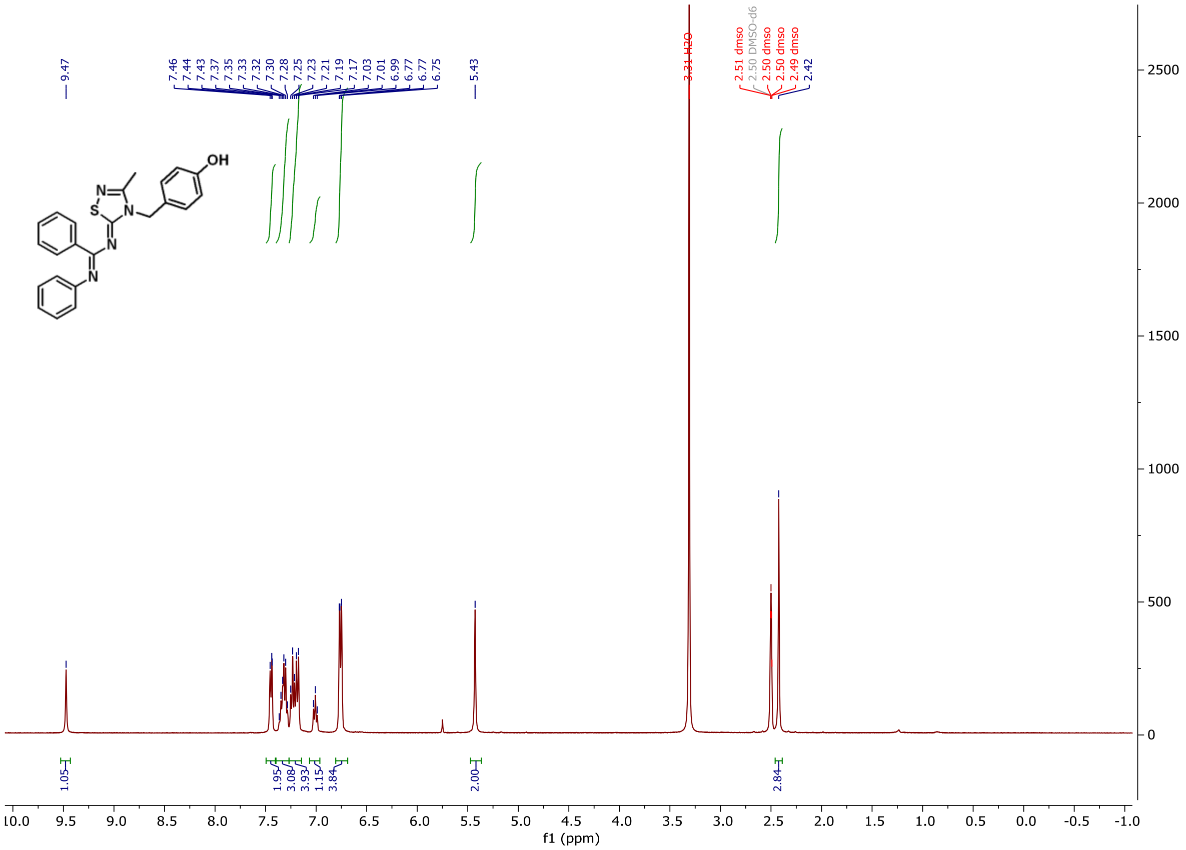

^{13}C-NMR spectrum (101 MHz, DMSO-d_6) of **8**

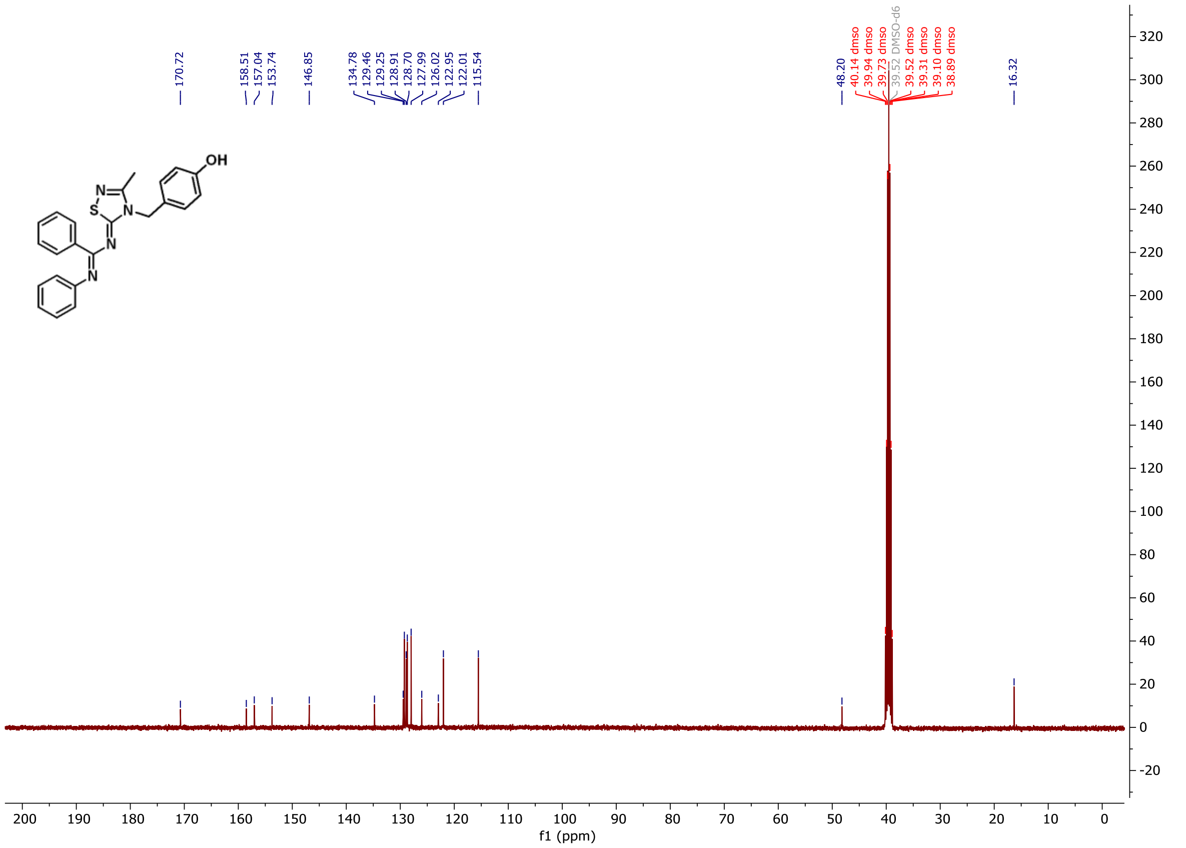

^{1}H-NMR spectrum (400 MHz, DMSO-d_6) of **9**

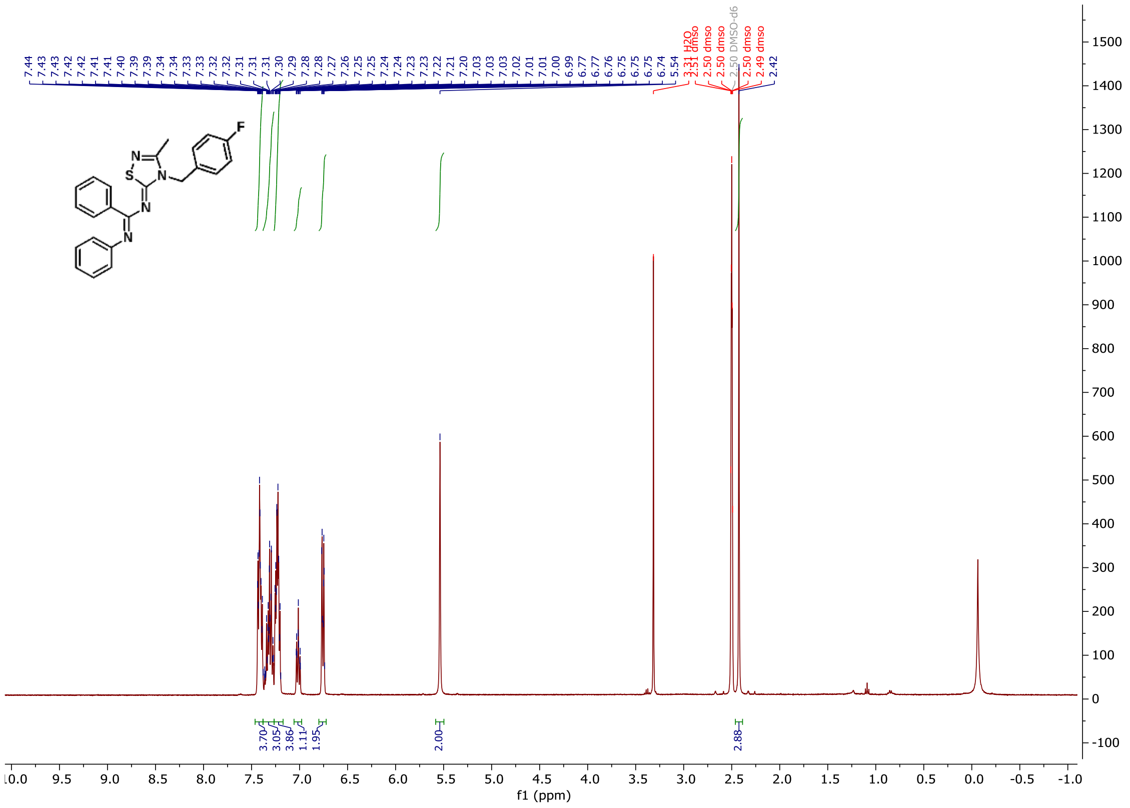

^{13}C-NMR spectrum (101 MHz, DMSO-d_6) of **9**

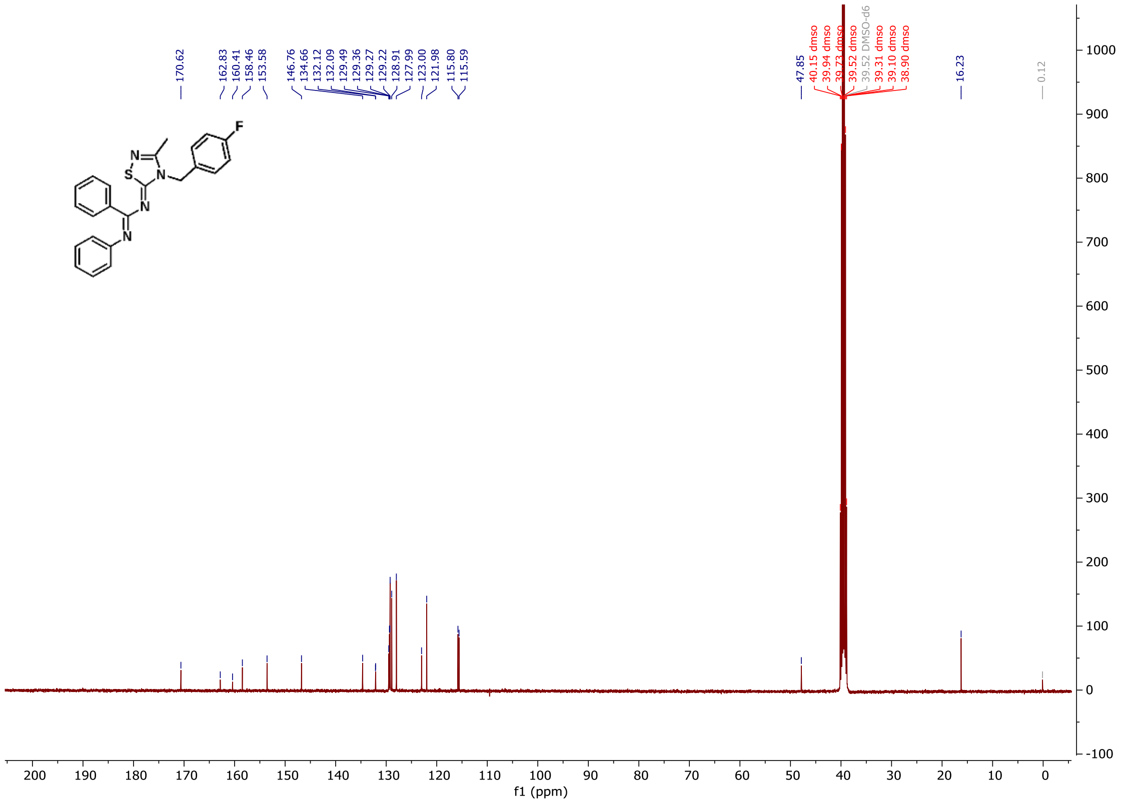

^{1}H-NMR spectrum (400 MHz, DMSO-d_6) of **10**

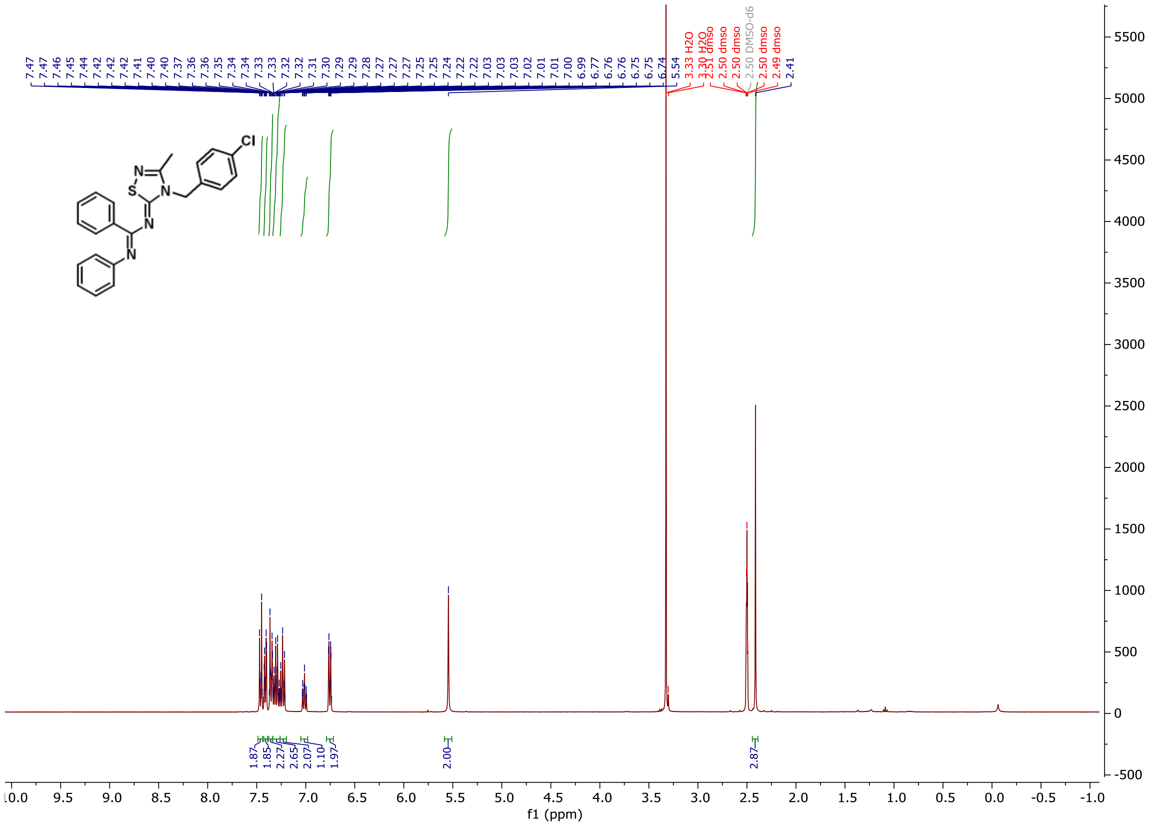

^{13}C-NMR spectrum (101 MHz, DMSO-d_6) of **10**

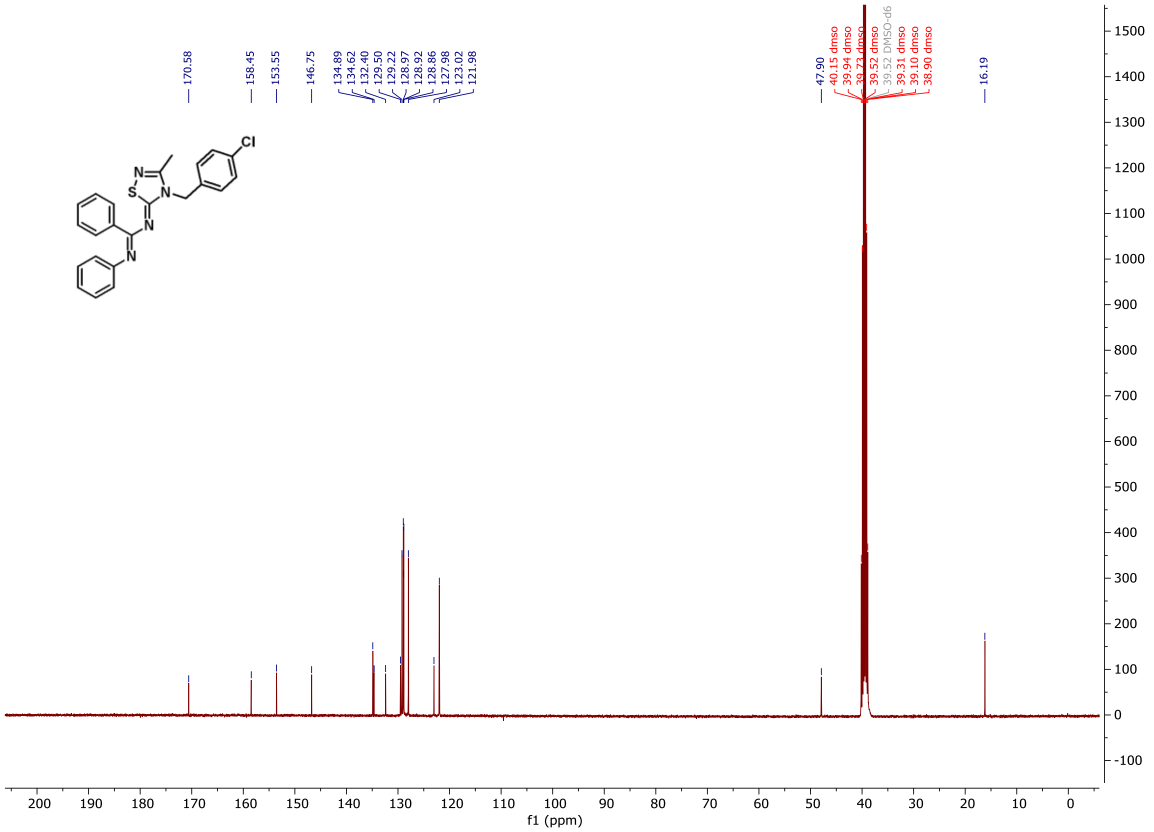

^{1}H-NMR spectrum (400 MHz, DMSO-d_6) of **11**

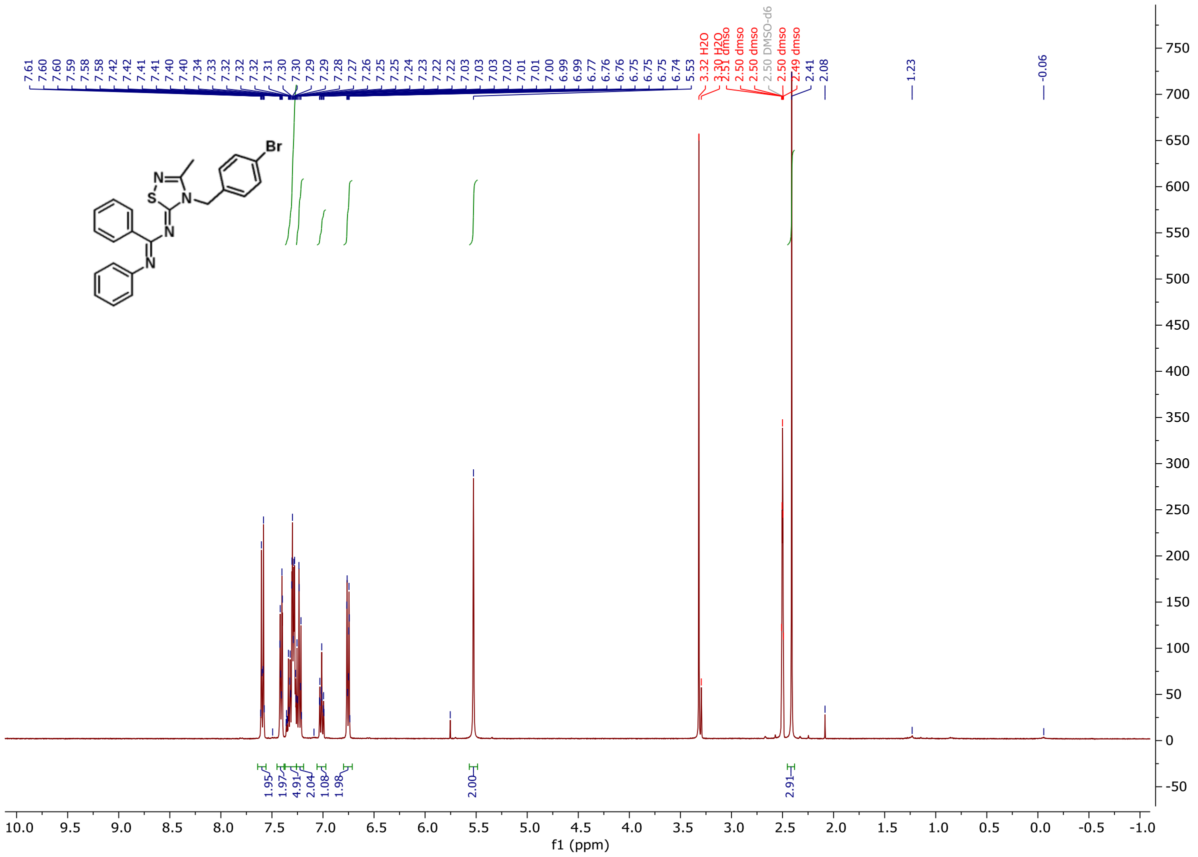

^{13}C-NMR spectrum (101 MHz, DMSO-d_6) of **11**

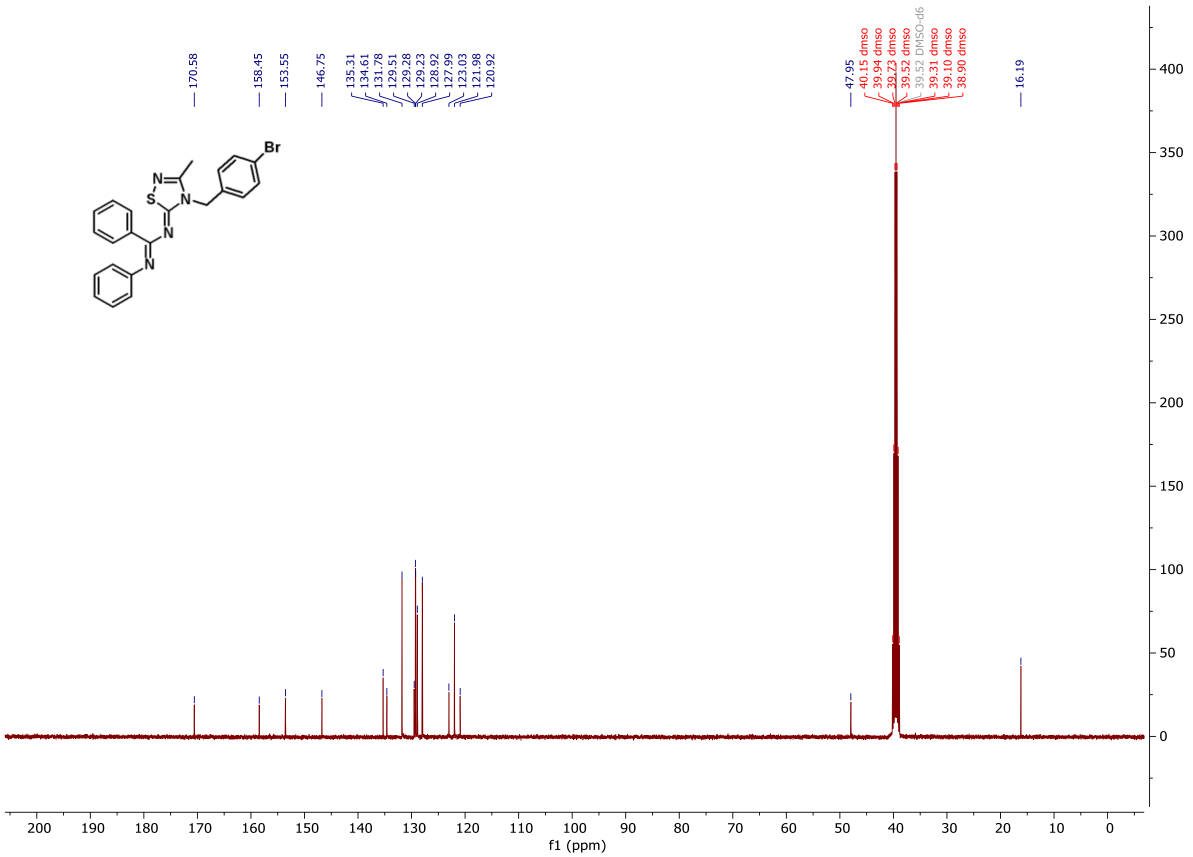

^{1}H-NMR spectrum (400 MHz, DMSO-d_6) of **12**

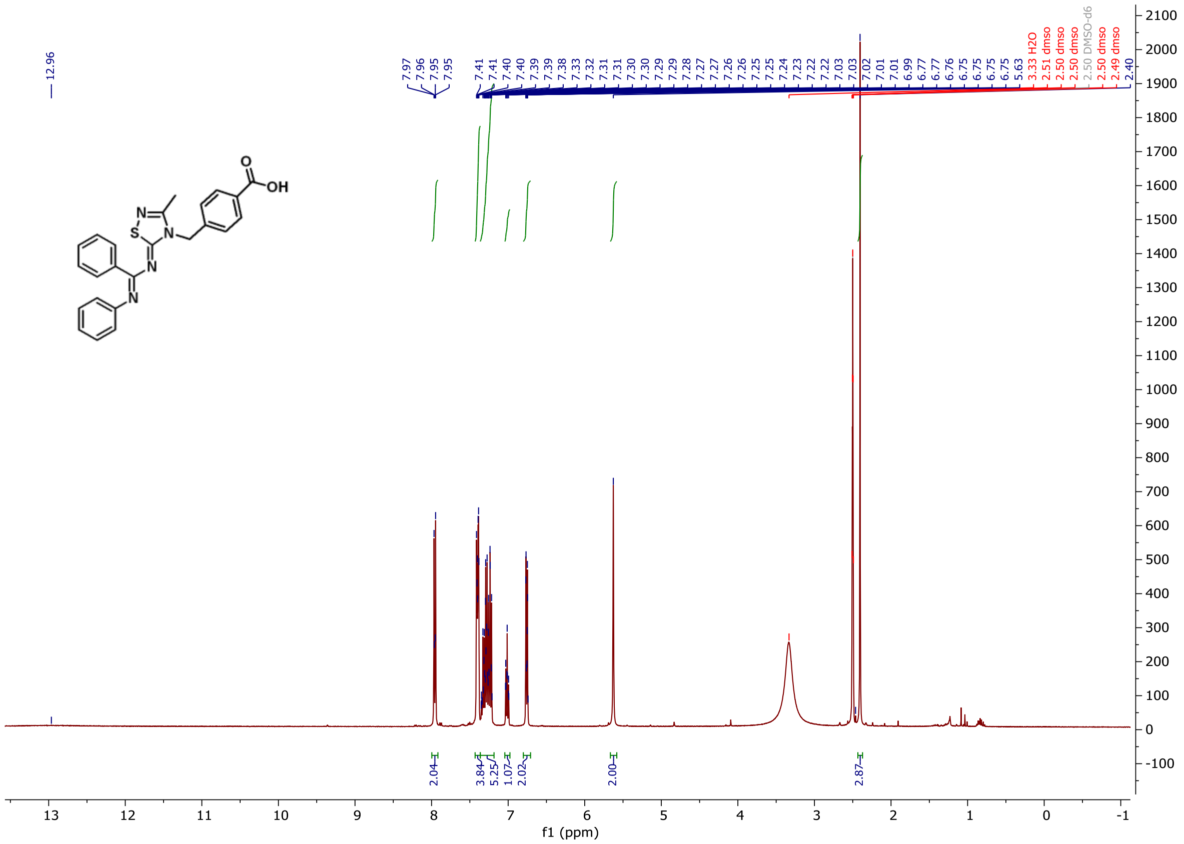

^{13}C-NMR spectrum (101 MHz, DMSO-d_6) of **12**

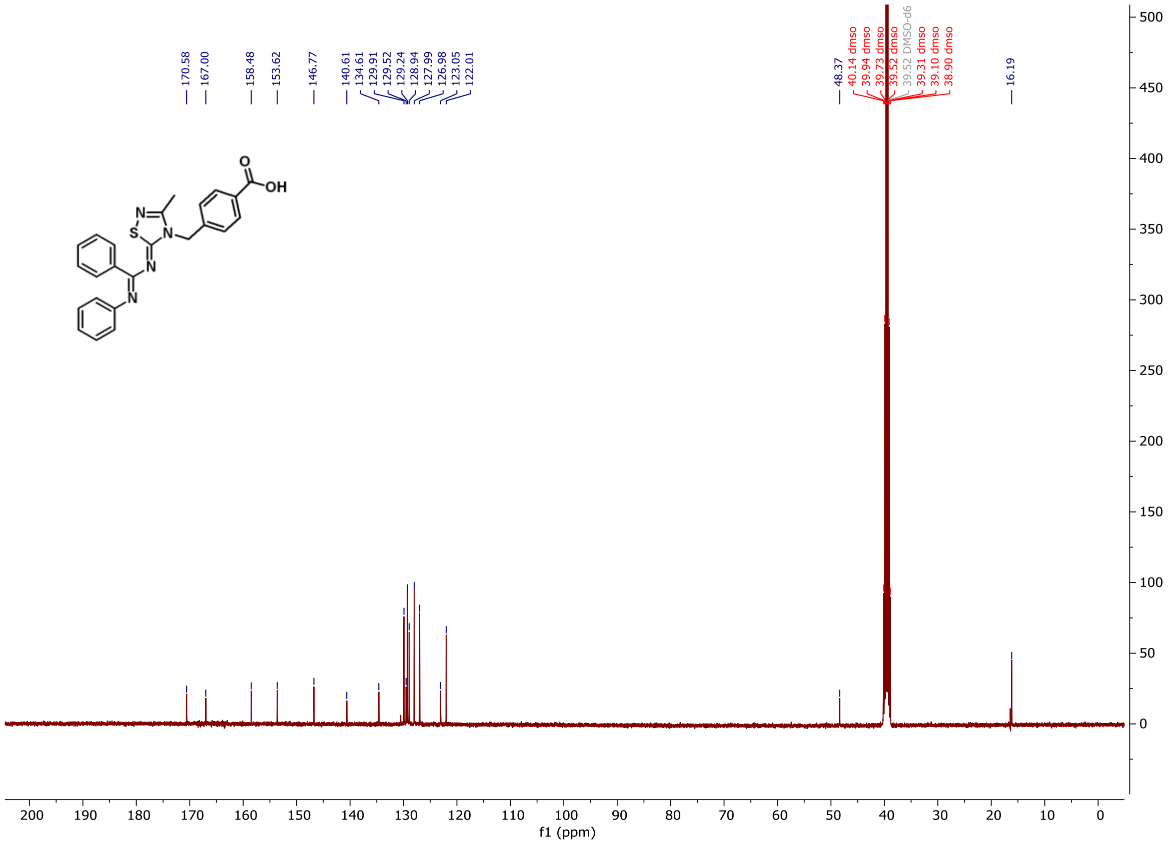

^{1}H-NMR spectrum (400 MHz, DMSO-d_6) of **13**

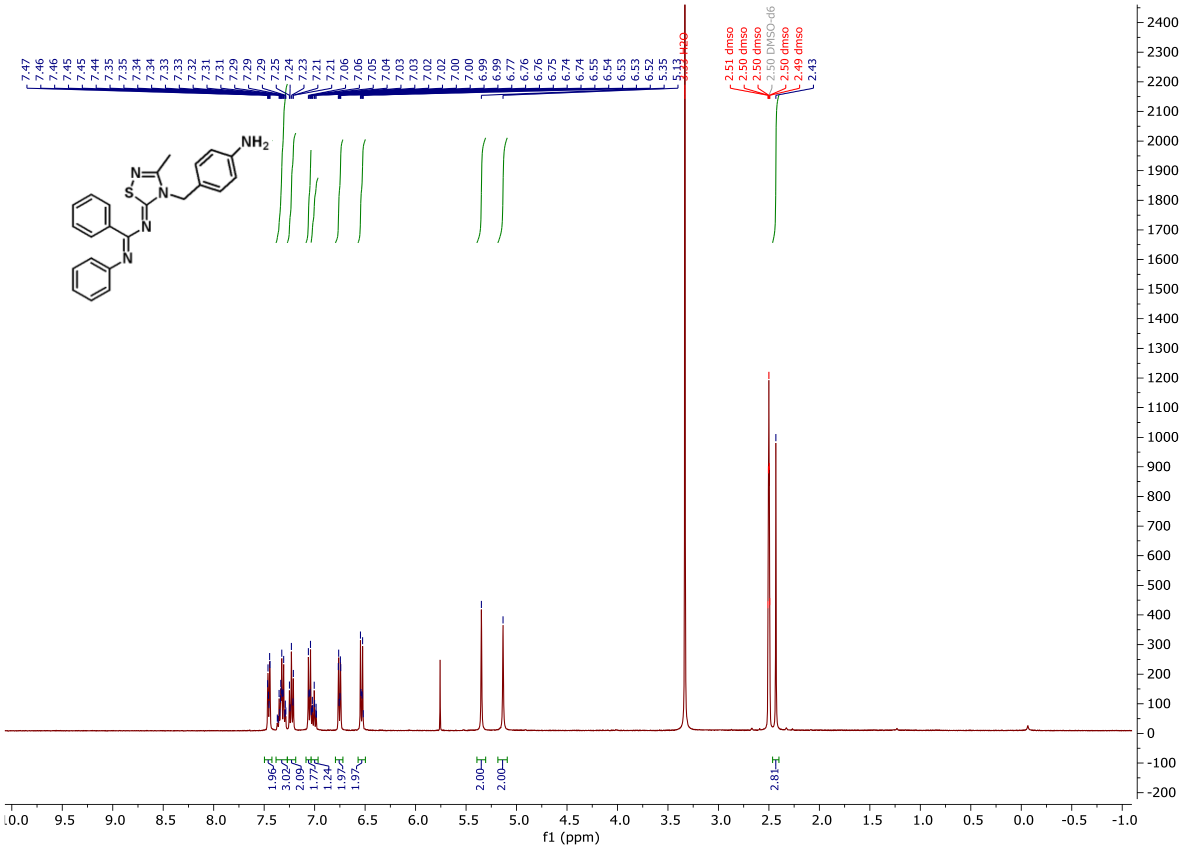

^{13}C-NMR spectrum (101 MHz, DMSO-d_6) of **13**

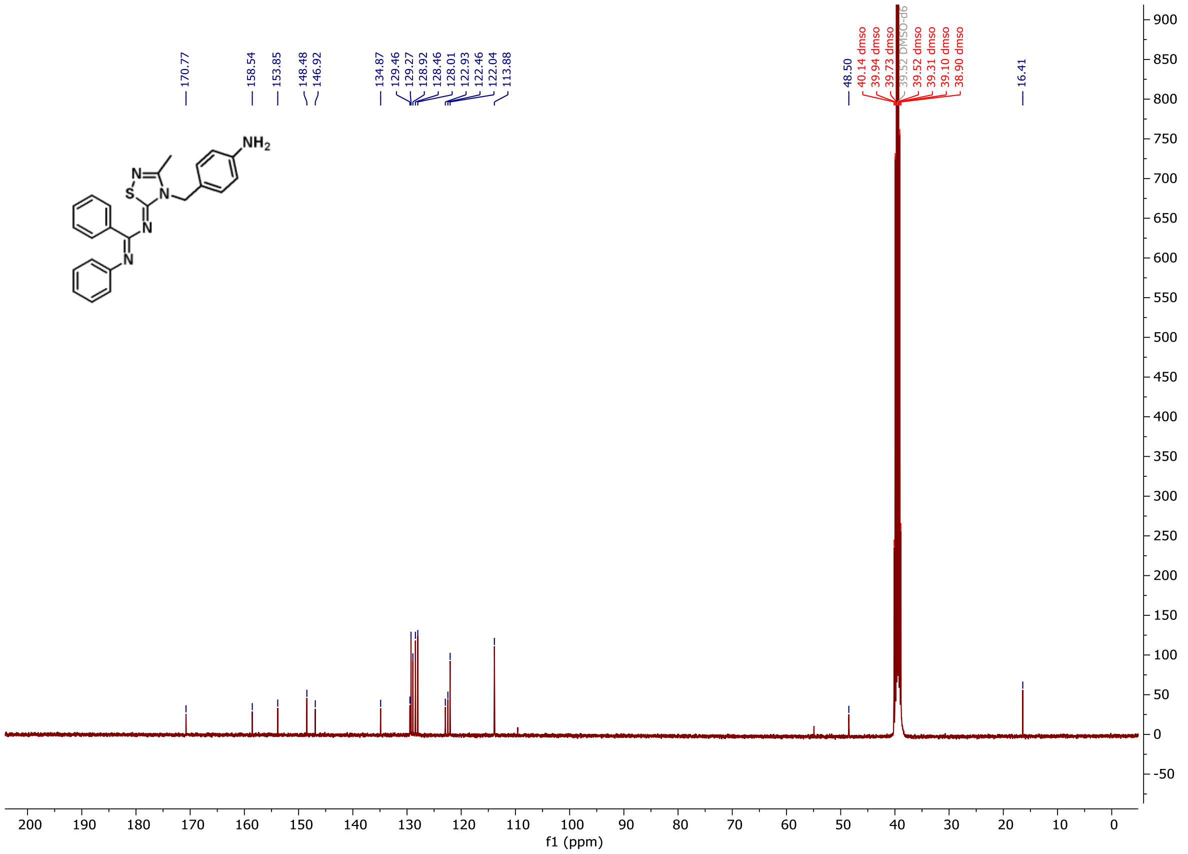

^{1}H-NMR spectrum (400 MHz, DMSO-d_6) of **14**

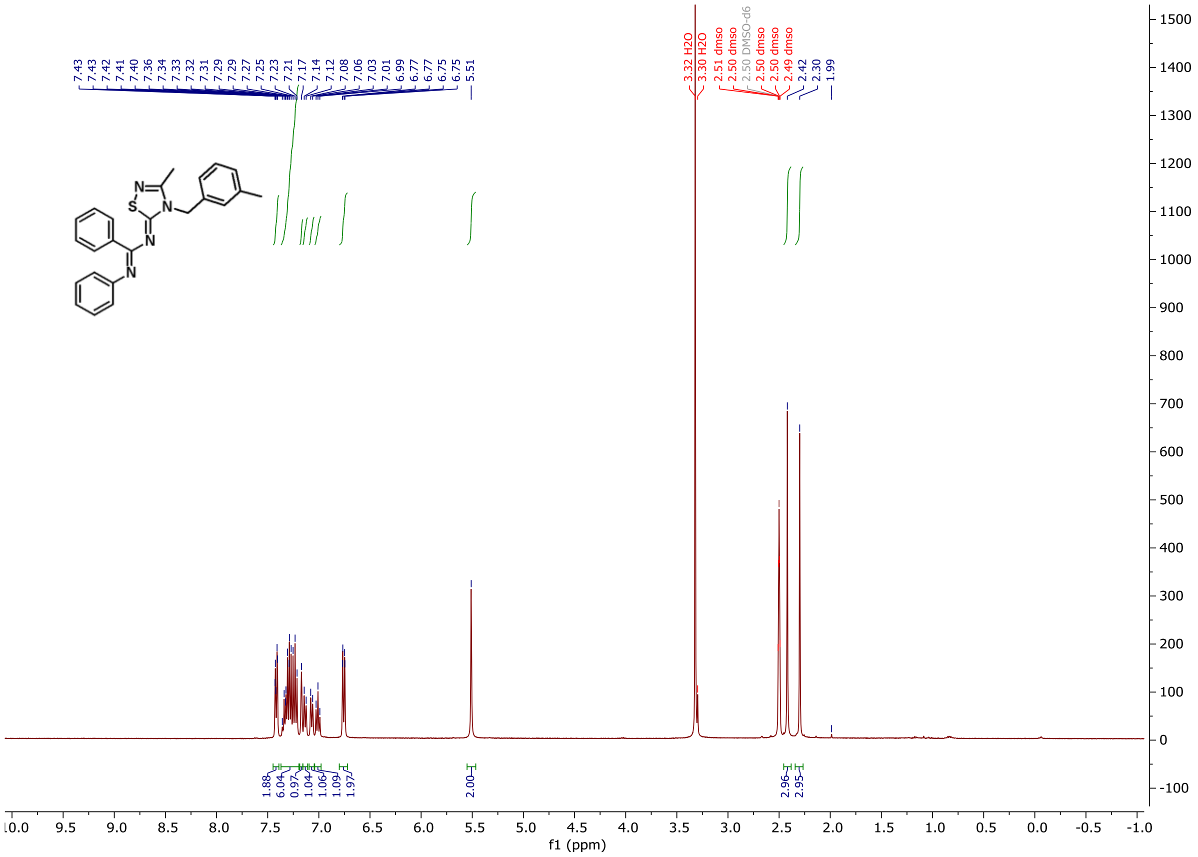

^{13}C-NMR spectrum (101 MHz, DMSO-d_6) of **14**

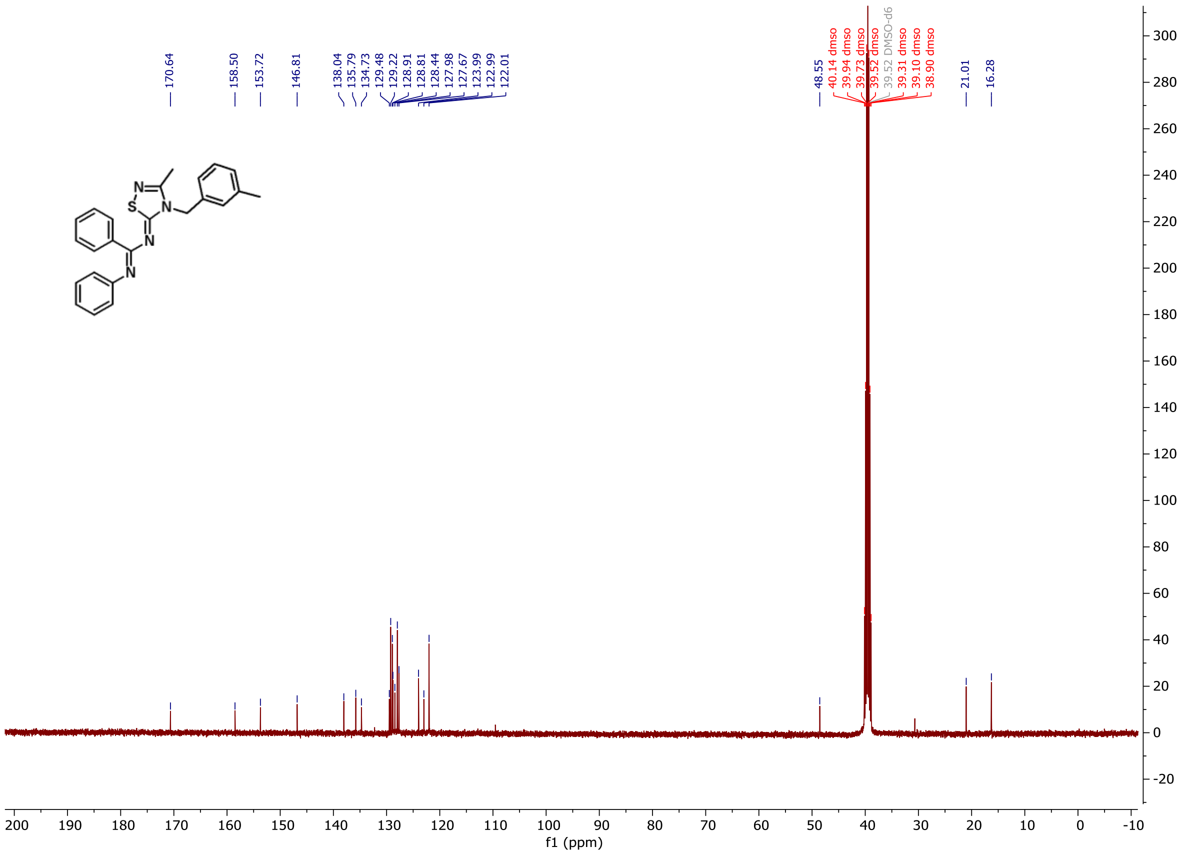

^{1}H-NMR spectrum (400 MHz, DMSO-d_6) of **15**

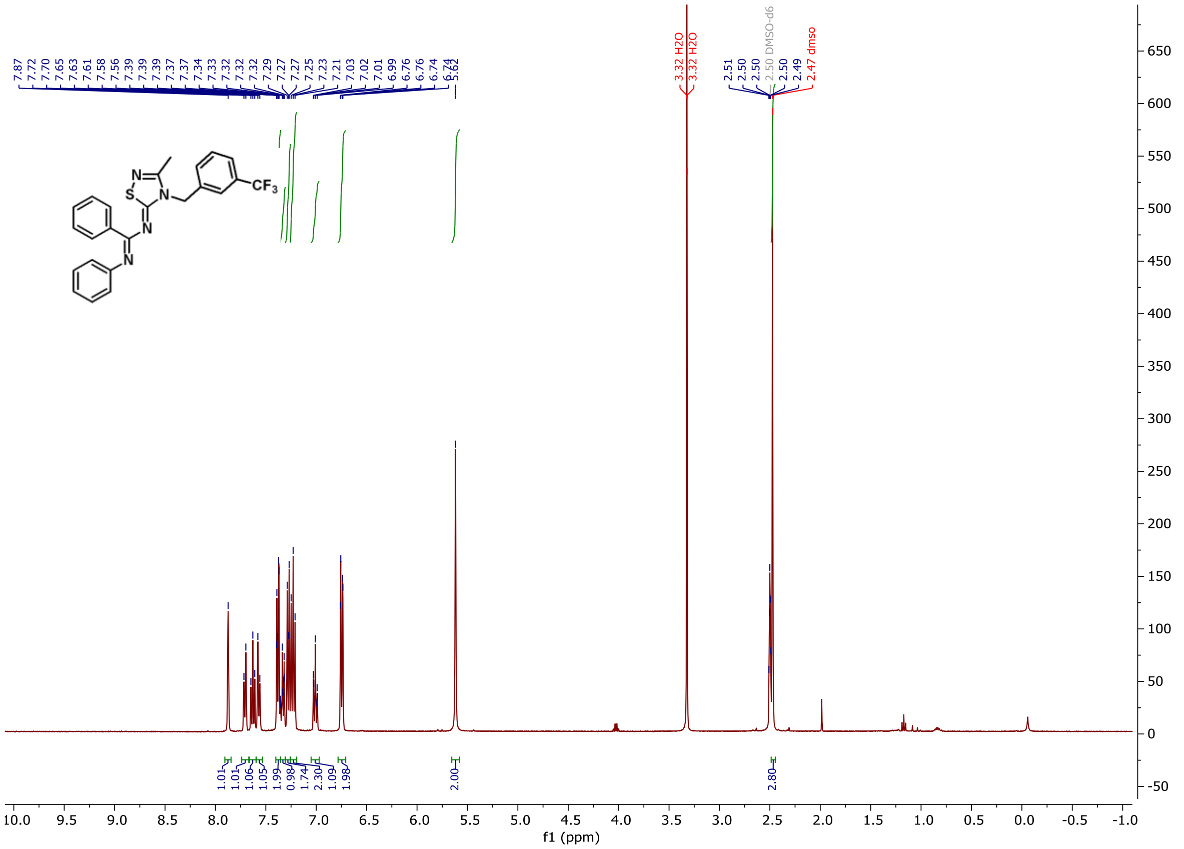

^{13}C-NMR spectrum (101 MHz, DMSO-d_6) of **15**

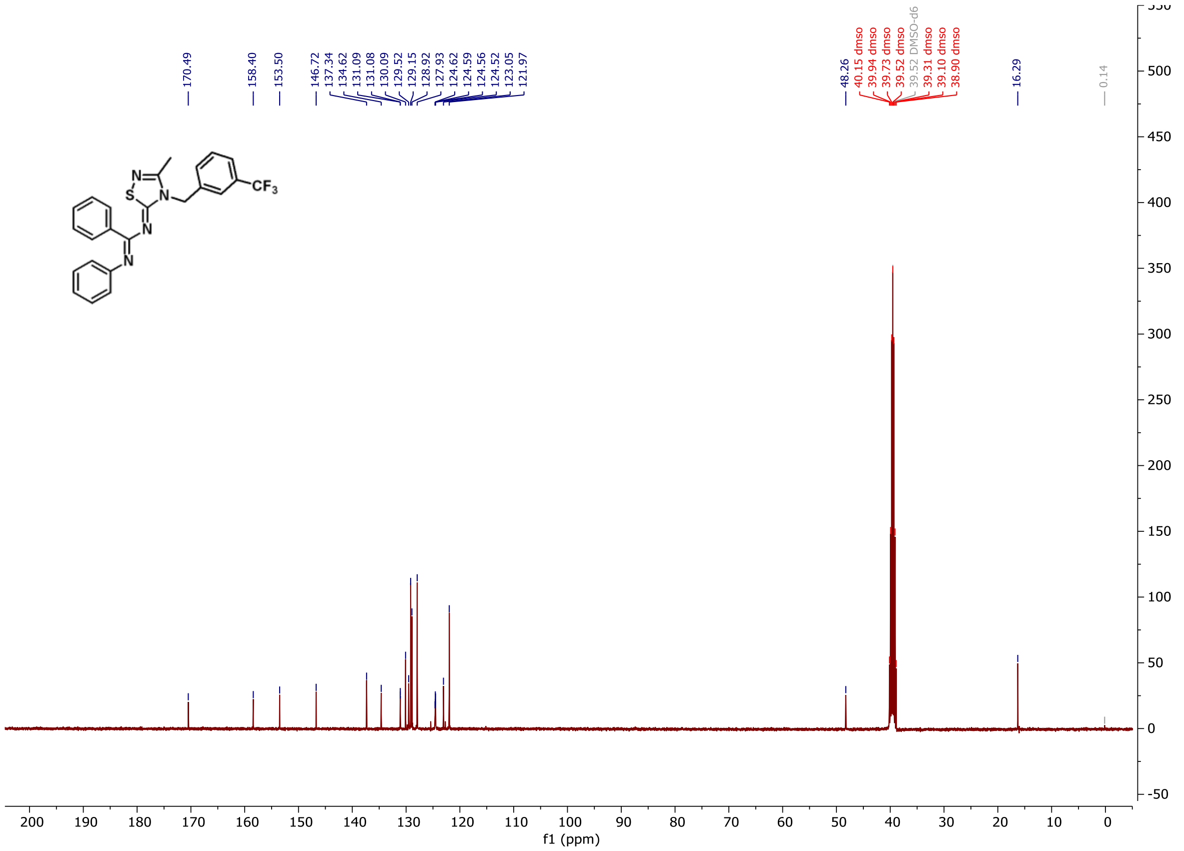

^{1}H-NMR spectrum (400 MHz, DMSO-d_6) of **16**

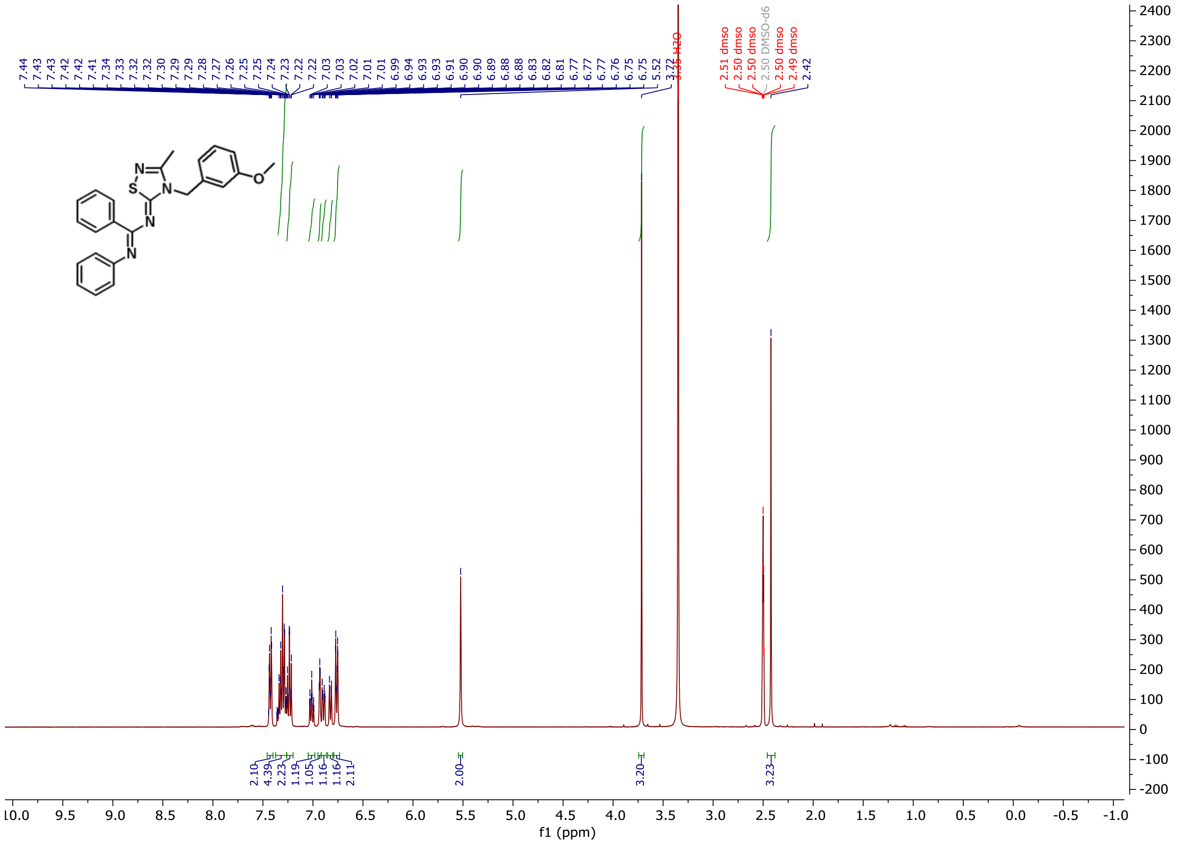

^{13}C-NMR spectrum (101 MHz, DMSO-d_6) of **16**

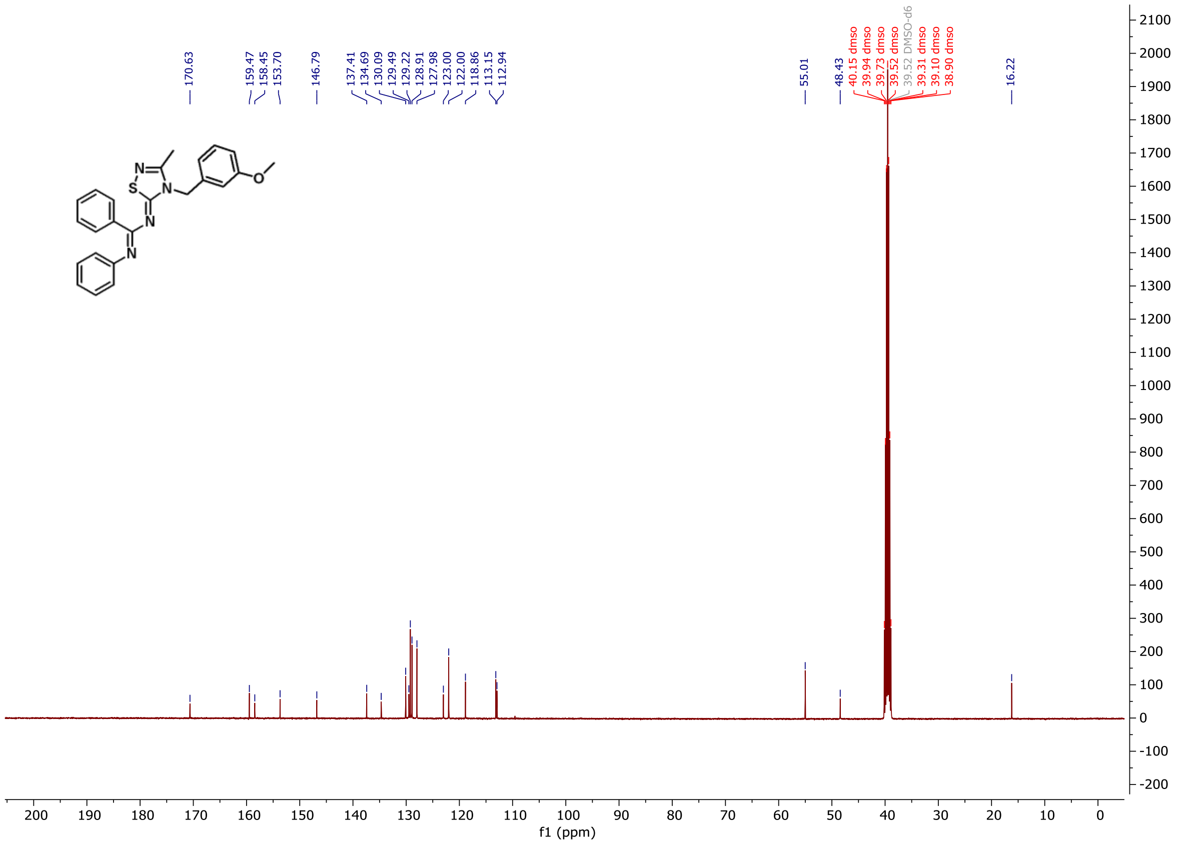

^{1}H-NMR spectrum (400 MHz, DMSO-d_6) of **17**

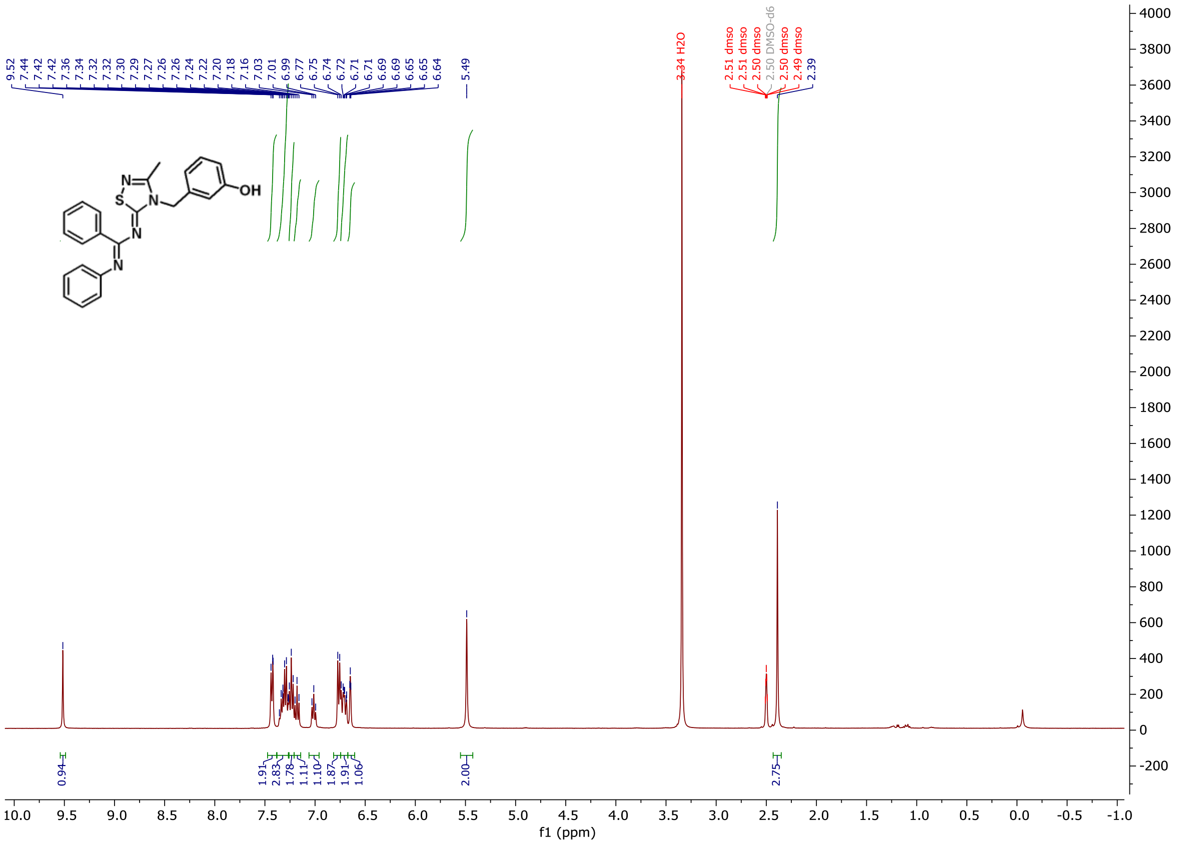

^{13}C-NMR spectrum (101 MHz, DMSO-d_6) of **17**

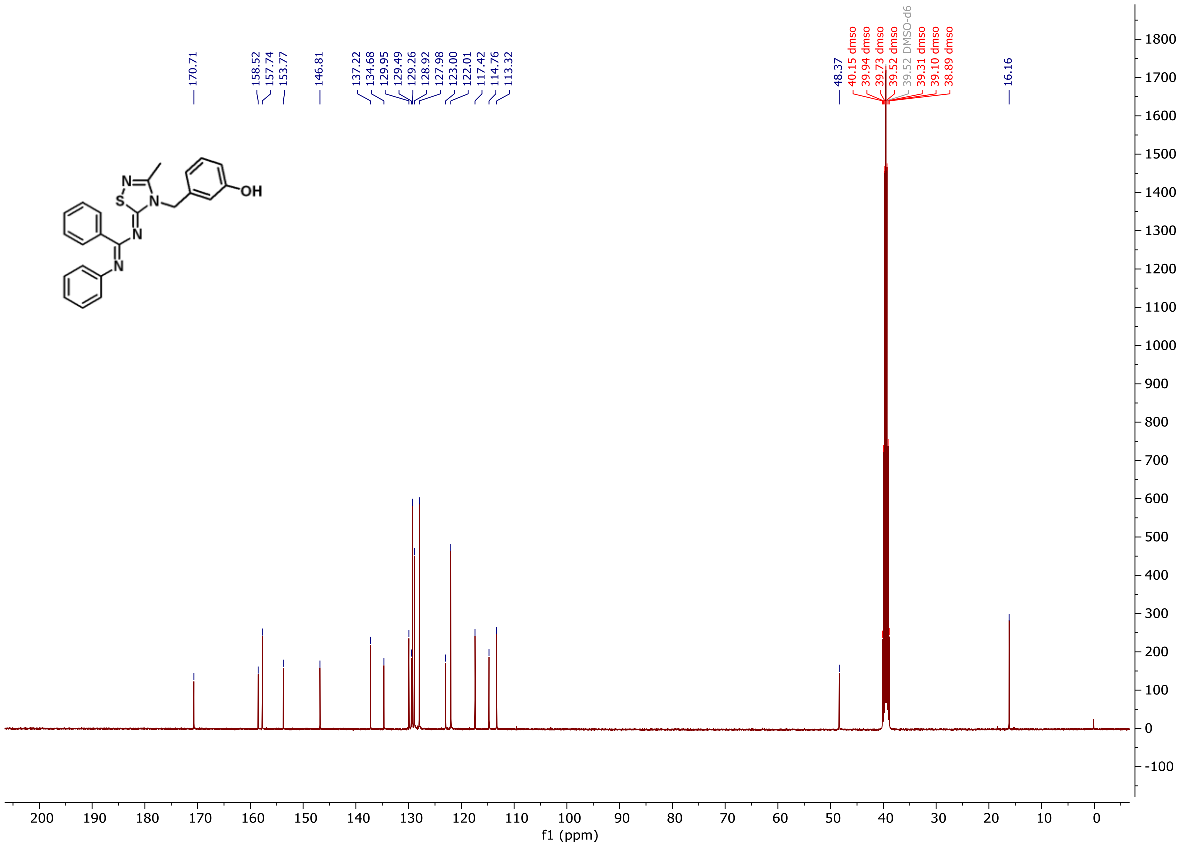

^{1}H-NMR spectrum (400 MHz, DMSO-d_6) of **18**

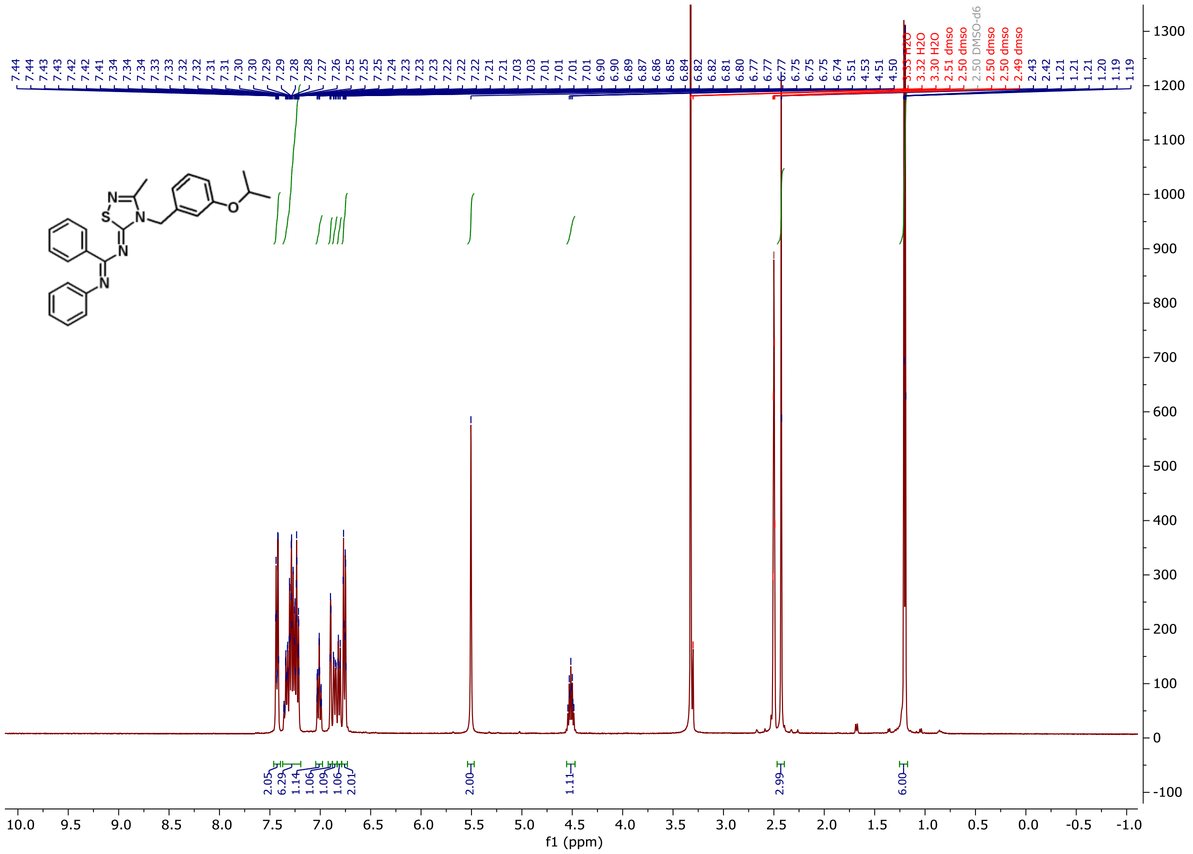

^{13}C-NMR spectrum (101 MHz, DMSO-d_6) of **18**

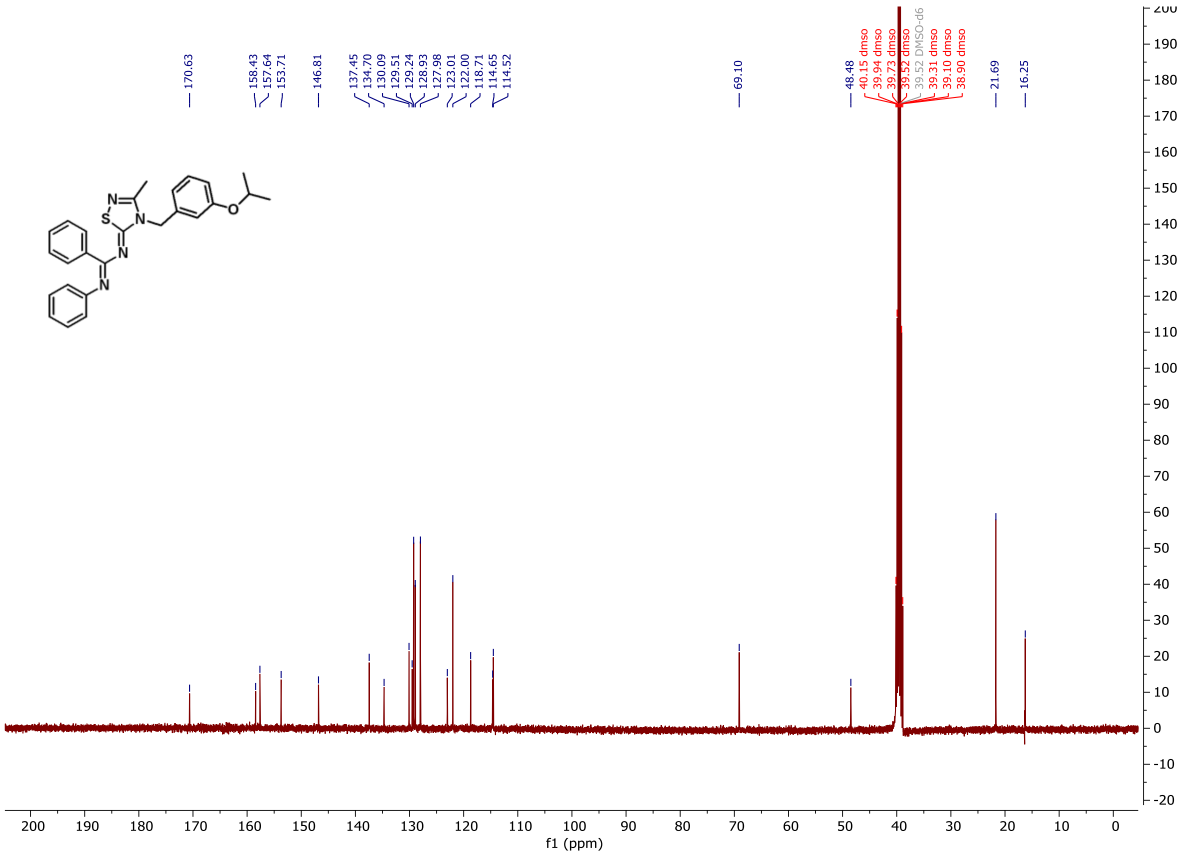

^{1}H-NMR spectrum (400 MHz, DMSO-d_6) of **19**

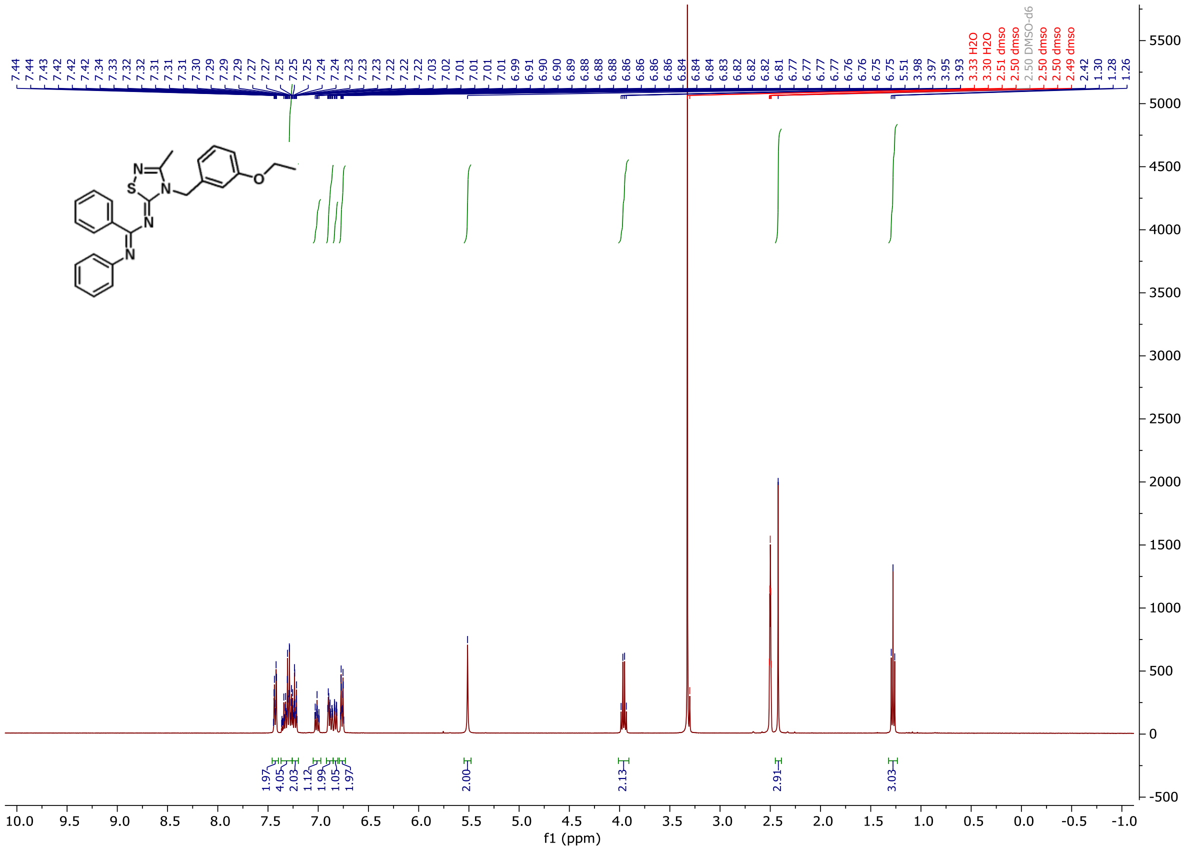

^{13}C-NMR spectrum (101 MHz, DMSO-d_6) of **19**

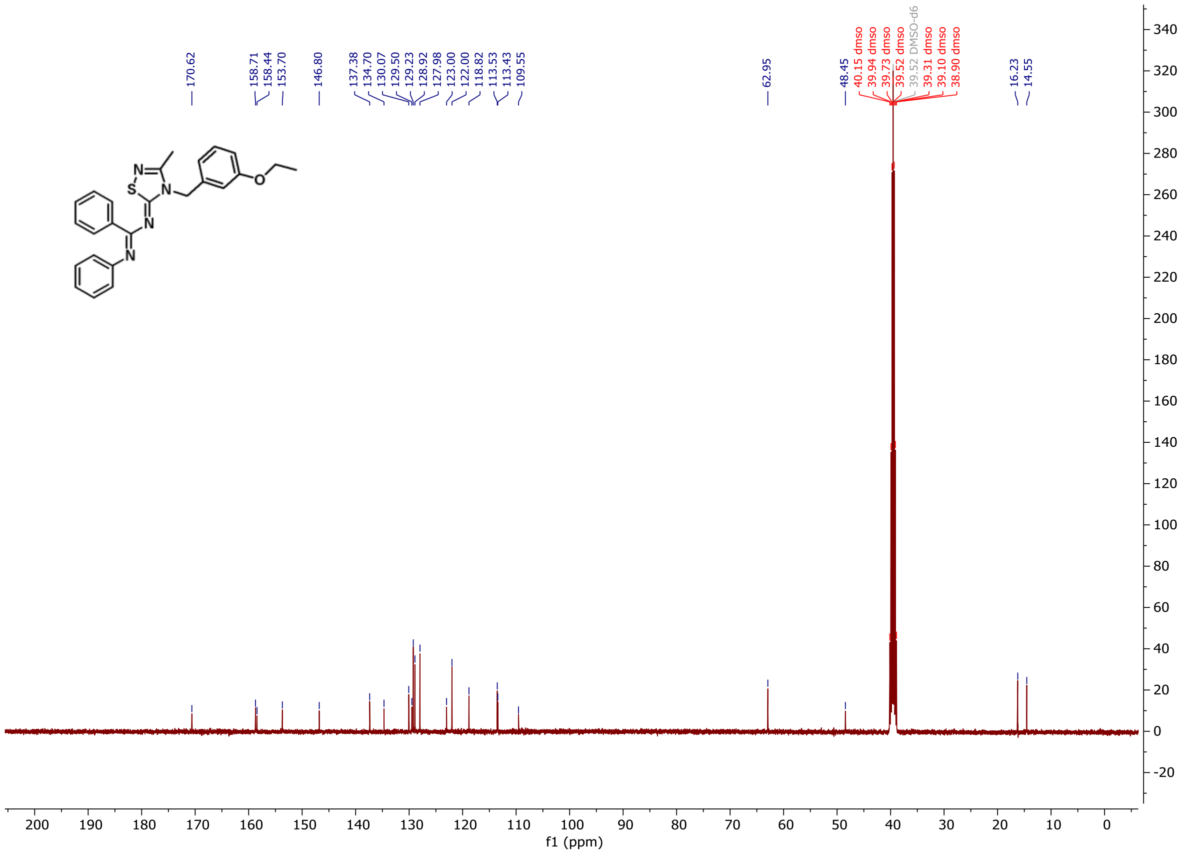

^{1}H-NMR spectrum (400 MHz, DMSO-d_6) of **20**

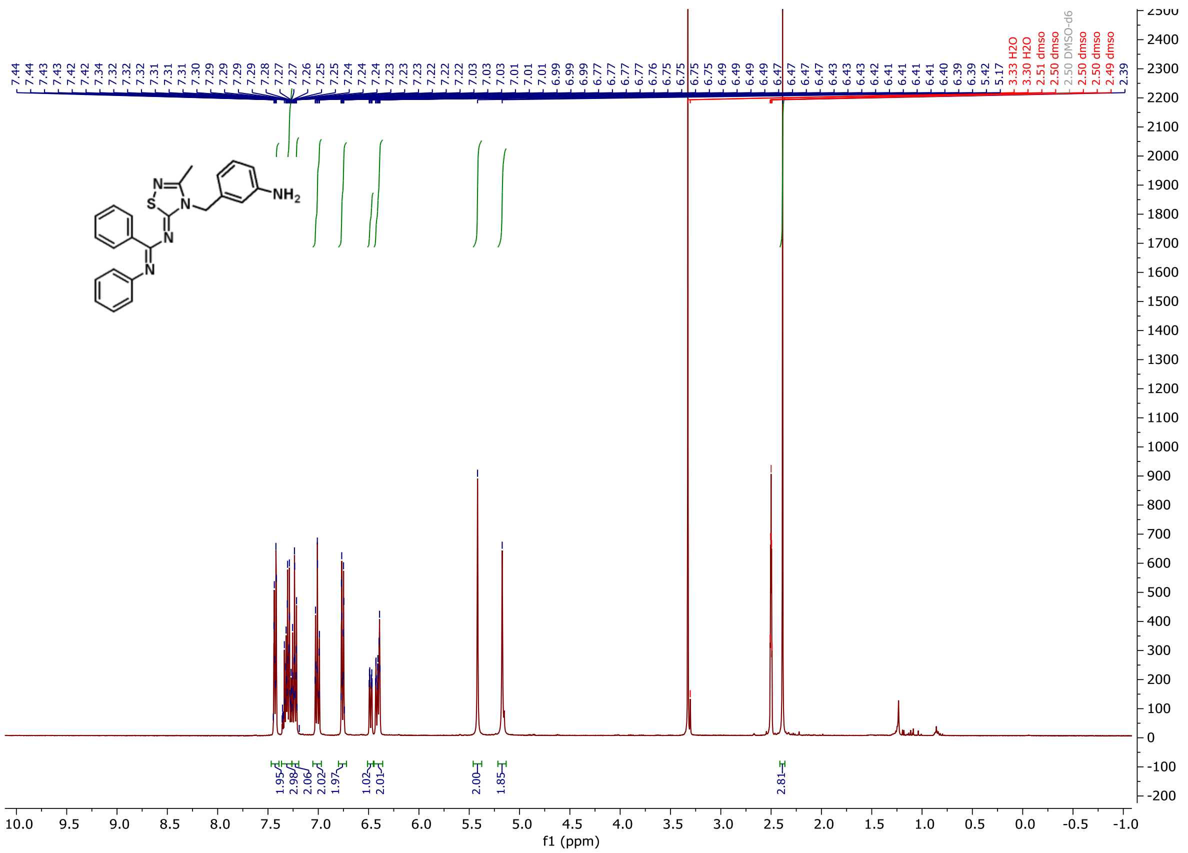

^{13}C-NMR spectrum (101 MHz, DMSO-d_6) of **20**

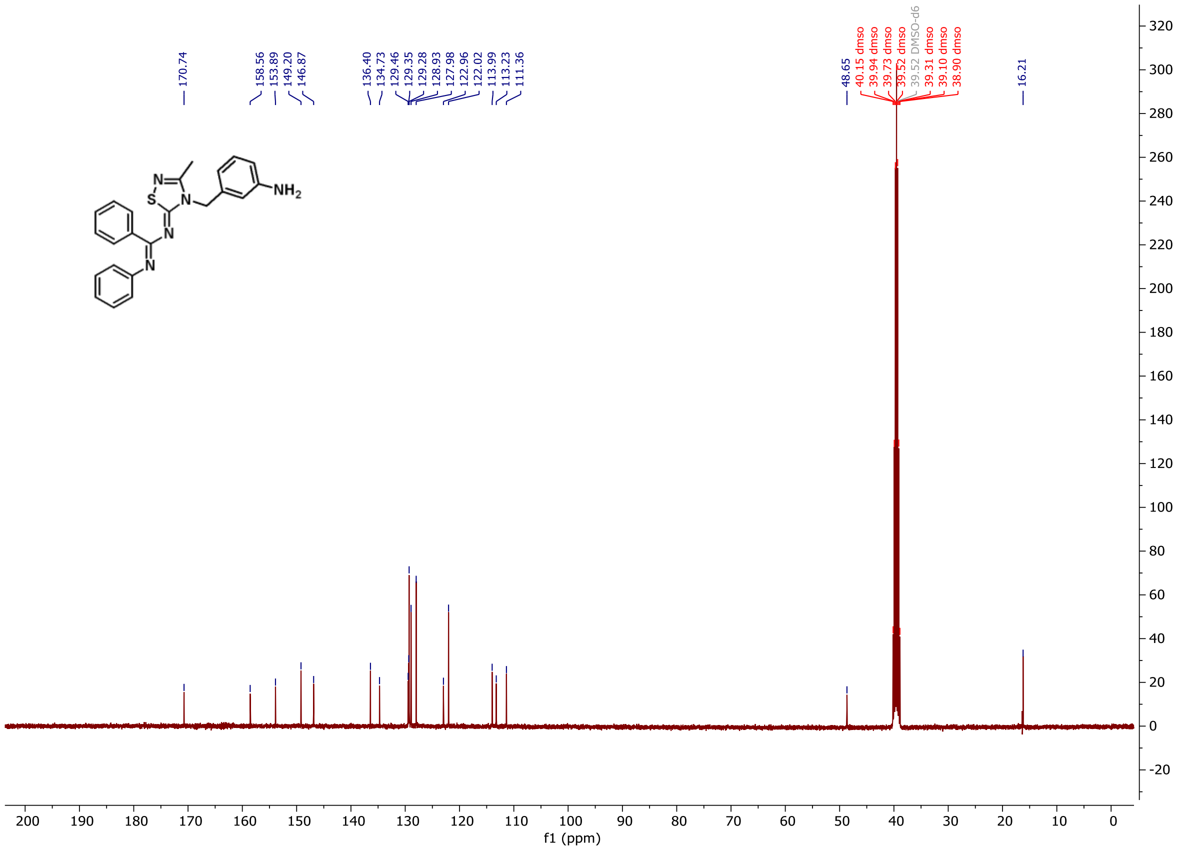

^{1}H-NMR spectrum (400 MHz, DMSO-d_6) of **21**

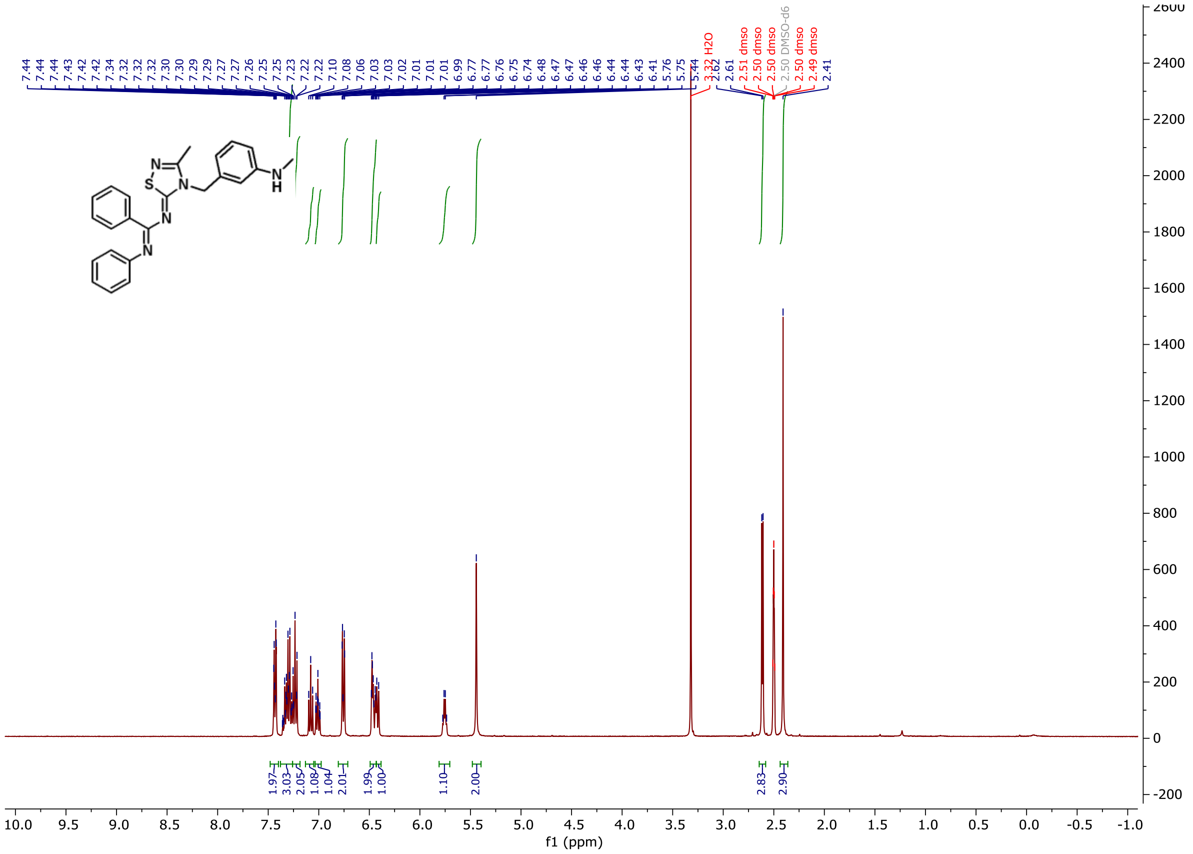

^{13}C-NMR spectrum (101 MHz, DMSO-d_6) of **21**

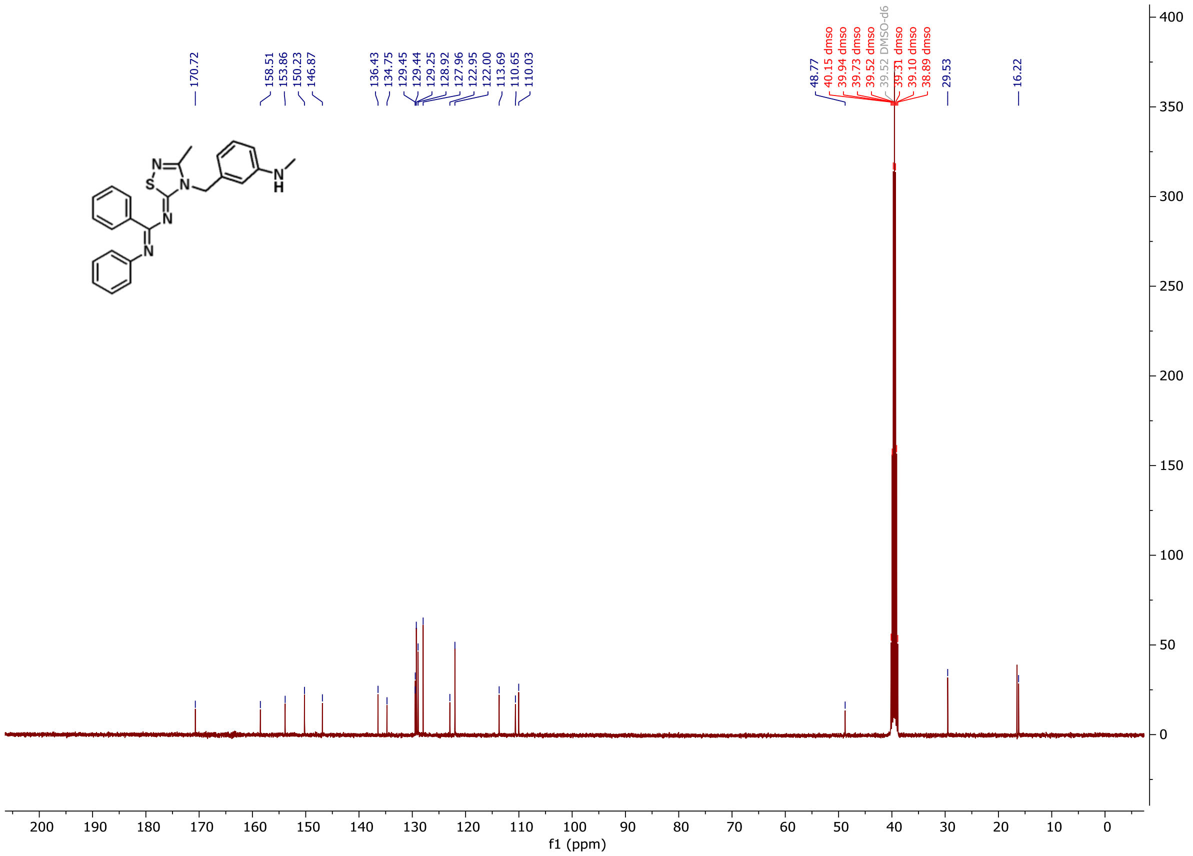

^{1}H-NMR spectrum (400 MHz, DMSO-d_6) of **22**

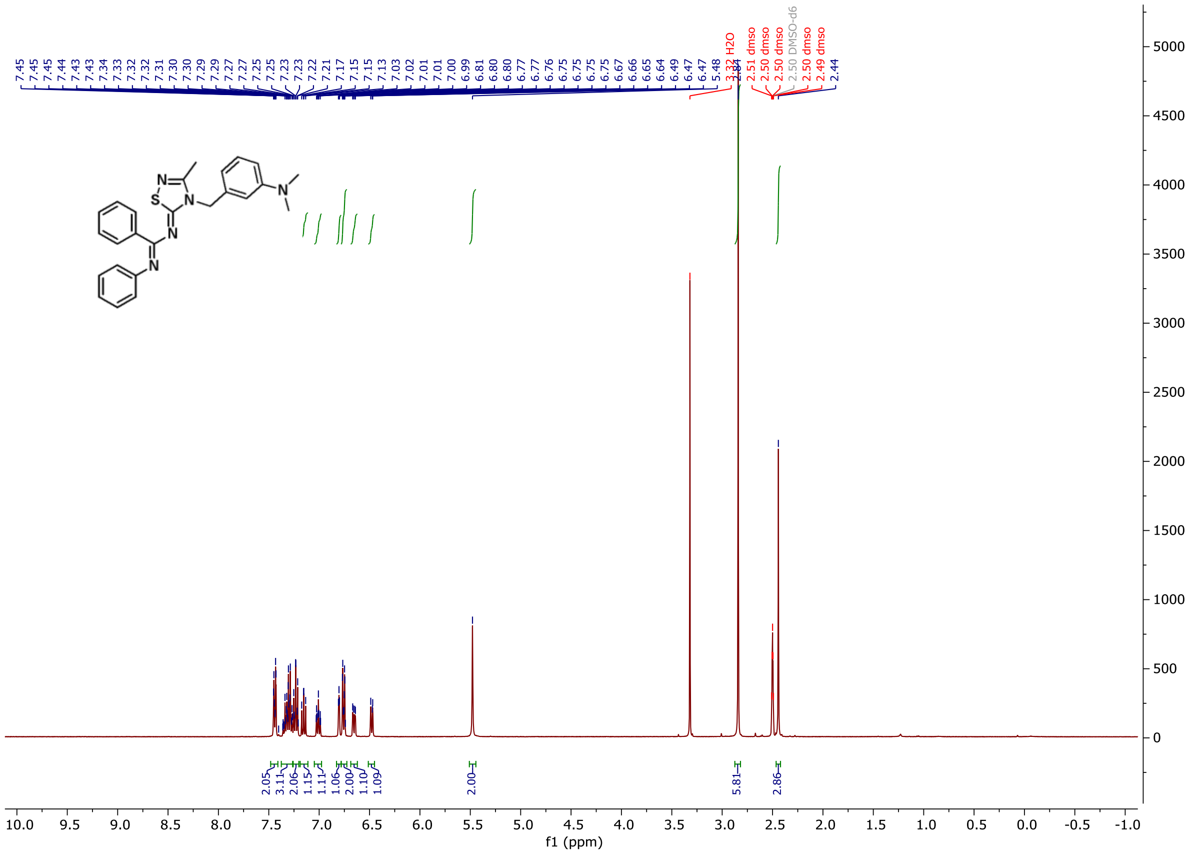

^{13}C-NMR spectrum (101 MHz, DMSO-d_6) of **22**

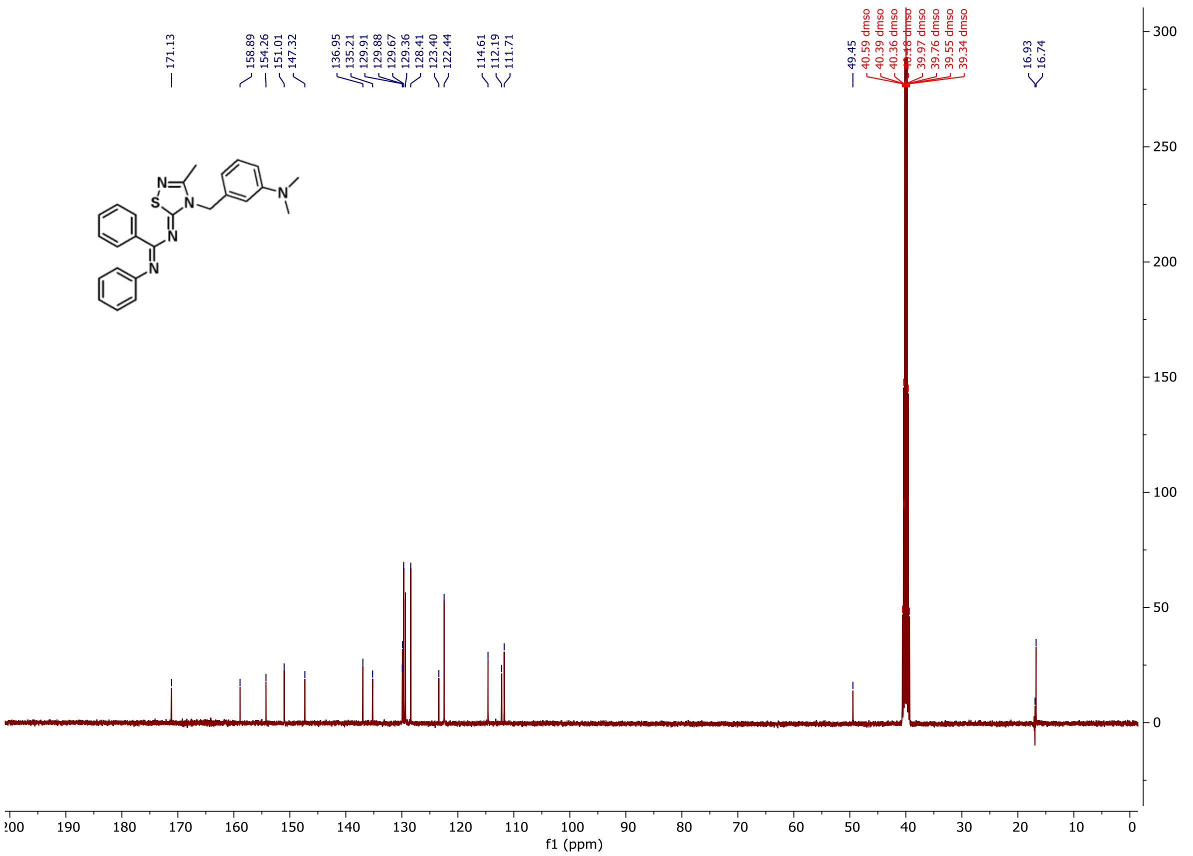

^{1}H-NMR spectrum (400 MHz, DMSO-d_6) of **23**

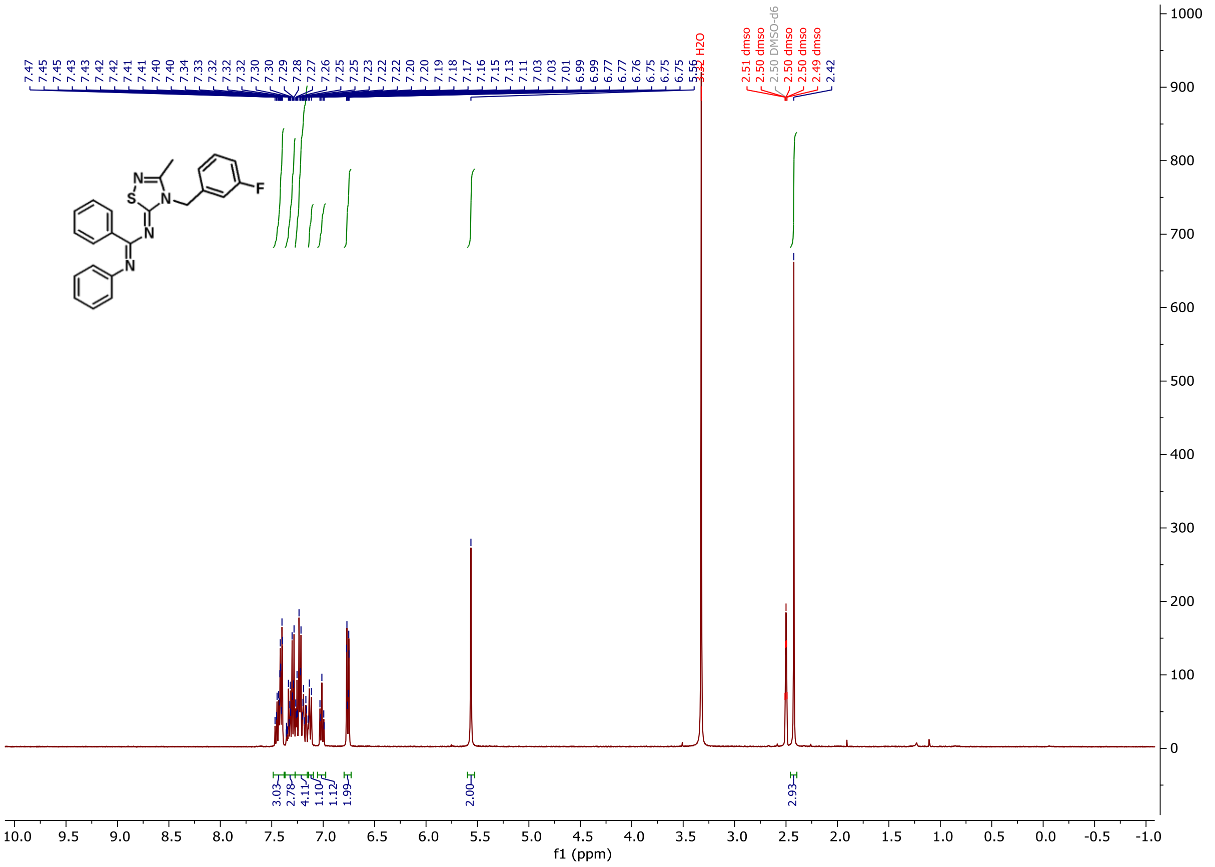

^{13}C-NMR spectrum (101 MHz, DMSO-d_6) of **23**

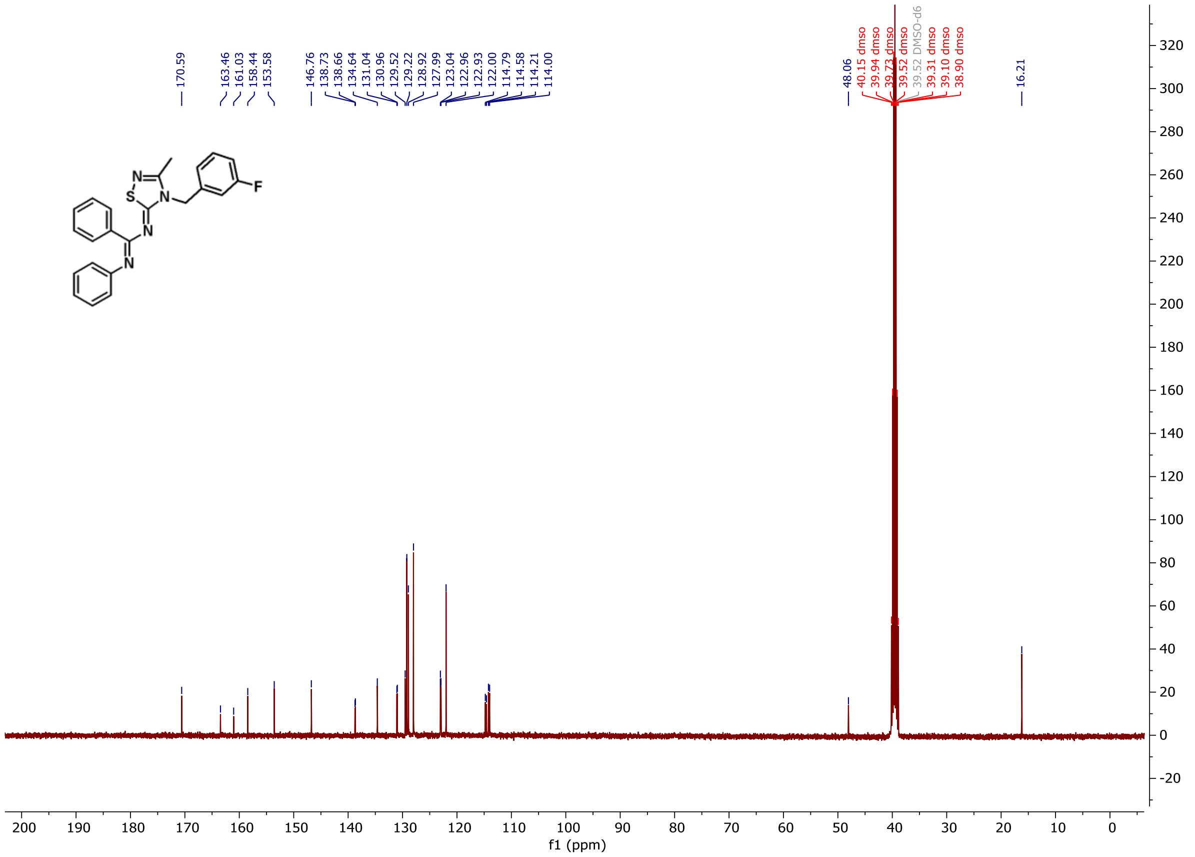

^{1}H-NMR spectrum (400 MHz, DMSO-d_6) of **24**

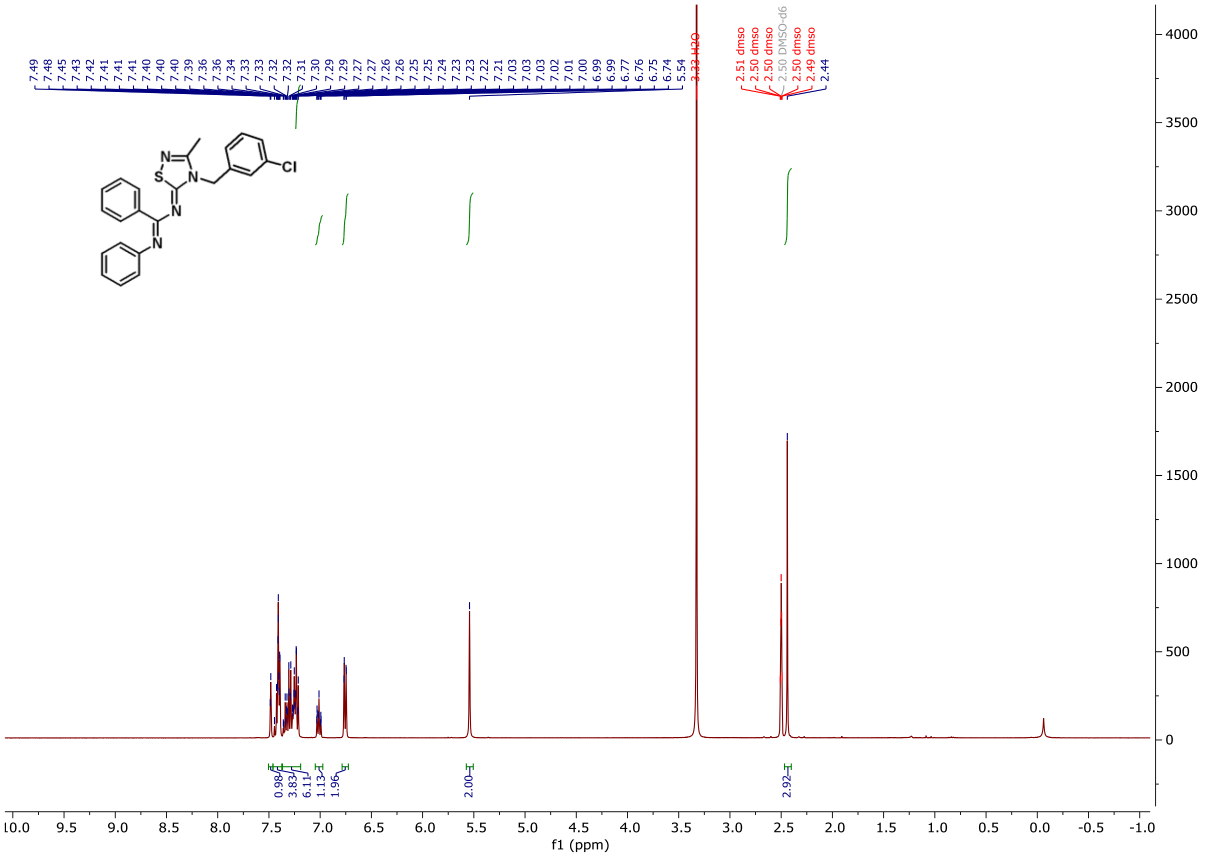

^{13}C-NMR spectrum (101 MHz, DMSO-d_6) of **24**

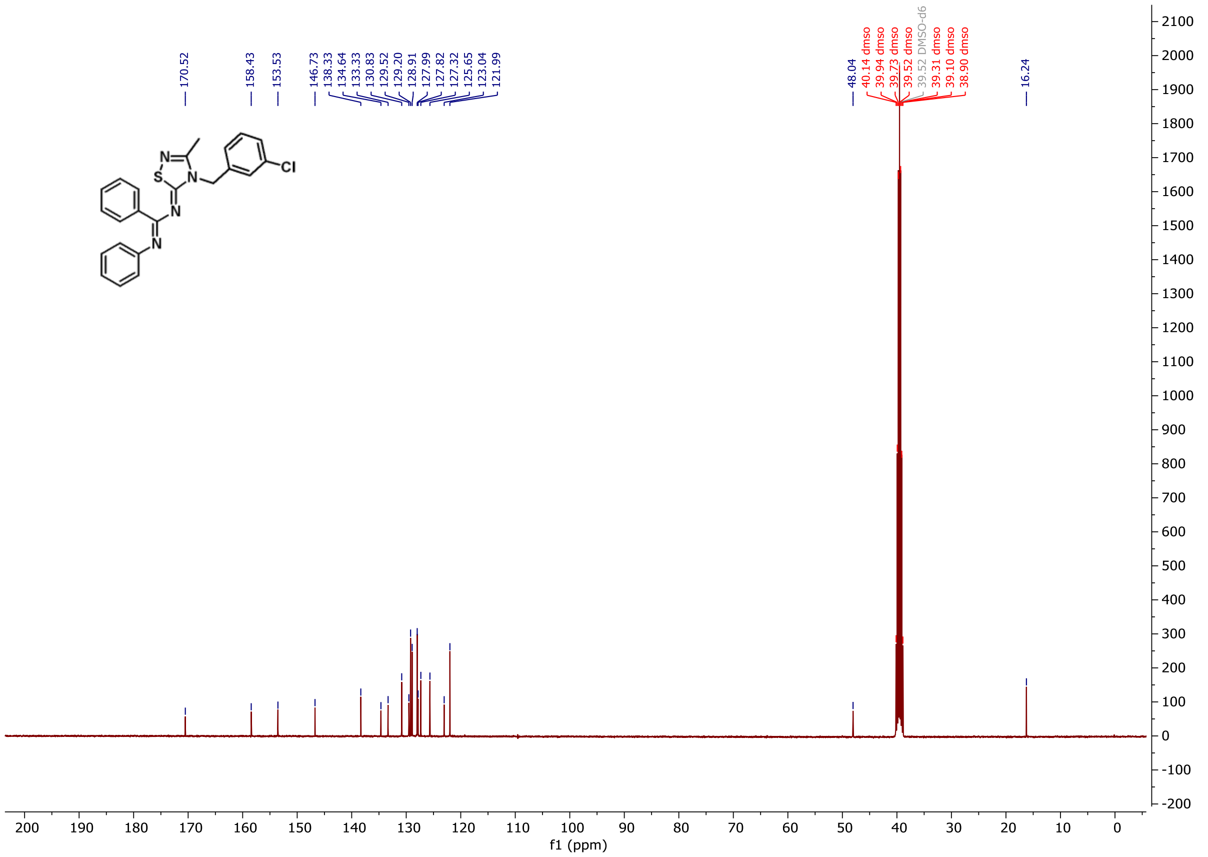

^{1}H-NMR spectrum (400 MHz, DMSO-d_6) of **25**

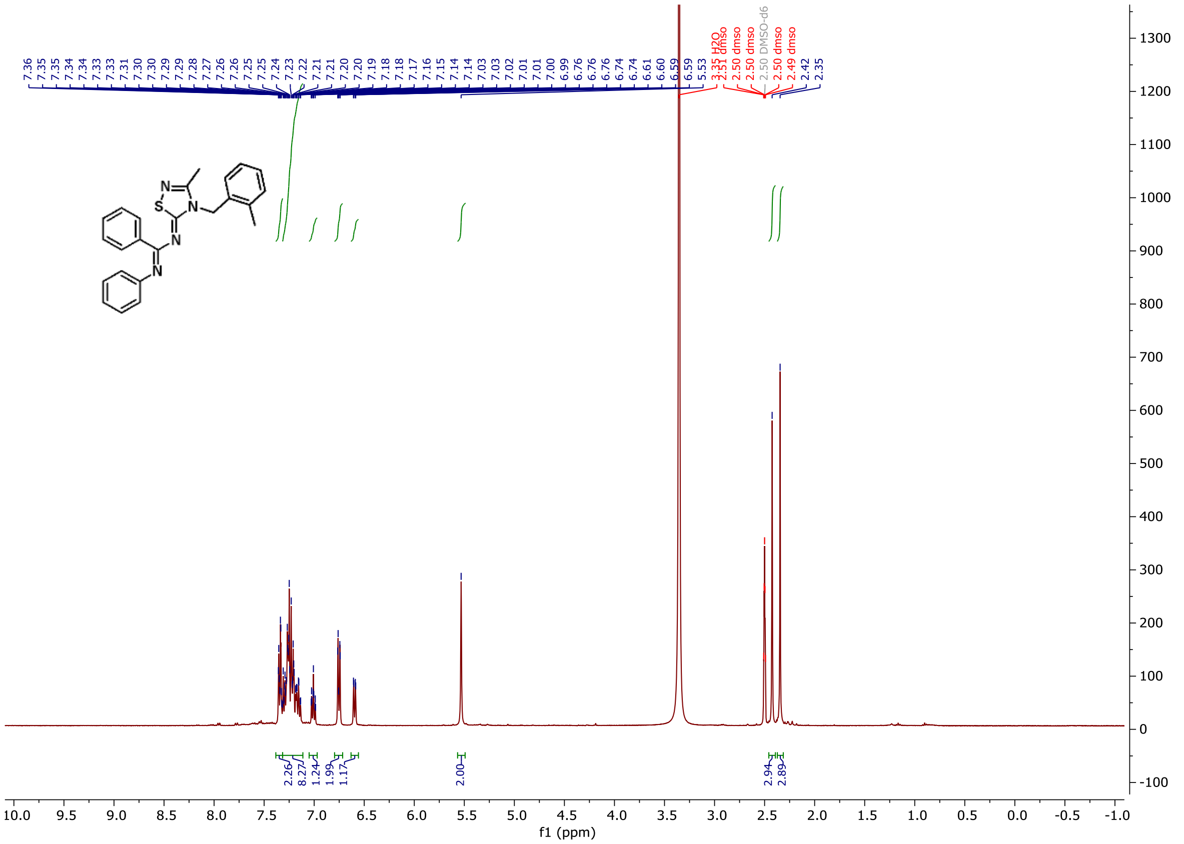

^{13}C-NMR spectrum (101 MHz, DMSO-d_6) of **25**

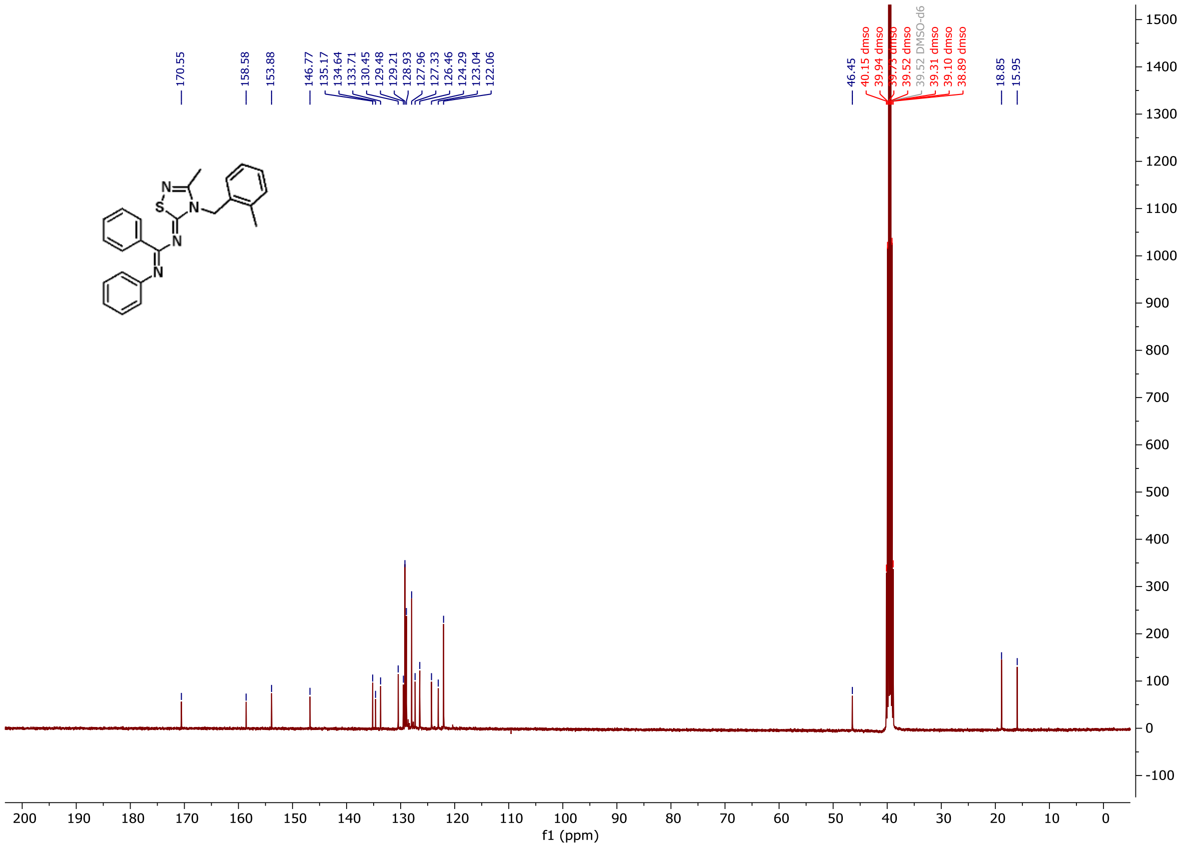

^{1}H-NMR spectrum (400 MHz, DMSO-d_6) of **26**

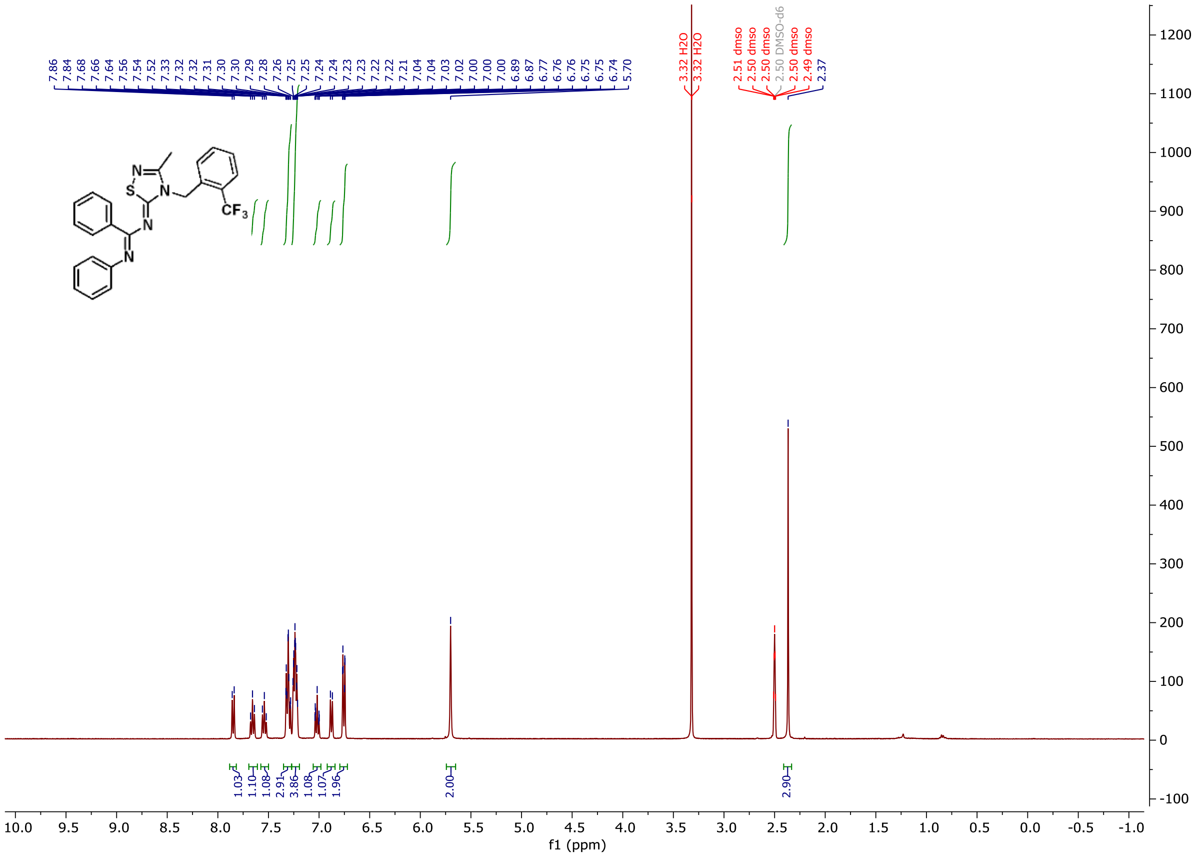

^{13}C-NMR spectrum (101 MHz, DMSO-d_6) of **26**

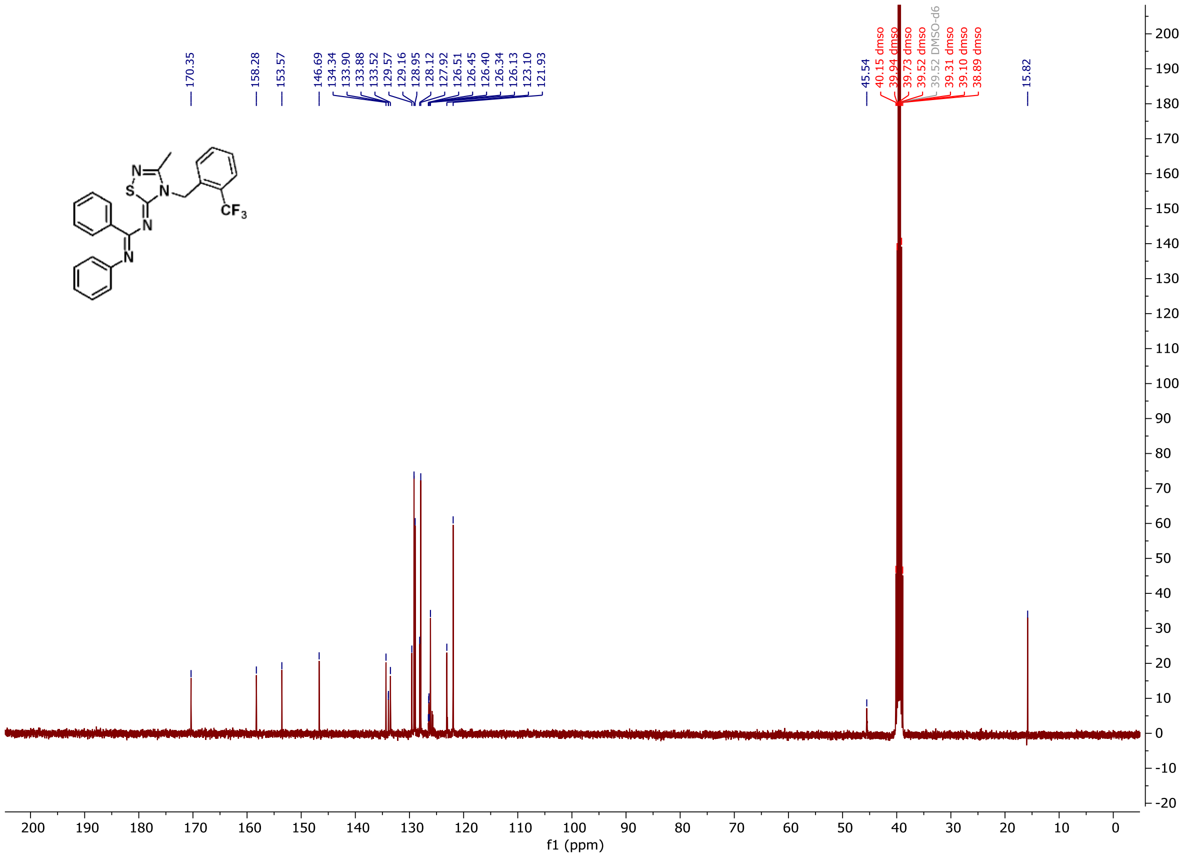

^{1}H-NMR spectrum (400 MHz, DMSO-d_6) of **27**

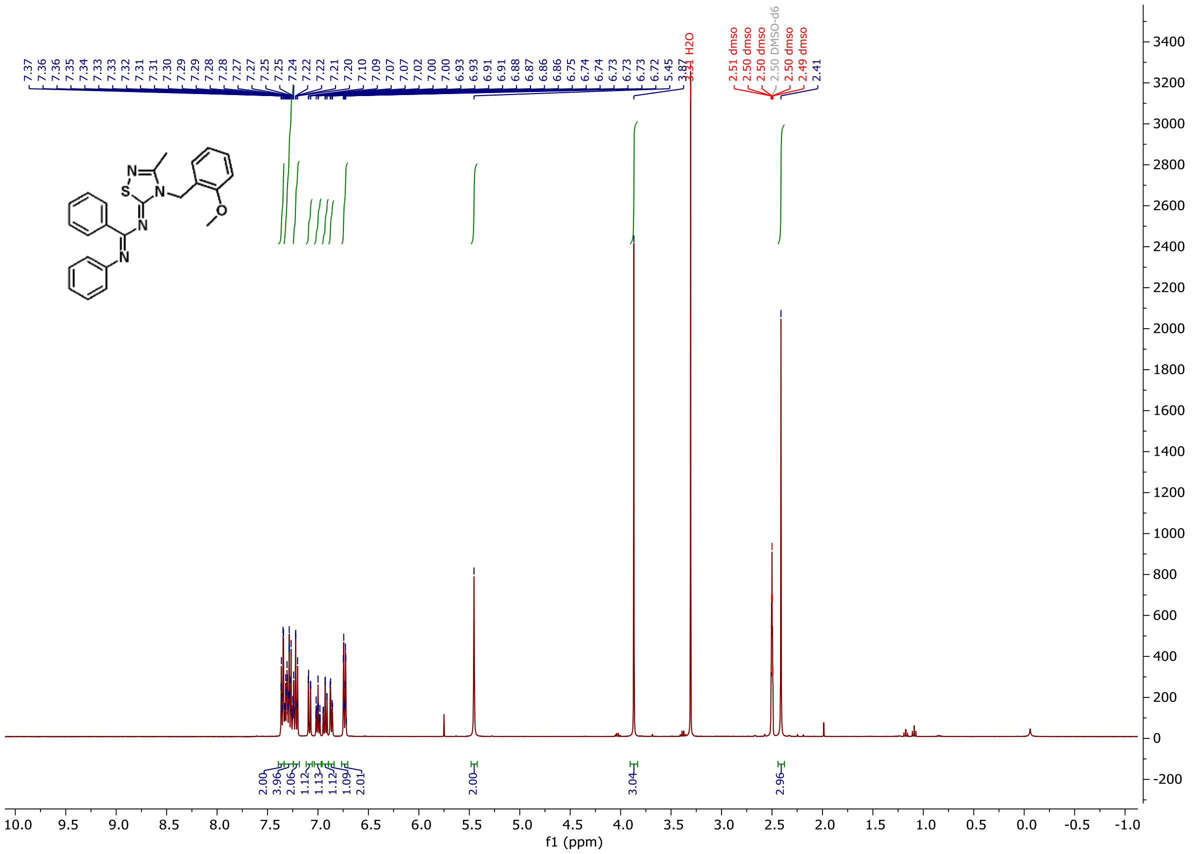

^{13}C-NMR spectrum (101 MHz, DMSO-d_6) of **27**

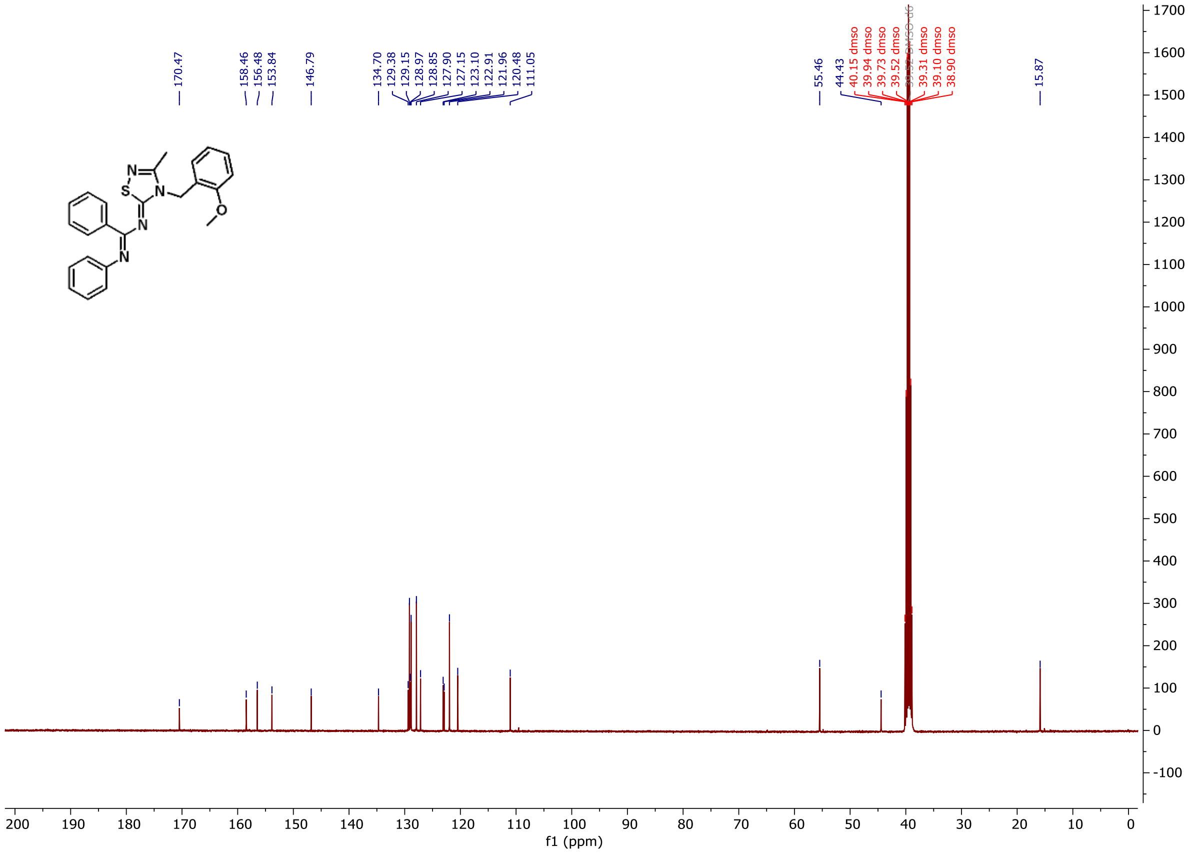

^{1}H-NMR spectrum (400 MHz, DMSO-d_6) of **28**

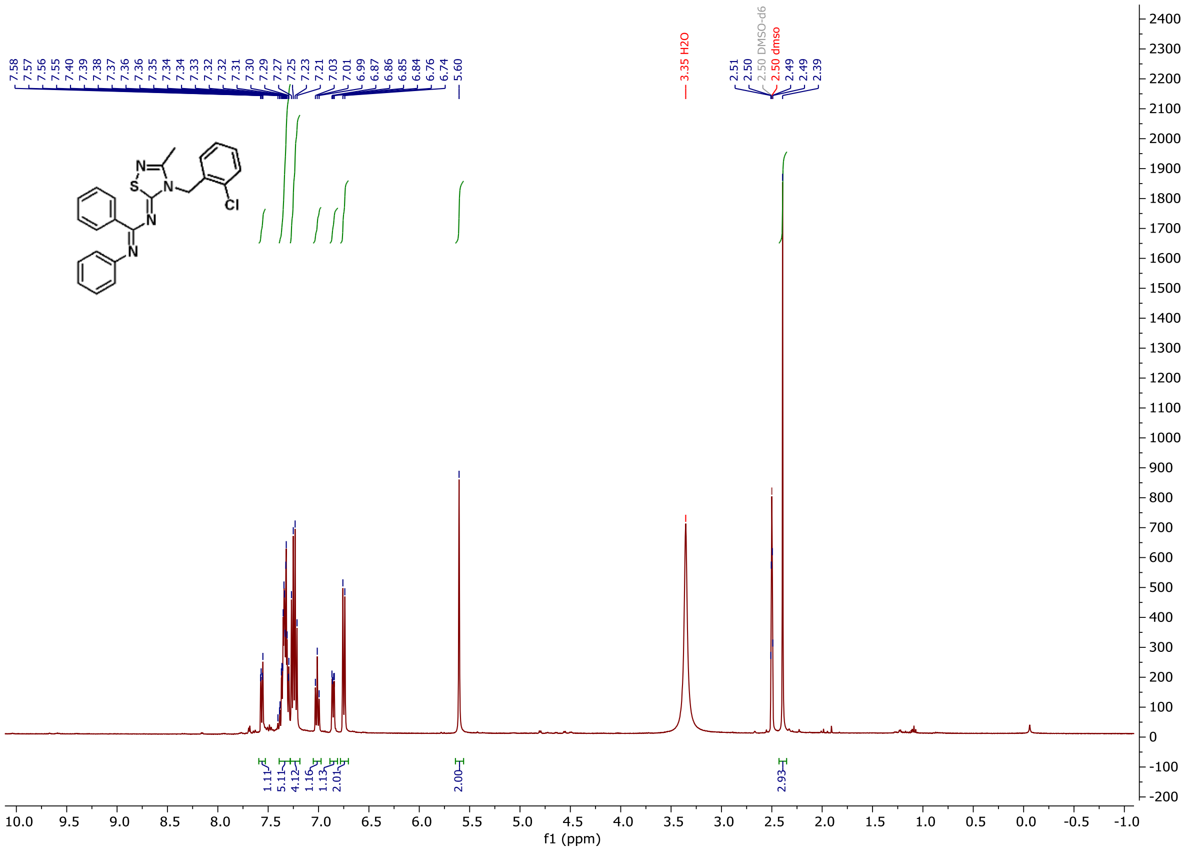

^{13}C-NMR spectrum (101 MHz, DMSO-d_6) of **28**

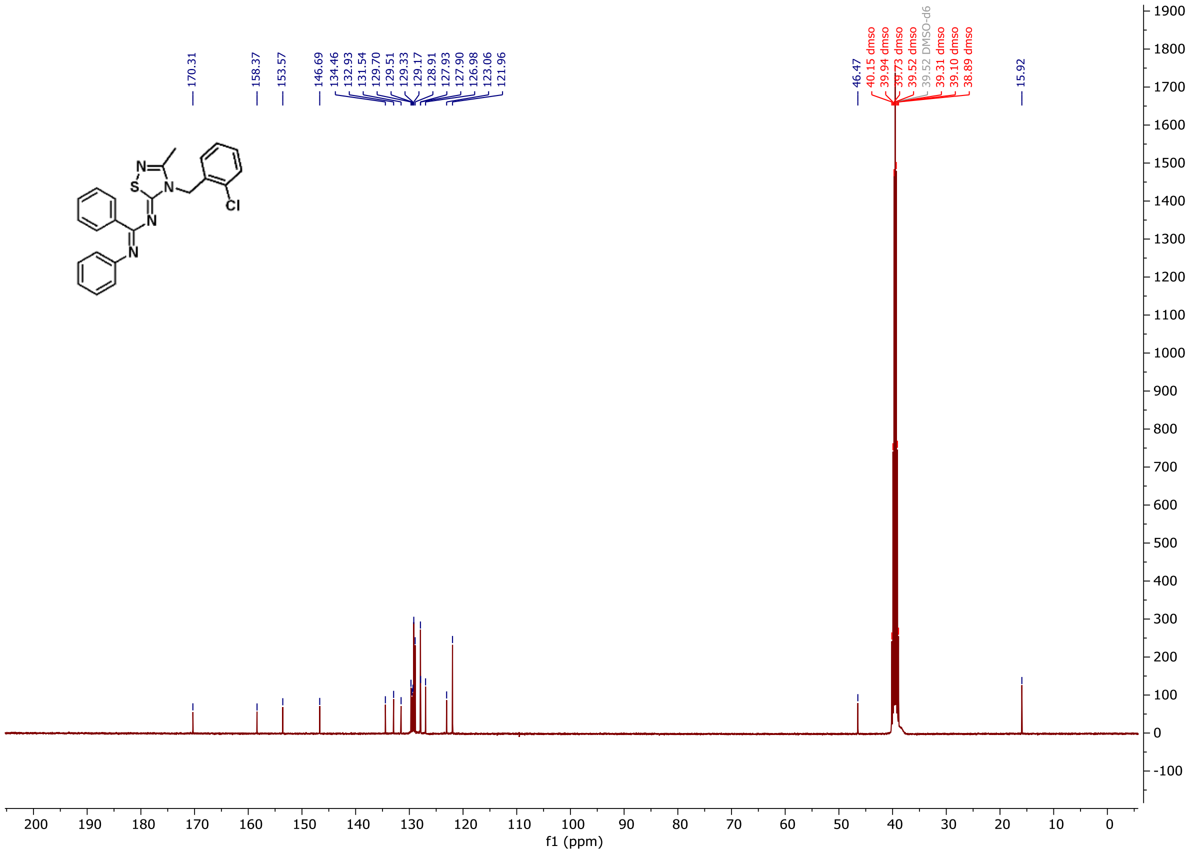

^{1}H-NMR spectrum (400 MHz, DMSO-d_6) of **29**

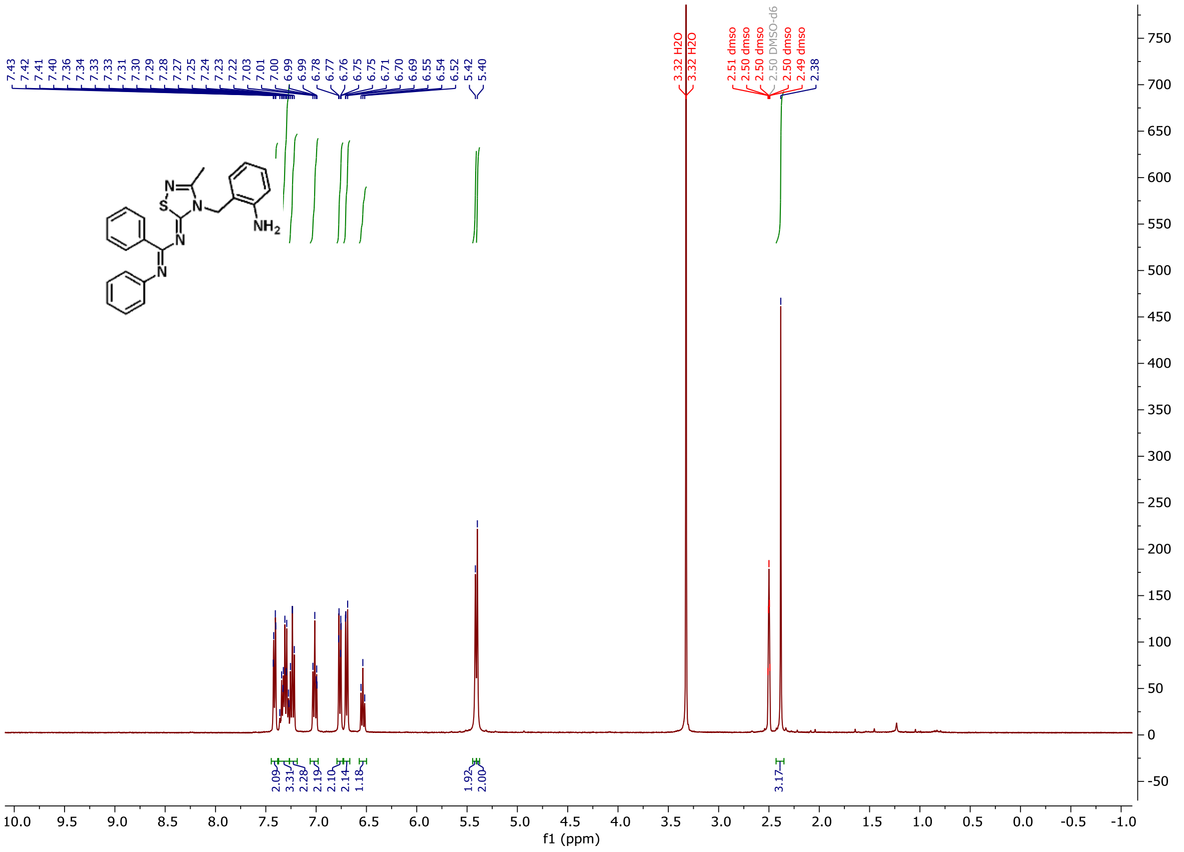

^{13}C-NMR spectrum (101 MHz, DMSO-d_6) of **29**

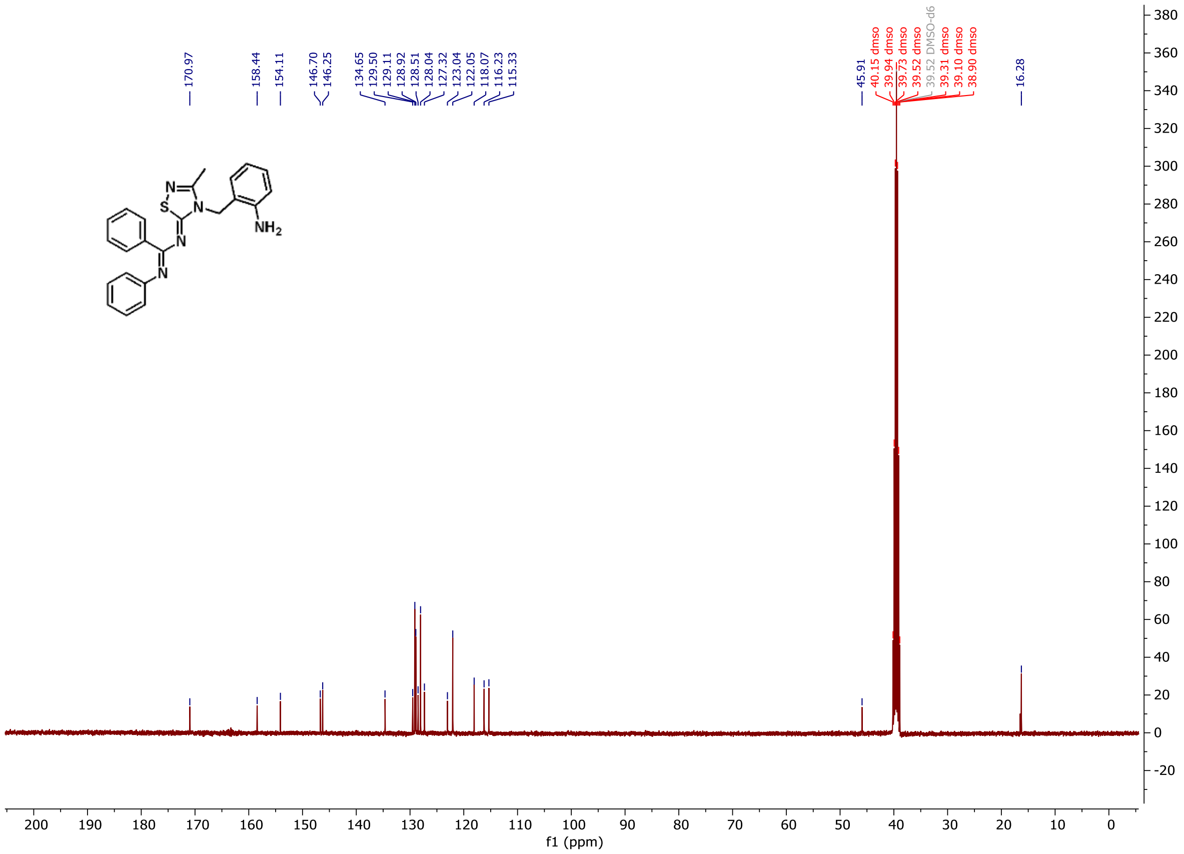

^{1}H-NMR spectrum (400 MHz, DMSO-d_6) of **30**

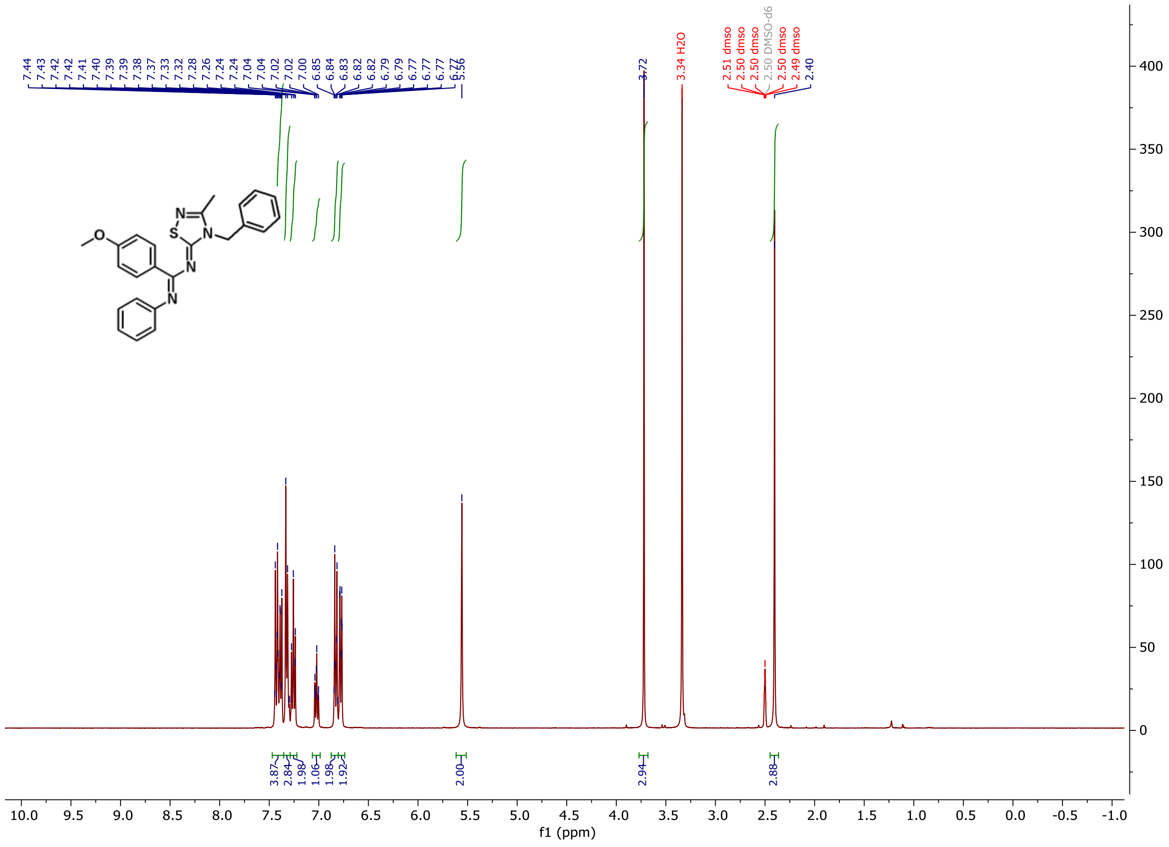

^{13}C-NMR spectrum (101 MHz, DMSO-d_6) of **30**

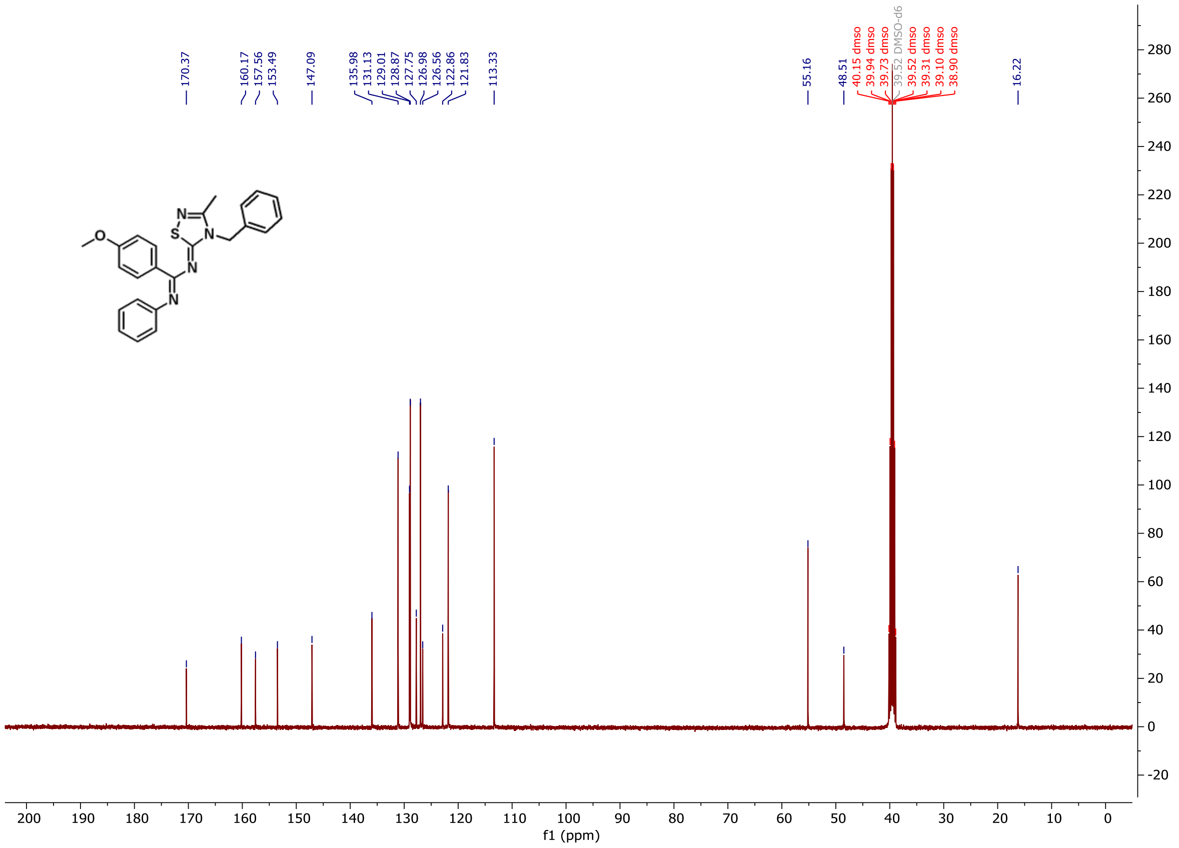

^{1}H-NMR spectrum (400 MHz, DMSO-d_6) of **31**

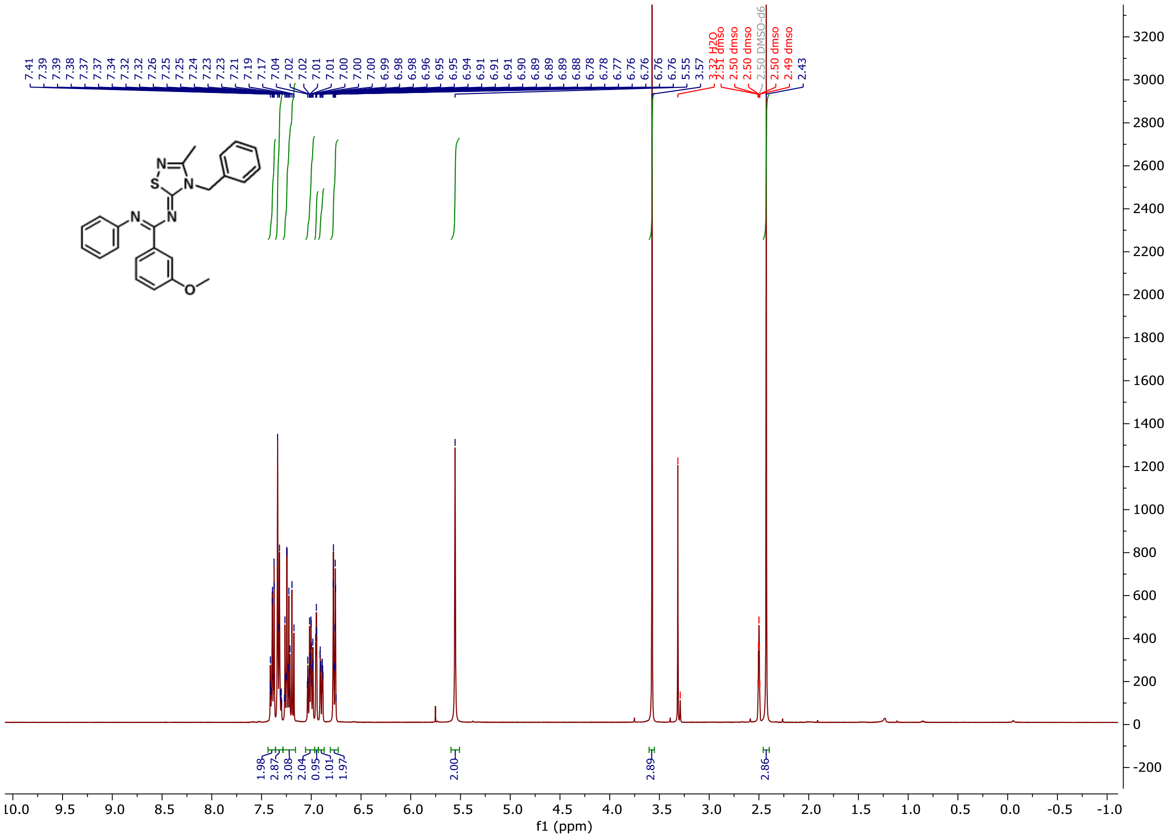

^{13}C-NMR spectrum (101 MHz, DMSO-d_6) of **31**

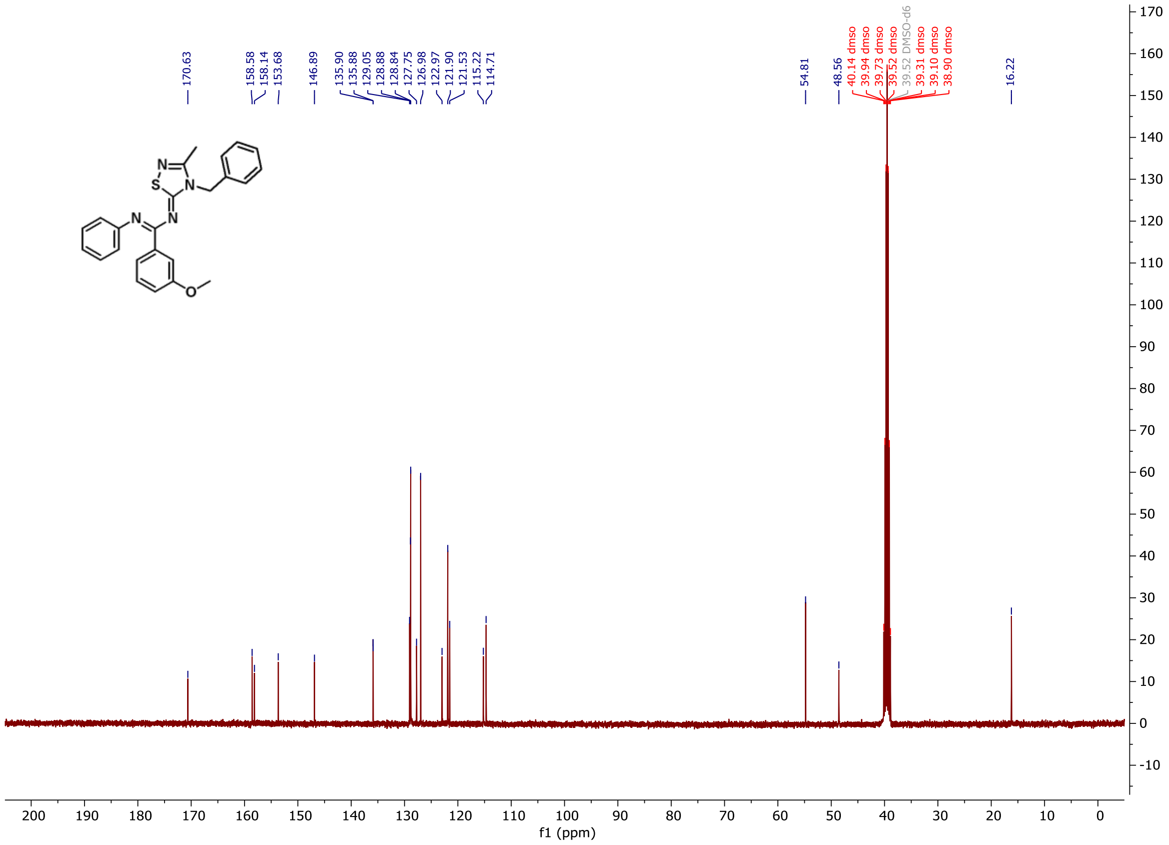

^{1}H-NMR spectrum (400 MHz, DMSO-d_6) of **32**

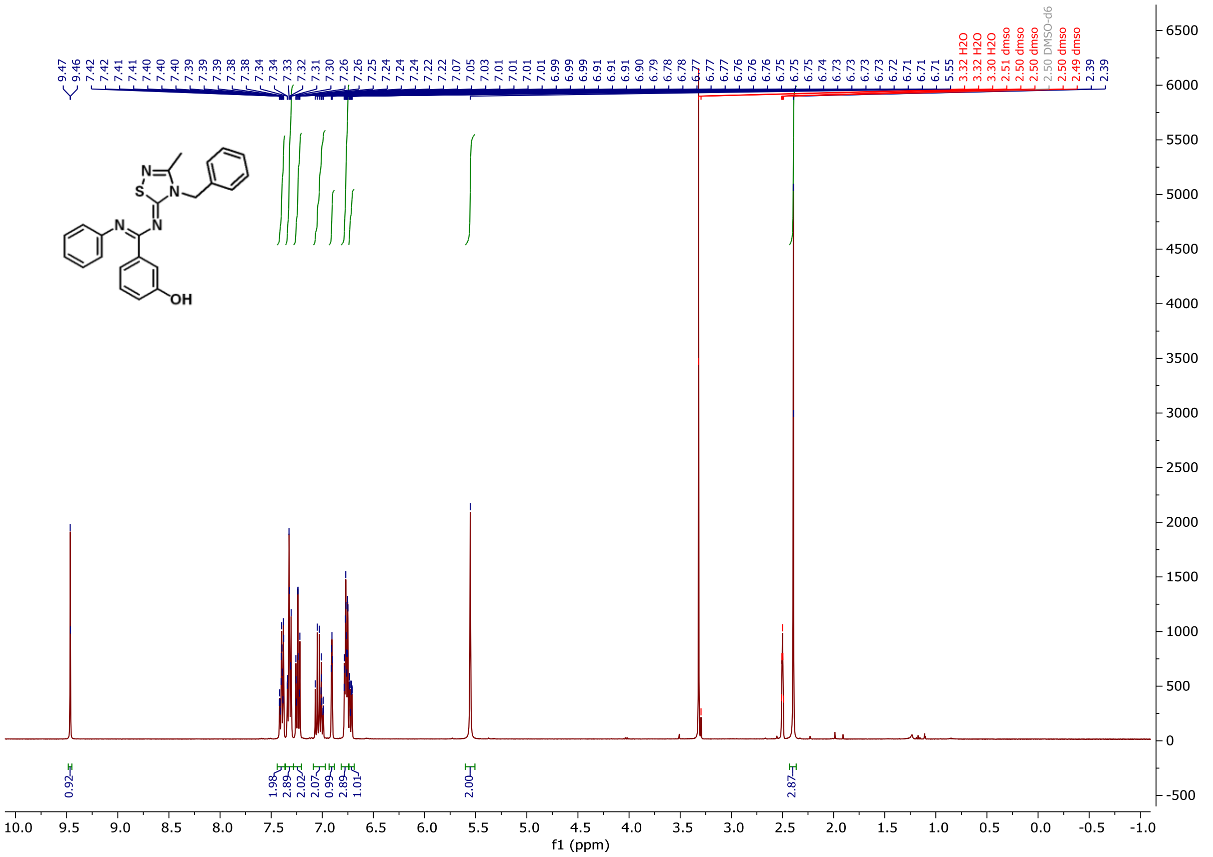

^{13}C-NMR spectrum (101 MHz, DMSO-d_6) of **32**

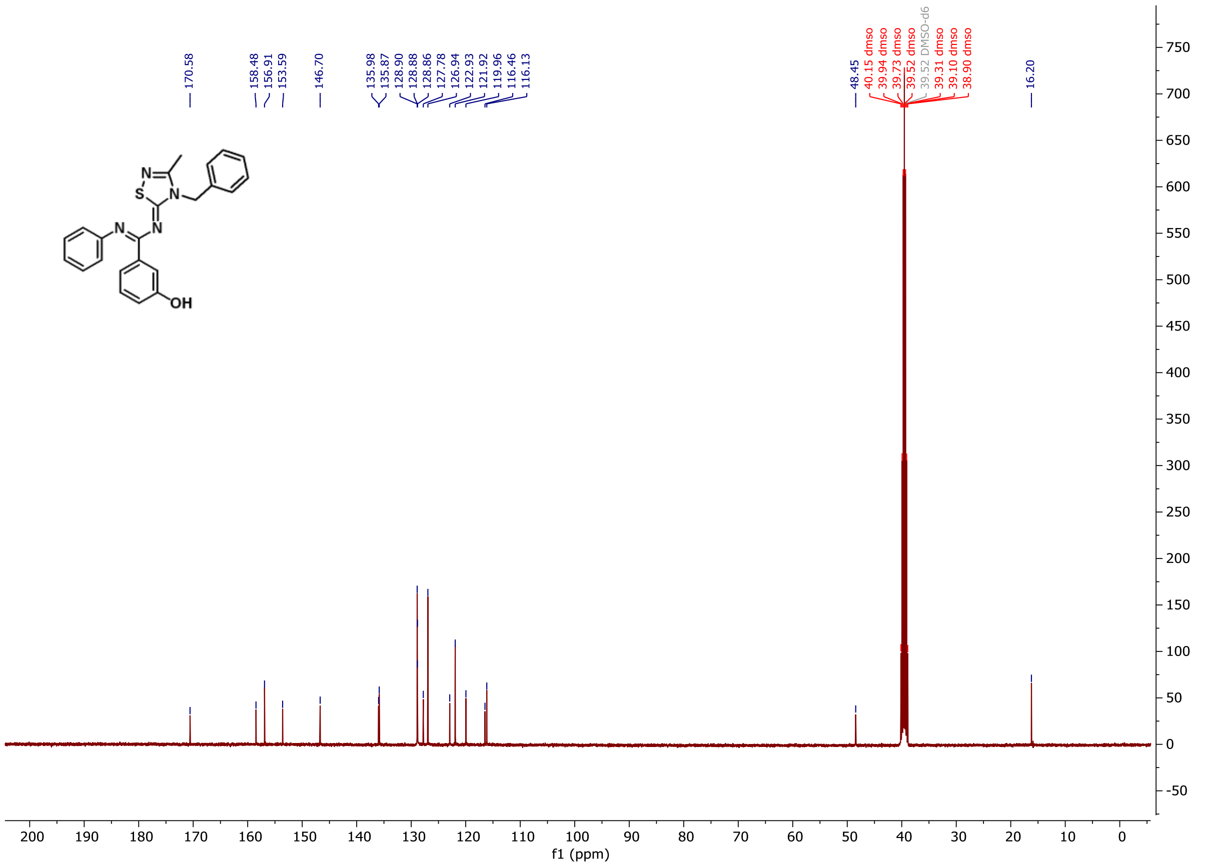

^{1}H-NMR spectrum (400 MHz, DMSO-d_6) of **33**

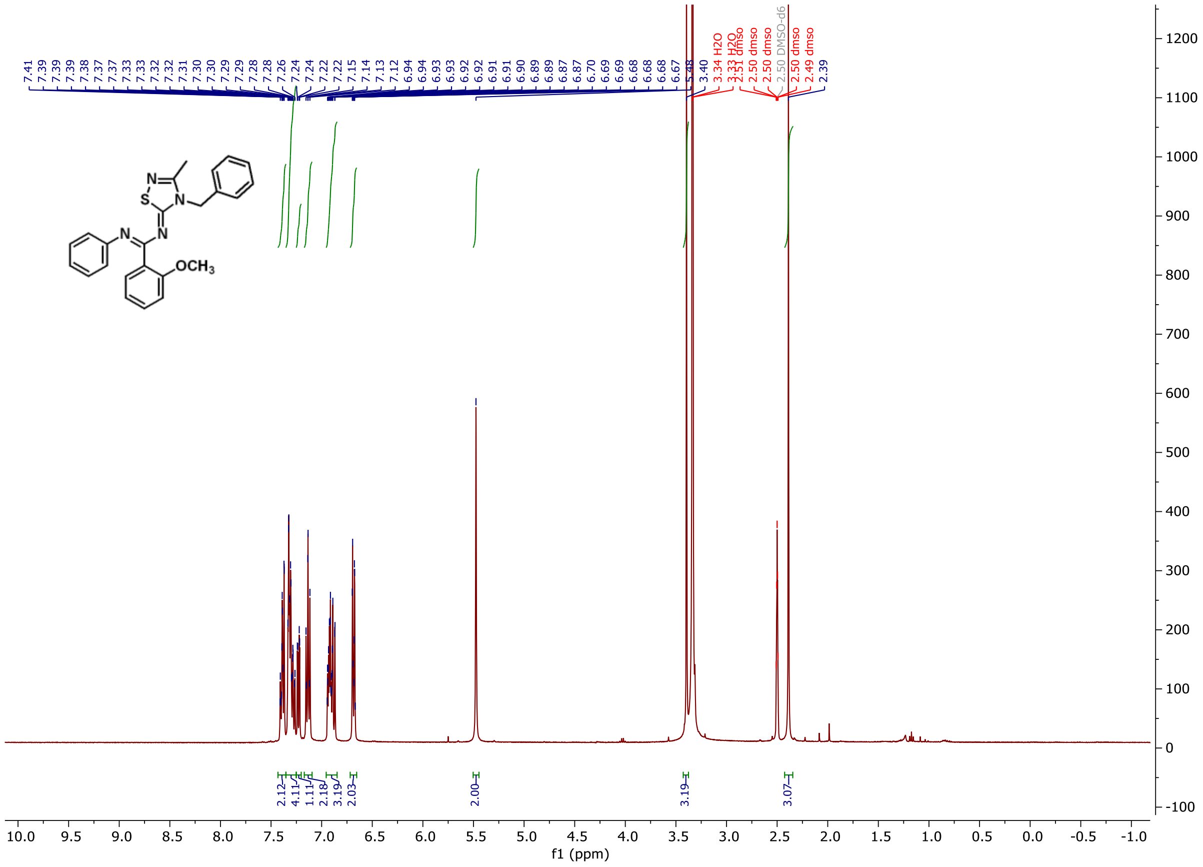

^{13}C-NMR spectrum (101 MHz, DMSO-d_6) of **33**

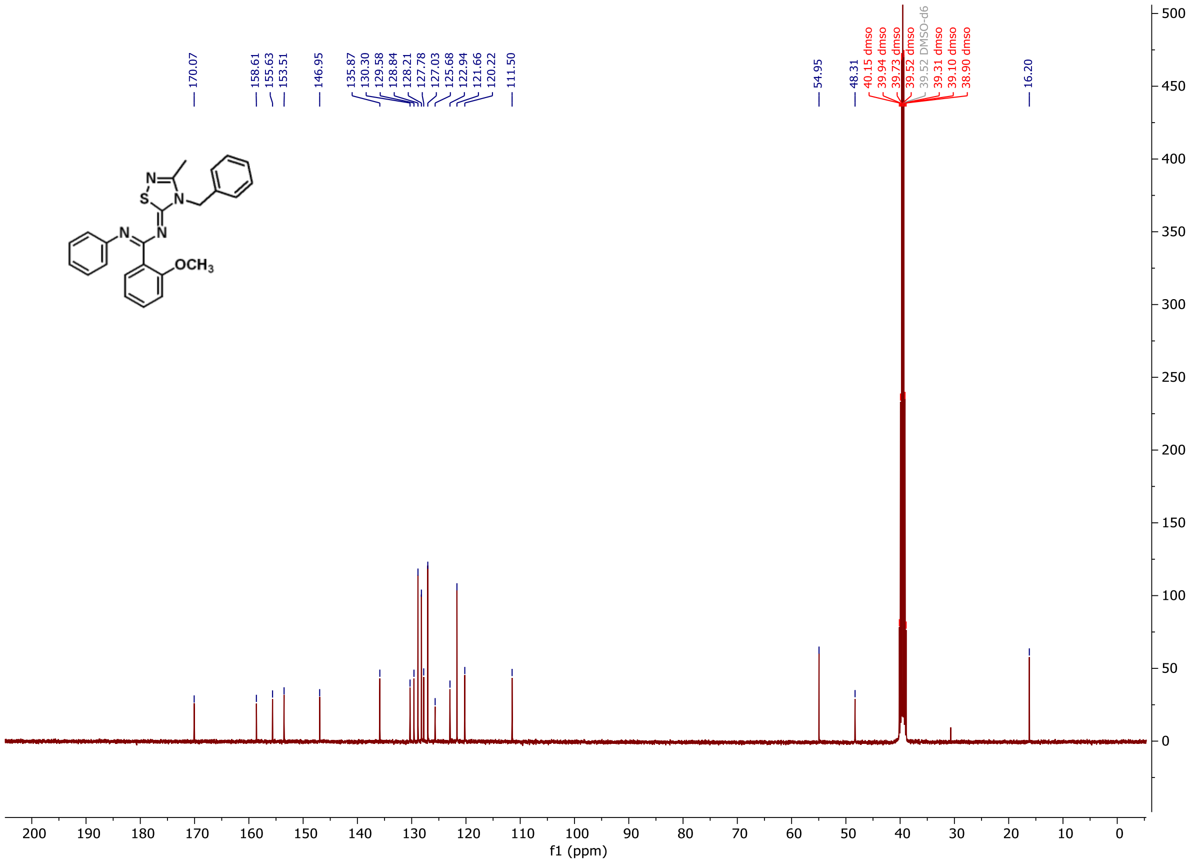

^{1}H-NMR spectrum (400 MHz, DMSO-d_6) of **34**

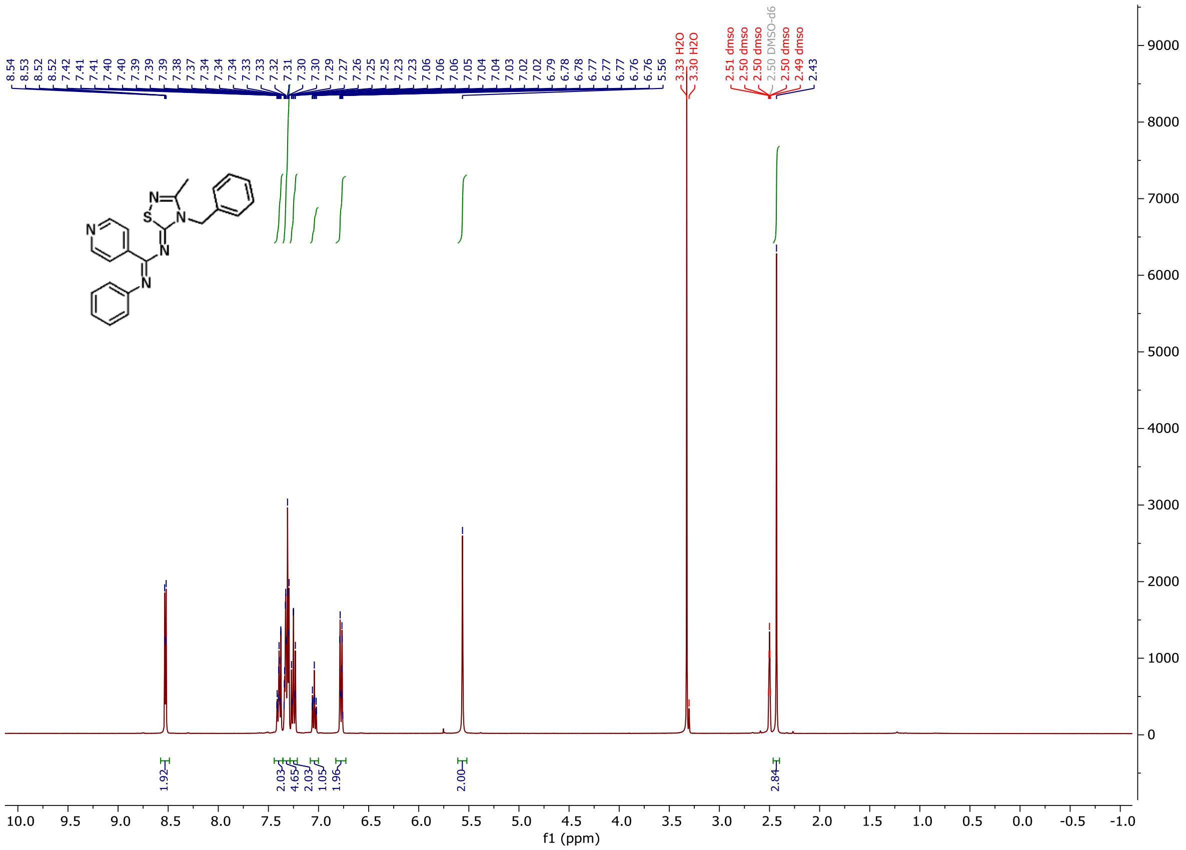

^{13}C-NMR spectrum (101 MHz, DMSO-d_6) of **34**

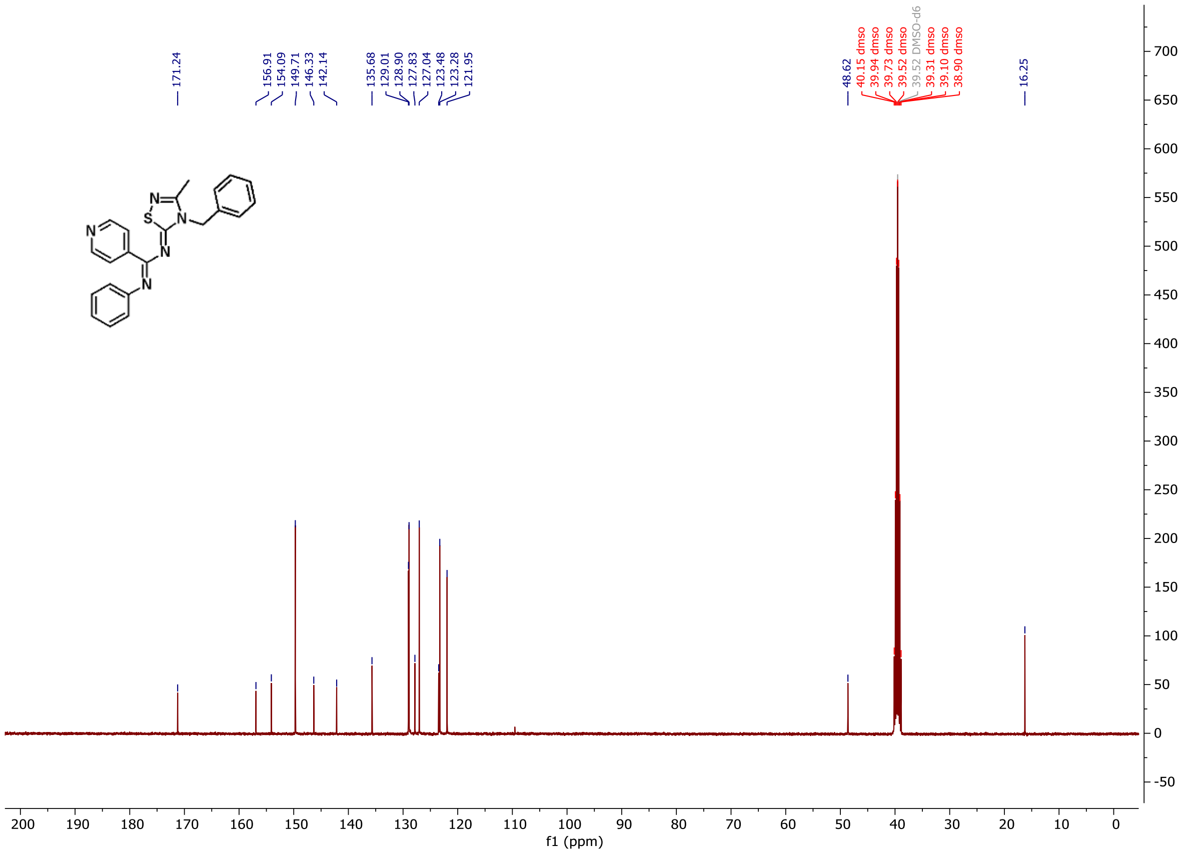

^{1}H-NMR spectrum (400 MHz, DMSO-d_6) of **35**

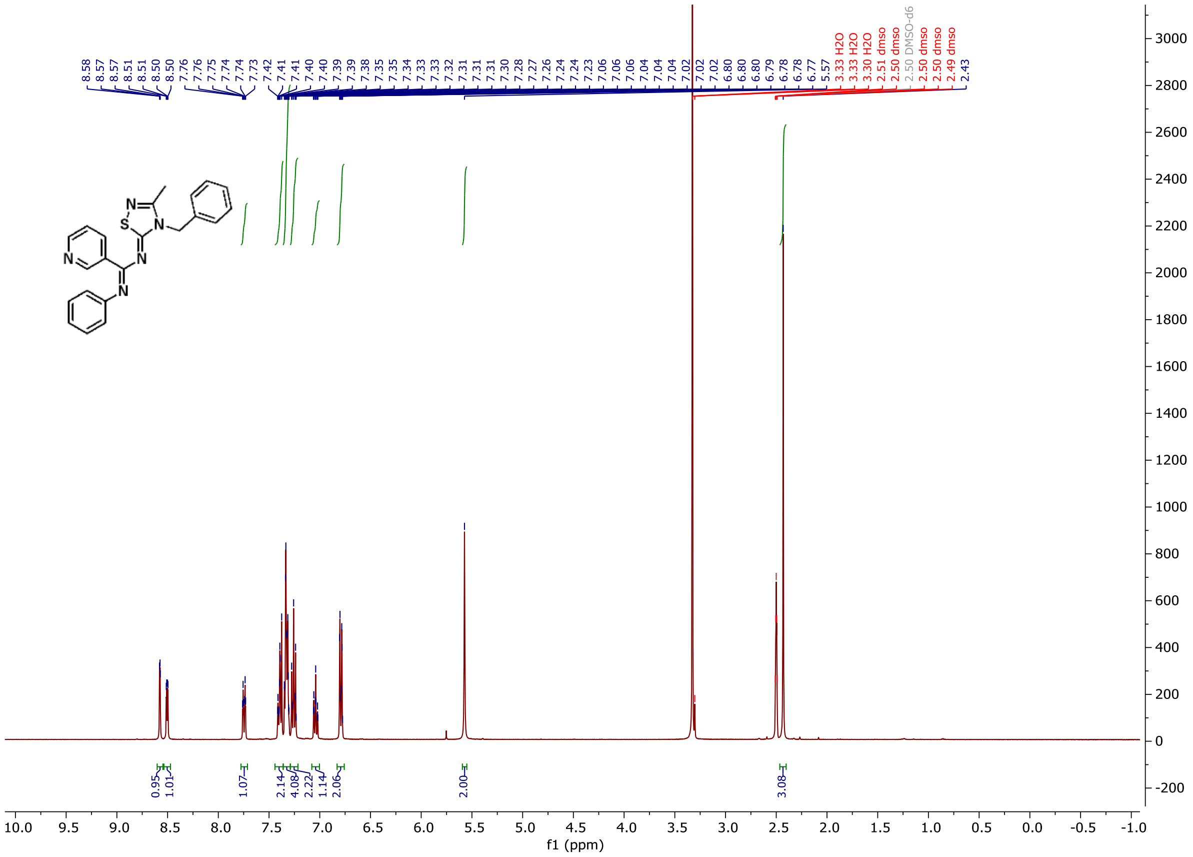

^{13}C-NMR spectrum (101 MHz, DMSO-d_6) of **35**

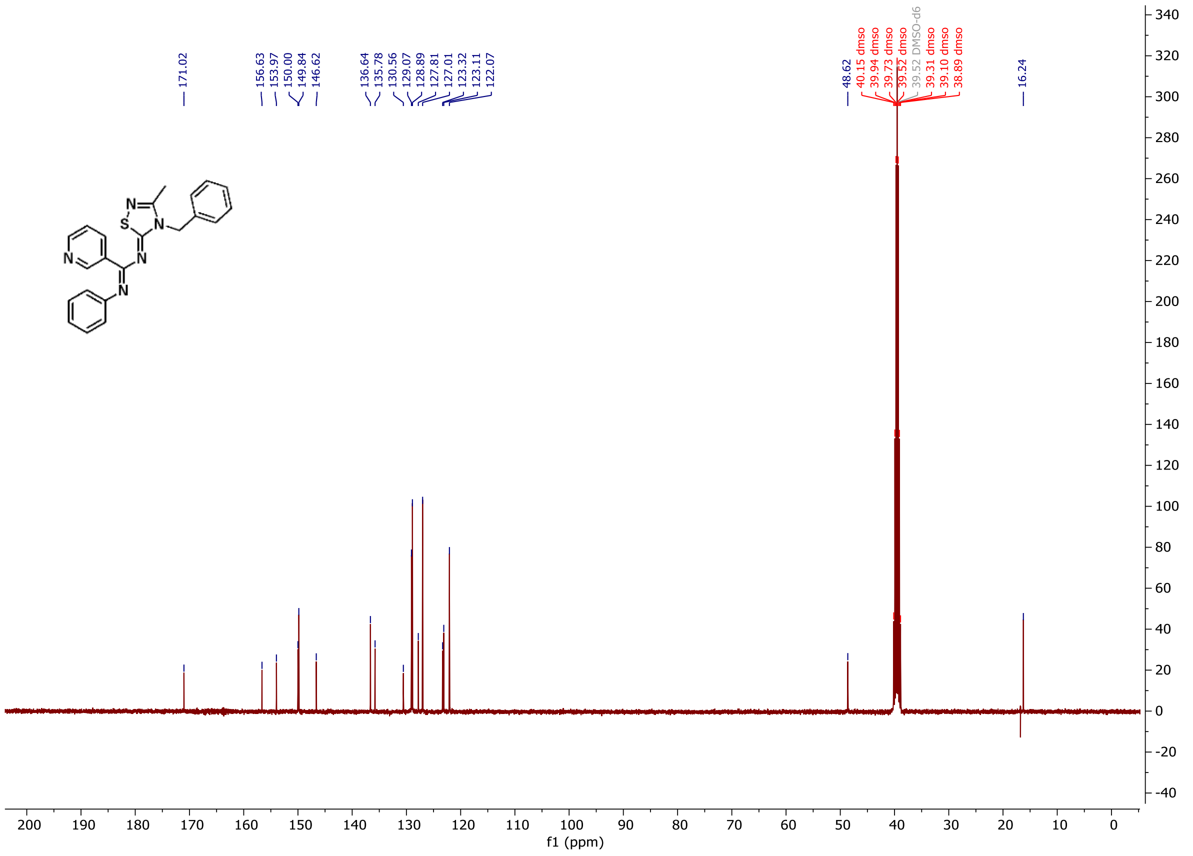

^{1}H-NMR spectrum (400 MHz, DMSO-d_6) of **36**

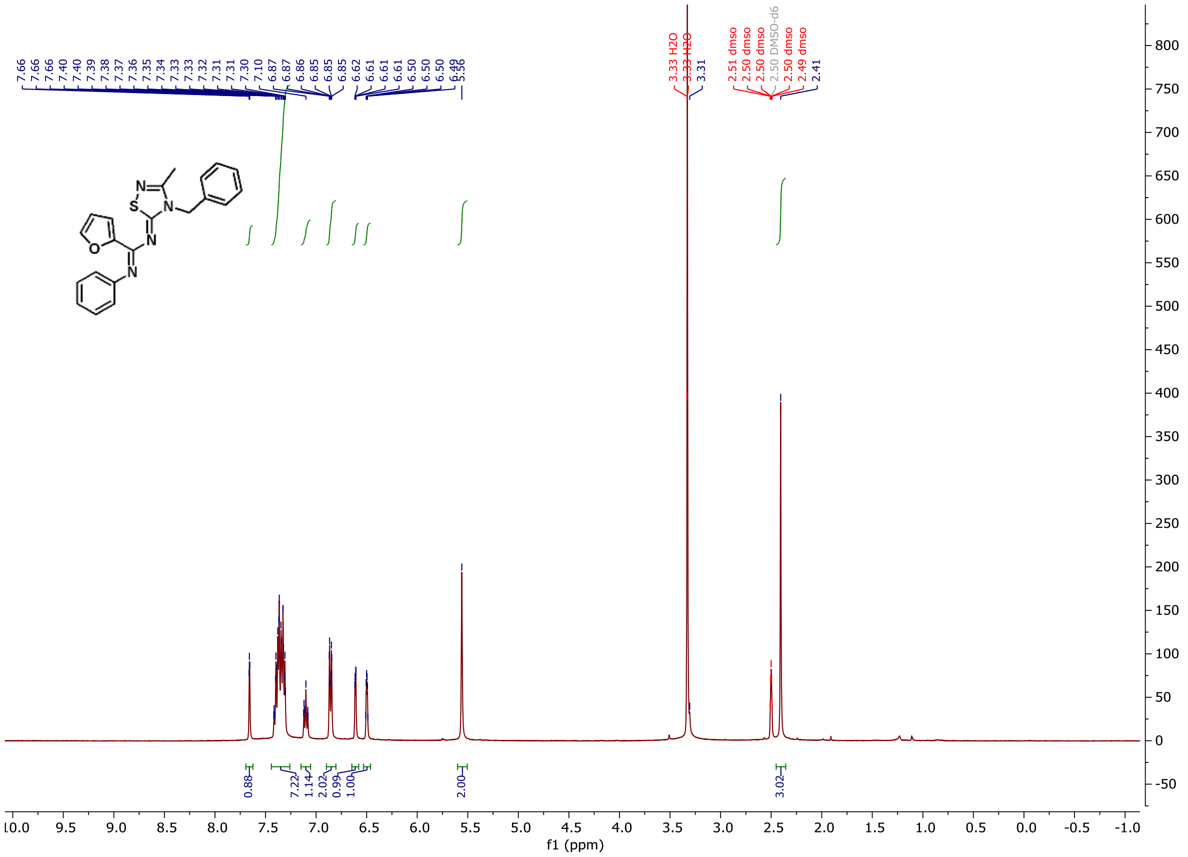

^{13}C-NMR spectrum (101 MHz, DMSO-d_6) of **36**

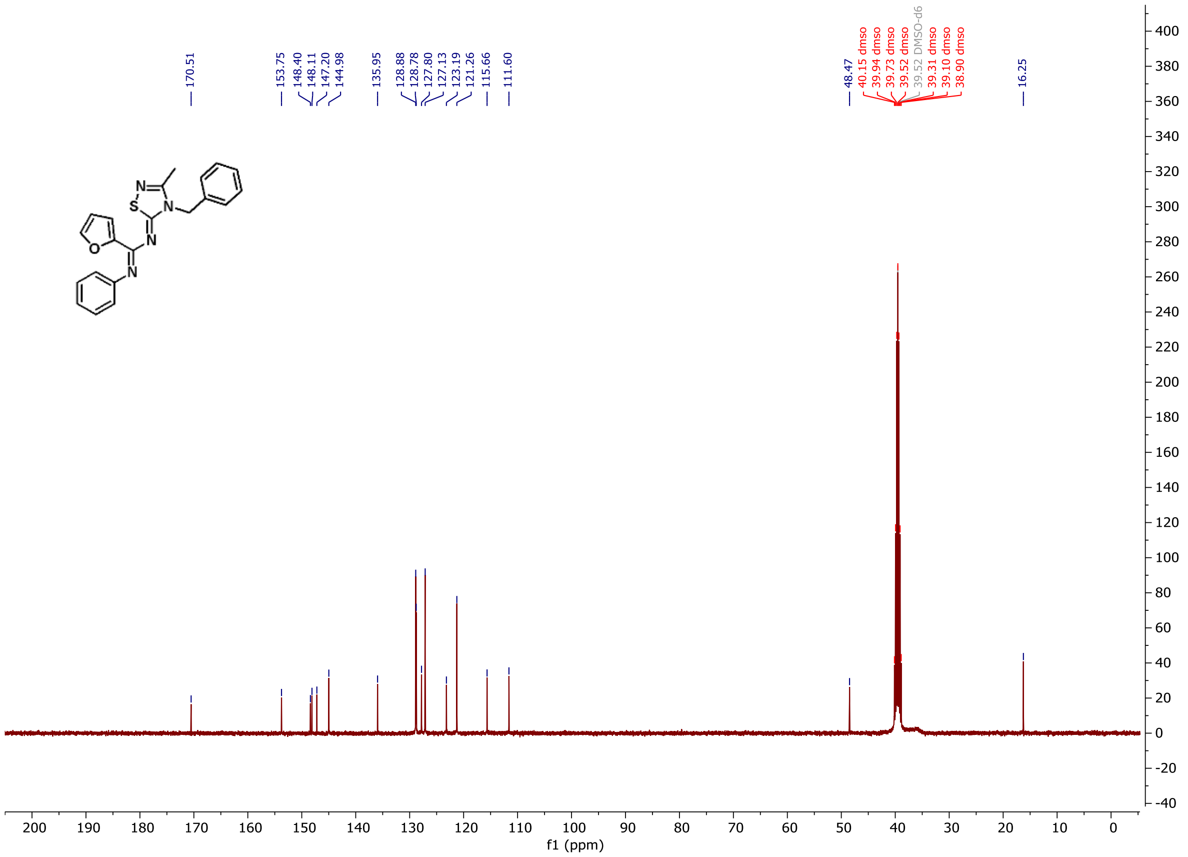

^{1}H-NMR spectrum (400 MHz, DMSO-d_6) of **37**

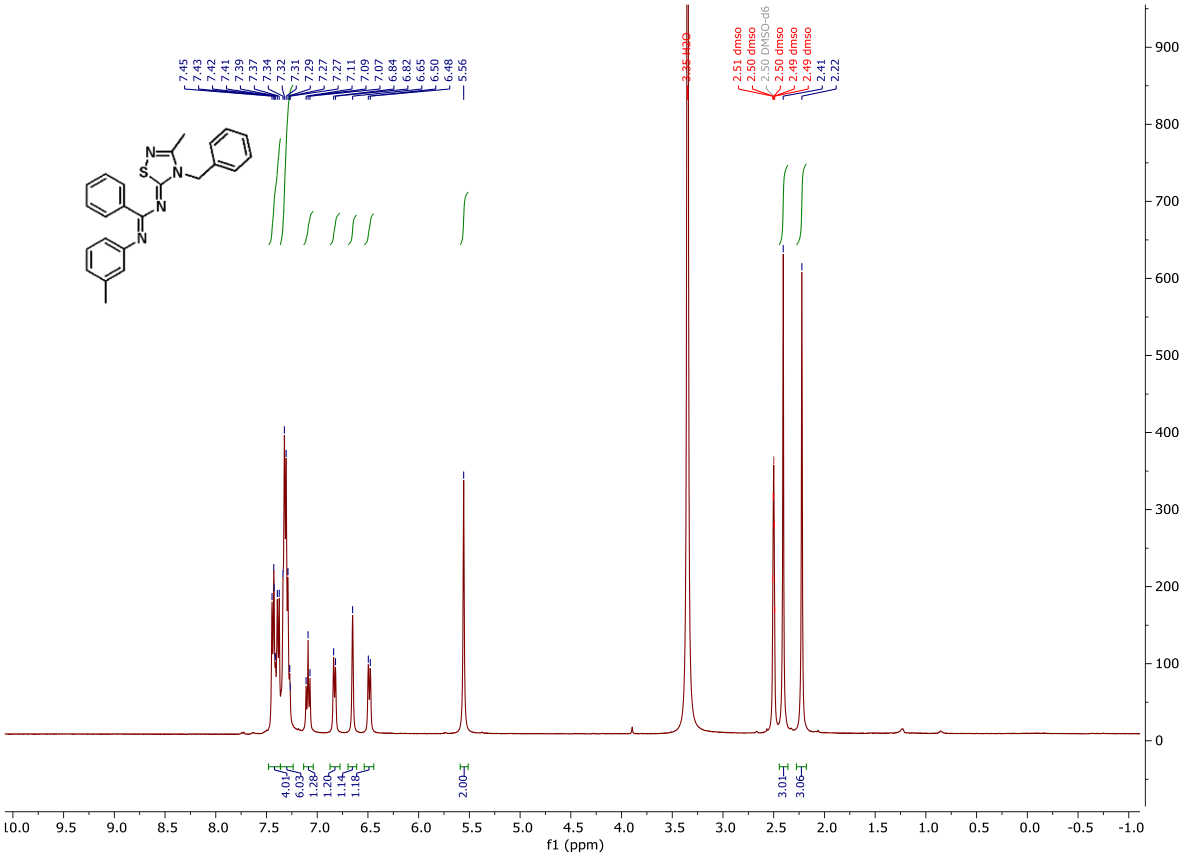

^{13}C-NMR spectrum (101 MHz, DMSO-d_6) of **37**

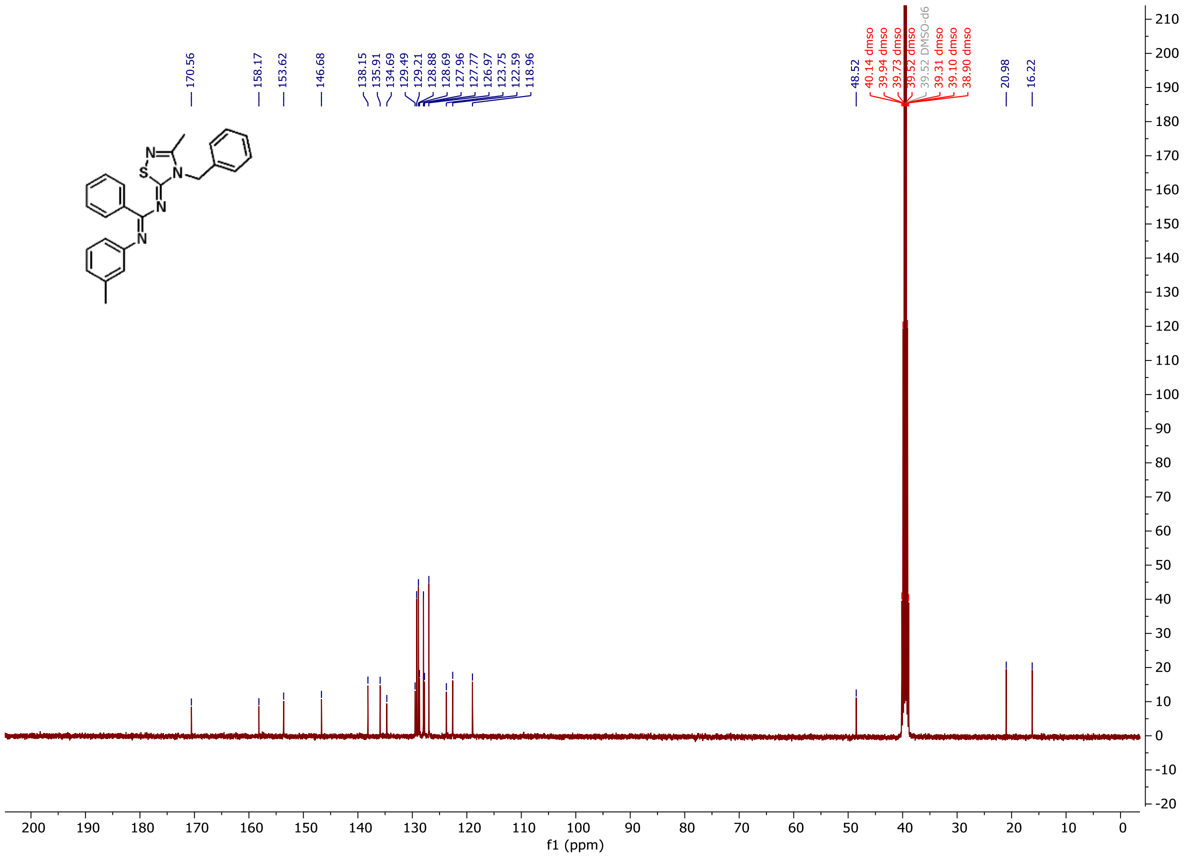

^{1}H-NMR spectrum (400 MHz, DMSO-d_6) of **38**

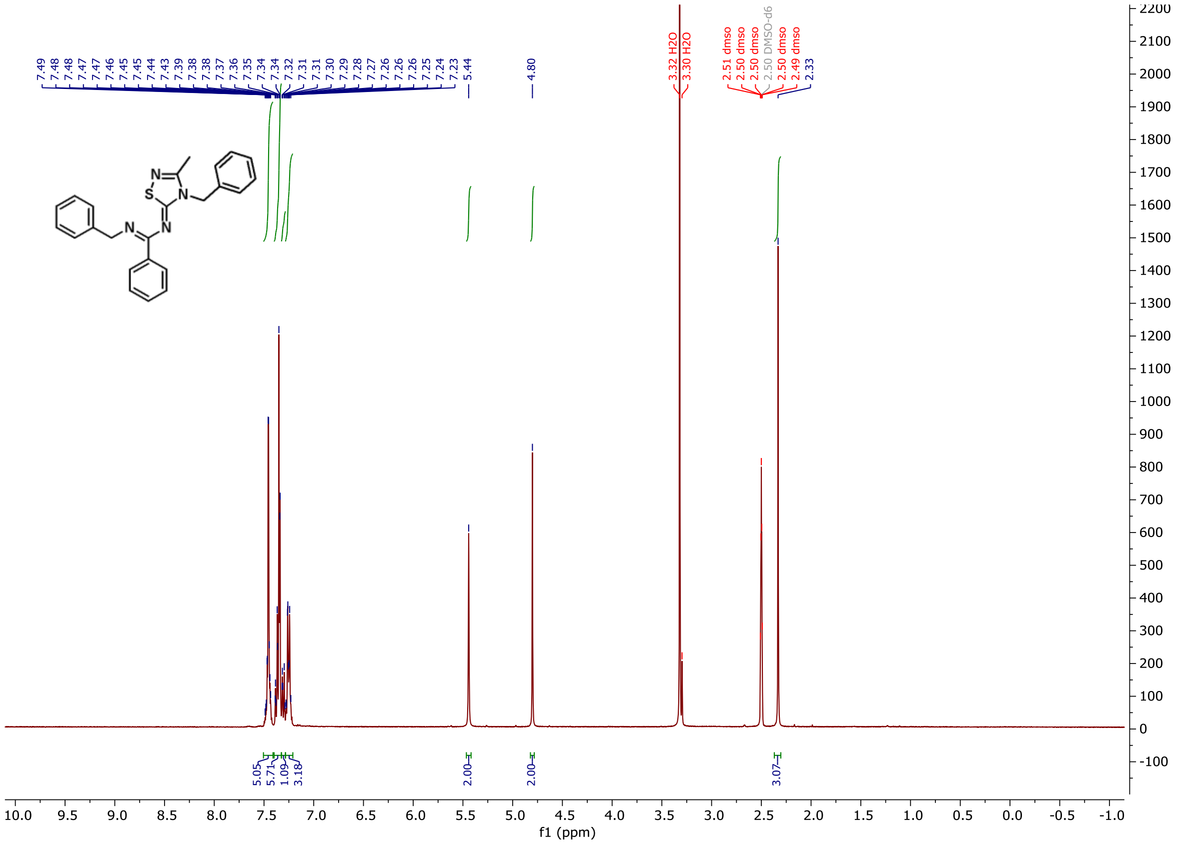

^{13}C-NMR spectrum (101 MHz, DMSO-d_6) of **38**

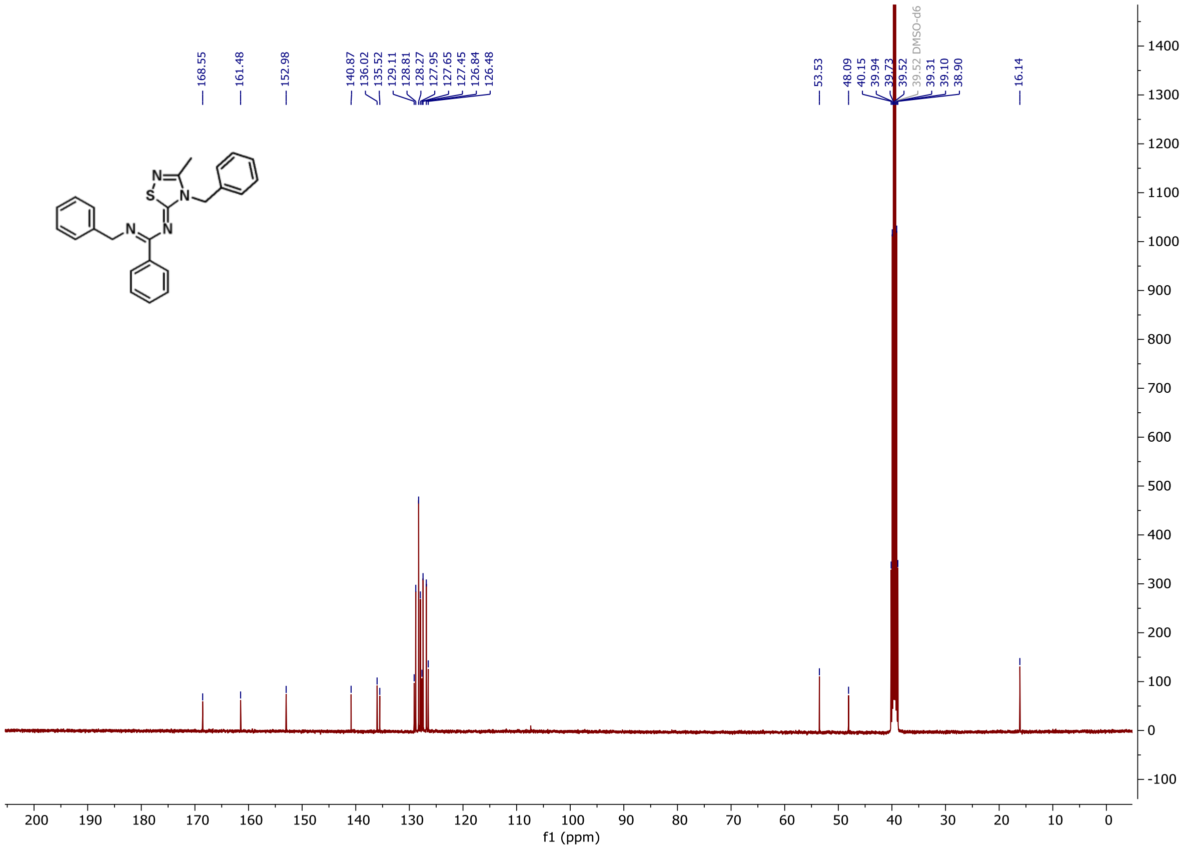

^{1}H-NMR spectrum (400 MHz, DMSO-d_6) of **39**

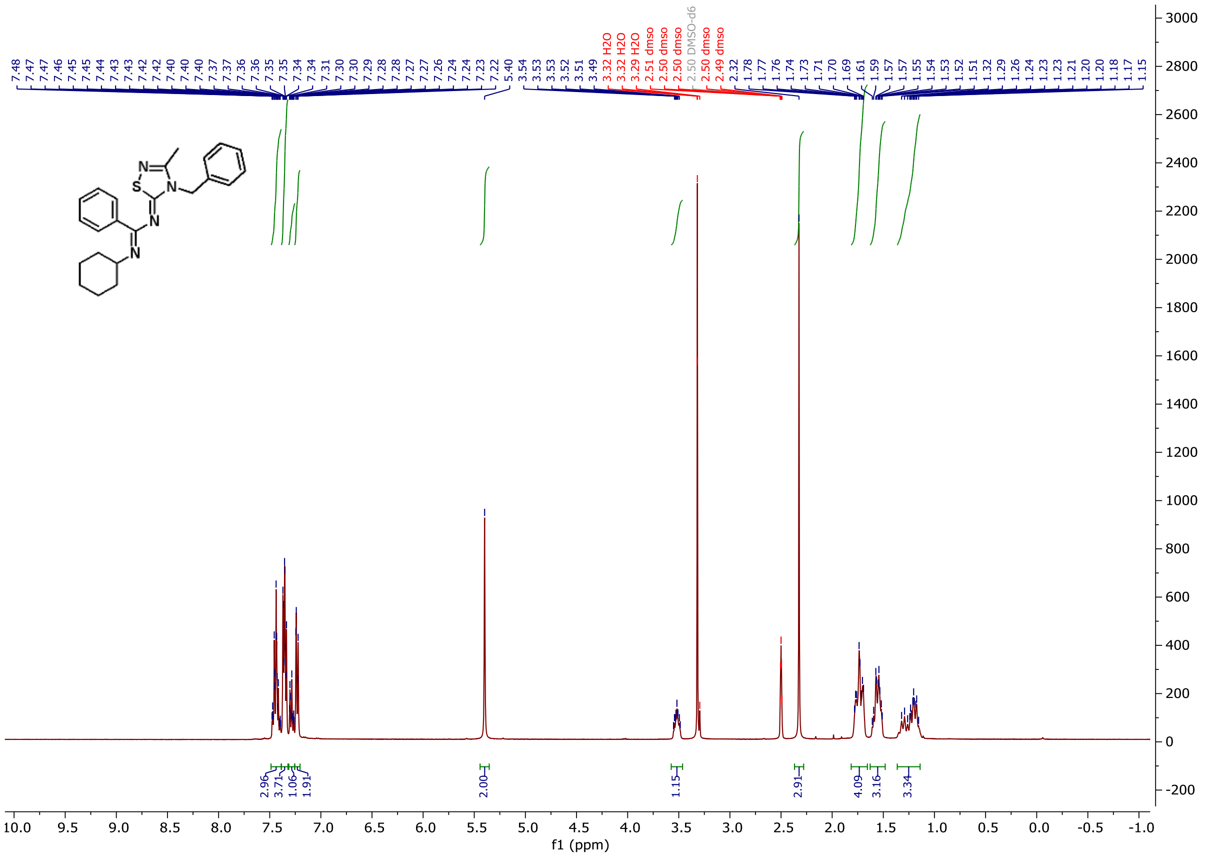

^{13}C-NMR spectrum (101 MHz, DMSO-d_6) of **39**

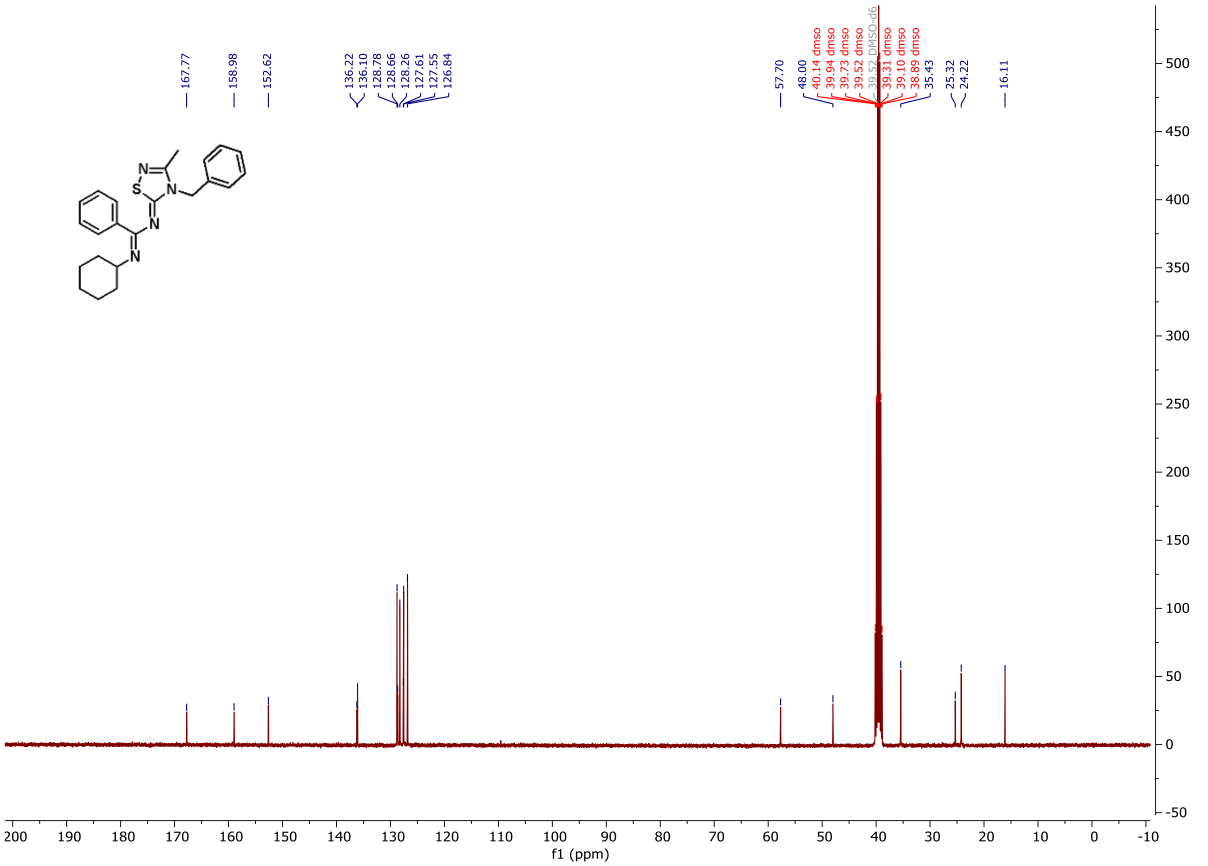

^{1}H-NMR spectrum (400 MHz, DMSO-d_6) of **40**

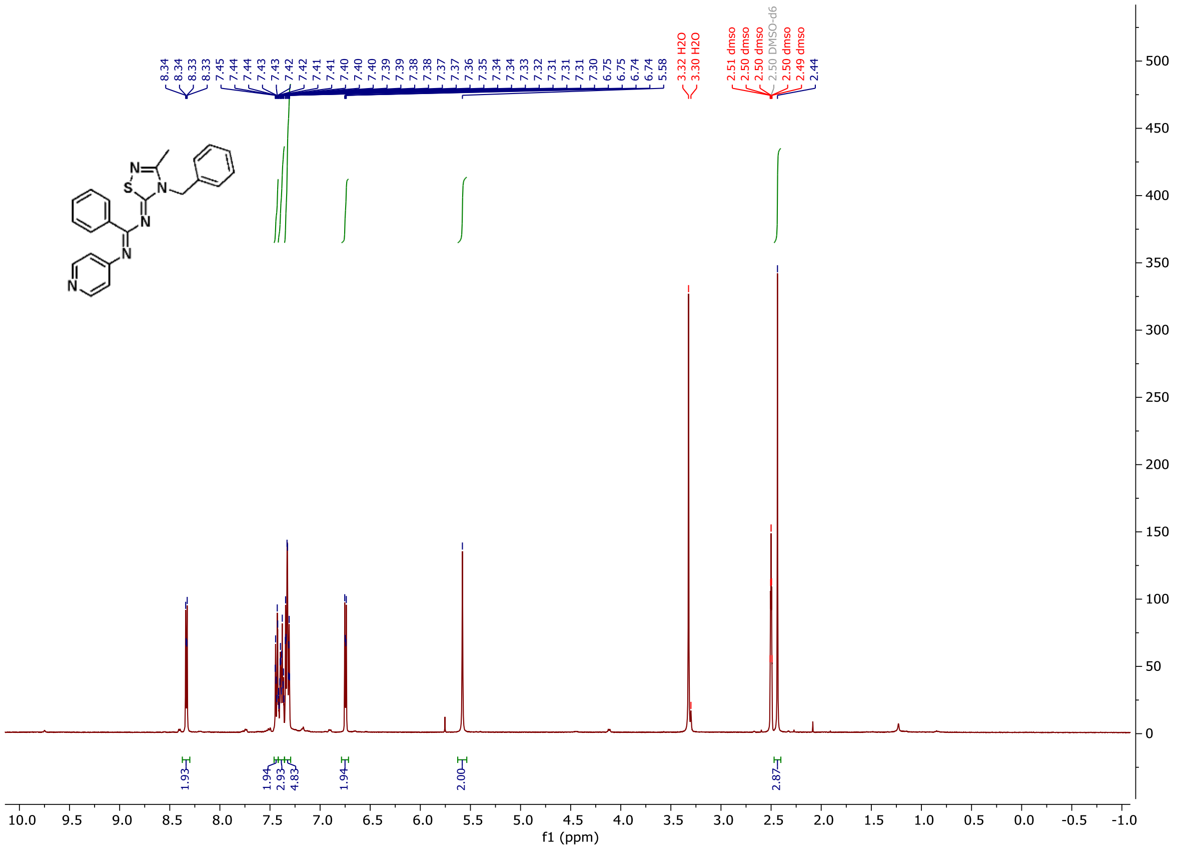

^{13}C-NMR spectrum (101 MHz, DMSO-d_6) of **40**

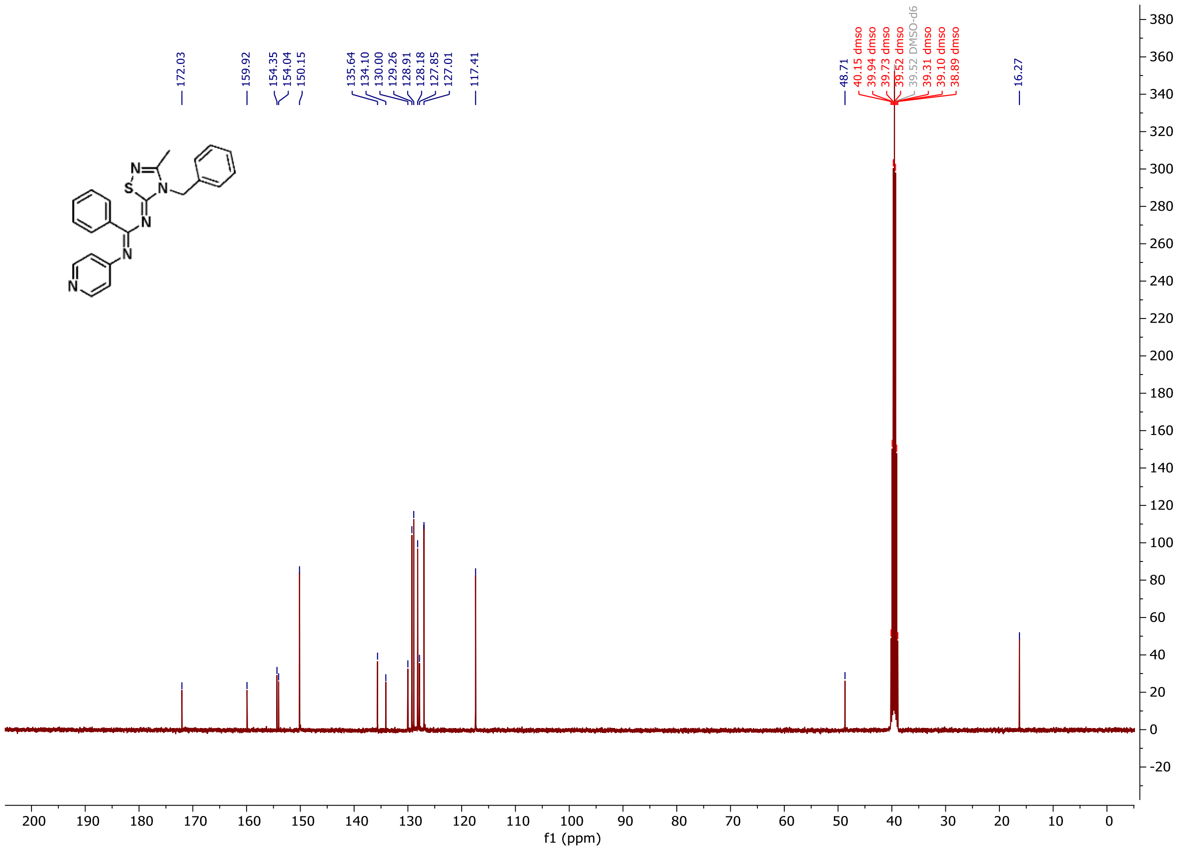

^{1}H-NMR spectrum (400 MHz, DMSO-d_6) of **41**

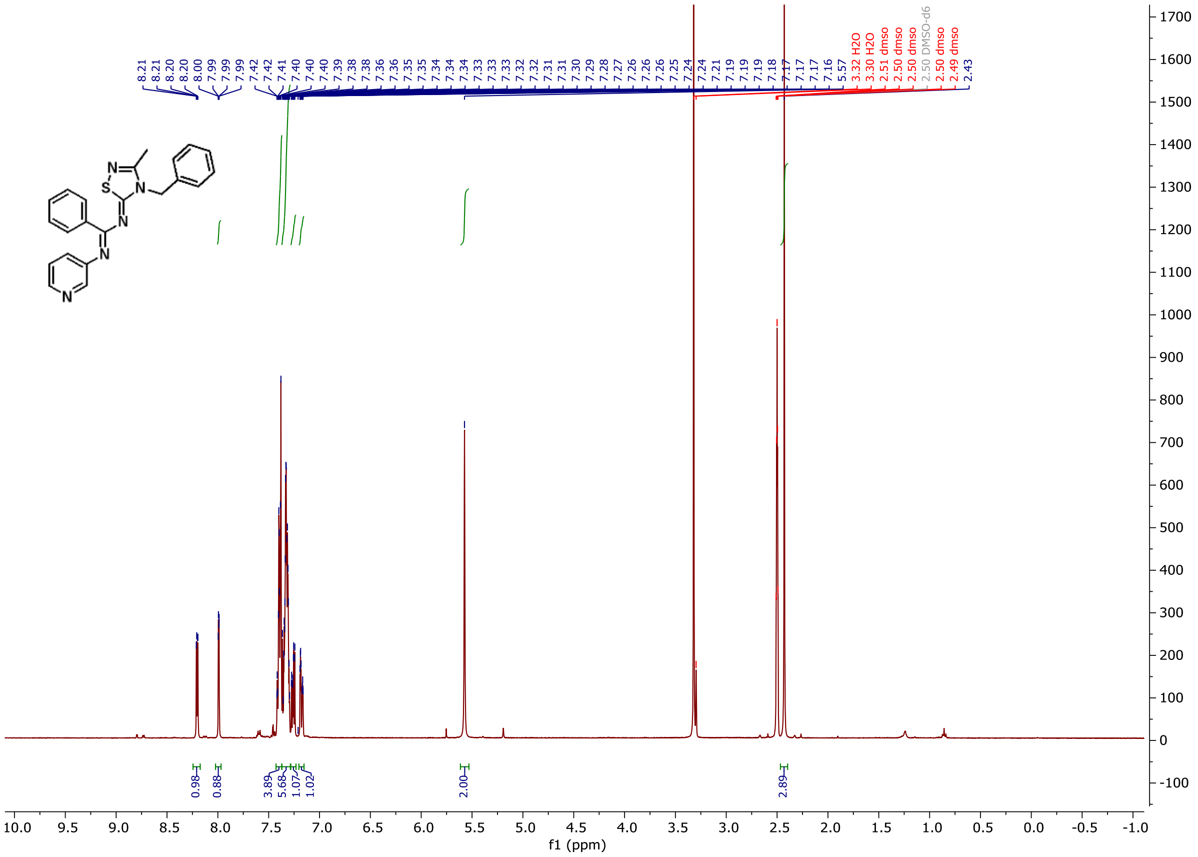

^{13}C-NMR spectrum (101 MHz, DMSO-d_6) of **41**

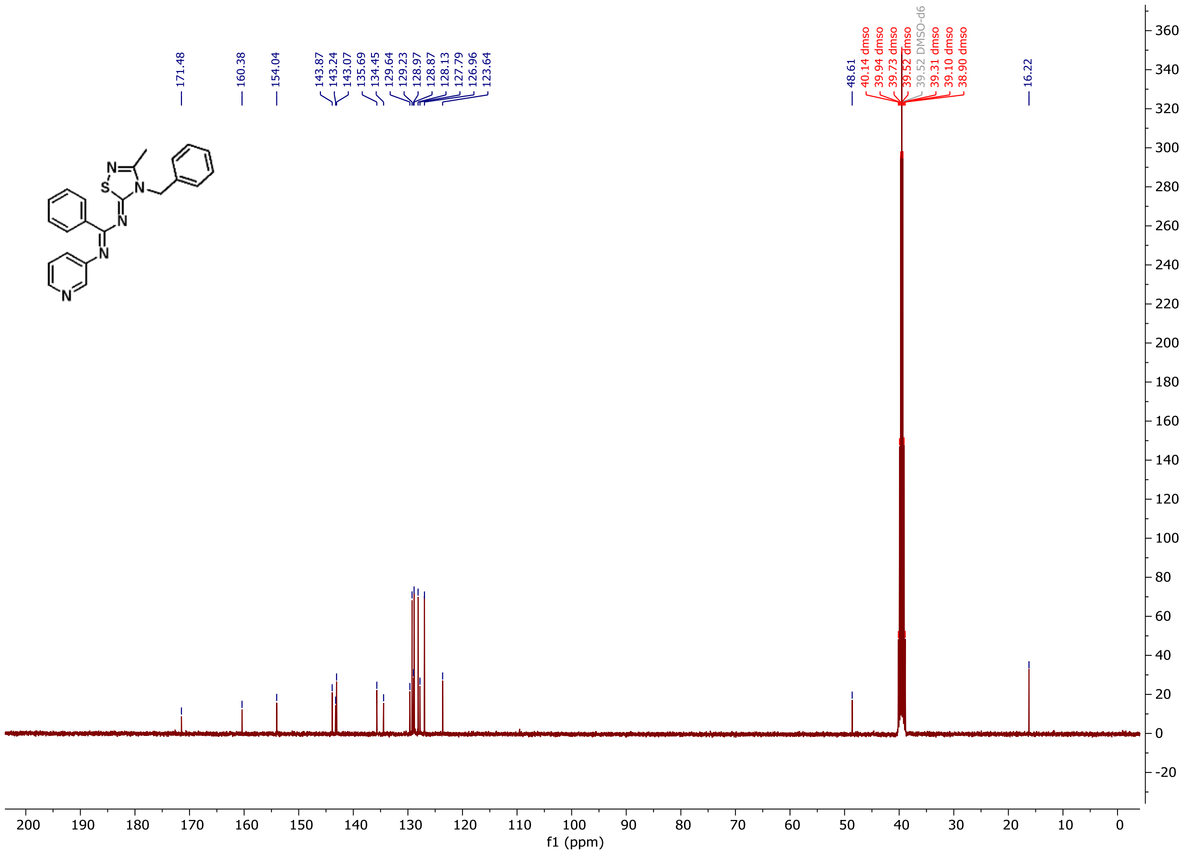

^{1}H-NMR spectrum (400 MHz, DMSO-d_6) of **42**

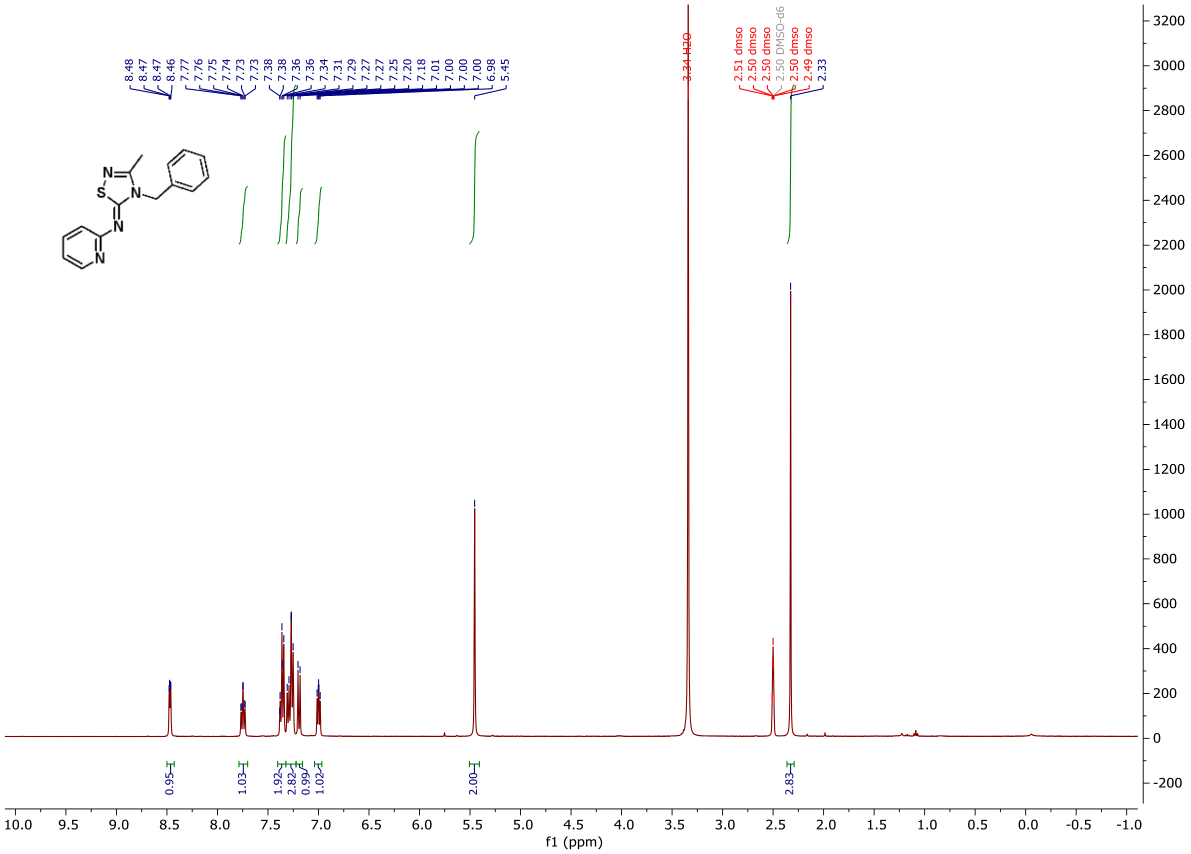

^{13}C-NMR spectrum (101 MHz, DMSO-d_6) of **42**

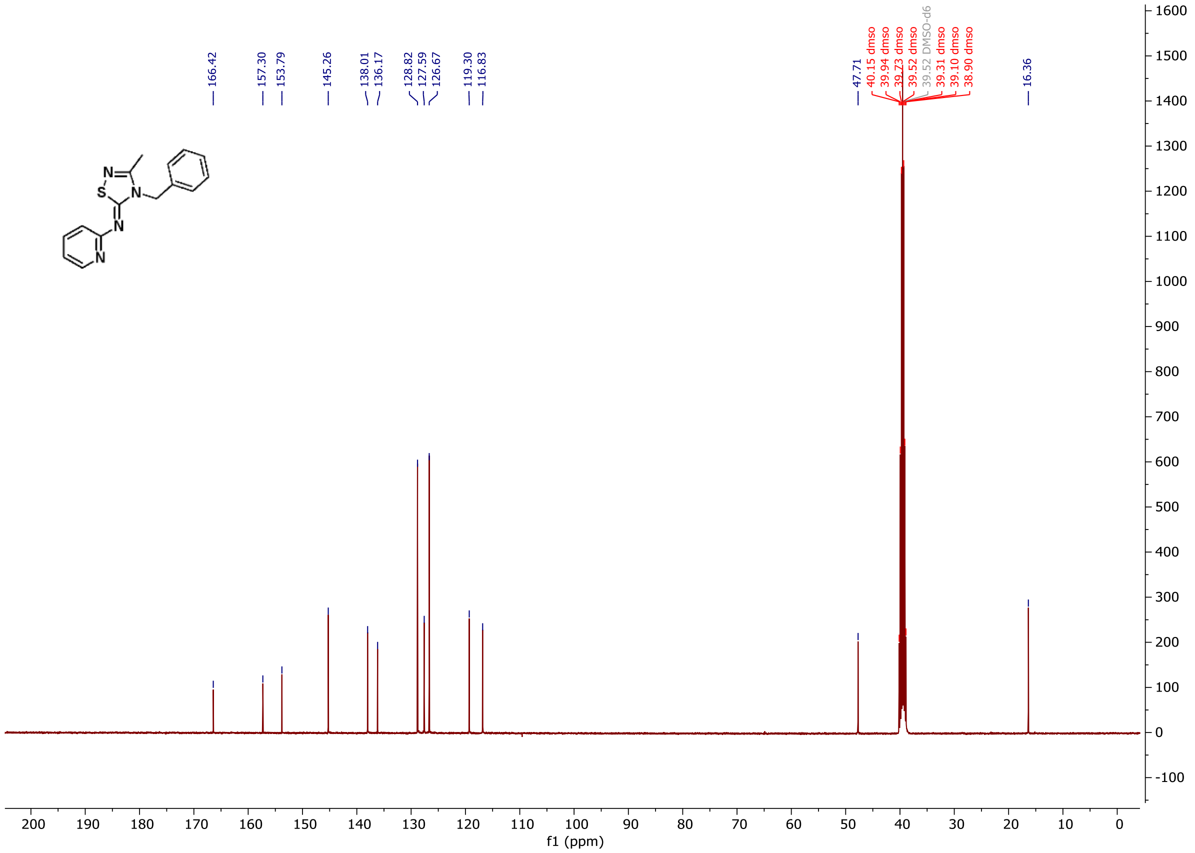

^{1}H-NMR spectrum (400 MHz, DMSO-d_6) of **43**

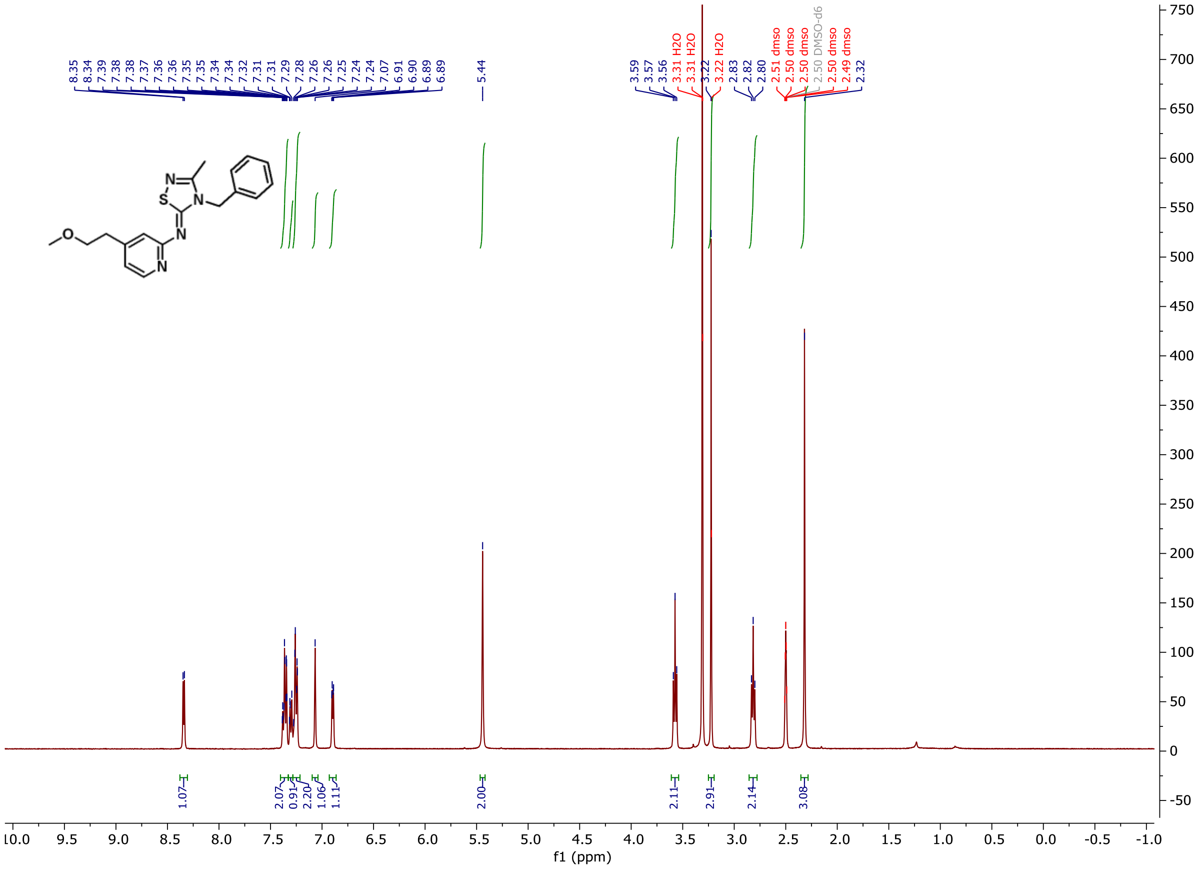

^{13}C-NMR spectrum (101 MHz, DMSO-d_6) of **43**

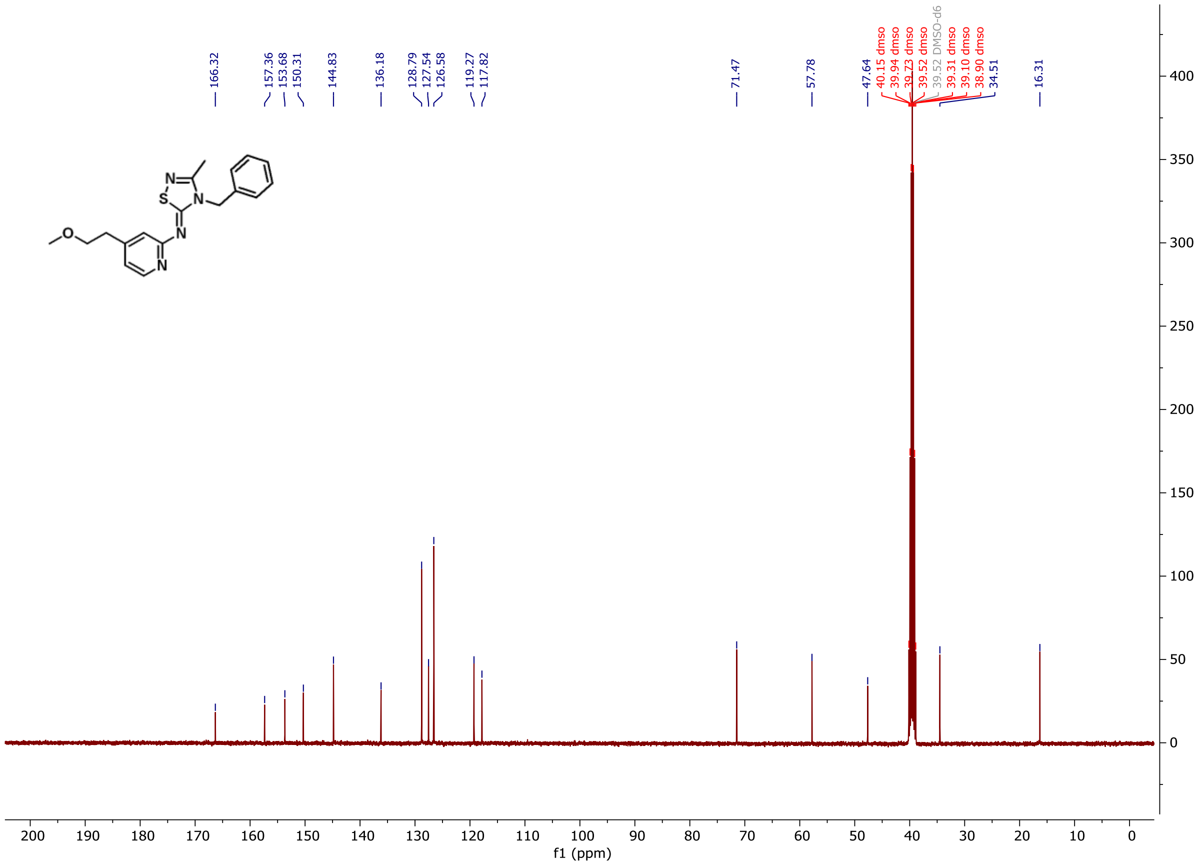

^{1}H-NMR spectrum (400 MHz, DMSO-d_6) of **44**

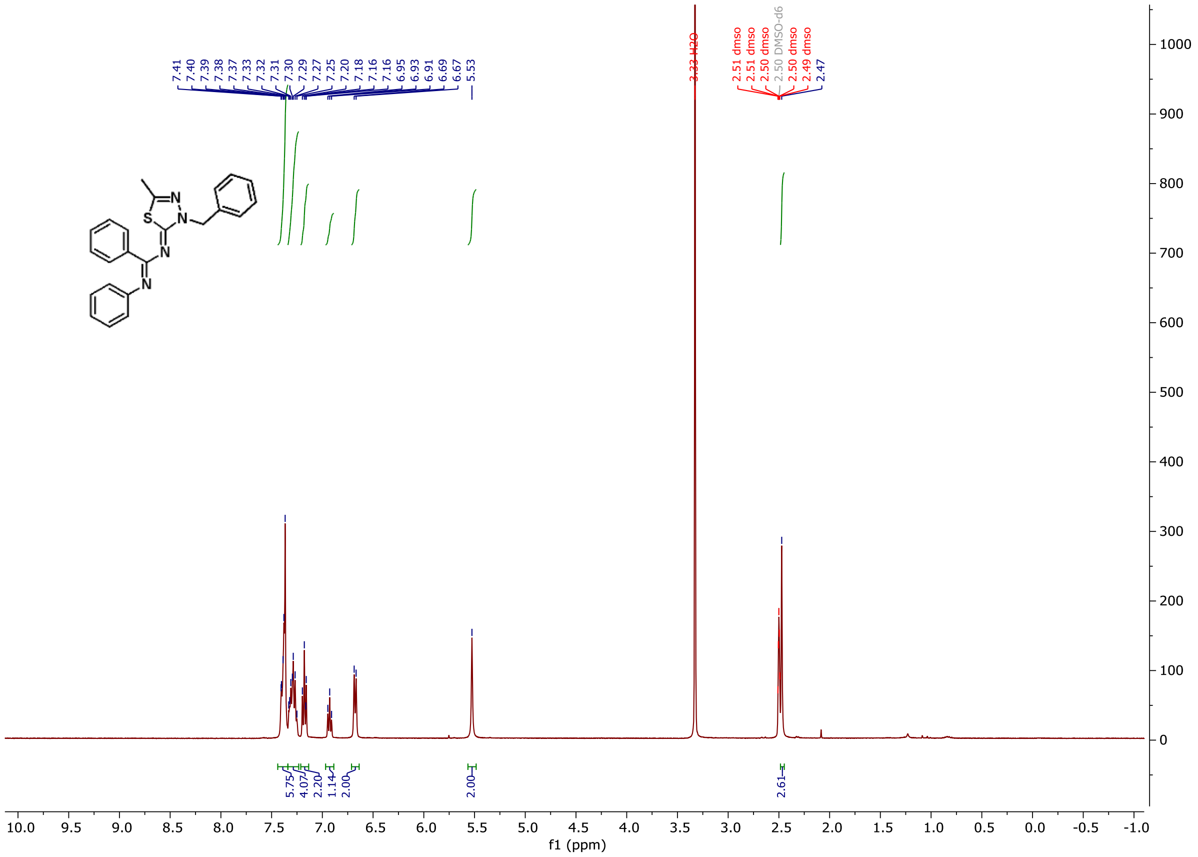

^{13}C-NMR spectrum (101 MHz, DMSO-d_6) of **44**

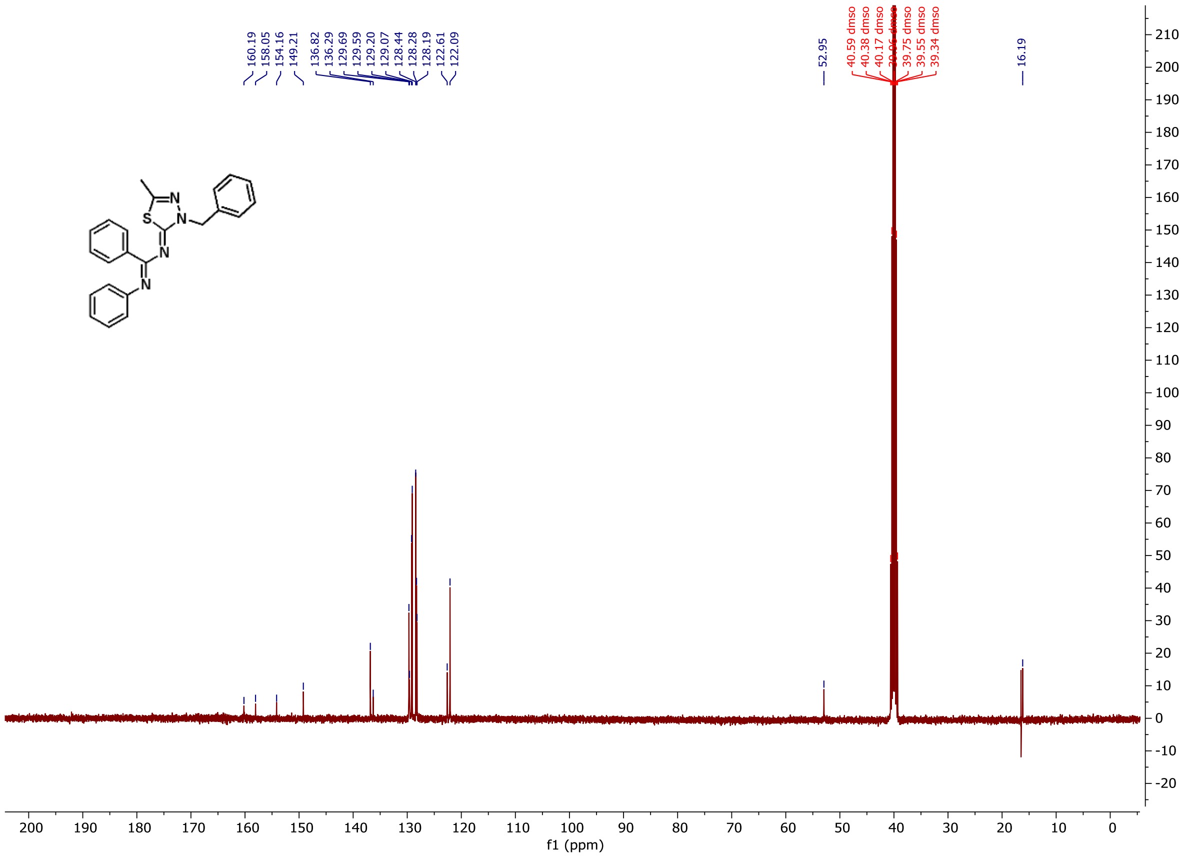

^{1}H-NMR spectrum (400 MHz, DMSO-d_6) of **45**

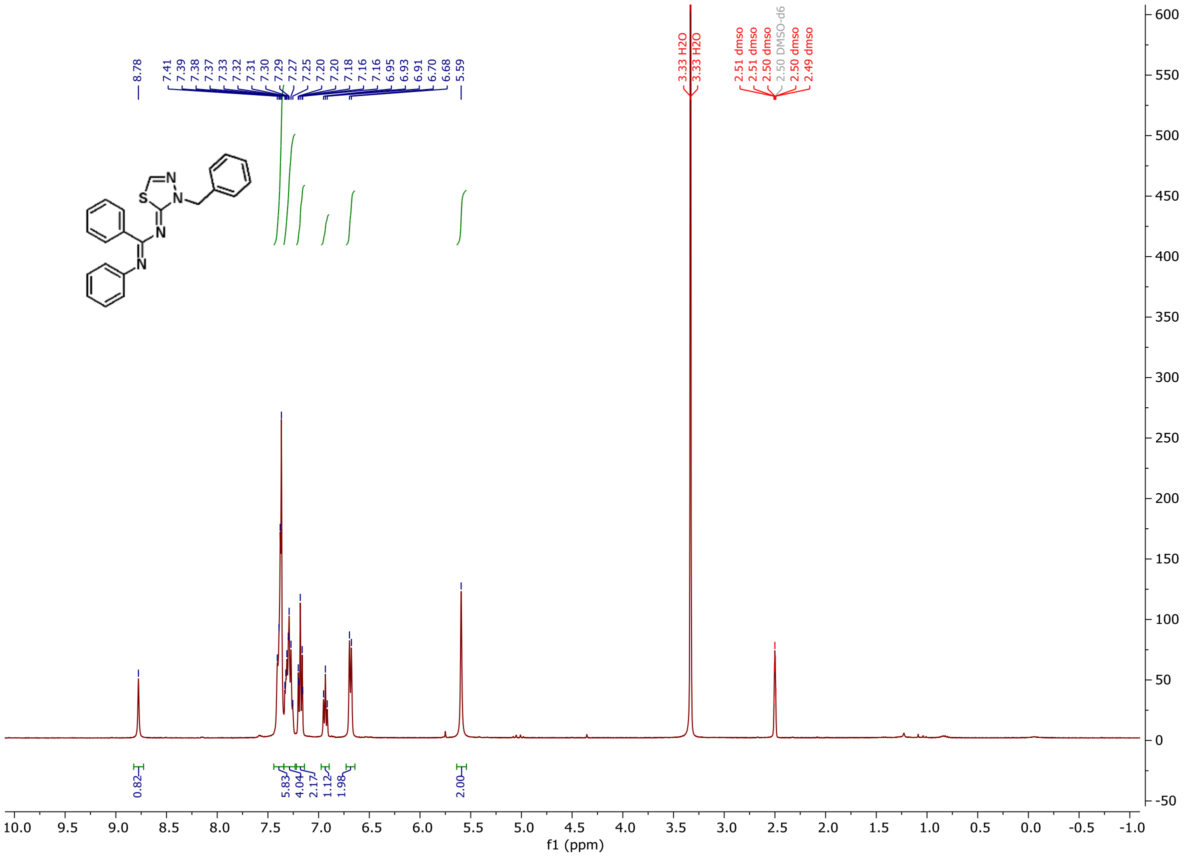

^{13}C-NMR spectrum (101 MHz, DMSO-d_6) of **45**

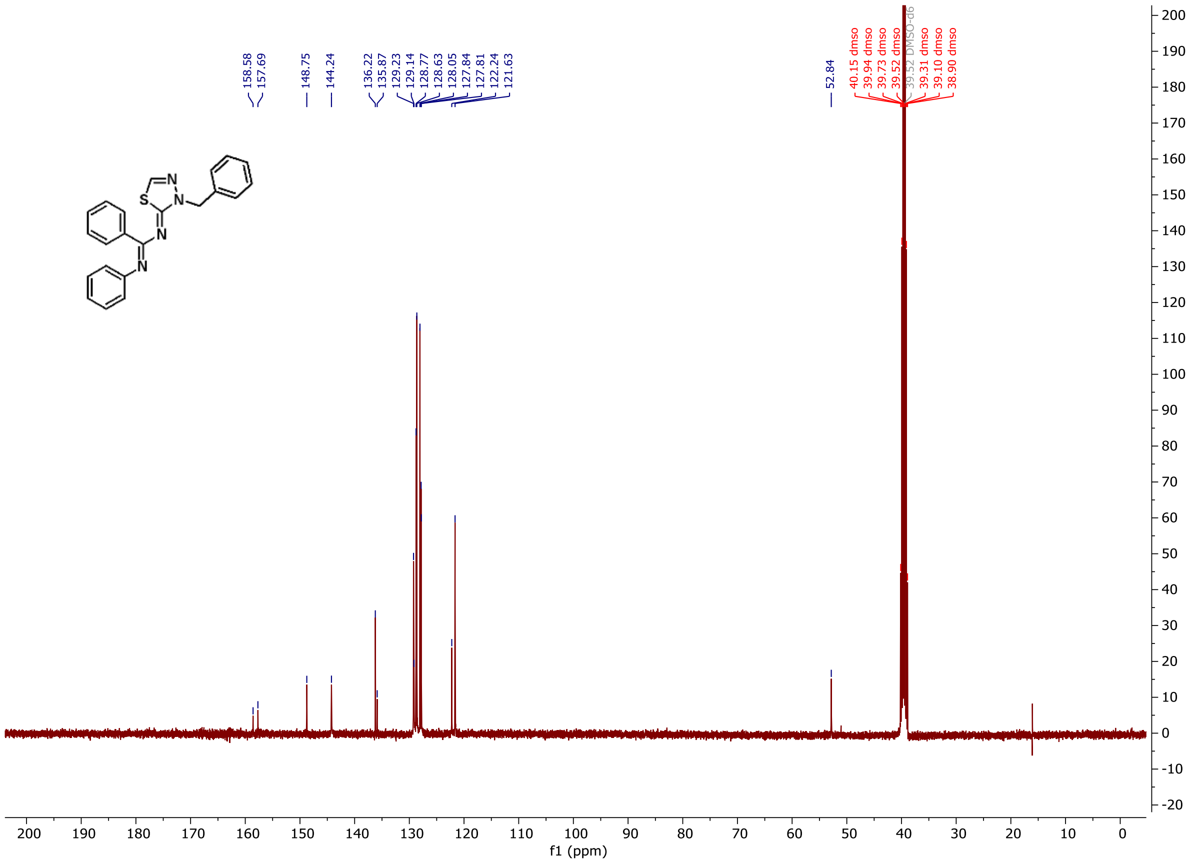

^{1}H-NMR spectrum (400 MHz, DMSO-d_6) of **46**

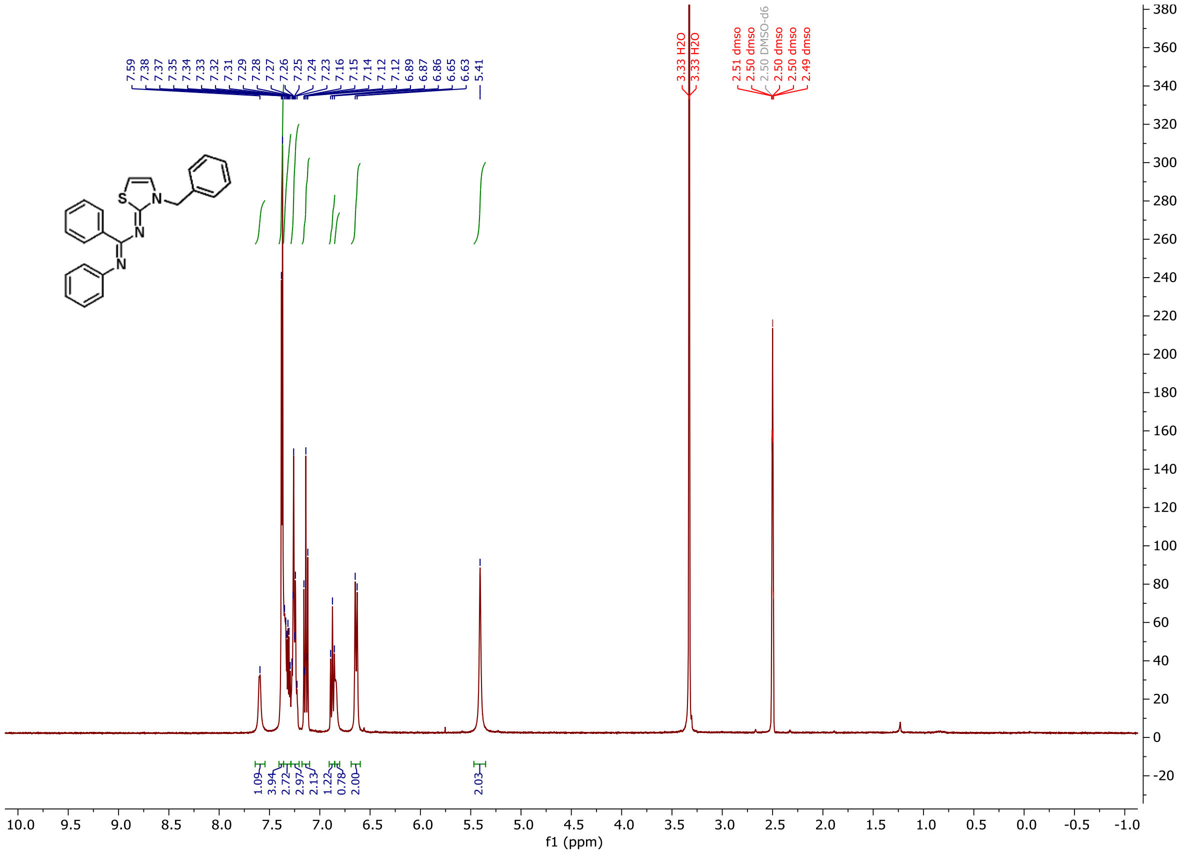

^{13}C-NMR spectrum (101 MHz, DMSO-d_6) of **46**

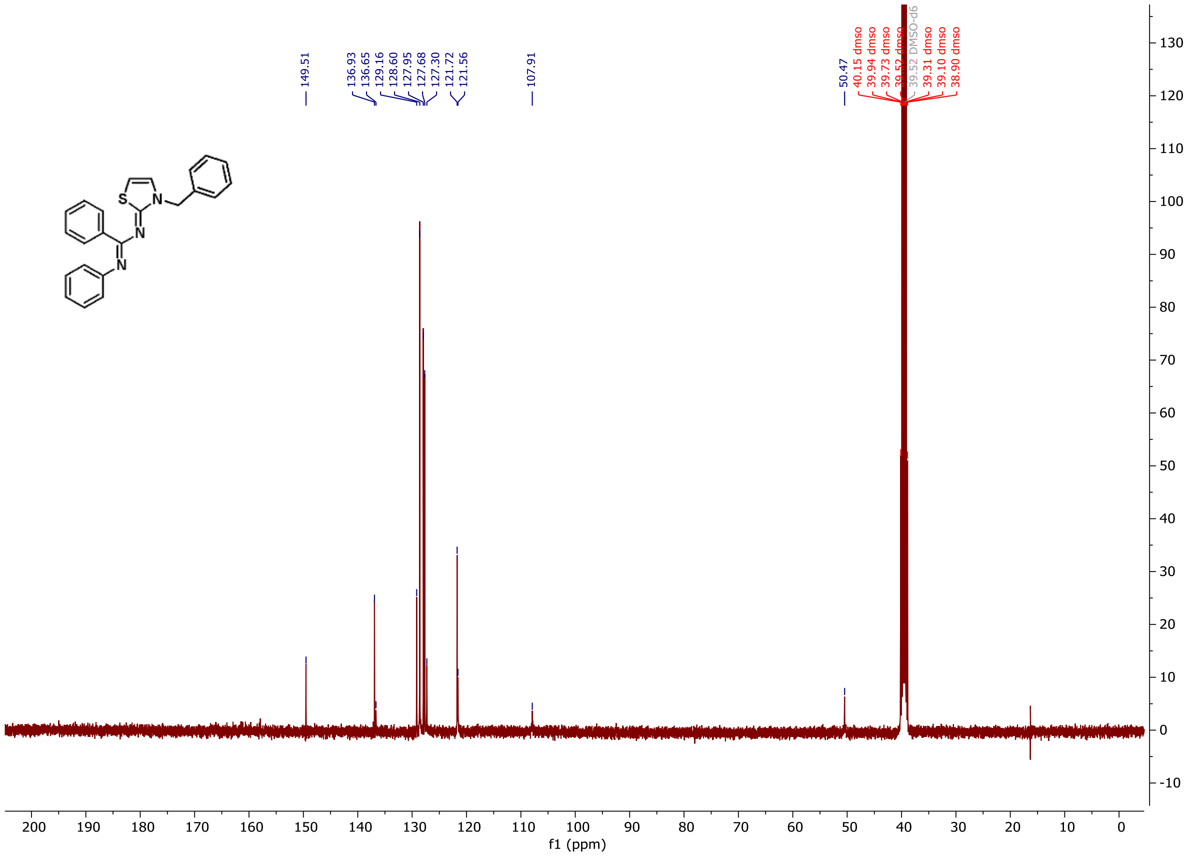

HPLC-MS analysis of selected analogues 6, 9-11, 14, 16, 17, 19, 21-25, 27-29 and 34

HPLC-MS analysis of **6**

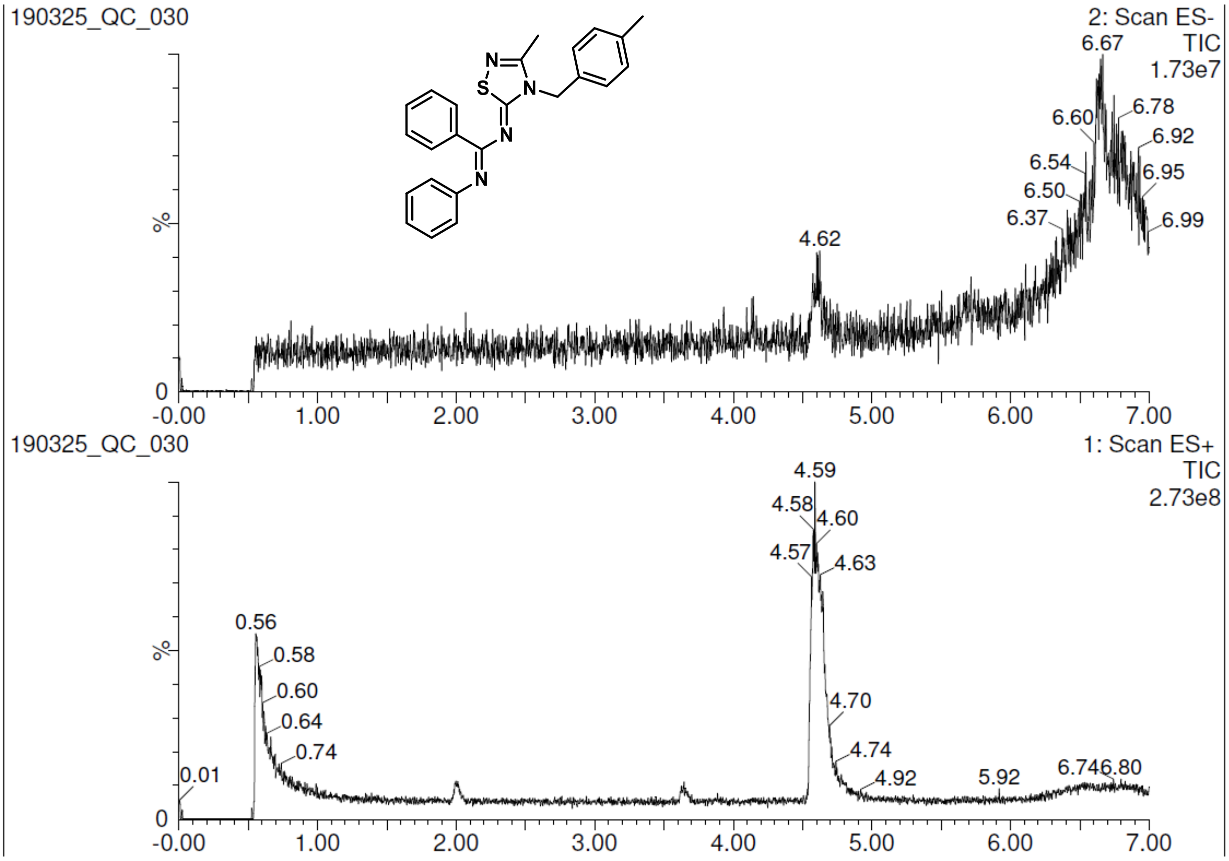

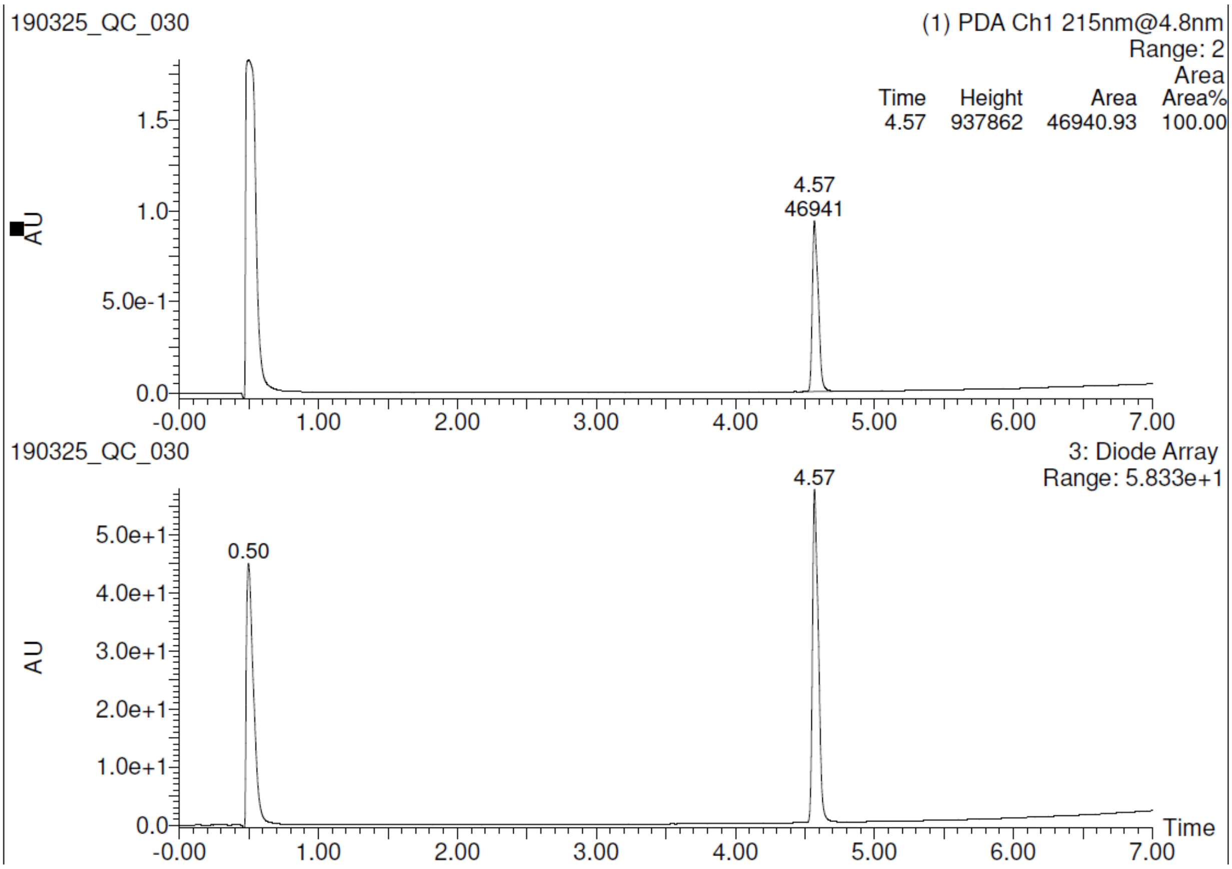

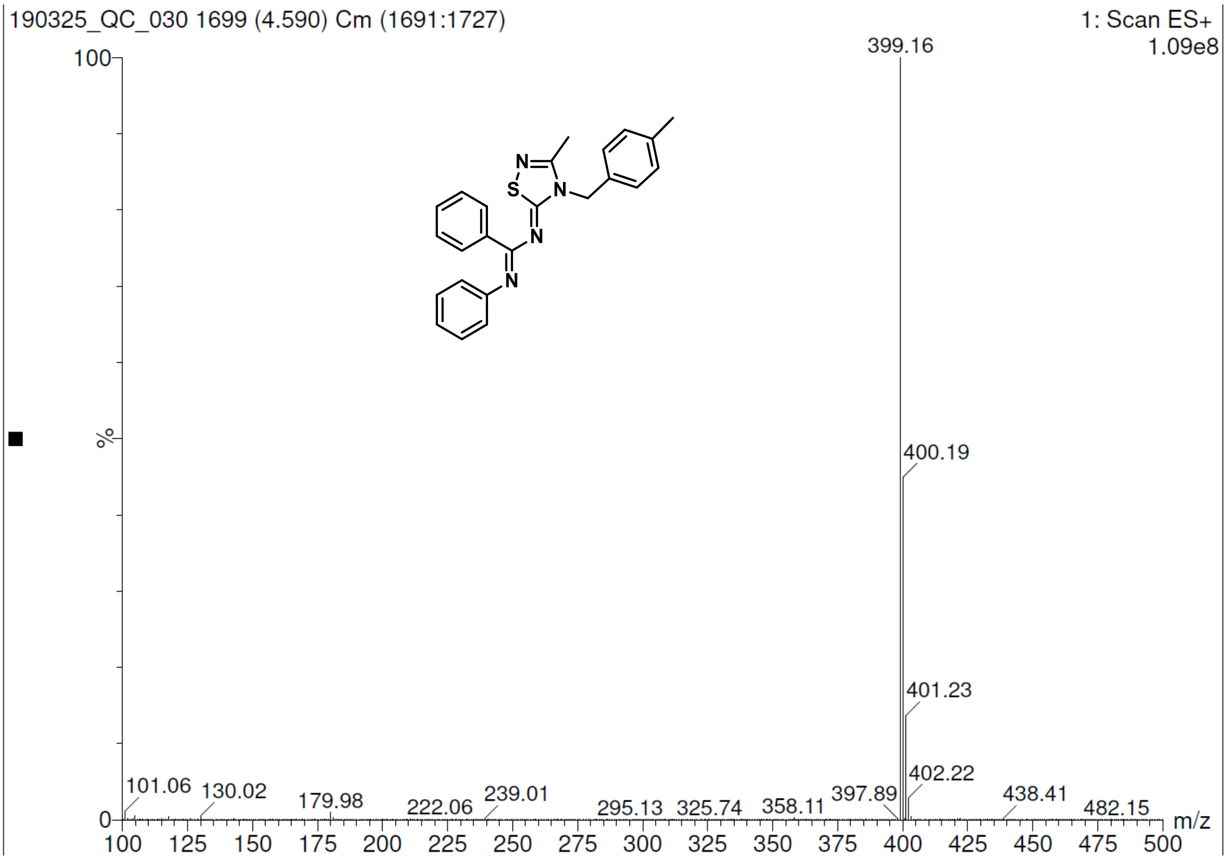
190325_QC_030 1699 (4.590) Cm (1691:1727)
1: Scan ES+
1.09e8
100
%
0
399.16
400.19
401.23
402.22
101.06
130.02
179.98
222.06
239.01
295.13
325.74
358.11
397.89
438.41
482.15
m/z
100 125 150 175 200 225 250 275 300 325 350 375 400 425 450 475 500
N
S
N
N
N

HPLC-MS analysis of **9**

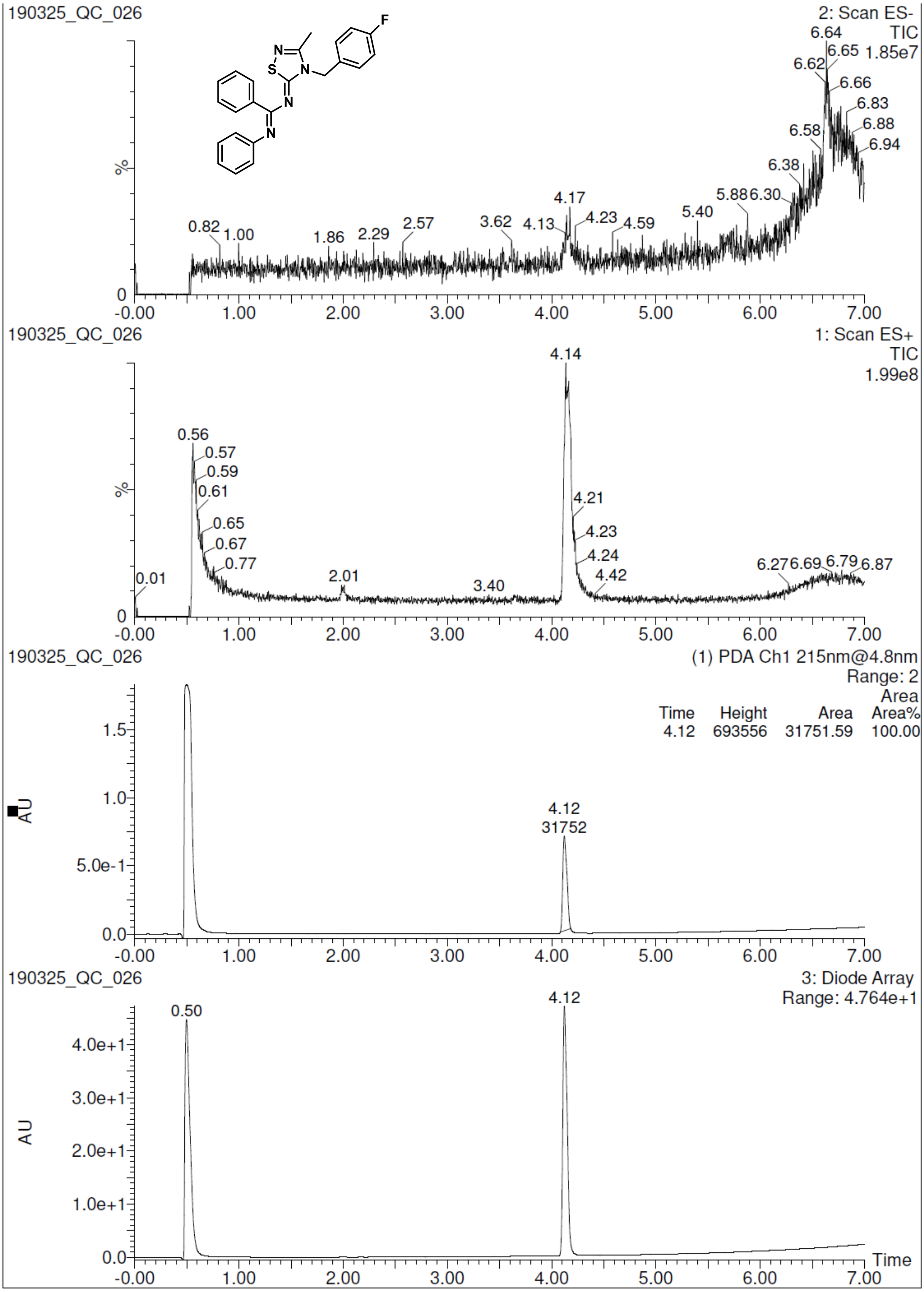

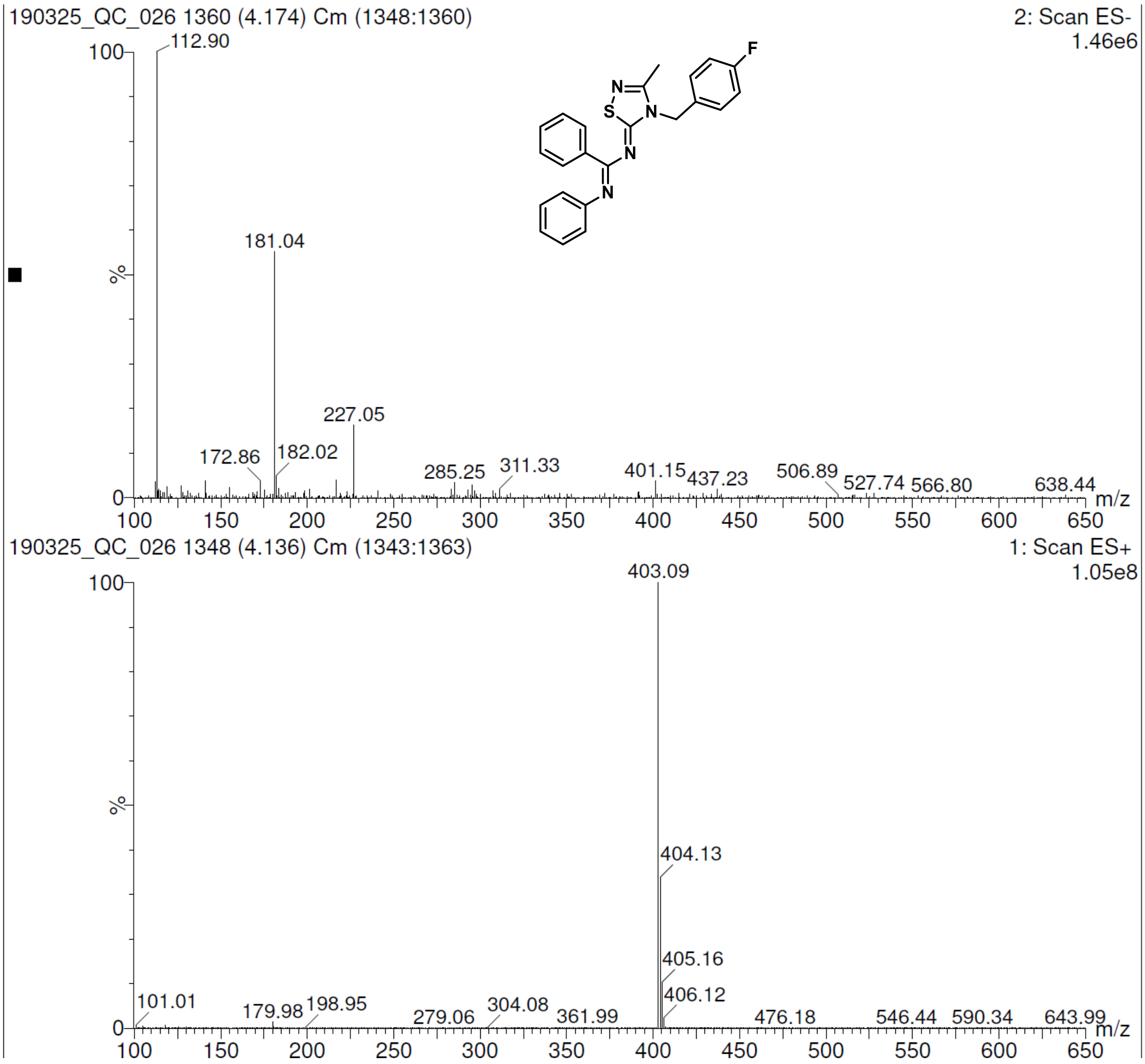
190325_QC_026 1360 (4.174) Cm (1348:1360)
2: Scan ES-
1.46e6
100
112.90
181.04
%
227.05
172.86
182.02
285.25
311.33
401.15
437.23
506.89
527.74
566.80
638.44
0
100 150 200 250 300 350 400 450 500 550 600 650
m/z
F
N
S
N
N
N
190325_QC_026 1348 (4.136) Cm (1343:1363)
1: Scan ES+
1.05e8
403.09
100
%
404.13
405.16
406.12
101.01
179.98
198.95
279.06
304.08
361.99
476.18
546.44
590.34
643.99
0
100 150 200 250 300 350 400 450 500 550 600 650
m/z

HPLC-MS analysis of **10**

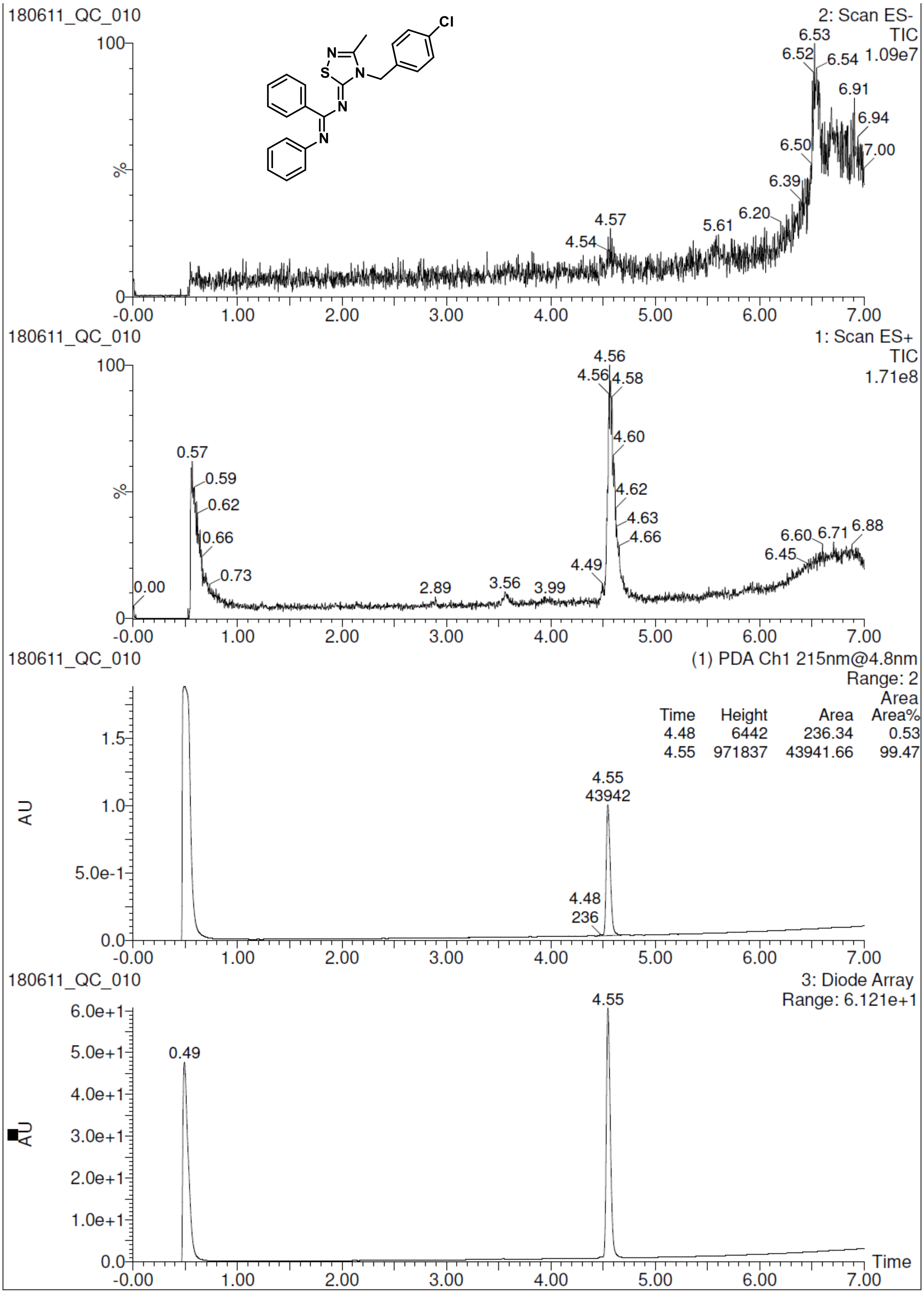

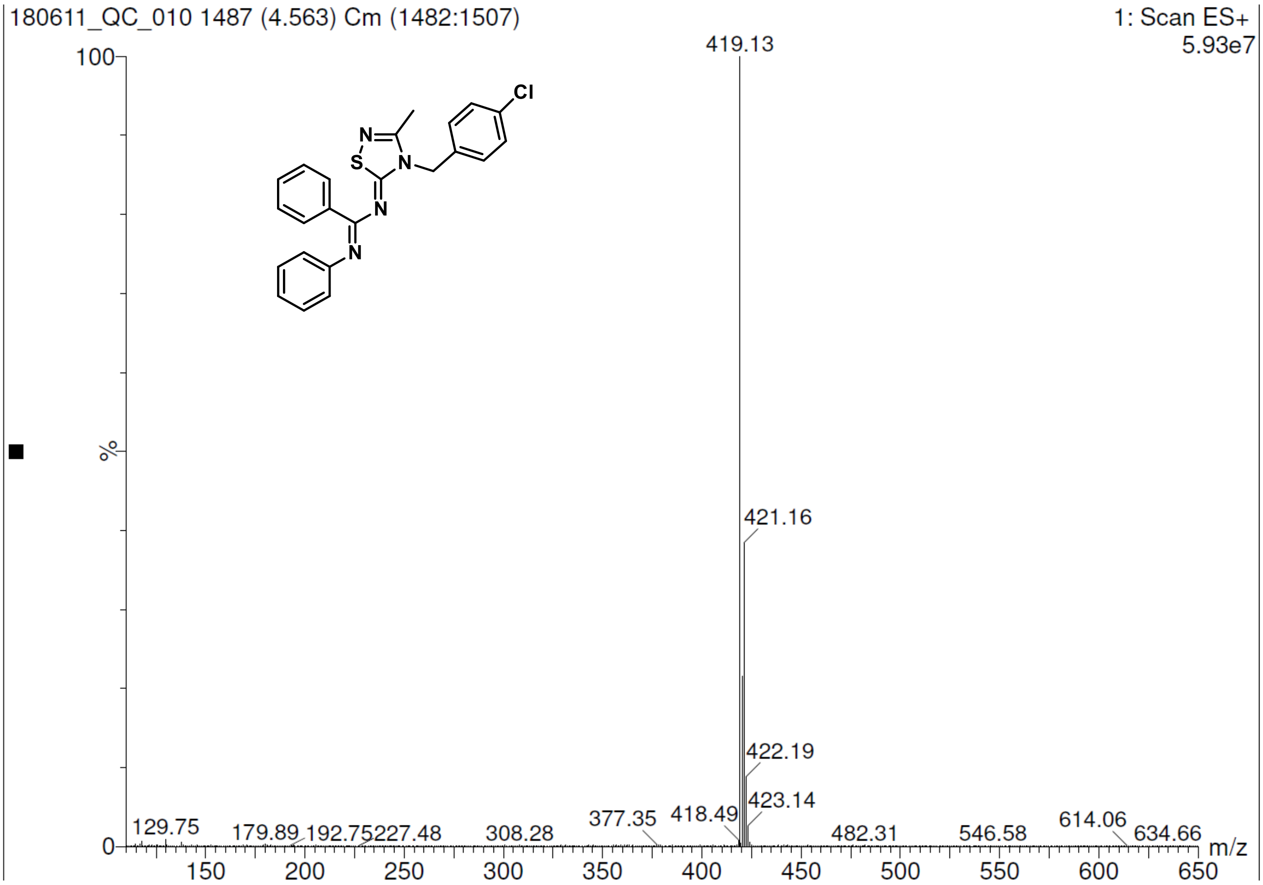
180611_QC_010 1487 (4.563) Cm (1482:1507)
1: Scan ES+
5.93e7
419.13
421.16
422.19
423.14
418.49
377.35
129.75
179.89
192.75
227.48
308.28
482.31
546.58
614.06
634.66
100
%
0
150
200
250
300
350
400
450
500
550
600
650
m/z
Cl
N
S
N
N
N

HPLC-MS analysis of **11**

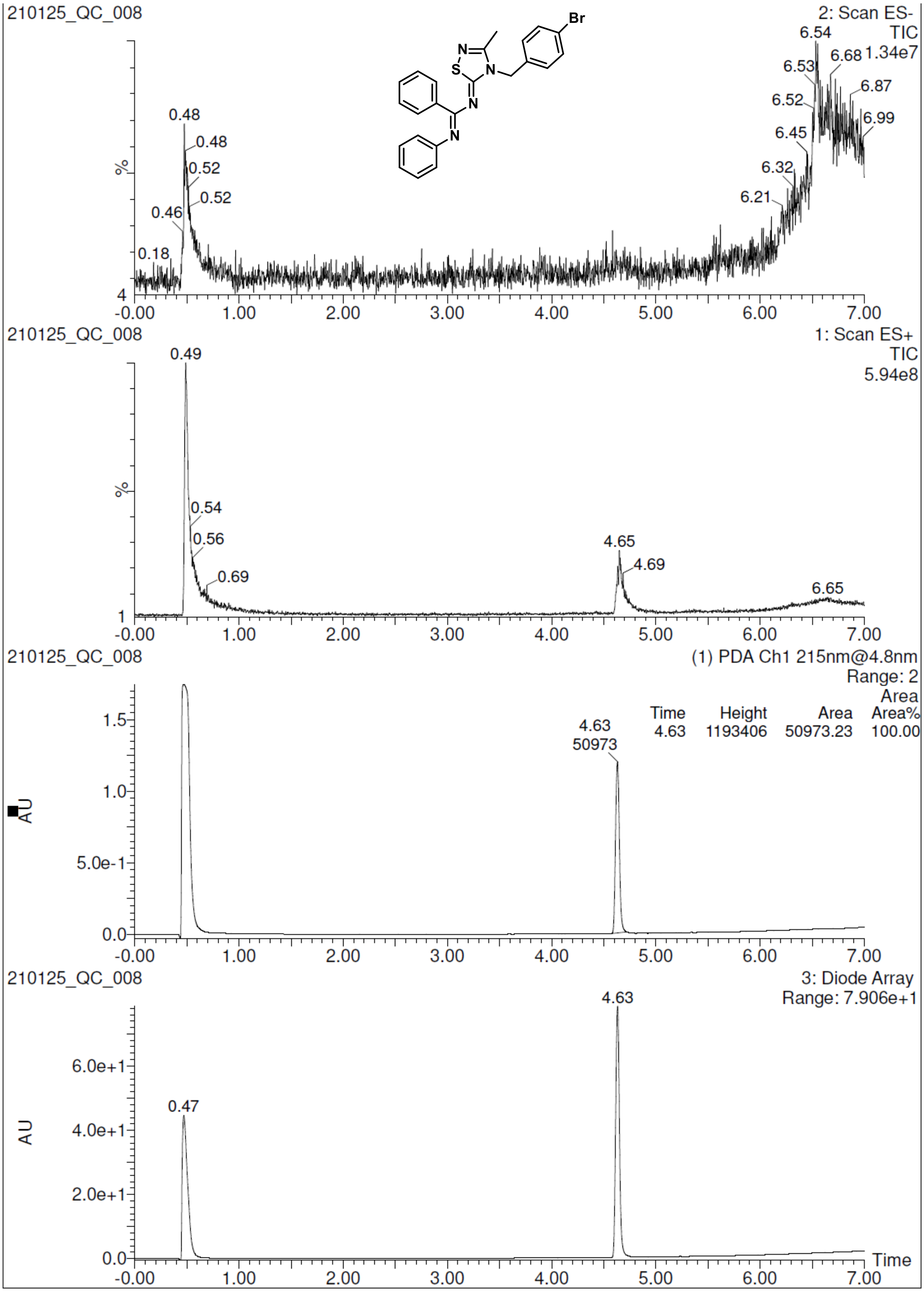

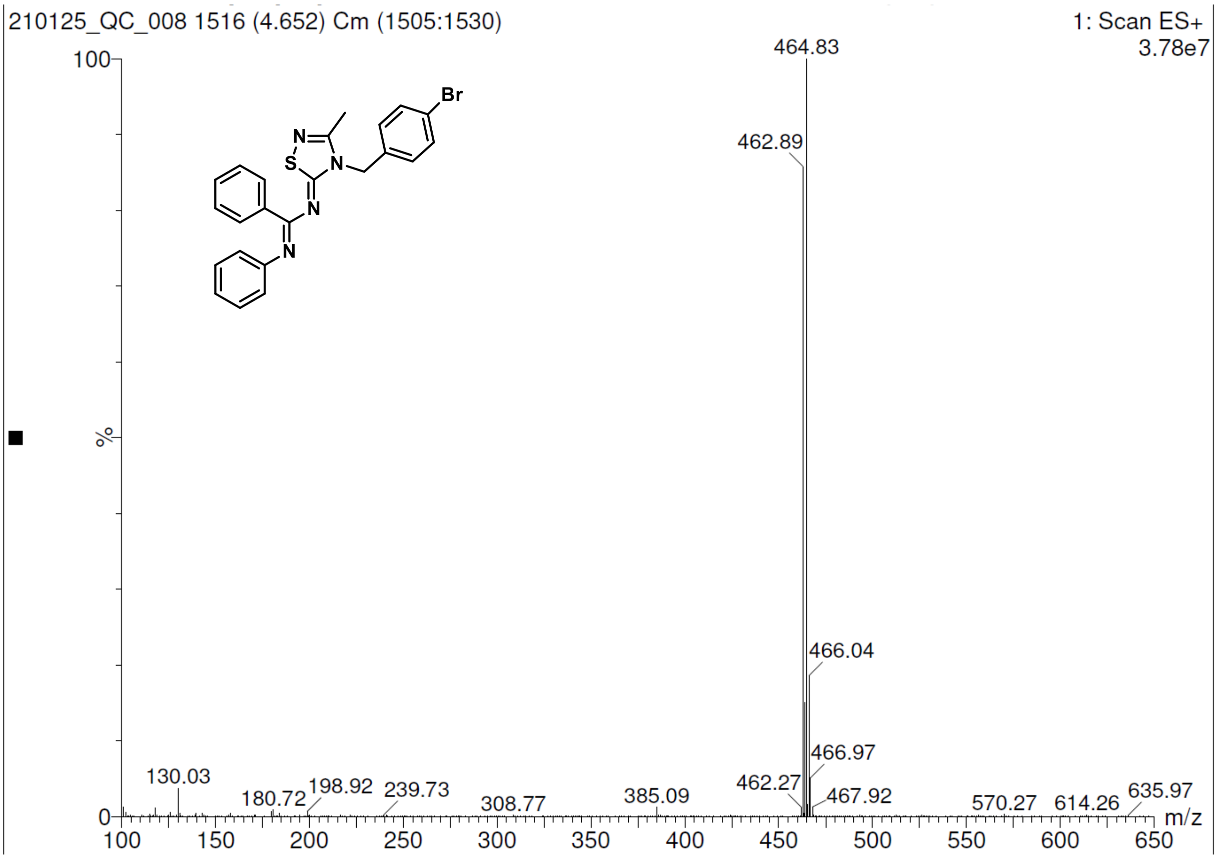

210125_QC_008 1516 (4.652) Cm (1505:1530)
1: Scan ES+
3.78e7
464.83
462.89
466.04
466.97
462.27
467.92
130.03
180.72
198.92
239.73
308.77
385.09
570.27
614.26
635.97
%
m/z
Br
N
S
N
N
N

HPLC-MS analysis of **14**

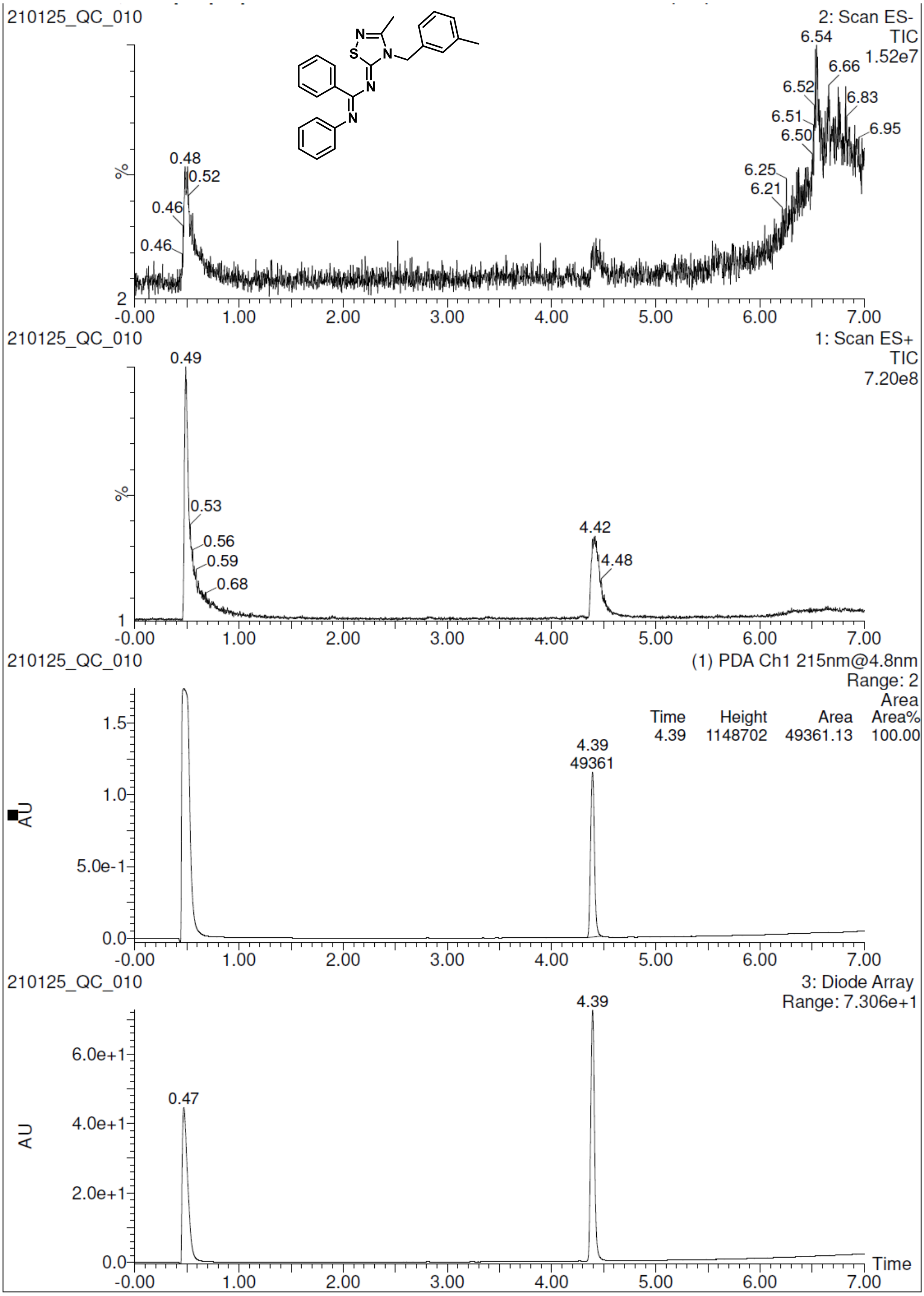

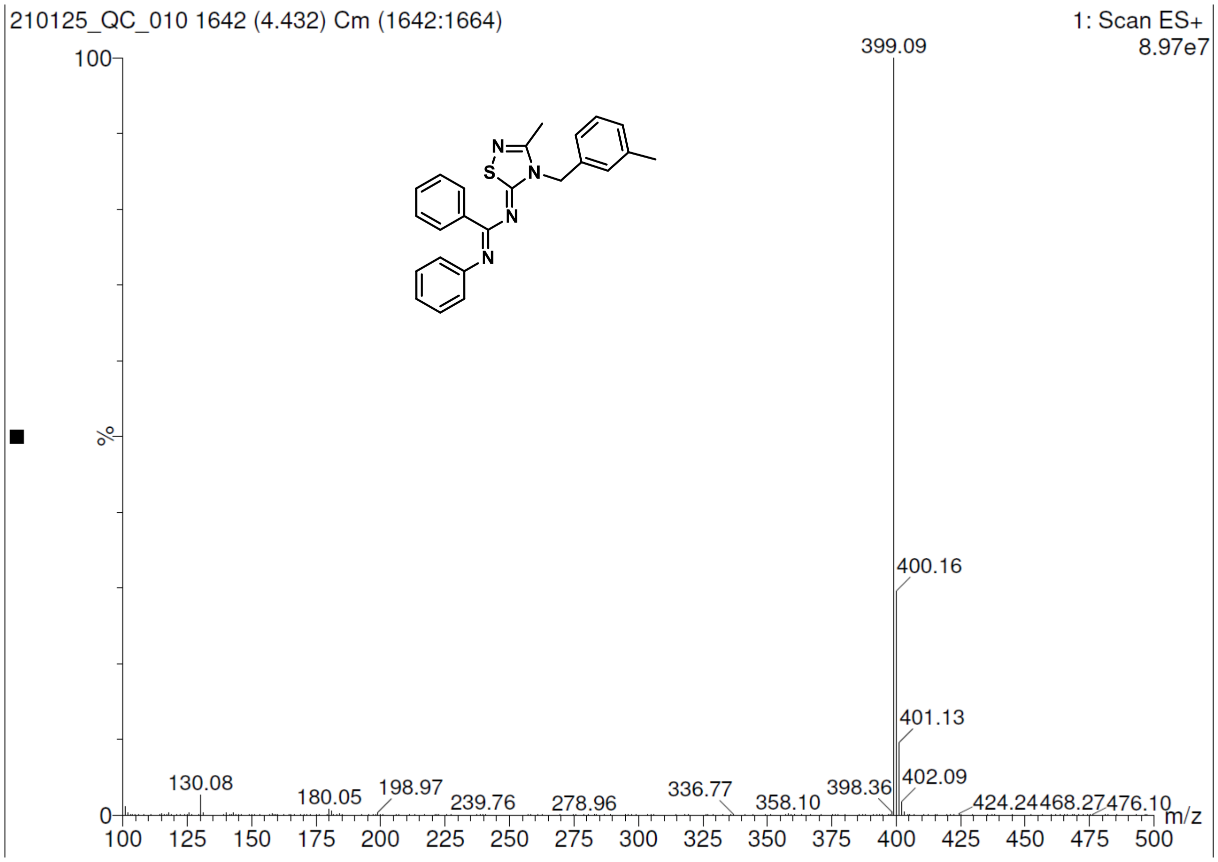
210125_QC_010 1642 (4.432) Cm (1642:1664)
1: Scan ES+
8.97e7
399.09
400.16
401.13
402.09
130.08
180.05
198.97
239.76
278.96
336.77
358.10
398.36
424.24
468.27
476.10
100
0
%
m/z
N
S
N
N
N

HPLC-MS analysis of **16**

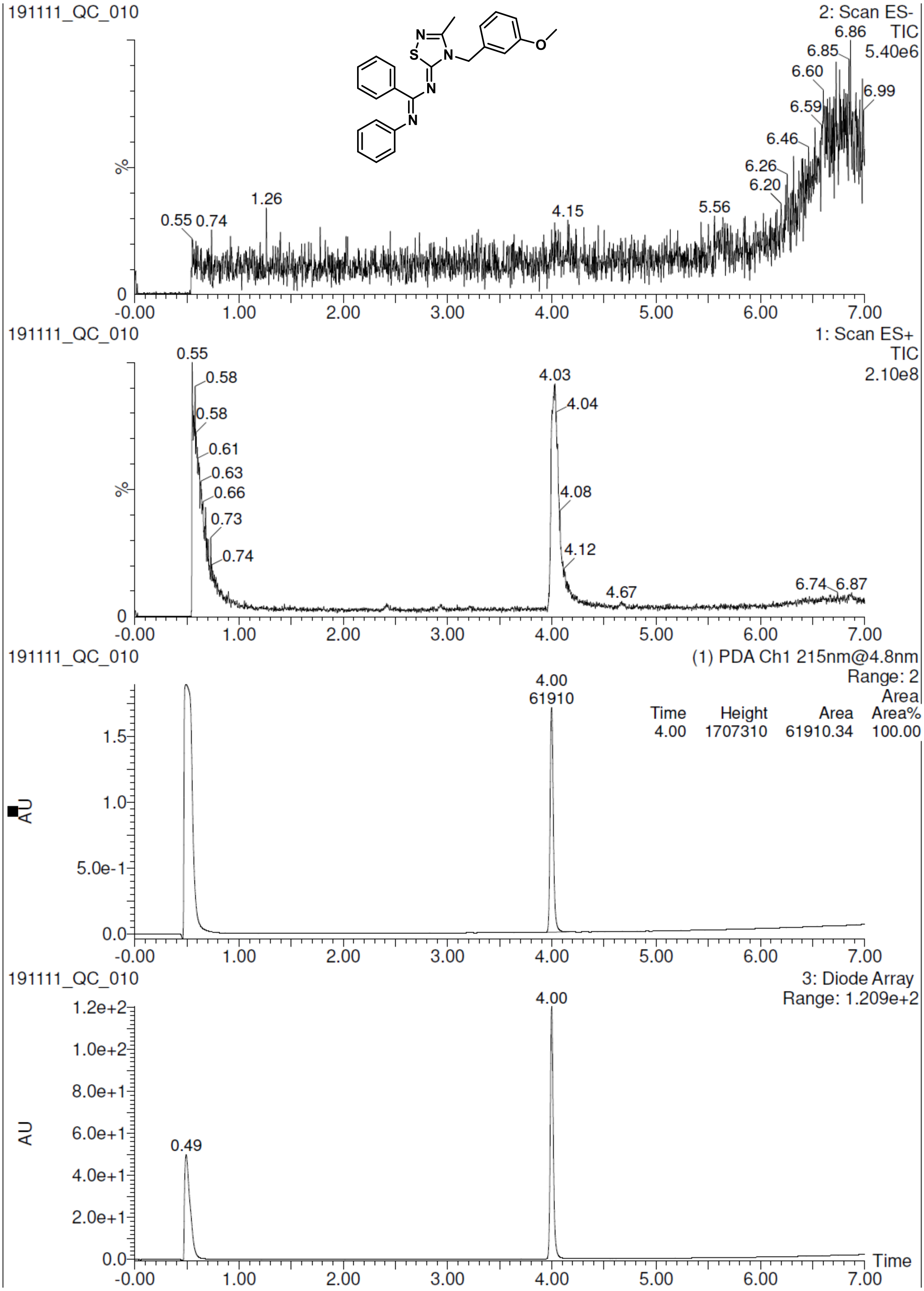

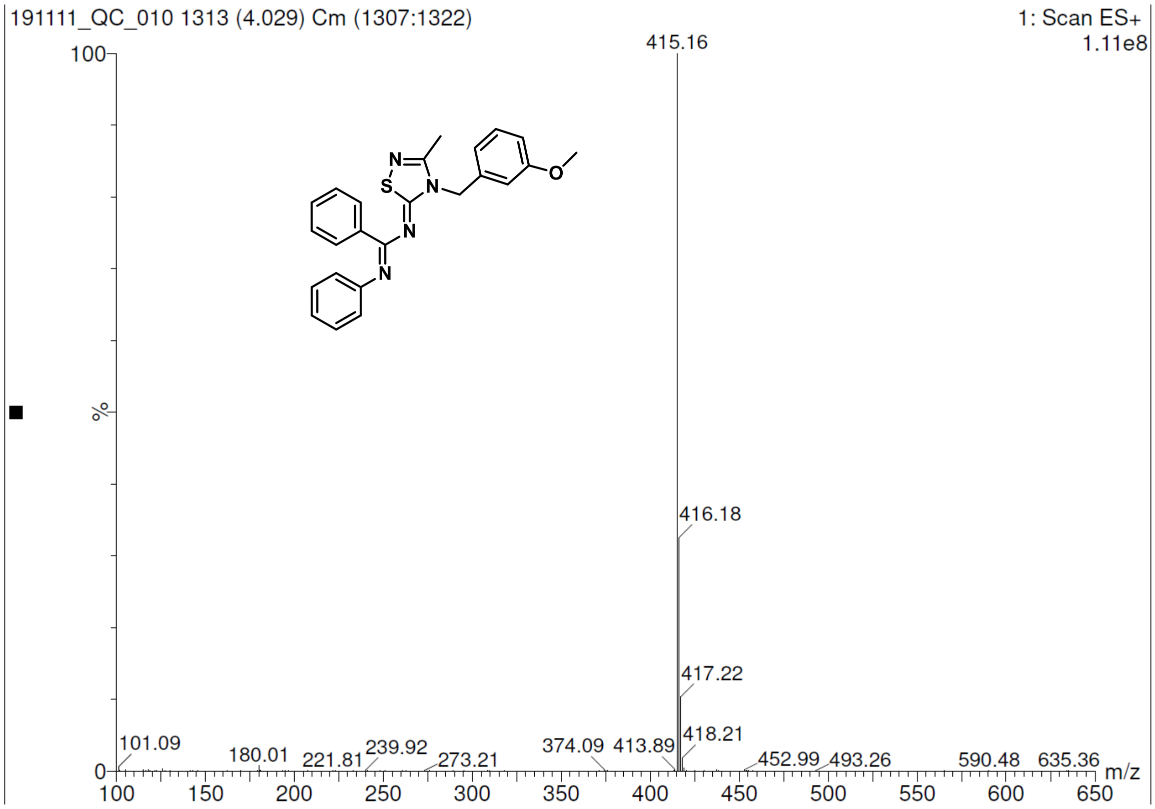

191111_QC_010 1313 (4.029) Cm (1307:1322)
1: Scan ES+
1.11e8
100
%
0
415.16
416.18
417.22
418.21
101.09
180.01
221.81
239.92
273.21
374.09
413.89
452.99
493.26
590.48
635.36
m/z
100
150
200
250
300
350
400
450
500
550
600
650
N
S
N
O
N
N

HPLC-MS analysis of **17**

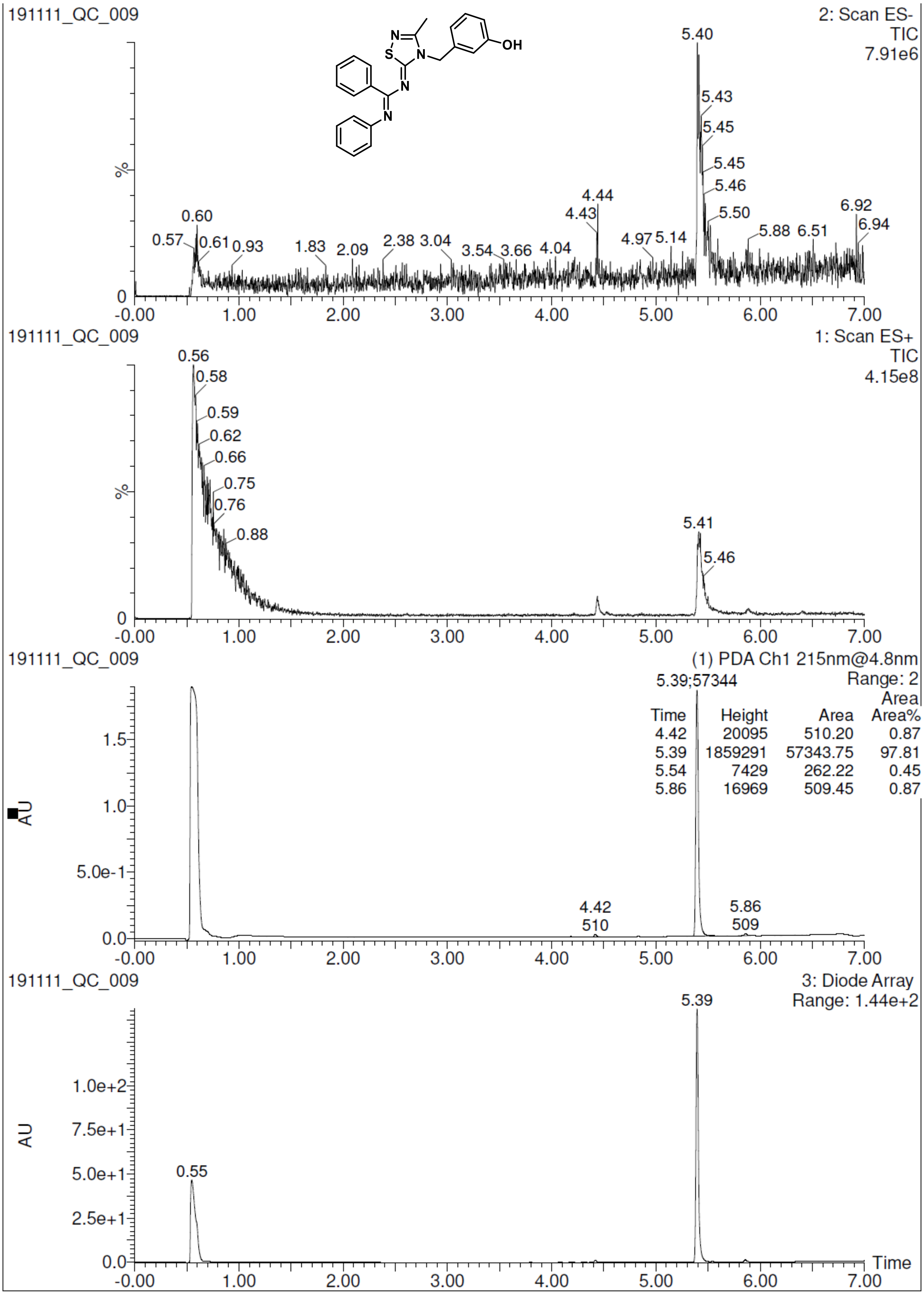

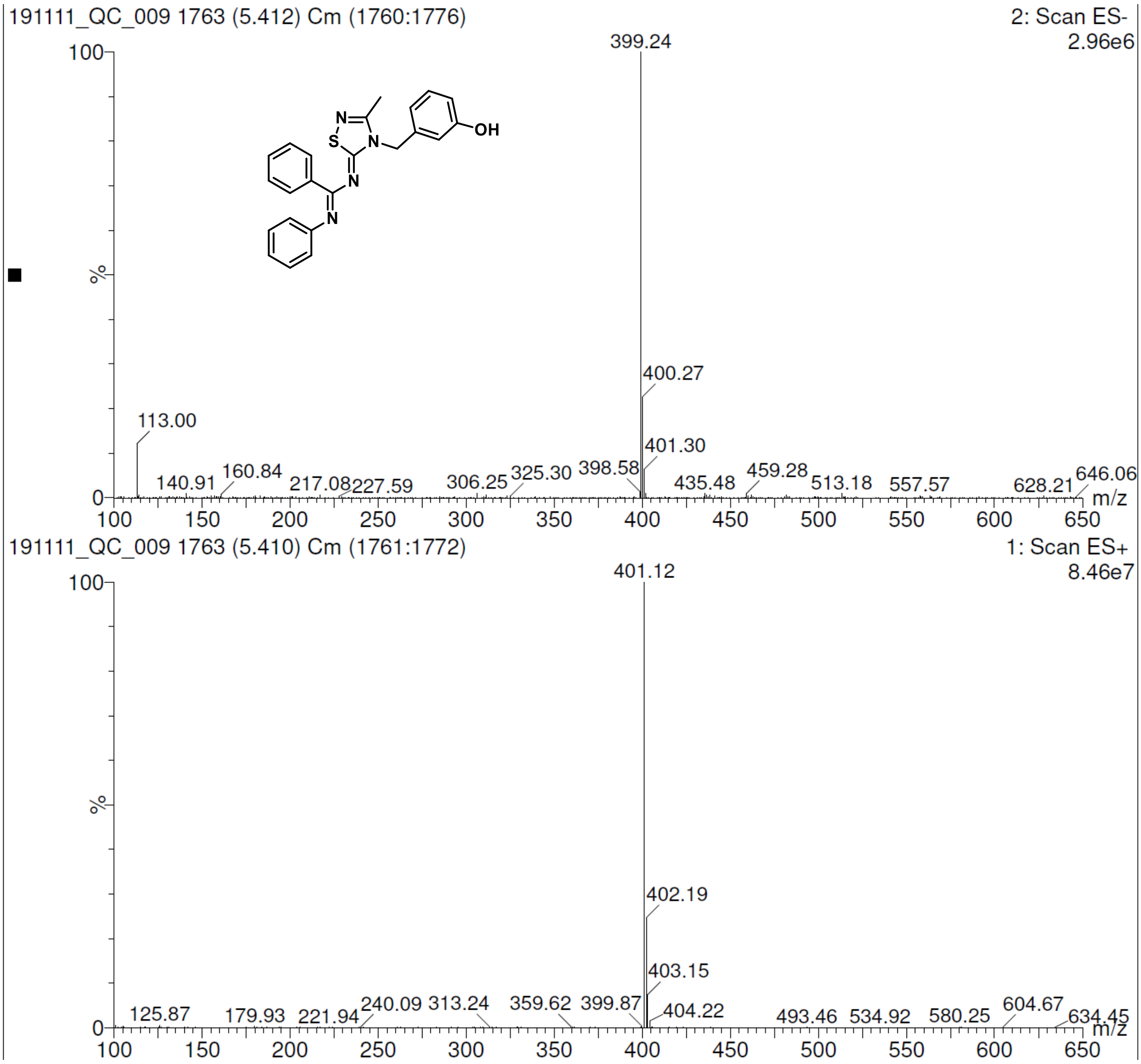
191111_QC_009 1763 (5.412) Cm (1760:1776)
2: Scan ES-
2.96e6
399.24
OH
N
S
N
N
N
400.27
113.00
401.30
140.91
160.84
217.08
227.59
306.25
325.30
398.58
435.48
459.28
513.18
557.57
628.21
646.06
100
%
0
m/z
100 150 200 250 300 350 400 450 500 550 600 650
191111_QC_009 1763 (5.410) Cm (1761:1772)
1: Scan ES+
8.46e7
401.12
402.19
403.15
125.87
179.93
221.94
240.09
313.24
359.62
399.87
404.22
493.46
534.92
580.25
604.67
634.45
100
%
0
m/z
100 150 200 250 300 350 400 450 500 550 600 650

HPLC-MS analysis of **19**

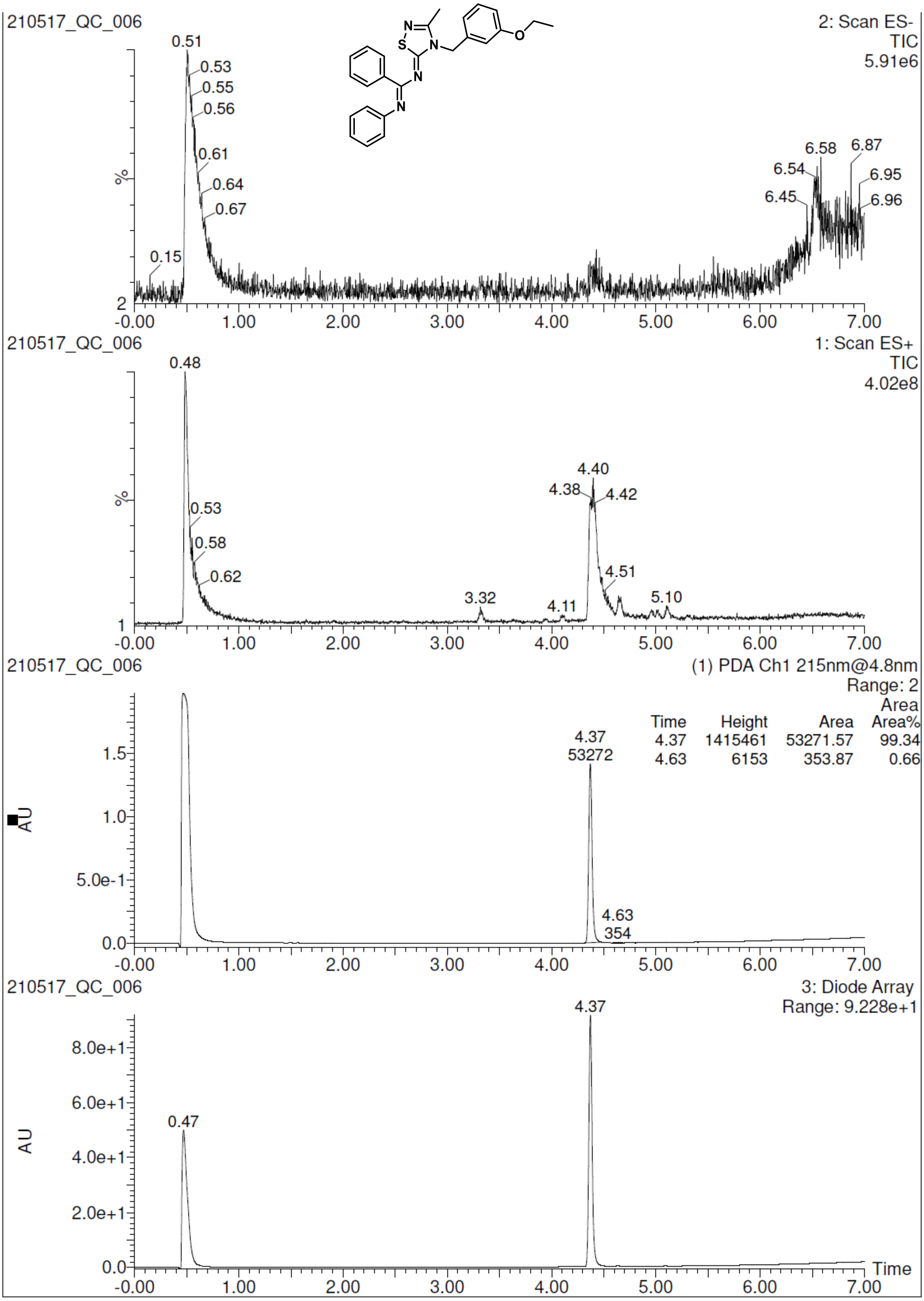

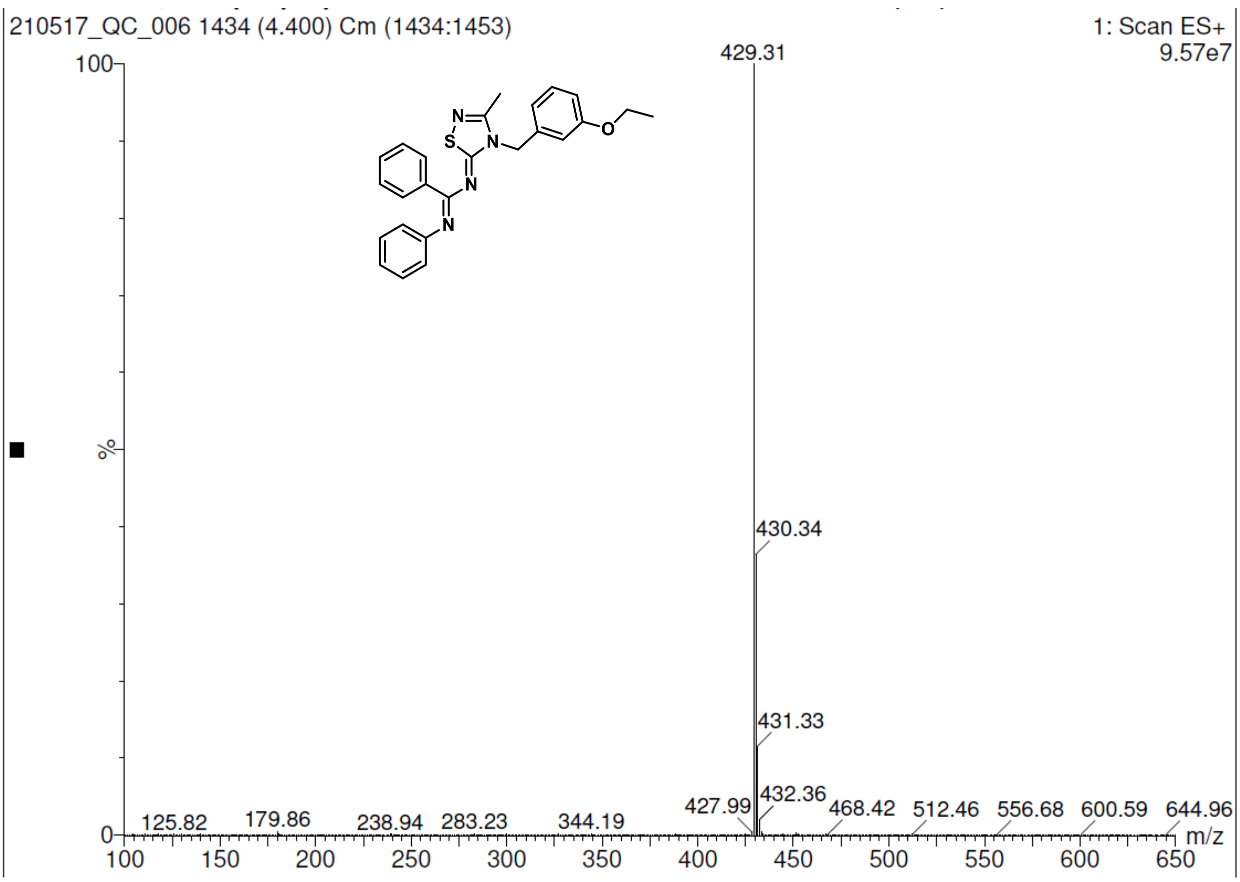
210517_QC_006 1434 (4.400) Cm (1434:1453)
1: Scan ES+
9.57e7
100
%
0
429.31
430.34
431.33
432.36
427.99
125.82
179.86
238.94
283.23
344.19
468.42
512.46
556.68
600.59
644.96
m/z
100
150
200
250
300
350
400
450
500
550
600
650
N
S
N
N
N
O

HPLC-MS analysis of **21**

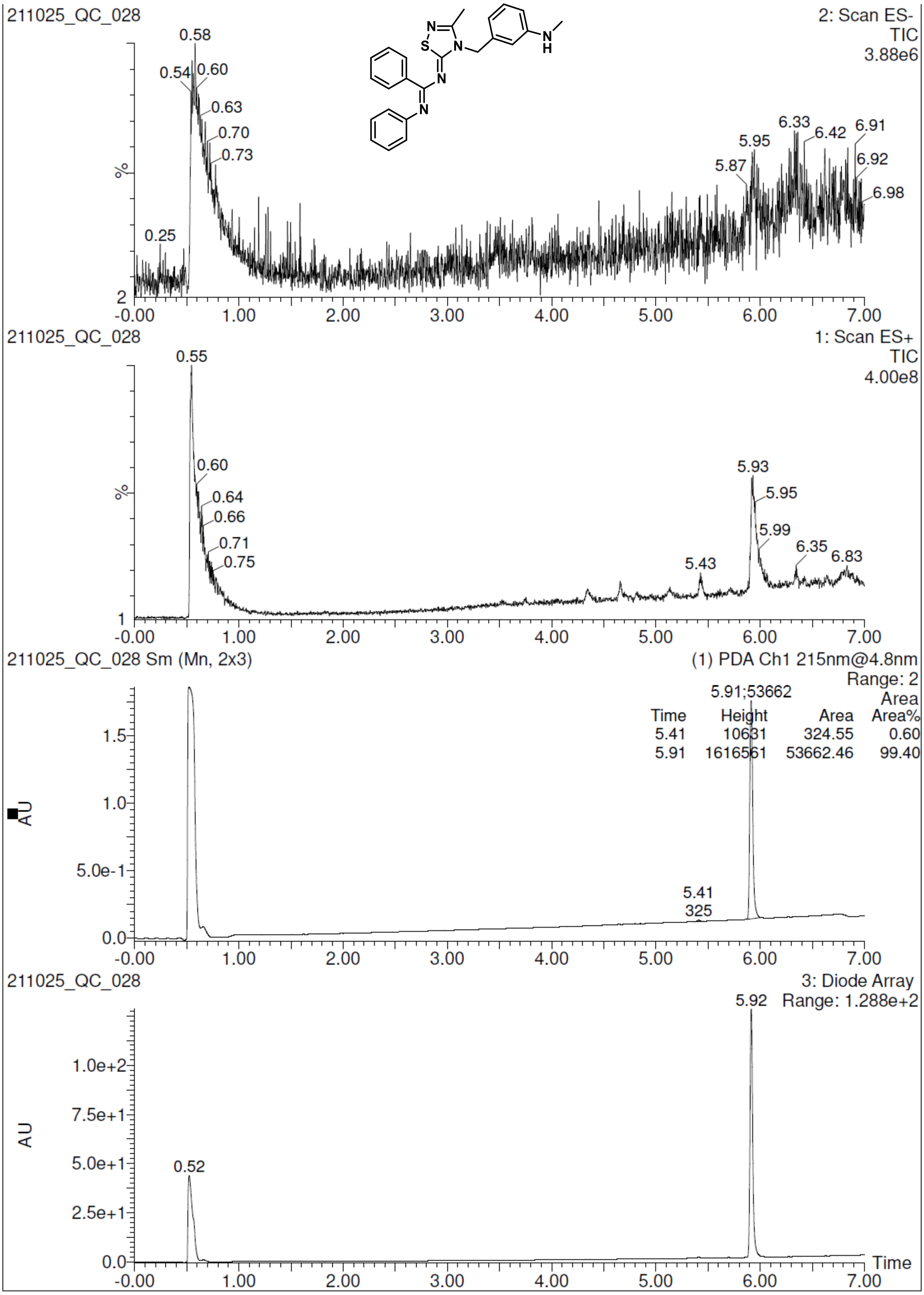

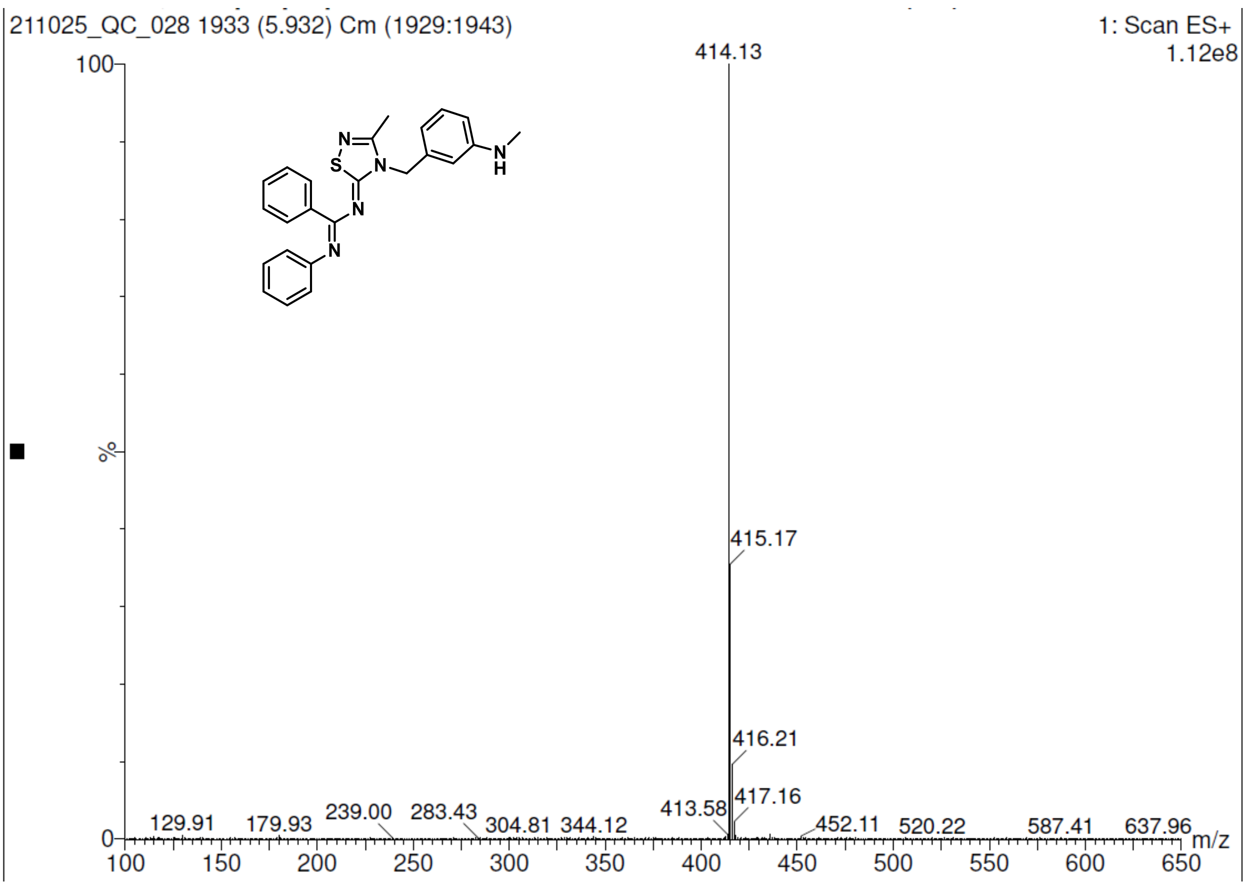

211025_QC_028 1933 (5.932) Cm (1929:1943)
1: Scan ES+
1.12e8
100
%
0
414.13
415.17
416.21
417.16
413.58
129.91
179.93
239.00
283.43
304.81
344.12
452.11
520.22
587.41
637.96
m/z
100
150
200
250
300
350
400
450
500
550
600
650
N
S
N
N
N
N
H

HPLC-MS analysis of **22**

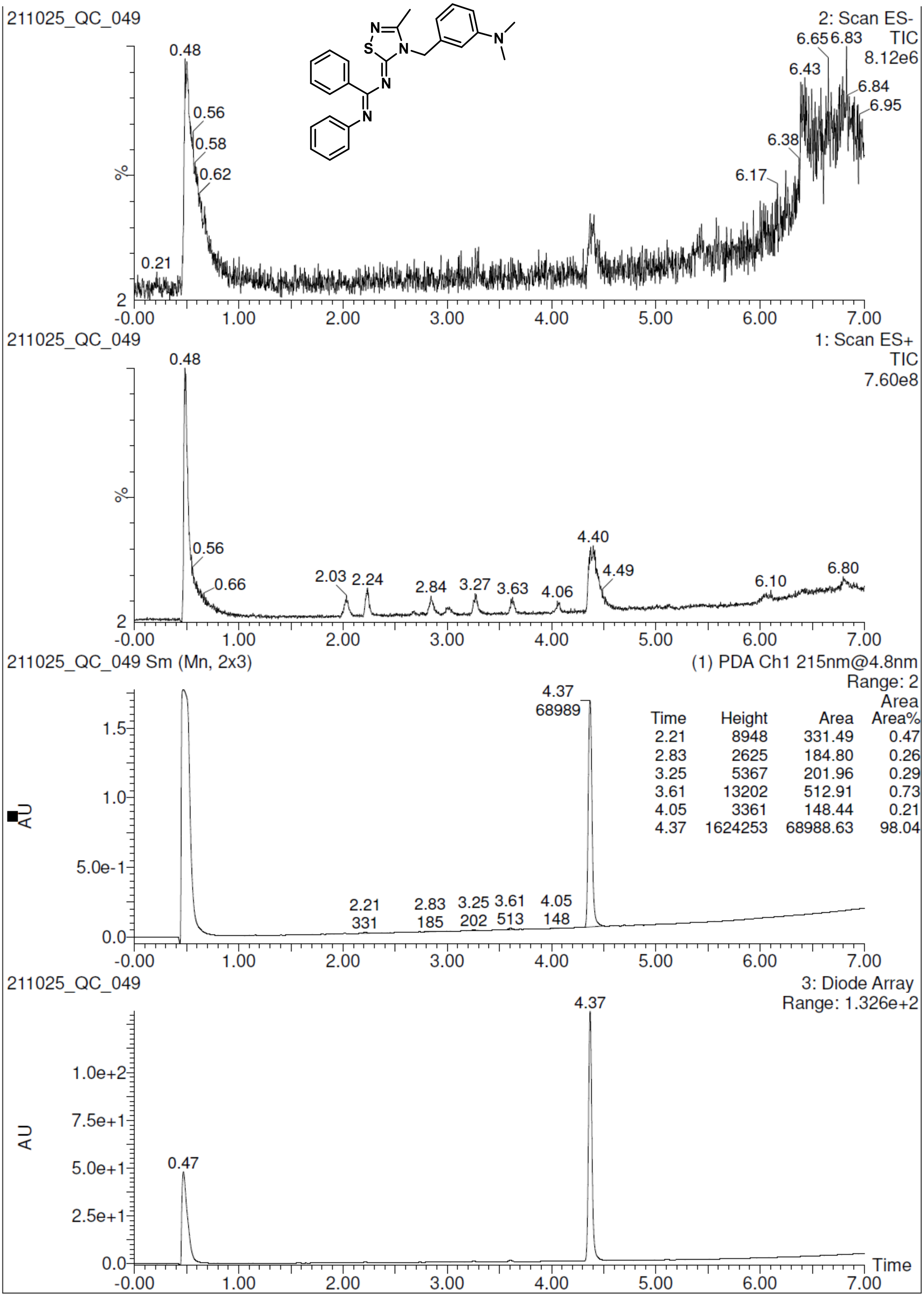

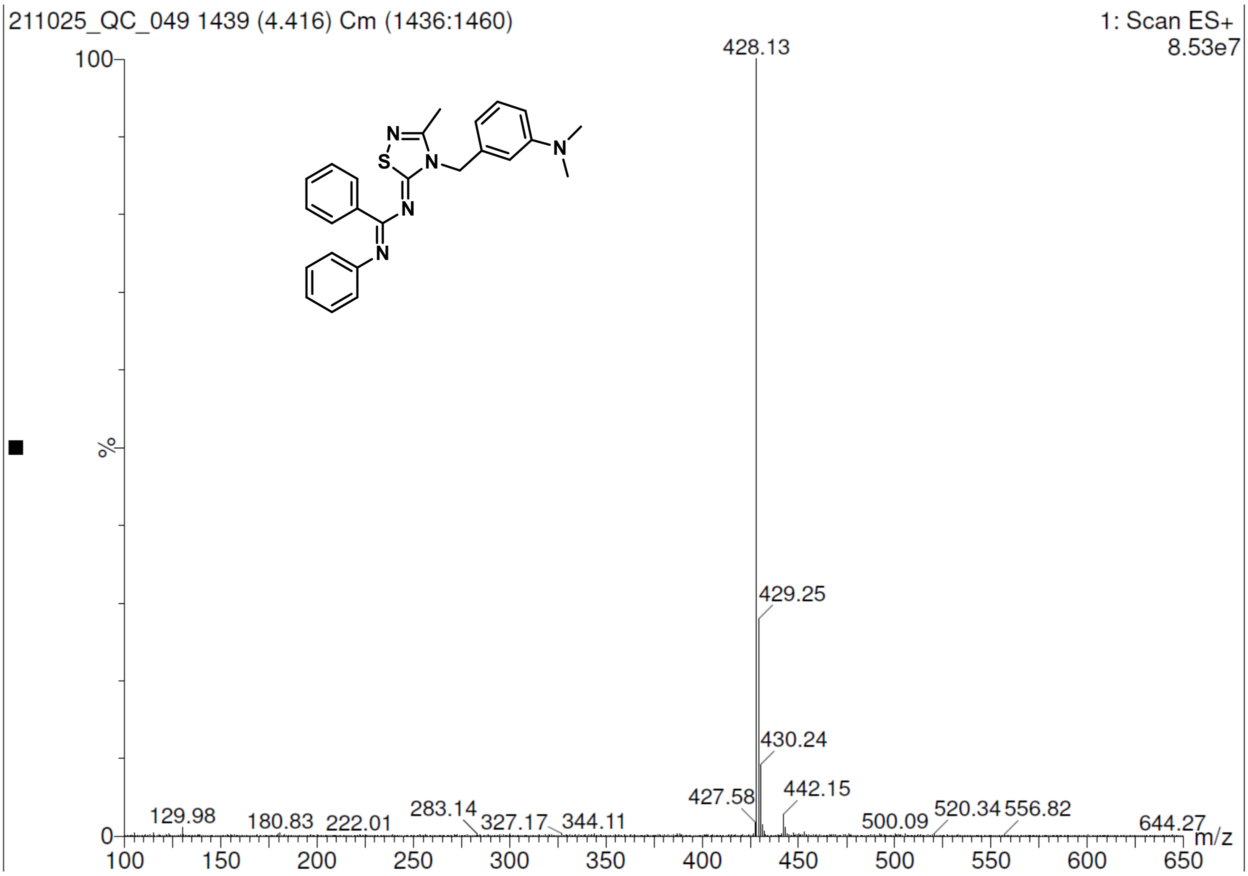
211025_QC_049 1439 (4.416) Cm (1436:1460)
1: Scan ES+
8.53e7
100
%
0
428.13
429.25
430.24
427.58
442.15
129.98
180.83
222.01
283.14
327.17
344.11
500.09
520.34
556.82
644.27
m/z
100
150
200
250
300
350
400
450
500
550
600
650
N
S
N
N
N
N

HPLC-MS analysis of **23**

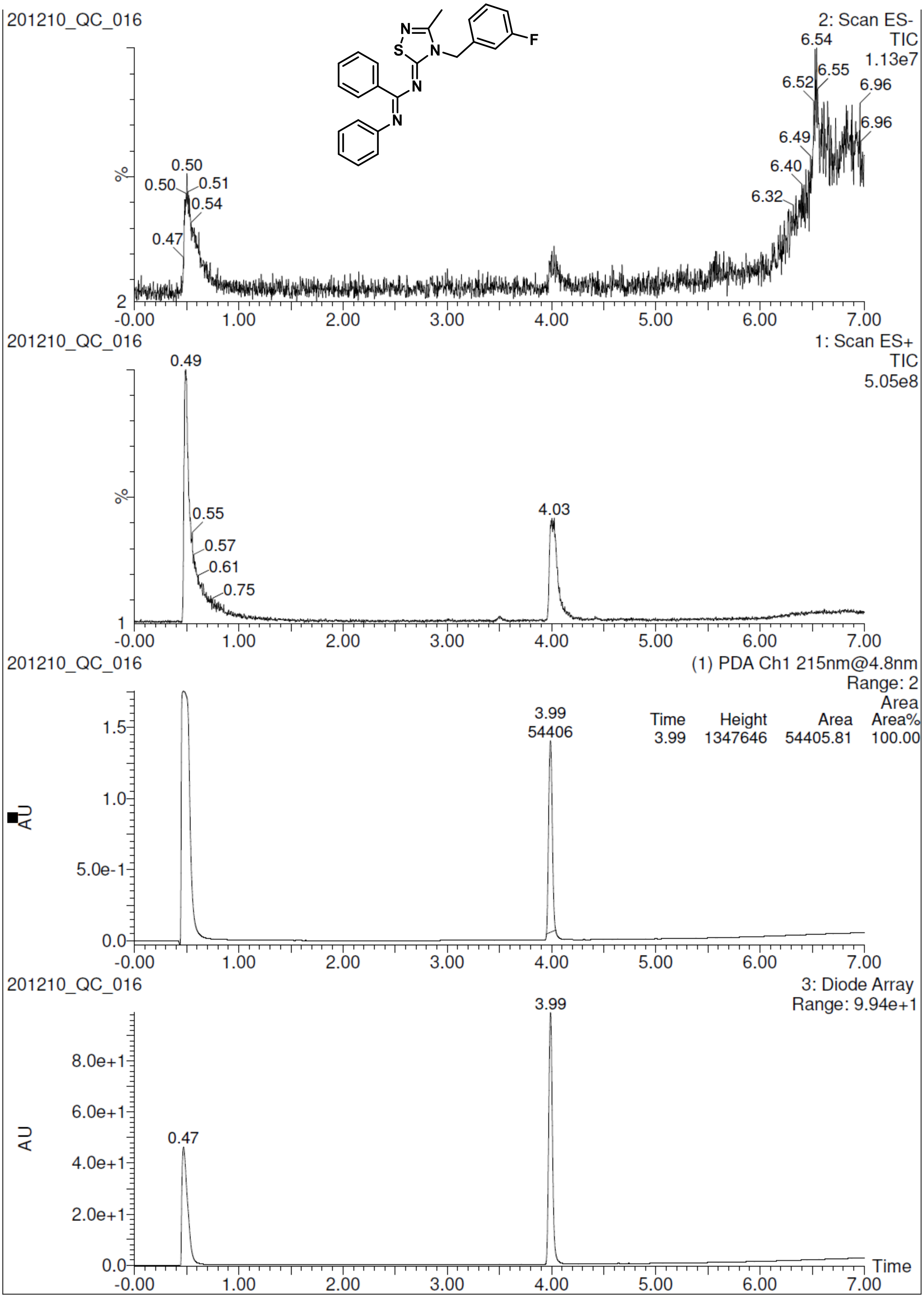

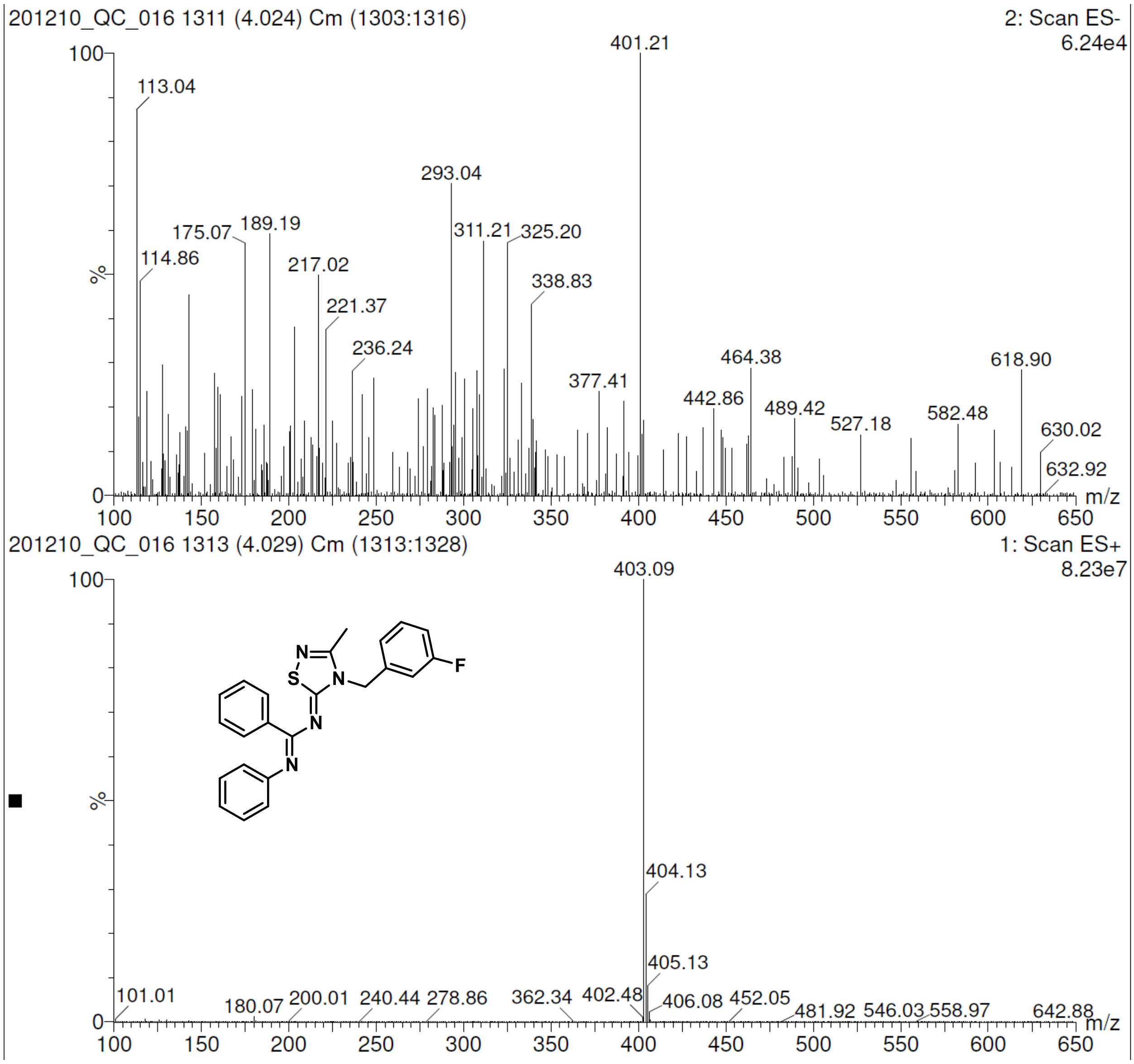
201210_QC_016 1311 (4.024) Cm (1303:1316)
2: Scan ES-
6.24e4
401.21
113.04
293.04
175.07
189.19
311.21
325.20
114.86
217.02
338.83
221.37
236.24
377.41
464.38
442.86
489.42
527.18
582.48
618.90
630.02
632.92
m/z
%
201210_QC_016 1313 (4.029) Cm (1313:1328)
1: Scan ES+
8.23e7
403.09
404.13
405.13
101.01
180.07
200.01
240.44
278.86
362.34
402.48
406.08
452.05
481.92
546.03
558.97
642.88
N
S
N
F
N
N

HPLC-MS analysis of **24**

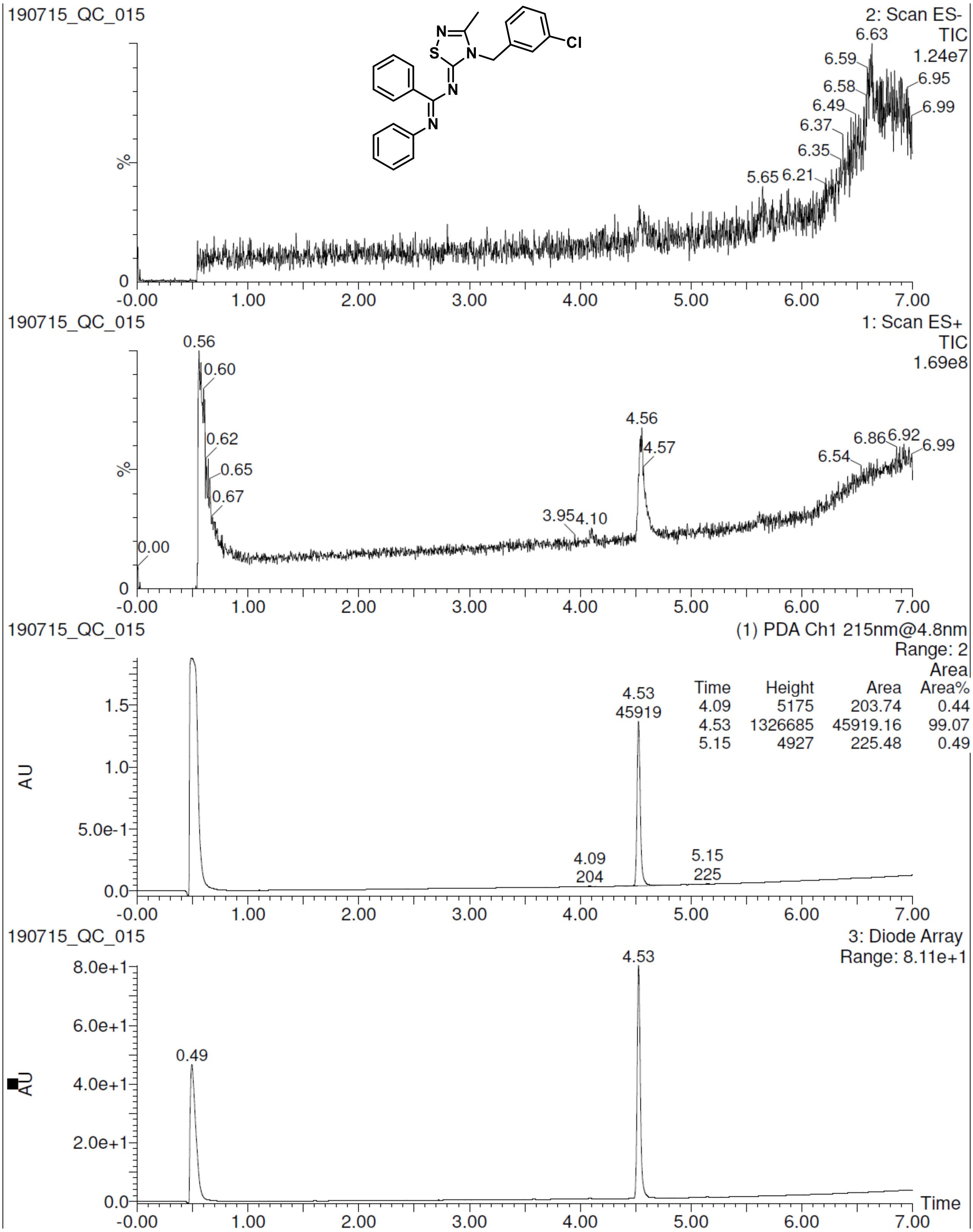

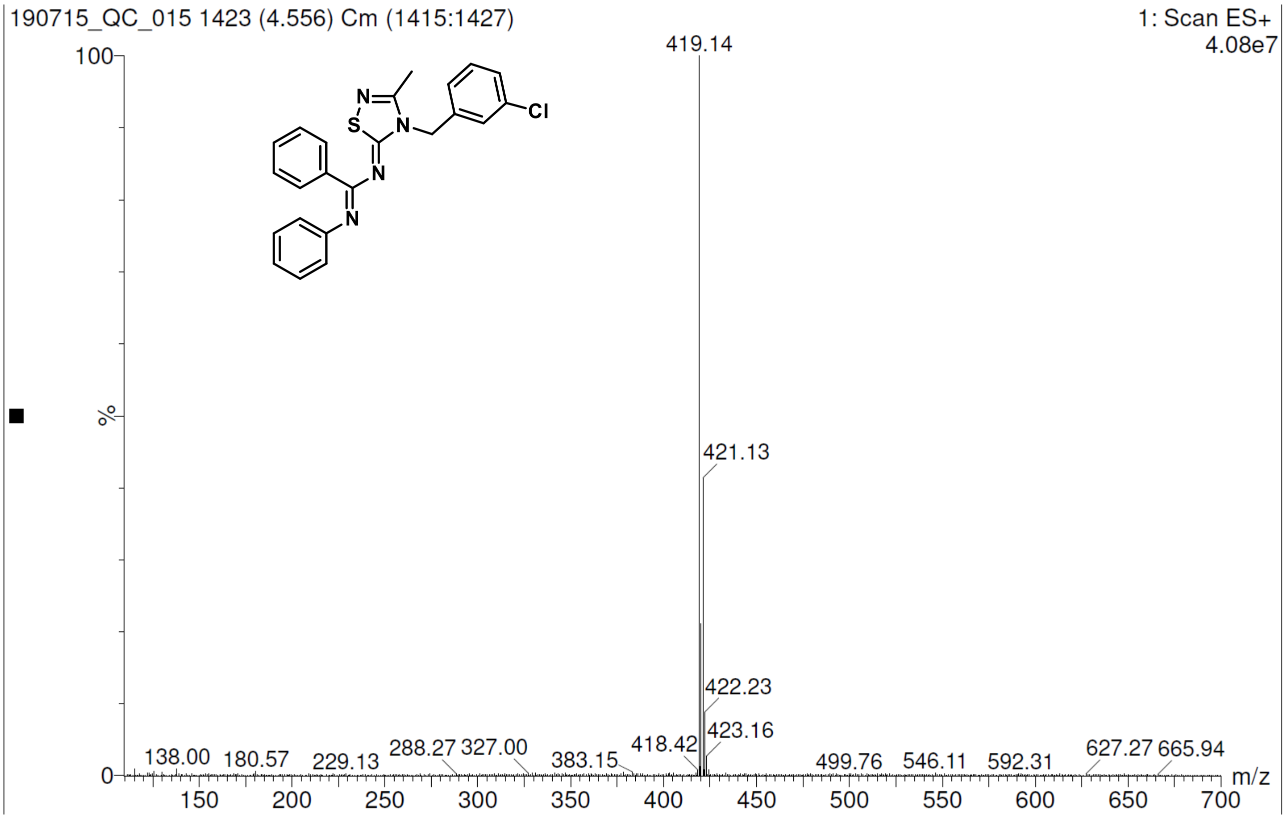
190715_QC_015 1423 (4.556) Cm (1415:1427)
1: Scan ES+
4.08e7
419.14
421.13
422.23
423.16
418.42
138.00
180.57
229.13
288.27
327.00
383.15
499.76
546.11
592.31
627.27
665.94
100
%
0
150
200
250
300
350
400
450
500
550
600
650
700
m/z
N
S
N
N
N
Cl

HPLC-MS analysis of **25**

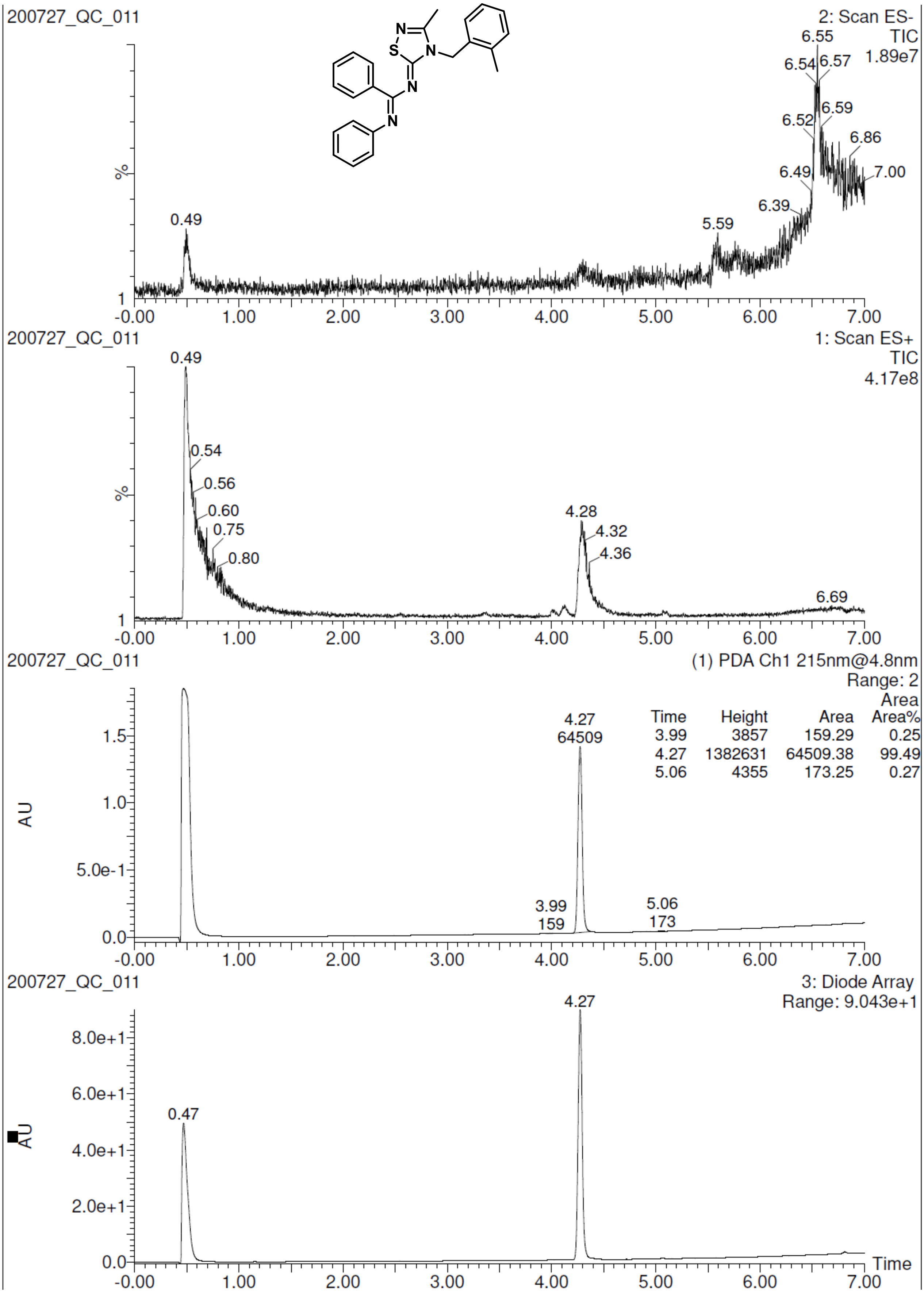

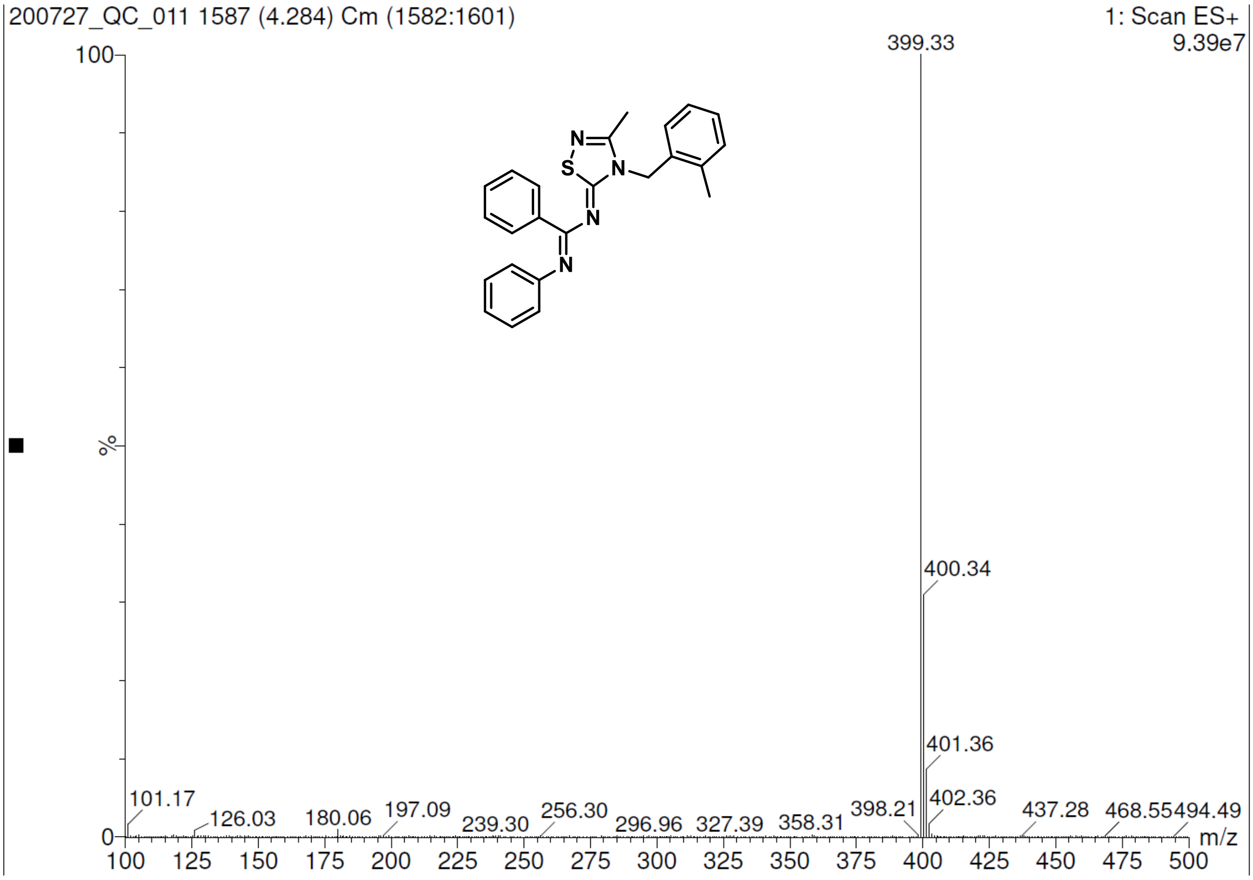
200727_QC_011 1587 (4.284) Cm (1582:1601)
1: Scan ES+
9.39e7
399.33
400.34
401.36
402.36
101.17
126.03
180.06
197.09
239.30
256.30
296.96
327.39
358.31
398.21
437.28
468.55
494.49
100
%
0
m/z
100 125 150 175 200 225 250 275 300 325 350 375 400 425 450 475 500

HPLC-MS analysis of **27**

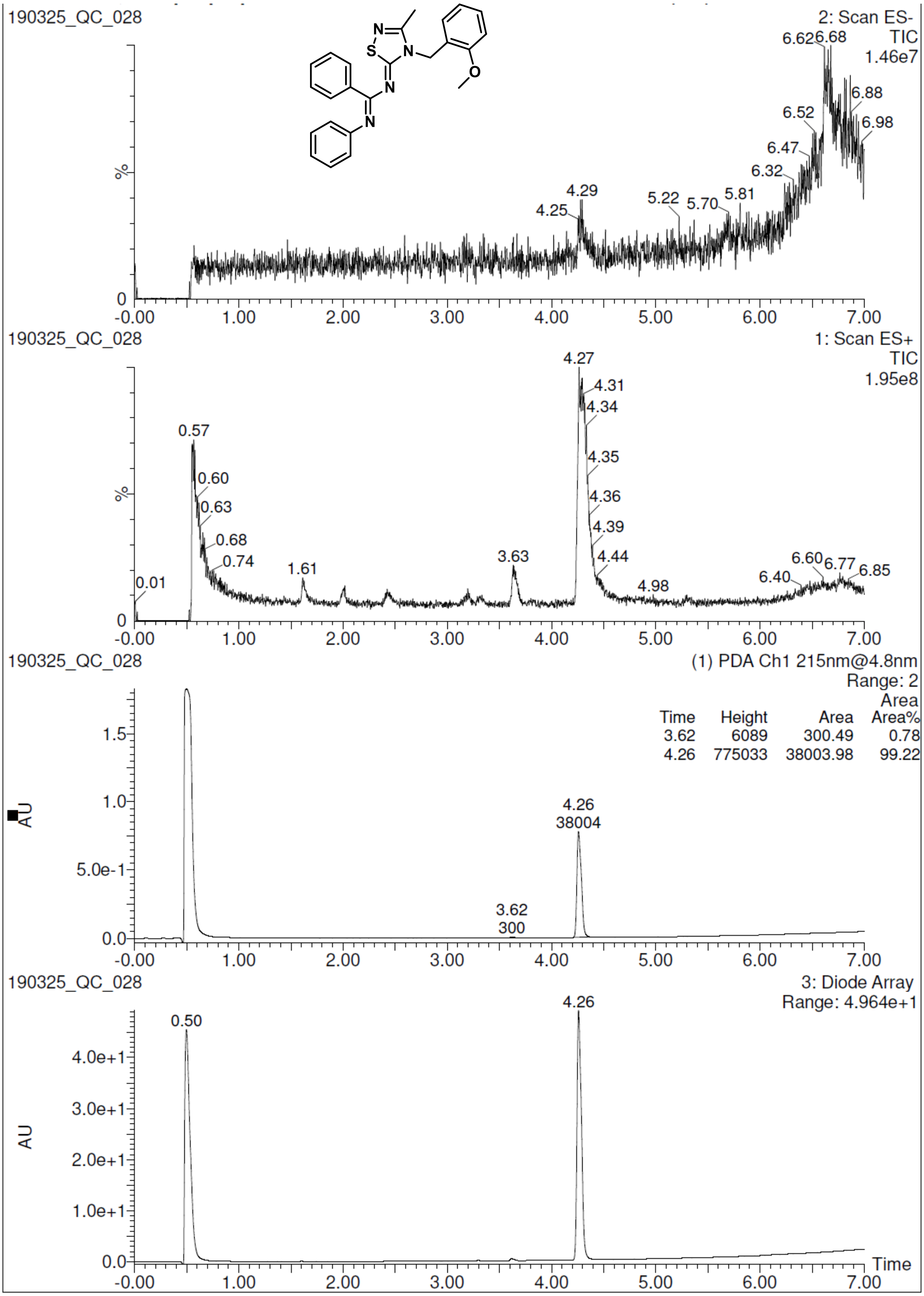

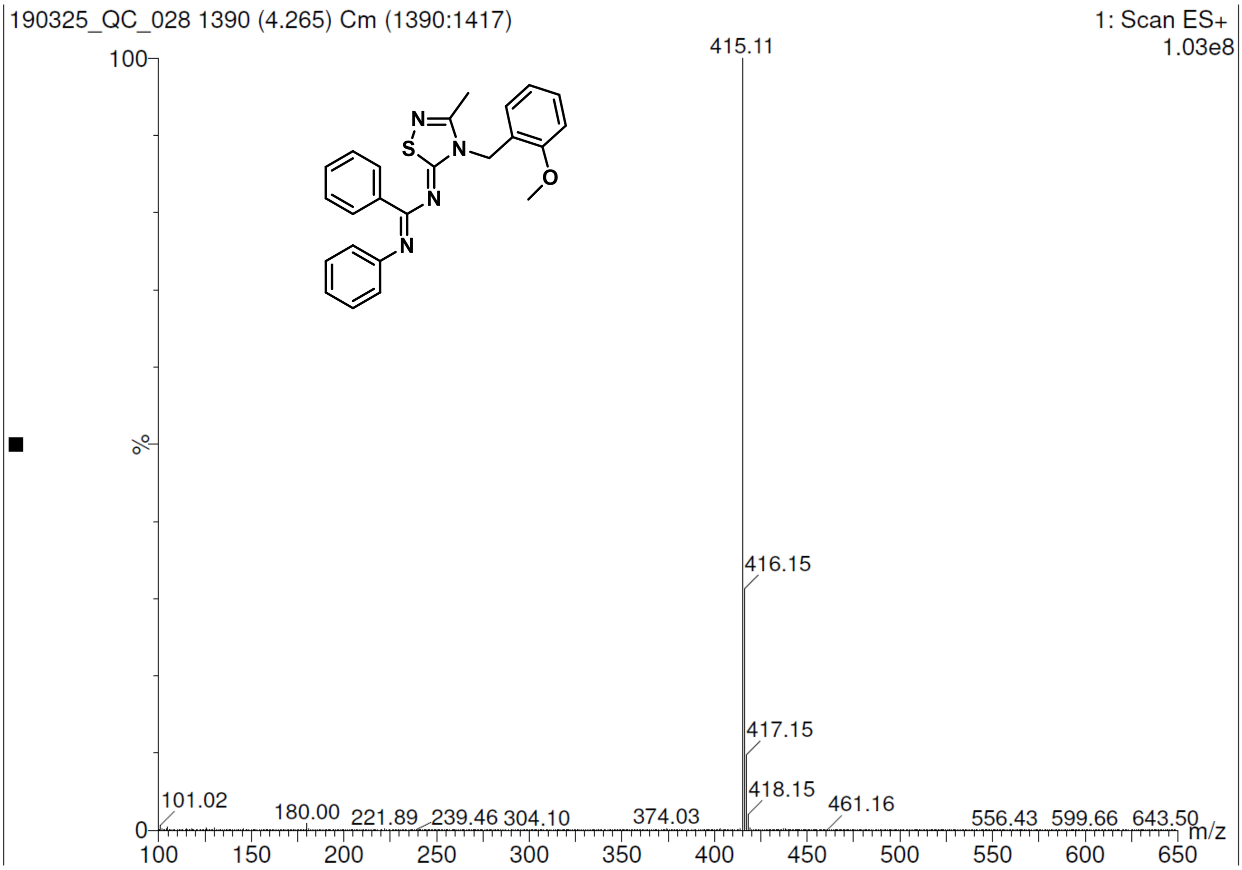
190325_QC_028 1390 (4.265) Cm (1390:1417)
1: Scan ES+
1.03e8
415.11
416.15
417.15
418.15
461.16
101.02
180.00
221.89
239.46
304.10
374.03
556.43
599.66
643.50
m/z
%
100
0
N
S
N
N
N
O

HPLC-MS analysis of **28**

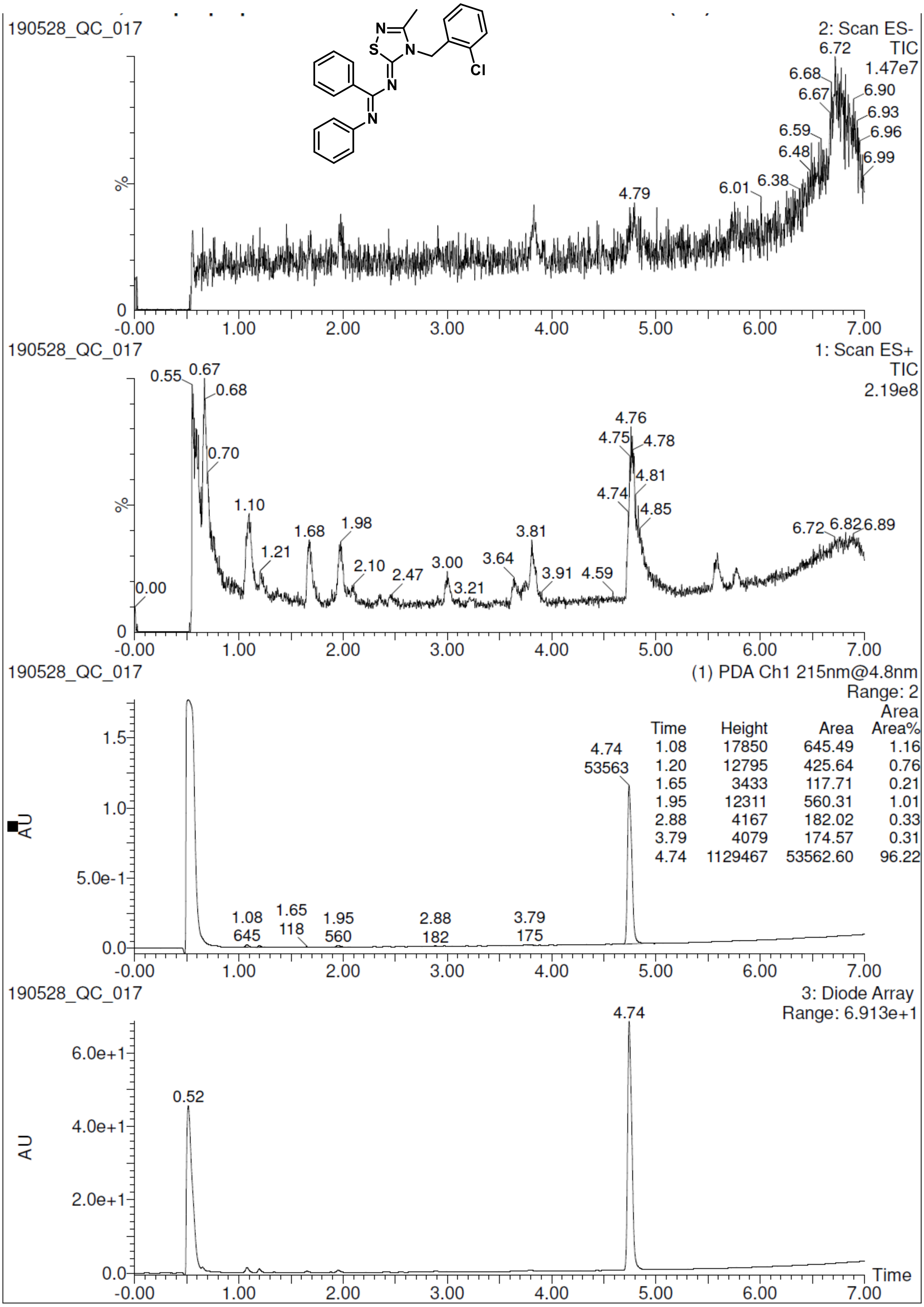

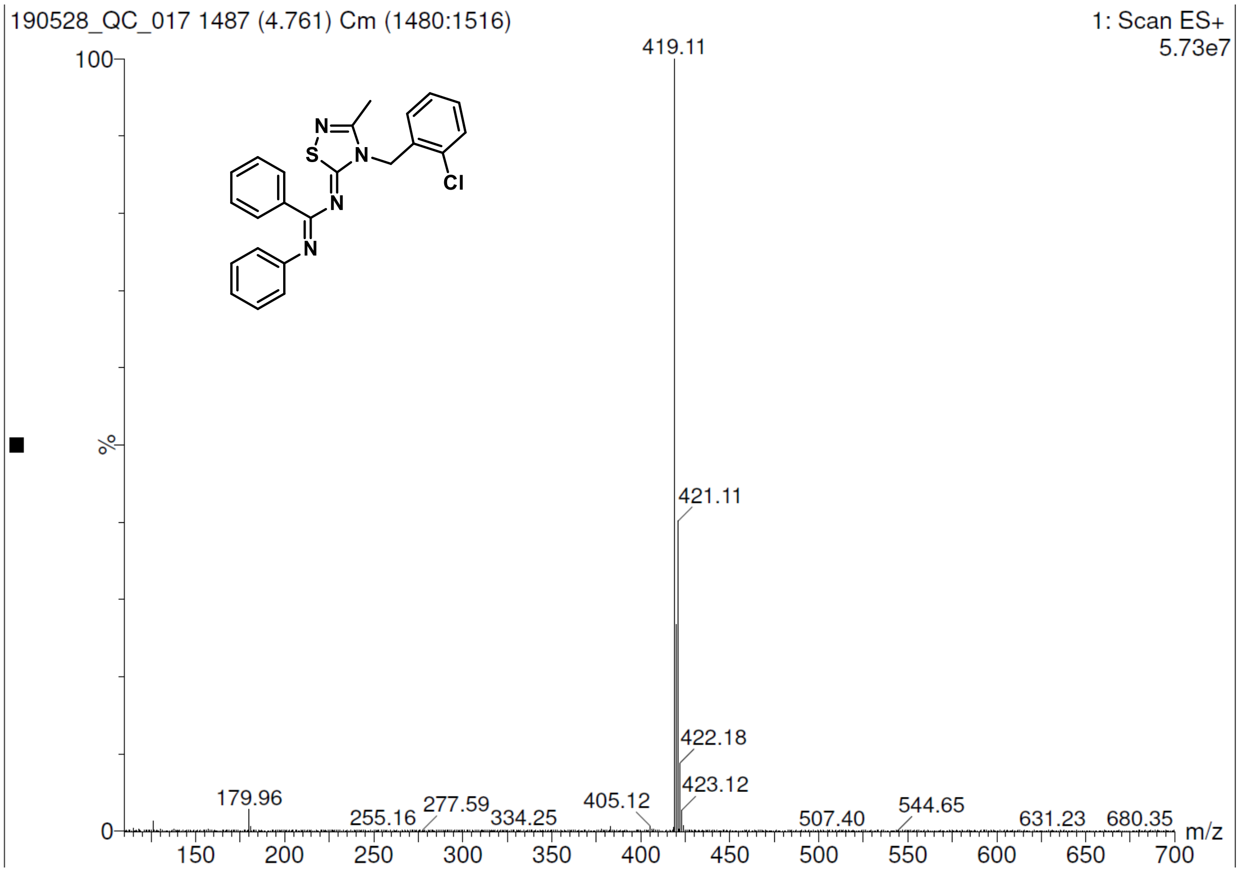

190528_QC_017 1487 (4.761) Cm (1480:1516)
1: Scan ES+
5.73e7
100
%
0
419.11
421.11
422.18
423.12
179.96
255.16
277.59
334.25
405.12
507.40
544.65
631.23
680.35
m/z
150
200
250
300
350
400
450
500
550
600
650
700
N
S
N
N
N
Cl

HPLC-MS analysis of **29**

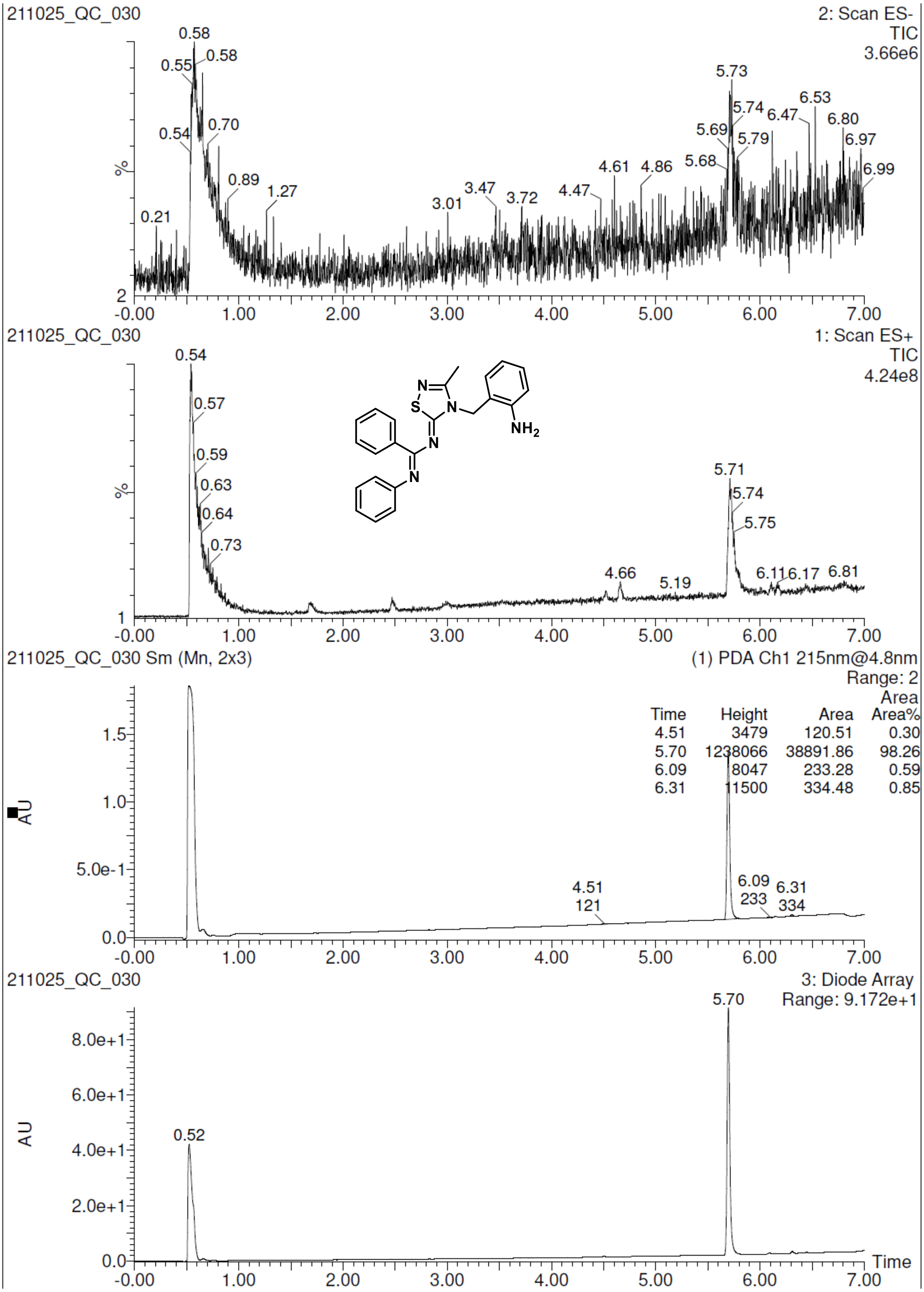

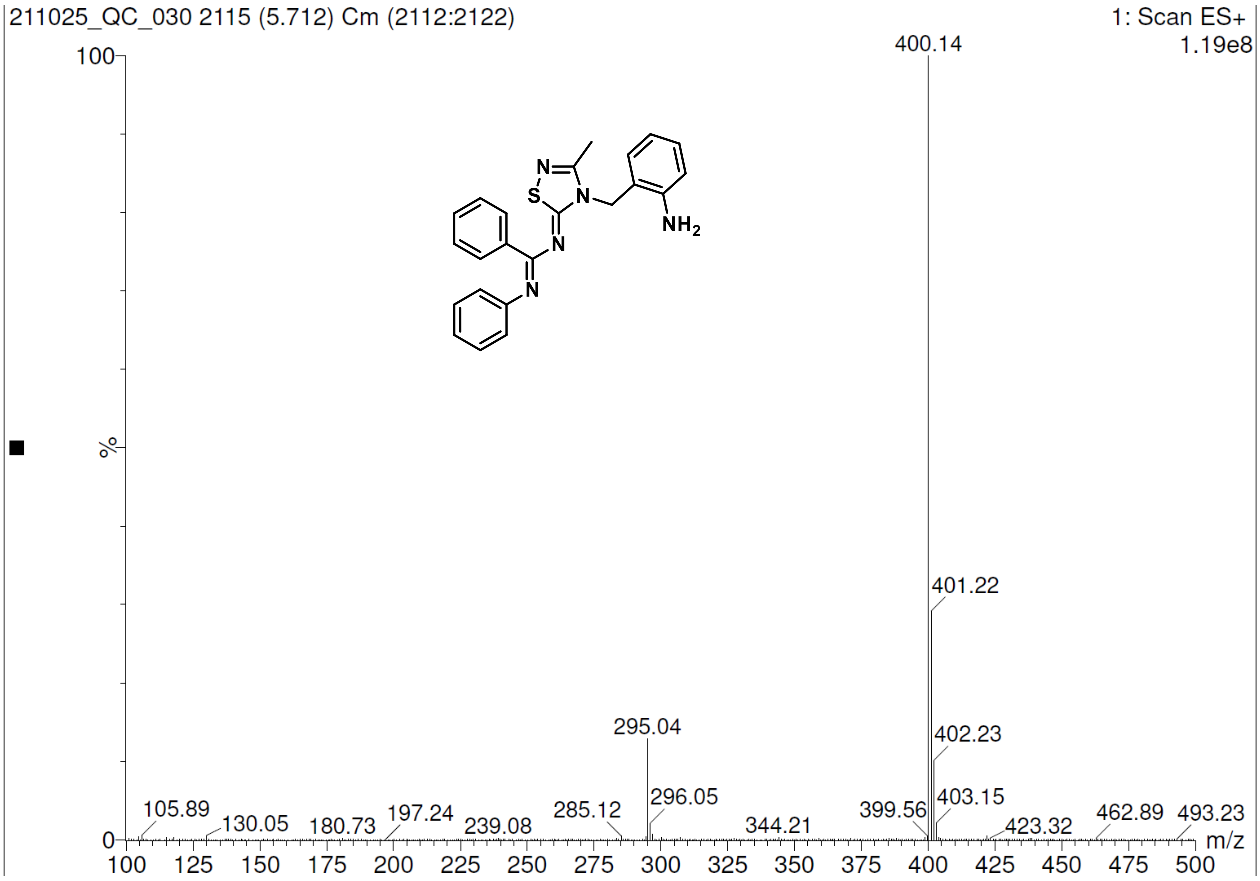
211025_QC_030 2115 (5.712) Cm (2112:2122)
1: Scan ES+
1.19e8
400.14
100
%
0
401.22
295.04
402.23
296.05
403.15
105.89
130.05
180.73
197.24
239.08
285.12
344.21
399.56
423.32
462.89
493.23
m/z
100 125 150 175 200 225 250 275 300 325 350 375 400 425 450 475 500
N
S
N
N
N
NH2

HPLC-MS analysis of **34**

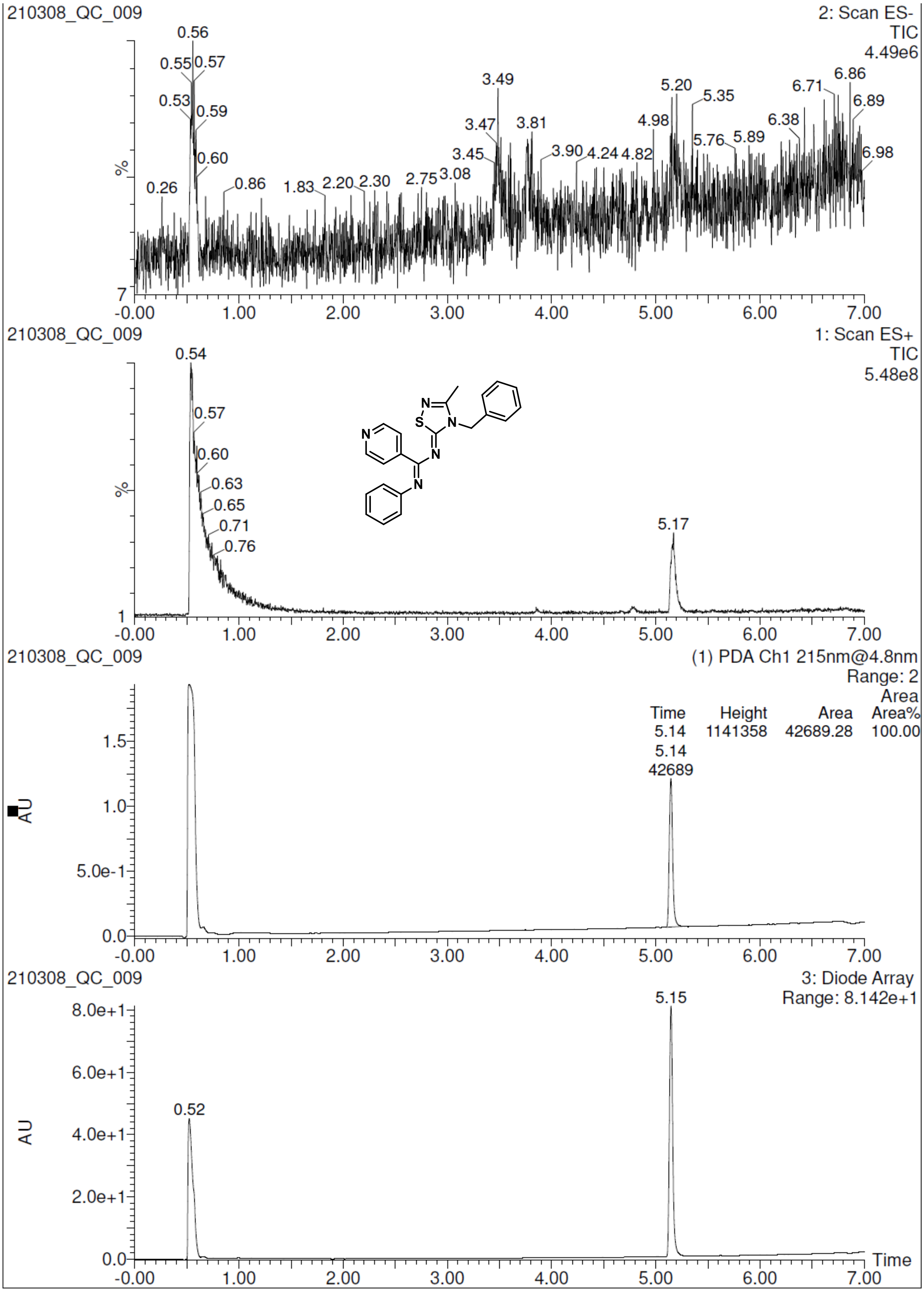

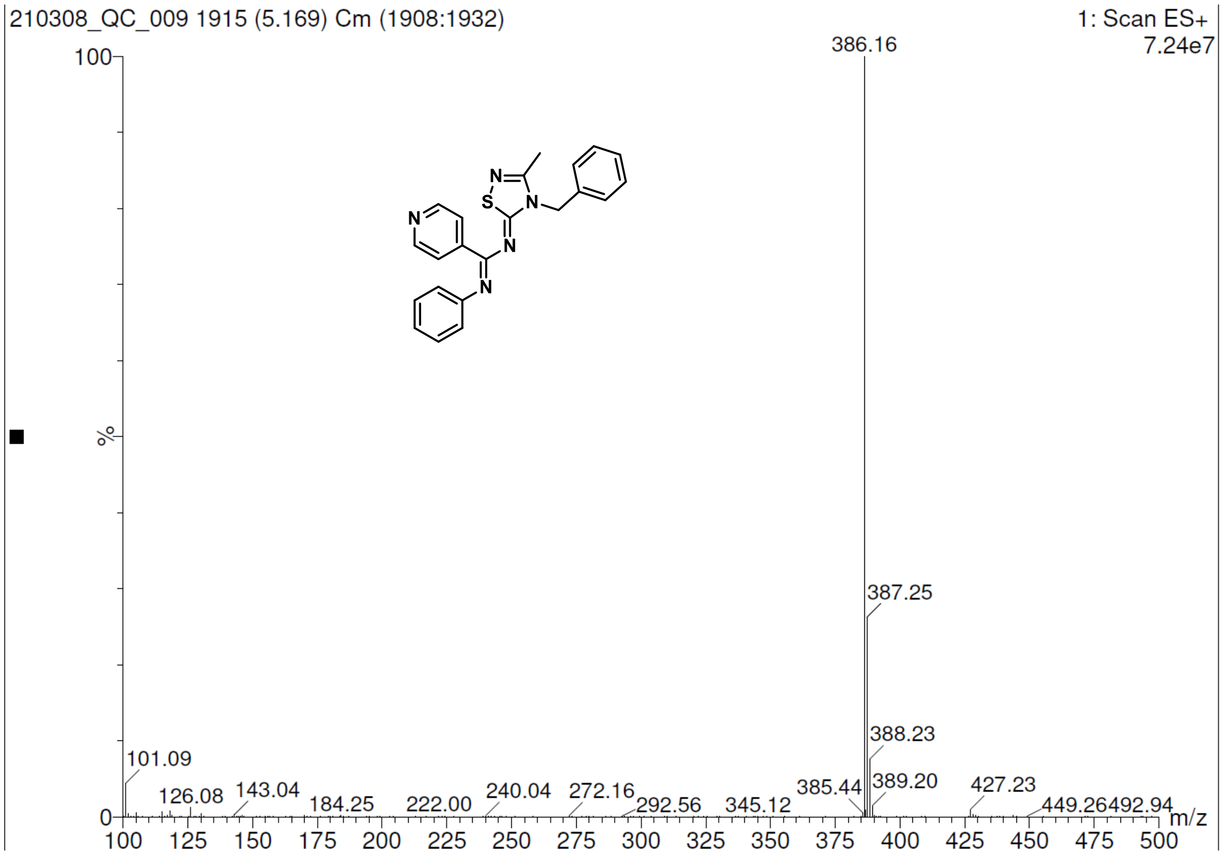

References

1. Sondo, E.; Falchi, F.; Caci, E.; Ferrera, L.; Giacomini, E.; Pesce, E.; Tomati, V.; Mandrup Bertozzi, S.; Goldoni, L.; Armirotti, A.; Ravazzolo, R.; Cavalli, A.; Pedemonte, N. Pharmacological Inhibition of the Ubiquitin Ligase RNF5 Rescues F508del-CFTR in Cystic Fibrosis Airway Epithelia. *Cell Chem. Biol.* **2018,** *25*, 891-905.e898.

2. Friesner, R. A.; Murphy, R. B.; Repasky, M. P.; Frye, L. L.; Greenwood, J. R.; Halgren,T. A.; Sanschagrin, P. C.; Mainz, D. T. Extra Precision Glide: Docking and Scoring Incorporating a Model of Hydrophobic Enclosure for Protein-Ligand Complexes. *J. Med. Chem.* **2006**, *49*, 6177–6196.

3. Greenwood, J. R.; Calkins, D.; Sullivan, A. P.; Shelley, J. C. Towards the comprehensive, rapid, and accurate prediction of the favorable tautomeric states of drug-like molecules in aqueous solution. *J. Comput. Aided Mol. Des.* **2010**, *24*, 591-604.
